# Physiological stress improves stem cell modeling of dystrophic cardiomyopathy

**DOI:** 10.1242/dmm.050487

**Published:** 2024-02-05

**Authors:** Dominic E. Fullenkamp, Alexander B. Willis, Jodi L. Curtin, Ansel P. Amaral, Kyle T. Dittloff, Sloane I. Harris, Ivana A. Chychula, Cory W. Holgren, Paul W. Burridge, Brenda Russell, Alexis R. Demonbreun, Elizabeth M. McNally

**Affiliations:** ^1^ Center for Genetic Medicine, Feinberg School of Medicine, Northwestern University, Chicago, IL 60611, USA; ^2^ Division of Cardiology, Department of Medicine, Feinberg School of Medicine, Northwestern University, Chicago, IL 60611, USA; ^3^ Department of Physiology and Biophysics, University of Illinois at Chicago, Chicago, IL 60612, USA; ^4^ Department of Pharmacology, Feinberg School of Medicine, Northwestern University, Chicago, IL 60611, USA

**Keywords:** Membrane, Annexin, Sarcolemma, Cardiomyocyte, Human induced pluripotent stem cell-derived cardiomyocytes, Duchenne muscular dystrophy

## Abstract

Heart failure contributes to Duchenne muscular dystrophy (DMD), which arises from mutations that ablate dystrophin, rendering the plasma membrane prone to disruption. Cardiomyocyte membrane breakdown in patients with DMD yields a serum injury profile similar to other types of myocardial injury with the release of creatine kinase and troponin isoforms. Human induced pluripotent stem cell-derived cardiomyocytes (hiPSC-CMs) are highly useful but can be improved. We generated hiPSC-CMs from a patient with DMD and subjected these cells to equibiaxial mechanical strain to mimic *in vivo* stress. Compared to healthy cells, DMD hiPSC-CMs demonstrated greater susceptibility to equibiaxial strain after 2 h at 10% strain. We generated an aptamer-based profile of proteins released from hiPSC-CMs both at rest and subjected to strain and identified a strong correlation in the mechanical stress-induced proteome from hiPSC-CMs and serum from patients with DMD. We exposed hiPSC-CMs to recombinant annexin A6, a protein resealing agent, and found reduced biomarker release in DMD and control hiPSC-CMs subjected to strain. Thus, the application of mechanical strain to hiPSC-CMs produces a model that reflects an *in vivo* injury profile, providing a platform to assess pharmacologic intervention.

## Teaser

**Editor's choice:** Applying physiological mechanical stress to a human induced pluripotent stem cell model of Duchenne muscular dystrophy-related cardiomyopathy produces a biomarker signature similar to that seen in patients.

**Research Simplified**

The heart is a muscle, which means that patients with muscle-wasting diseases, such as Duchenne muscular dystrophy (DMD), often experience heart problems, known as cardiomyopathy. Although researchers have made advances that improve the health of these patients, developing new drugs to specifically help their hearts is exceedingly difficult. Growing heart tissue in a dish allows researchers to test new therapies, but the tissue must behave as similarly to the organ as possible, including mimicking heartbeats.

The authors of this study engineered cells from a patient with DMD to make them resemble heart muscle cells and grew them on a stretchy membrane. Each stretch of this membrane mimicked a heartbeat. The authors found that engineered heart muscle cells from the patient became injured in response to these ‘heartbeats’ much faster than cells from an unaffected individual. They also found that cells from the patient secrete molecules in response to this injury that are similar to those found in the bloodstream of patients with DMD experiencing cardiomyopathy. This similarity means that these heart muscle cells grown on a stretchy membrane mimic important features of the diseased heart and could thus be used to test drugs that could prevent or treat cardiomyopathy.

## INTRODUCTION

Duchenne muscular dystrophy (DMD) is an X-linked disease that results from mutations in the *DMD* gene, which codes for the protein dystrophin (Koenig et al., 1987). Clinically, DMD presents in the first decade with weakness and markedly elevated serum biomarkers, including creatine kinase (Bushby et al., 2010). Cardiac involvement, although variable in onset and progression, is typically evident by the second decade and contributes to morbidity and mortality in DMD (McNally et al., 2015). In heart and skeletal muscle, dystrophin localizes to the plasma membrane and is concentrated in the membrane above the Z-disc, colocalizing with other proteins of the dystrophin complex, including the sarcoglycans and dystroglycans (Campbell and Kahl, 1989; Ervasti and Campbell, 1991; Ervasti et al., 1991). This complex forms a critical transmembrane structural and signaling connection between the sarcomere and the extracellular matrix (Briggs et al., 2016; Campbell and Kahl, 1989; Ibraghimov-Beskrovnaya et al., 1992; Klietsch et al., 1993; Rybakova et al., 2000). Disruptions along this axis produce membrane fragility and account for multiple forms of muscular dystrophy with cardiac involvement (Bloch and Gonzalez-Serratos, 2003; Ervasti, 2003; Townsend et al., 2011). Early initiation of angiotensin-converting enzyme (ACE) inhibitors slows the progression of the cardiomyopathy (Duboc et al., 2005, 2007; Silva et al., 2017), and cardiomyopathy treatment and heart failure management in DMD largely relies on guideline-directed heart failure strategies (Buddhe et al., 2018; Feingold et al., 2017; McNally et al., 2015; Yancy et al., 2017). Antisense-mediated exon skipping agents are now approved for use in DMD, but these agents have relatively poor penetration into the myocardium and are useful for less than 25% of DMD mutations (Johnston and McNally, 2021; Sheikh and Yokota, 2022). Gene therapy with micro-dystrophin was recently approved for patients with DMD between the ages of 4 and 5 years (Reardon, 2023), but their durability and effect on the human heart are not known. Novel therapeutics for the treatment of DMD are currently under investigation, including additional gene replacement therapy with micro-dystrophins, gene editing approaches and membrane re-sealants (Duan, 2018; Hauck et al., 2019; Houang et al., 2018; Kyrychenko et al., 2017; Lowe et al., 2020; Yasuda et al., 2005). For clinical agents treating skeletal muscle in DMD, most studies have relied on endpoints such as time to loss of mobility or measures of muscle strength or performance (Ricci et al., 2022). Clinical trials for DMD cardiomyopathy are complicated by patients having reduced or no ambulatory capabilities (Johnston and McNally, 2021).

Human induced pluripotent stem cell-derived cardiomyocytes (hiPSC-CMs) can be used to evaluate patient-specific therapies in a human cell context (Sayed et al., 2016). hiPSC-CMs generated from patients with DMD (DMD hiPSC-CMs) have been shown to have an increased arrhythmia propensity (Kamdar et al., 2020), an increase in sensitivity to the local mechanical environment leading to altered contractility and telomere length (Chang et al., 2021) and altered calcium handling (Lin et al., 2015). DMD hiPSC-CMs have also been used to assess clinically relevant therapeutic strategies such as exon skipping (Dick et al., 2013) and CRISPR-based gene editing (Kyrychenko et al., 2017). However, hiPSC-CMs do not fully recapitulate the phenotype of adult cardiomyocytes and are generally cultured under conditions that fail to mimic the cyclic load and deformation seen by the human heart (Karakikes et al., 2015; Tu et al., 2018). Despite progress with tissue engineering methods, which can partially improve maturity (Breckwoldt et al., 2017; Stein et al., 2021), approaches to evaluate dynamic physiological mechanical stress are still under development. Studies using rat neonatal cardiomyocytes or mouse embryonic fibroblasts investigated the effects of mechanical stress to understand early signaling responses that lead to cardiac hypertrophy (Yamamoto et al., 2001) and pathological signaling responses in nuclear membrane defects (Lammerding et al., 2004). Here, we investigated the differential response of mechanical stress on DMD and healthy control hiPSC-CMs using an aptamer-based protein profiling system to characterize protein release at baseline and in response to mechanical stress, finding significant correlation with serum biomarkers from patients with DMD. Additionally, we evaluated the response to a resealing protein, recombinant annexin A6, which was previously identified as a genetic modifier of muscular dystrophy and a potential therapeutic target (Demonbreun et al., 2019; Swaggart et al., 2014).

## RESULTS

### Generation, differentiation and expansion of high-quality hiPSC-CMs

**Fig. 1.**
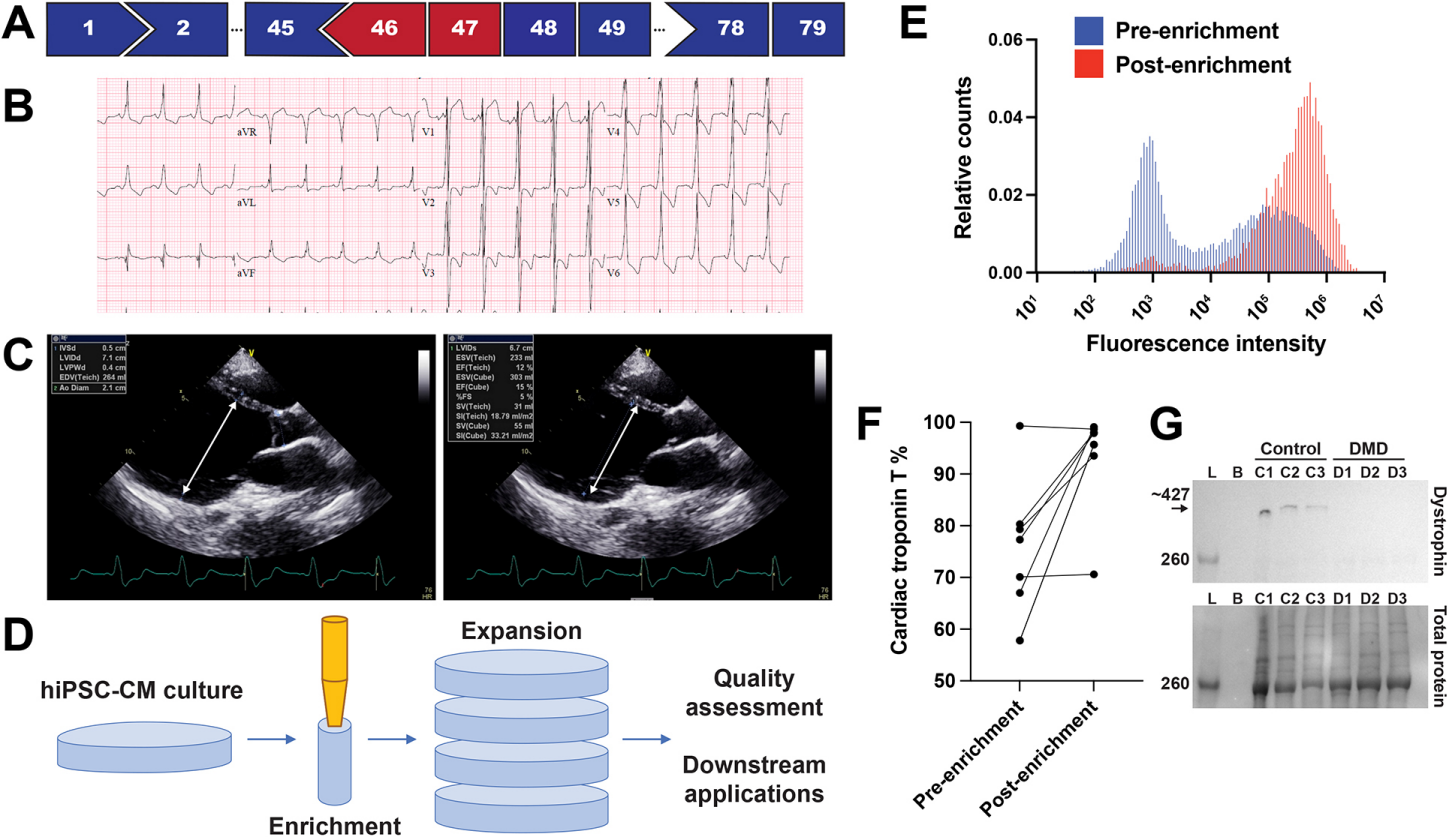
**Clinical characteristics of the patient with DMD and hiPSC-CM generation strategy.** (A) Abbreviated *DMD* exon map, showing an out-of-frame exon 46-47 deletion, highlighted in red. (B) Baseline electrocardiogram from the patient with DMD at age 19. (C) Still images from an echocardiogram from the patient with DMD at age 27, demonstrating an end diastolic dimension of 7.1 cm (arrow, left) and end systolic dimension of 6.7 cm (arrow, right). Ejection fraction was 12% by biplane measurement. (D) Overview of generation, enrichment and expansion strategy with quality assessment by cardiac troponin T flow cytometry. After differentiation, hiPSC-CMs were first enriched using the Miltenyi MACs system followed by expansion. (E) Representative cardiac troponin T staining as assessed by flow cytometry before and after enrichment, with an increase in cardiac troponin T positivity from 58.7% to 95.7%. (F) Validation of enrichment strategy, showing change in cardiac troponin T positivity pre- and post-enrichment for the DMD-G01 line (*n*=7 from seven differentiations). (G) Immunoblots of control and DMD hiPSC-CMs at the time of replating, demonstrating full-length dystrophin expression (arrow, molecular mass ∼427 kDa) in control hiPSC-CMs and not in DMD hiPSC-CMs from three separate differentiations for each line (upper blot). A loading control is shown in the lower panel corresponding to total protein (molecular mass ∼220 kDa for myosin heavy chain band). Lanes from left to right: ladder (L), blank (B), control cells (C1-C3) and DMD cells (D1-D3).

HiPSCs were generated from a patient with DMD patient with an out-of-frame, large deletion spanning *DMD* exons 46 and 47 (Fig. 1A). The patient had a typical DMD course with loss of ambulation before the age of 11 and developed an associated severe cardiomyopathy with a left ventricular ejection fraction of ∼12% despite guideline-directed therapy that included metoprolol, lisinopril and spironolactone, and biventricular chronic resynchronization therapy (Fig. 1B,C). He was never treated with glucocorticoid steroids. To reduce variability in hiPSC-CM differentiation, we applied a two-step hiPSC-CM enrichment and expansion protocol (Fig. 1D). HiPSCs were initially differentiated into ventricular-like hiPSC-CMs by conventional methods (Burridge et al., 2015; Gacita et al., 2021), followed by a second step in which hiPSC-CMs were enriched using a magnetic separation system. Assessment of pre- and post-enrichment by magnetic separation confirmed improved cardiac troponin T (TNNT2) positivity (Fig. 1E,F). This enriched hiPSC-CM cell population was then expanded using an established method (Buikema et al., 2020). Combining hiPSC-CM enrichment with expansion generated sufficient numbers of high-quality hiPSC-CMs for downstream applications. Dystrophin complex formation has been shown previously to occur by day 60 (Kamdar et al., 2020). We verified full-length dystrophin expression in control and not in DMD hiPSC-CMs at the time of replating by immunoblotting (Fig. 1G).

### Dystrophic hiPSC-CMs demonstrate an increased susceptibility to mechanical stress

**Fig. 2.**
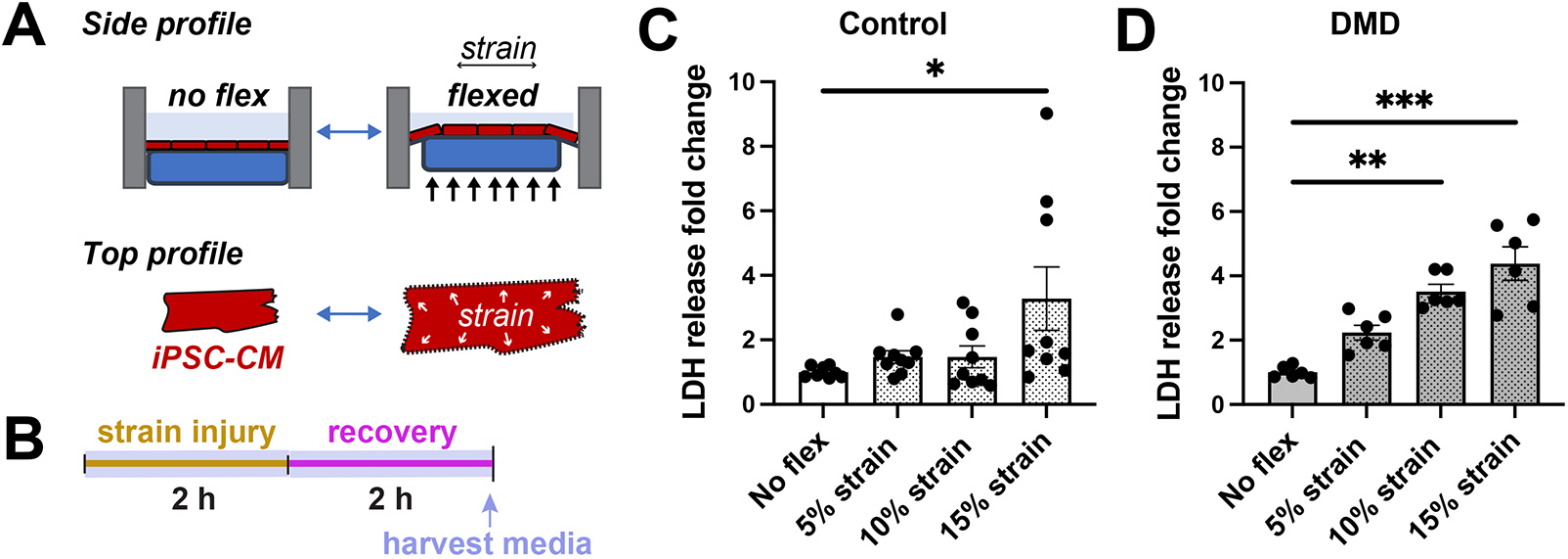
**DMD hiPSC-CMs show a differential response to equibiaxial strain.** (A) Schematic of application of mechanical stress using the FlexCell system that deforms hiPSC-CMs adhered to flexible silicone elastomer membranes using a rigid post, imparting equibiaxial strain. (B) Overview of the injury protocol timeline. hiPSC-CMs were subjected to mechanical stress for 2 h followed by a 2 h recovery period. The medium was then harvested to determine total LDH release. (C) Control hiPSC-CMs did not show a significant increase in the release of LDH compared to no-flex conditions at 5% and 10% strain. At 15% strain, LDH fold release increased by 2.3 (95% c.i., 0.1 to 4.5, *P=0.03, Kruskal–Wallis test with Dunn's multiple comparisons). *n*=8-9 from three differentiations. (D) DMD hiPSC-CMs showed an increase susceptibility to mechanical stress-induced injury compared to healthy control hiPSC-CMs. At 10% and 15% strain, LDH fold release increased relative to no-flex conditions by 2.51 (95% c.i., 1.9 to 3.0, ***P<0.002, Kruskal–Wallis test with Dunn's multiple comparisons) and 3.4 (95% c.i., 2.1 to 4.7, ***P<0.0004), respectively. *n*=6 from two differentiations. Data represent the mean±s.e.m.

Dystrophin-deficient cardiomyocytes from animal models have increased susceptibility to mechanical stress relative to that of controls (Danialou et al., 2001; Yasuda et al., 2005). Similarly, serum biomarkers reflective of membrane leakage are elevated in patients with DMD (Hathout et al., 2015; Spurney et al., 2021). Therefore, we initially sought to define a physiological degree of mechanical stress to impart on hiPSC-CMs that differentiated DMD hiPSC-CMs from healthy control hiPSC-CMs. HiPSC-CMs were plated onto flexible membranes in a six-well plate format and radial deformation was applied to impart a homogenous equibiaxial strain onto plated cells *in vitro* (Fig. 2A). Healthy control hiPSC-CMs and DMD hiPSC-CMs were subjected to 2 h of 0% (no flex), 5%, 10% or 15% strain and the cell culture medium was collected for biomarker determination (Fig. 2B). Lactate dehydrogenase (LDH) is a clinically relevant serum biomarker of tissue injury, including cardiac injury (Jaffe et al., 1996). LDH levels in control hiPSC-CM medium after 5% and 10% strain remained similar to those of unstressed (no-flex) conditions (Fig. 2C). At 15% strain, there was an increase in LDH release and an increase in the variability of the data, likely from the severity of the injury. The medium collected from DMD hiPSC-CMs showed a dose-dependent increase in LDH levels following strain injury (Fig. 2D), demonstrating that dystrophic hiPSC-CMs are more susceptible to strain-induced injury compared to control hiPSC-CMs. Similar to control hiPSC-CMs, the variability of LDH release for DMD hiPSC-CMs increased at 15% strain, likely related to the severe injury at this high level of strain. Based on our initial considerations to define a physiological degree of mechanical stress, we observed that 10% strain did not result in significant LDH release in control hiPSC-CMs, whereas it did result in a significant increase in LDH release in DMD hiPSC-CMs. Thus, subsequent experiments were performed at 10% strain.

### Application of stress generates a biomarker profile reflective of patients with DMD

**Fig. 3.**
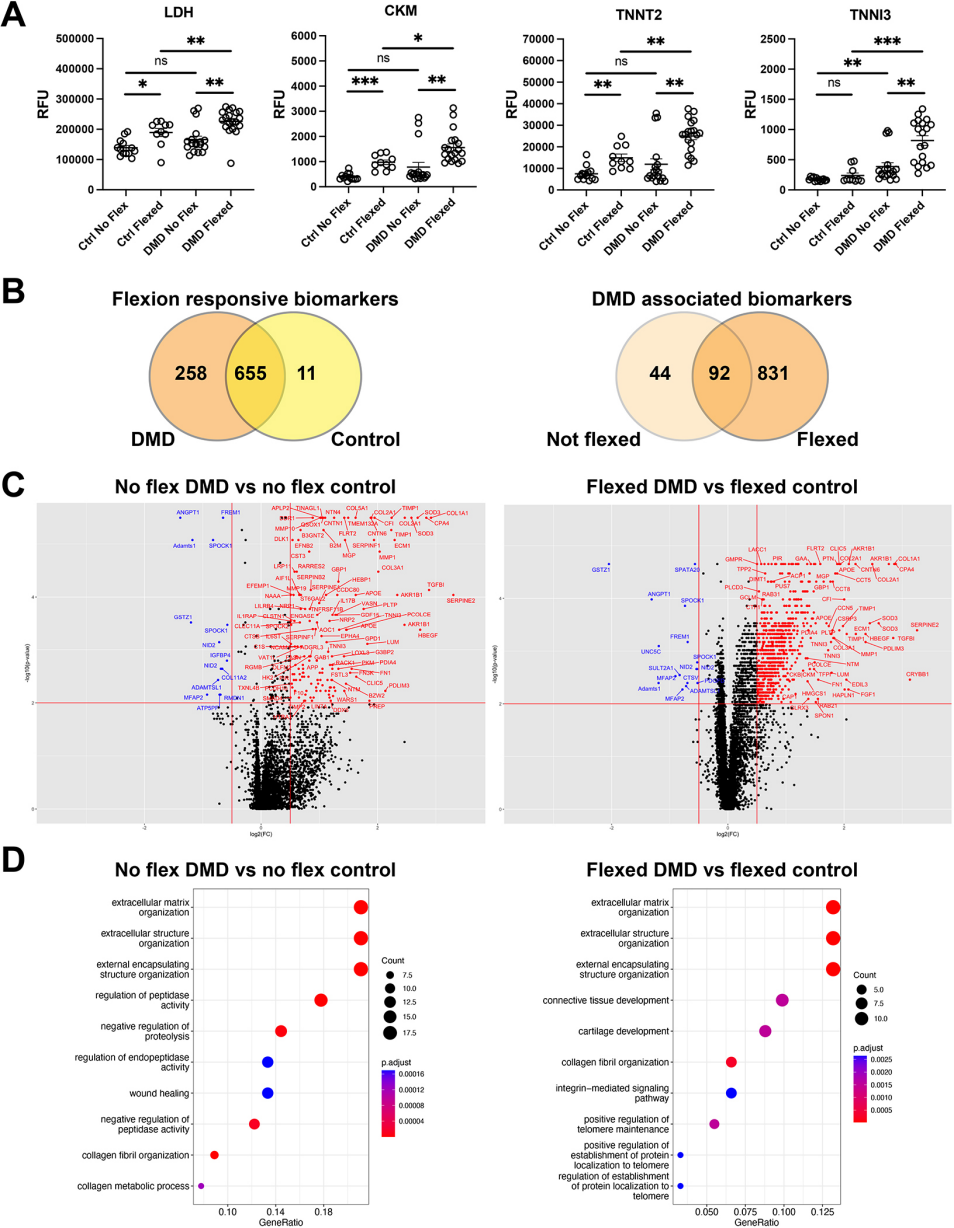
**Aptamer-based protein analysis indicates that DMD hiPSC-CMs show an increase in matrix-related proteins relative to those in control.** (A) Aptamer measurements of clinically relevant injury responsive biomarkers (relative fluorescence units or RFU). Data represent the mean±s.e.m. (B) Venn diagrams of biomarkers comparing the effect of flexion on the biomarker profile [FDR<0.01, absolute(log_2_FC)>0.5]. The Venn diagram on the left compares DMD flexed/DMD no-flex and control flexed/control no-flex conditions. The Venn diagram on the right compares DMD no-flex/control no-flex and DMD flexed/control flexed conditions. (C) Volcano plots comparing aptamer profiles from control and DMD hiPSC-CMs (left, no flex; right, flexed). A full listing of significant changes is provided in Tables S1 and S2. (D) Corresponding pathway enrichment analysis of the top 100 terms (C) as stratified by log_2_FC. Control no flex, *n*=13 from four differentiations; control flexed, *n*=10 from three differentiations; DMD no flex, *n*=18 from six differentiations; and DMD flexed, *n*=19 from six differentiations. ns, not significant; *P<0.05; **P <0.01; ***P<0.0001 (Wilcoxon rank-sum test).

A previous study conducted aptamer-based profiling on ambulatory and nonambulatory patients with DMD and otherwise healthy individuals (Hathout et al., 2015). These serum profiles measured 1125 markers, reflecting both skeletal and cardiac muscle disease in DMD. We employed this same technology to assess biomarker release into the medium from no-flex and flexed hiPSC-CMs after 2 h at 10% equibiaxial strain. As shown in Fig. 3A, the clinically relevant serum injury responsive biomarkers LDH, creatine kinase M-type (CKM), TNNT2 and TNNI3 were evaluated. After flexion, the aptamer assay detected variably increased CKM, LDH and TNNT2 levels in media from control and DMD hiPSC-CMs. Although the DMD samples were highly variable, these biomarkers did not significantly differ between control and DMD cells in the absence of flexing. For baseline (no-flex) comparisons, only TNNI3 levels were significantly different between DMD and control hiPSC-CMs. In contrast, flexing resulted in a significant increase in CKM, LDH, TNNT2 and TNNI3 levels in media from DMD hiPSC-CMs compared to those in media from healthy control hiPSC-CMs, mirroring what is seen clinically in serum from patients with DMD. Flexion of DMD and control hiPSC-CMs resulted in a common significant change of 655 biomarkers (Fig. 3B, left). An additional 258 compared to 11 biomarkers were changed in DMD compared to control hiPSC-CMs, respectively. In the baseline (no-flex) condition, 136 biomarkers were found to be significantly different between DMD and control cells (Fig. 3B, right), whereas flexing induced 831 additional significant biomarker changes and diminished 44 of the original 136 baseline changes. Collectively, these data support the notion that DMD hiPSC-CMs are more susceptible to mechanically induced injury compared to control hiPSC-CMs.

Fig. 3C shows a volcano plot comparing DMD and control hiPSC-CMs in the flexed and no-flex states. Reflective of decreased membrane stability in dystrophin-deficient cells, the vast majority of significantly released biomarkers were elevated in DMD compared to control hiPSC-CMs. Pathway enrichment analysis of the released biomarker profile of the top 100 terms by log fold change (FC) highlighted a baseline increase in extracellular matrix-related proteins in DMD hiPSC-CMs compared to control hiPSC-CMs, including matrix metalloproteinases (Fig. 3D). A similar profile was seen in the pathway enrichment analysis of flexed DMD compared to control hiPSC-CMs. Fig. S1 shows that TIMP1, TIMP2, MMP2 and MMP9 were elevated in media collected from baseline DMD hiPSC-CMs compared to their levels in healthy control hiPSC-CMs, indicating an elevation of these markers in the unflexed state. Flexing had minimal effect on these markers, indicating that the release of tissue and matrix metalloproteinases was not dependent on strain. Further comparison to a database of human matrisome proteins (Naba et al., 2012), as shown in Fig. S2, demonstrated that a subset of matrix proteins that were elevated at baseline in DMD hiPSC-CMs compared to their levels in control hiPSC-CMs, including the aforementioned MMPs and TIMPs, periostin (POSTN), annexin A1 (ANXA1), lumican (LUM) and plasminogen activator inhibitor-1 (PAI-1 or SERPINE1). Of note, upregulation of the transcripts for these proteins has previously been described in muscle biopsies from ‘presymptomatic’ patients with DMD under 2 years of age (Pescatori et al., 2007). Interestingly, flexion also caused the release of various metabolic pathway proteins in DMD relative to control hiPSC-CMs (Fig. S3). Thirty-two proteins previously implicated in muscle membrane repair were found on the aptamer panel (Bansal et al., 2003; Benink and Bement, 2005; Cai et al., 2009; Demonbreun et al., 2016; Griffin et al., 2016; Leung et al., 2013; Marg et al., 2012; Nakamura et al., 2023; Roostalu and Strahle, 2012; Scheffer et al., 2014). Analysis of these membrane repair proteins demonstrated a clear response to equibiaxial strain in DMD compared to control hiPSC-CMs (Fig. S4), supporting that flexion induces membrane injury and elicits downstream repair processes in DMD more than in control hiPSC-CMs.

**Fig. 4.**
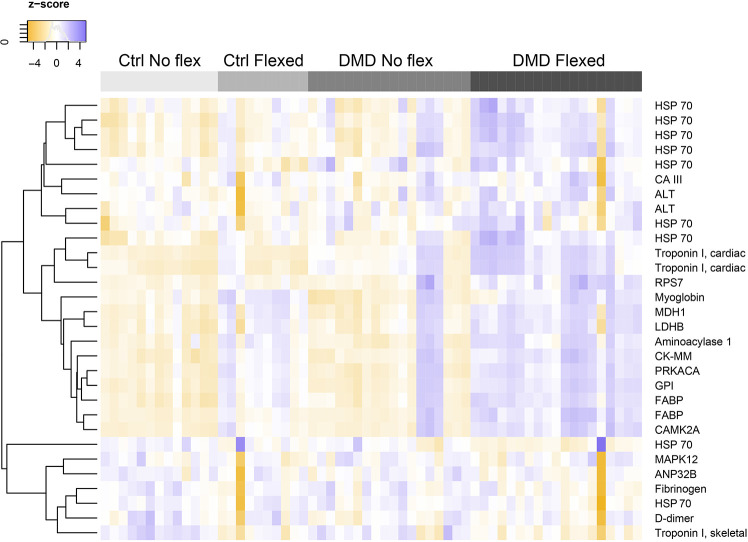
**Biomarkers from patients with DMD correlate with biomarkers released from flexed DMD hiPSC-CMs.** Hathout et al. (2015) conducted aptamer-based profiling on serum collected from patients with DMD from multiple stages of disease progression. Group 1 markers are those seen in early DMD that are different from those seen in individuals without DMD. Group 1 markers decrease over the DMD lifespan, consistent with loss of muscle mass and replacement of muscle by fibrosis. In comparison to the aptamer-based protein biomarkers seen in serum from patients with DMD, media isolated from DMD hiPSC-CMs subjected to equibiaxial strain showed similar elevated biomarkers, consistent with mechanical stress-induced protein release from cultured cells. Several proteins were detected by multiple aptamers and are included for completeness. HSP, heat shock protein; CA III, carbonic anhydrase 3 (CA3); ALT, alanine aminotransferase 1; FABP, fatty acid-binding protein, heart; RPS7, 40S ribosomal protein S7; MDH1, malate dehydrogenase, cytoplasmic; LDHB, L-lactate dehydrogenase B chain; PRKACA, cAMP-dependent protein kinase catalytic subunit α; CK-MM, creatine kinase M-type (CKM); GPI, glucose-6-phosphate isomerase; CAMK2A, calcium/calmodulin-dependent protein kinase II α; ANP32B, acidic leucine-rich nuclear phosphoprotein 32 family member B; MAPK12, mitogen-activated protein kinase 12. Control (Ctrl) no flex, *n*=13 from four differentiations; control flexed, *n*=10 from three differentiations; DMD no flex, *n*=18 from six differentiations; and DMD flexed, *n*=19 from six differentiations.

Hathout et al. (2015) previously used the aptamer method to define serum proteins in patients with DMD at different disease stages. They identified group 1 proteins as increased in early-disease ambulatory patients with DMD compared to age-matched otherwise healthy individuals (study age range from 2 to 28 years), and these group 1 markers decreased over time in patients with DMD, consistent with the progressive loss of muscle mass seen over time in these patients. We expected group 1 proteins to be most similar to those expressed in the conditions mimicked by hiPSC-CMs, where striated muscle cells were present but leaky due to mechanical injury in the setting of baseline fragile membranes. DMD skeletal and cardiac muscle is characterized by fibrofatty infiltrate that is absent in hiPSC-CM monolayer cultures. Fig. 4 shows a heatmap evaluating the DMD group 1 biomarkers and compares their release into the media in control and DMD hiPSC-CMs at baseline and flexed states. In control hiPSC-CMs, few of these group 1 markers were elevated, and there was little shift in response to mechanical stress. In contrast, group 1 biomarkers showed a striking response to mechanical stress in the DMD hiPSC-CMs, with nearly all markers having greater release into the medium after the application of equibiaxial stress. For example, alanine aminotransferase 1 (ALT or GPT), aspartate aminotransferase 1 (AST or GOT1), heat shock 70 kDa protein 1A (HSP70 or HSPA1A), MDH1, FABP (or FABP3), CAMK2A and myoglobin (MB) were detected at higher levels after flexing in DMD hiPSC-CMs, indicating that a component of the serum biomarker elevation seen in patients with DMD may derive from the heart. As shown in Fig. S5, this pattern was specific to group 1 biomarkers, as we did not see this pattern in the group 2, 3 or 4 proteins identified by Hathout et al. (2015). Of the group 1 biomarkers, ANP32B, MAPK12, troponin I (TNNI2, skeletal isoform) and fibrinogen/D-dimer did not demonstrate this pattern (Fig. 4, bottom grouping). MAPK12 is involved in myogenesis and may therefore be a skeletal muscle-specific response to stress (Brennan et al., 2021). Similarly, TNNI2 is produced almost exclusively in skeletal muscle (Cummins and Perry, 1978; Wade et al., 1990). Levels of the broadly expressed ANP32B, as well as liver-expressed fibrinogen and its breakdown product D-dimer, did not differ between control and DMD cells or in response to flexing and may reflect their low expression in hiPSC-CMs (Reilly et al., 2011; Weisel, 2005). Overall, these data demonstrate that the application of stress to hiPSC-CMs generates a biomarker profile more reflective of what is seen in ambulatory patients with DMD early on in the disease course and emphasizes the importance of physical culture conditions for DMD hiPSC-CMs in eliciting a clinically relevant phenotype.

### Recombinant annexin A6 limits LDH release after strain injury in control hiPSC-CMs

**Fig. 5.**
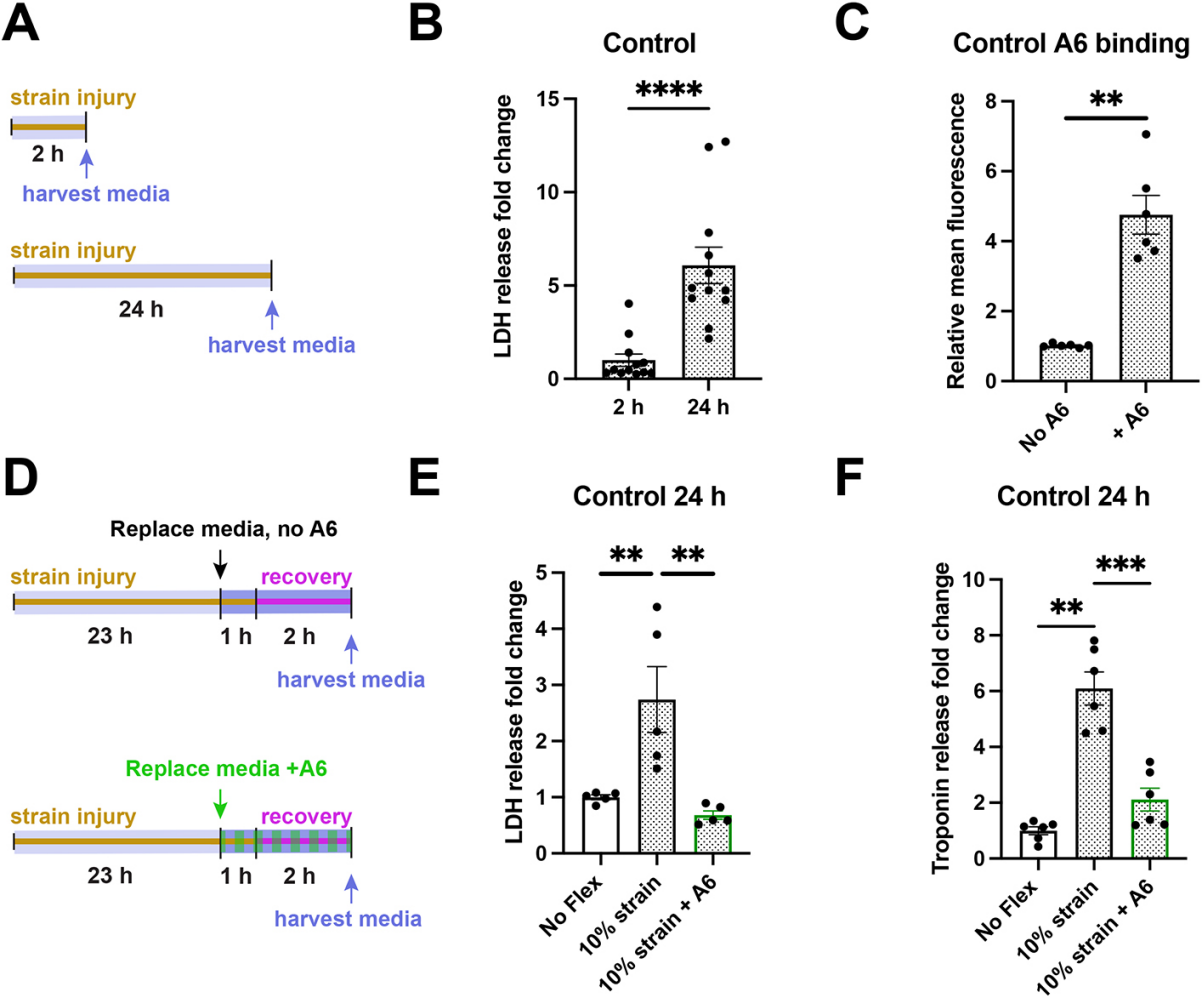
**Recombinant annexin A6 reduces injury biomarker release after strain in healthy control hiPSC-CMs.** (A) Schematic of the injury protocol comparing 2 h and 24 h at 10% strain as healthy control hiPSC-CMs require greater duration of mechanical stress to induce injury. (B) LDH release increased by 5.1(±1.0)-fold (****P<0.0001, Mann–Whitney test) at 24 h compared to 2 h of 10% strain in control hiPSC-CMs. *n*=12 from one differentiation. (C) Fold change of relative fluorescence intensity increased by 3.8±0.6 (**P=0.002, Mann–Whitney test) in control hiPSC-CMs treated with fluorescently labeled recombinant annexin A6, which was added for the last 1 h of a 10% strain protocol lasting 24 h. *n*=6 from two differentiations. (D) Protocol for assessing recombinant annexin A6 with a 24 h injury protocol. (E) LDH release increased by 1.7(±0.6)-fold (**P=0.008, Mann–Whitney test) relative to no-flex control hiPSC-CMs after a 24 h 10% strain protocol. Recombinant annexin A6 reduced LDH fold release by 2.1±0.1 (**P=0.001, Kruskal–Wallis test with Dunn's multiple comparisons) under a 10% strain protocol relative to untreated strained hiPSC-CMs, and no significant difference was observed between no-flex and treated 10% strained hiPSC-CMs (*P*=0.2, Mann–Whitney test). *n*=5 from two differentiations. (F) Troponin release increased by 5.1(±0.6)-fold (**P=0.002, Mann–Whitney test) after a 10% strain 24 h protocol. Treatment with recombinant annexin A6 under the same protocol reduced troponin release by 4.0(±0.4)-fold (***P=0.0005, Kruskal–Wallis test with Dunn's multiple comparisons), with no significant difference compared to the no-flex condition (*P*=0.06). *n*=6 from two differentiations. Data represent the mean±s.e.m.

Having defined a strain exposure that differentiated between DMD and healthy control hiPSC-CMs, we tested whether longer exposure to strain could induce injury in healthy control hiPSC-CMs (Fig. 5A). As shown in Fig. 5B, LDH release fold change increased by 5.1±1.0 (****P<0.0001) after 24 h of flexing compared to the non-injury-inducing 2 h time period. This finding is consistent with healthy control hiPSC-CMs having a higher threshold for LDH release compared to DMD hiPSC-CMs, and these findings are reflective of human myocardial injury, where LDH release can be detected after injury in non-DMD hearts. Annexin A6 is a known membrane repair protein that localizes at the site of skeletal muscle and cardiomyocyte injury where it promotes repair (Demonbreun et al., 2022). Recombinant annexin A6 was previously shown to promote resealing in mouse skeletal myofibers after laser injury (Demonbreun et al., 2019; Swaggart et al., 2014). Based on these findings, we assessed the efficacy of recombinant annexin A6 to reduce biomarker release in hiPSC-CMs using this mechanical injury model. We first assessed whether fluorescently labeled recombinant annexin A6 bound to control hiPSC-CMs after exposure to strain. As shown in Fig. 5C, the relative mean fluorescent intensity increased by 3.8±0.6 (**P=0.002) in treated compared to untreated control hiPSC-CMs as assessed by flow cytometry, consistent with recombinant annexin A6-hiPSC-CM binding. Fig. 5D depicts the experimental strategy for assessing response to recombinant annexin A6 in which membrane damage is followed by exposure to recombinant annexin A6 or vehicle, followed by 1 h of continued strain and subsequently a 2 h recovery period. In the absence of recombinant annexin A6, equibiaxial strain resulted in a fold change increase in LDH release of 1.7±0.6 (**P=0.008) compared to no-flex controls (Fig. 5E). When recombinant annexin A6 was present during the post-injury recovery period, LDH release was similar to LDH release from no-flex cells (*P*=0.2). To corroborate these findings, troponin T release was also measured (Fig. 5F) and was found to be similarly increased by 5.1±0.6 (**P=0.002) with the application of mechanical stress. Troponin T was reduced to near baseline levels with recombinant annexin A6 treatment (*P*=0.06). Together, these data demonstrate the utility of this system for assessing the effect of a membrane resealing agent on biomarker release from injured hiPSC-CMs.

### Annexin A6 limits biomarker release from dystrophic hiPSC-CMs

**Fig. 6.**
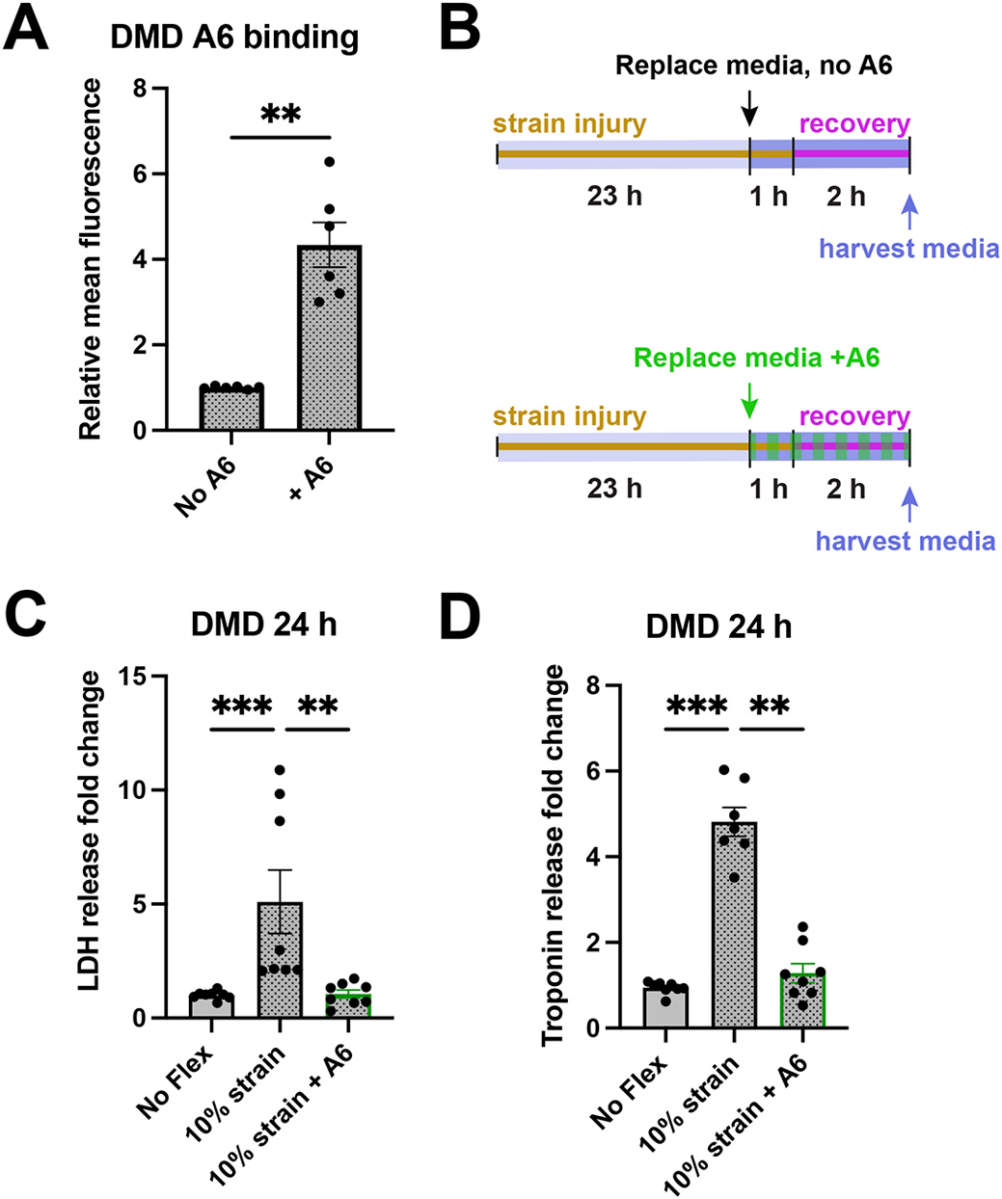
**Recombinant annexin A6 reduces injury biomarker release after strain in DMD hiPSC-CMs.** (A) Fold change of mean fluorescence intensity increased by 3.4±0.5 (**P=0.002, Mann–Whitney test) in DMD hiPSC-CMs treated with fluorescently labelled recombinant annexin A6, which was added for the last 1 h of a 10% strain protocol lasting 24 h. *n*=6 from two differentiations. (B) Overview of injury protocol for assessing recombinant annexin A6 with a 24 h injury protocol. (C) LDH release increased by 4.1(±1.4)-fold (***P=0.0002, Mann–Whitney test) relative to no-flex DMD hiPSC-CMs after a 24 h 10% strain protocol. Recombinant annexin A6 reduced LDH release by 4.0(±0.2)-fold (**P=0.002, Kruskal–Wallis test with Dunn's multiple comparisons) under a 10% strain protocol relative to that in untreated strained hiPSC-CMs, and no significant difference was observed between no-flex and treated 10% strained hiPSC-CMs (*P*=0.99). *n*=8 from three differentiations. (D) Troponin release increased by 3.9(±0.3)-fold (***P=0.0003, Mann–Whitney test) after a 10% strain 24 h protocol. Treatment with recombinant annexin A6 under the same protocol reduced troponin release by 3.5(±0.2)-fold (**P=0.007, Kruskal–Wallis test with Dunn's multiple comparisons) with no significant difference compared to the no-flex condition (*P*=0.9). *n*=7-8 from three differentiations. Data represent the mean±s.e.m.

Knowing that dystrophic cells are highly prone to membrane injury, we assessed whether recombinant annexin A6 could reduce biomarker release from severely injured DMD hiPSC-CMs. We first assessed fluorescently labeled recombinant annexin A6 binding to DMD hiPSC-CMs after a 24 h strain protocol. As shown in Fig. 6A, the relative mean fluorescence intensity increased by 3.4(±0.5)-fold (**P=0.002) in treated, strained DMD hiPSC-CMs, demonstrating recombinant annexin A6 binding. DMD hiPSC-CMs were subjected to the same 24 h, 10% strain injury protocol that is capable of injuring control hiPSC-CMs (Fig. 6B). As shown in Fig. 6C, LDH release increased by 4.1(±1.4)-fold (***P=0.0002) compared to that in no-flex DMD hiPSC-CMs. With the addition of recombinant annexin A6 during the post-injury recovery period, LDH levels were similar to those in no-flex hiPSC-CM media (*P*=0.99) and significantly lower than in media from flexed cells lacking recombinant annexin A6 [4.0(±0.2)-fold reduction in LDH release, **P=0.002]. Relative troponin release mirrored relative LDH levels, increasing 3.9(±0.3)-fold (***P=0.0003) post-injury in the absence of recombinant annexin A6 compared to no-flex controls (Fig. 6D). Treatment with recombinant annexin A6 reduced relative troponin levels by 3.5(±0.2)-fold (**P=0.007), with no significant difference compared to those of the no-flex condition (*P*=0.9). These results demonstrate that the application of recombinant annexin A6 reduced the release of injury biomarkers from dystrophic hiPSC-CMs.

## DISCUSSION

*In vivo*, cardiomyocytes are under constant cyclic stress due to repetitive cardiac contraction. Membrane damage and repair are part of normal physiology; however, certain diseases are associated with excessive membrane damage (Clarke et al., 1993; McNeil and Steinhardt, 1997). Previous work has demonstrated in both *in vitro* and *in vivo* settings that physiological stress of the rat myocardium with isoproterenol induces transient membrane damage, marked by biomarker release (Clarke et al., 1993). In the *mdx* mouse, which lacks full-length dystrophin at the sarcolemma, skeletal muscle is known to have a fragile membrane, readily prone to disruption. Similarly, cardiomyocytes from the *mdx* mouse have an increased susceptibility to membrane injury (Danialou et al., 2001). This membrane fragility is viewed as the primary deficit in dystrophin-deficient skeletal myofibers and cardiomyocytes where membrane damage is the initial cellular insult leading to a host of downstream consequences (Bloch and Gonzalez-Serratos, 2003; Ervasti, 2003; Houang et al., 2018; Townsend et al., 2011; Yasuda et al., 2005), reflected by elevated serum proteins of both skeletal and cardiac origin in patients with DMD (Hathout et al., 2015; Spurney et al., 2021).

HiPSCs offer the advantage of harboring human pathogenic variants in a native cell and genomic context that can be differentiated and tested for treatment response (Blinova et al., 2019; Tu et al., 2018). However, despite the ability to generate hiPSC-CMs, the conditions under which most cells are studied fail to simulate afterload and preload. In the case of DMD cardiomyopathy, this is critical to creating micro-injury in the plasma membrane. Engineered heart tissues can be used to improve the alignment of hiPSC-CMs, which may improve membrane maturation; however, present methods for imparting dynamic mechanical stress are limited (Breckwoldt et al., 2017). In a recent report, Sewanan et al. (2021) simulated pressure-volume loops in decellularized porcine myocardium engineered heart tissue seeded with hiPSC-CMs (Sewanan et al., 2021). By employing flexible membranes capable of deformation by equibiaxial strain to monolayer hiPSC-CMs, we successfully applied mechanical strain to hiPSC-CMs in a physiologically meaningful way to study DMD-associated cardiomyopathy with clinically relevant protein biomarker outputs. The biomarkers released after the application of strain included small proteins known to be released in DMD serum, and those markers are primarily reflective of myocardial injury. We also observed protein markers consistent with extracellular matrix remodeling; given the absence of other cell types within these hiPSC-CM cultures, these matrix markers reflect the cardiomyocyte contribution to matrix remodeling. These findings parallel single-cell RNA sequencing studies using DMD hiPSC-CMs, where the authors found increased activation of fibrosis-associated genes in DMD hiPSC-CMs compared to controls (Kamdar et al., 2020). Use of an aptamer-based method allowed us to study more than 1000 proteins and permitted a direct comparison of our results to protein profiling from human DMD serum. A striking correlation was seen when comparing serum from patients with DMD to hiPSC-CMs after 10% equibiaxial strain had been applied to the cells. HiPSC-CMs selected and differentiated under these conditions are primarily cardiomyocytes, and these cultures lack the typical infiltrative cells that characterize intact dystrophic heart or muscle tissue. These data are consistent with the notion that physiological mechanical stress is necessary to bring out the clinically relevant phenotype in these cell models. Isogenic DMD and control lines can be created by editing the *DMD* gene, provided the primary mutation is editable, helping to eliminate the effects of genetic background. Future studies with additional cell lines derived from patients with DMD, as well as with isogenic DMD and control lines using CRISPR-based gene editing, would add additional strength to the findings. However, many large deletions in the *DMD* gene are not readily corrected using gene-editing strategies, making it challenging to have representative isogenic cell lines derived from patients with DMD.

Several therapeutic approaches for the treatment of DMD have targeted increased membrane fragility. Poloxamer 188 is a triblock copolymer that has been extensively investigated for its membrane stabilization properties and has been shown to improve *mdx* hemodynamics and cardiomyocyte resistance to stretch-mediated injury (Houang et al., 2018; Yasuda et al., 2005). Enhancing native membrane repair is an alternative strategy. Mitsugumin 53 (MG53 or TRIM72) is a protein critical for muscle membrane repair that is also implicated in ischemic preconditioning (Cai et al., 2009; Cao et al., 2010). Recombinant MG53 has been shown to enhance membrane repair and ameliorate aspects of muscle pathology in the *mdx* mouse (Weisleder et al., 2012). Mineralocorticoid receptor antagonism with spironolactone and finerenone has been shown to improve membrane integrity in skeletal and cardiac muscle in murine models of DMD (Hauck et al., 2019; Lowe et al., 2020). *Anxa6*, the gene encoding annexin A6, was discovered as a genetic modifier of muscular dystrophy, including genetic signals that implicated annexin A6 in cardiac function in a mouse model of muscular dystrophy (Swaggart et al., 2014). Overexpression of annexin A6 enhances membrane repair in murine skeletal myofibers, and exogenously added recombinant annexin A6 similarly improves resealing of injured murine skeletal muscle myofibers and murine cardiomyocytes (Demonbreun et al., 2022, 2019, 2016). This work builds on these findings, demonstrating that recombinant annexin A6 reduced leakage of injury biomarkers in DMD hiPSC-CMs. Based on prior studies with recombinant annexin A6, we expect the reduction in injury biomarkers in the medium reflects enhanced membrane resealing and repair. The effectiveness of recombinant annexin A6 on biomarker release from healthy control hiPSC-CMs highlights its role in mediating general cellular repair (Demonbreun et al., 2022). This platform provides a potential technique for comparing the relative efficacy of membrane-stabilizing therapeutic strategies such as recombinant annexin A6, poloxamer 188, MG53 and mineralocorticoid antagonists, as well as understanding potential synergistic effects among various treatment strategies. Inotropic stress with agents such as isoproterenol has been shown in animal (Danialou et al., 2001; Meyers et al., 2019) and hiPSC-CM (Kamdar et al., 2020) models of DMD to also produce a clinically relevant phenotype. Both stressors increase membrane stress, and a direct comparison of equibiaxial strain and inotropic stimulation would provide an interesting future line of research.

Given that membrane disruption and injury are a part of normal physiology, endogenous repair mechanisms are considered sufficient, provided injury is not so extensive. However, when faced with physiological stress resulting in greater than normal membrane damage, as in the case of DMD, or in stressors that are greater than normal, as in a myocardial infarction or other myocardial injury, pathological damage ensues. Given these findings, recombinant annexin A6 may be useful in treating other forms of myocardial injury.

### Conclusions and limitations

In this work, equibiaxial strain was applied to hiPSC-CMs to assess the role of mechanical stress. Using this assay, we demonstrated a dose-dependent increase in protein biomarker release in DMD hiPSC-CMs and showed a response to a protein-resealing therapeutic, highlighting the value of monitoring these clinically useful biomarkers. We also identified that proteins released into the medium after equibiaxial strain overlapped with those seen in serum from ambulatory patients with DMD early on in the disease course , consistent with mechanical stress being a significant driver of DMD pathology. These data establish the importance of incorporating mechanical stress into cell-based assays of DMD cell injury; however, we expect that application of strain to other DMD genotypes and even other control lines will require adjustment of duration and degree of strain applied. Titration and calibration of experimental conditions also reflect the variability seen in human DMD and mouse models subjected to strain. Although we have taken steps to obtain highly pure hiPSC-CMs, *in vitro* hiPSC differentiation to hiPSC-CMs will inherently result in some non-cardiomyocyte lineage cells that may have contributed to some of our observations. In DMD, the primary genotype and secondary modifier genotypes influence disease onset and progression. Similarly, it can be expected that healthy control lines are also likely to have a range of tolerance to injury arising from both genetic and environmental conditions. Nonetheless, the range of parameters shown here provides guidance on conditions for those assays.

## MATERIALS AND METHODS

### HiPSC generation, hiPSC culture, cardiac differentiation, enrichment, expansion and characterization

Urine-derived epithelial cells were obtained from a patient with DMD and reprogrammed using published methods to generate the cell line DMD-G01 (Kim et al., 2019). The control line hiPSC line (GM033488, male donor) has been previously published (Gacita et al., 2021). HiPSC culture and differentiation were performed per previously published methods (Burridge et al., 2015; Gacita et al., 2021). At days 8-10 post initiation of differentiation with CHIR99021 (Tocris, 4423), hiPSC-CMs were harvested by collagenase digestion for 2 h (Breckwoldt et al., 2017) with the following modified digestion solution (1 ml per well of a six-well plate): 1 mg/ml collagenase II (Worthington, LS0041762), 10 mM HEPES, 2 µM thiazovivin (STEMCELL Technologies, 72254) and 30 µM N-benzyl-p-toluenesulfonamide (Tokyo Chemical Industry, B3082-5G) in Hanks’ balanced salt solution (Gibco, 14175095). Cells were isolated by centrifugation at 200 ***g*** for 5 min, followed by aspiration of the collagenase solution. HiPSC-CMs were separated from non-cardiomyocytes by magnetic labeling of non-hiPSC-CMs using a commercially available kit (Miltenyi Biotec, 130-110-188). Manufacturer instructions were followed, with the following modifications: (1) MACS buffer was defined as 0.5% KnockOut Serum Replacement (Gibco, 10828028) and 2 mM EDTA in calcium- and magnesium-free DPBS (Gibco, 14190144) and (2) only the first negative selection step was performed, omitting the second positive selection step. Enriched hiPSC-CMs were expanded in a protocol adapted from Buikema et al. (2020). hiPSC-CMs were replated at 2 million cells per 10 cm plate in B27 (Gibco, 17-504-044) in RPMI 1640 medium (Gibco, 11875101) containing 2 µM thiazovivin, and 10% KnockOut Serum Replacement. 10 cm plates were coated with 1:400 Matrigel (Corning, 354277) in Dulbecco's modified Eagle medium/F12 (Corning, MT10090CV) for at least 1 h prior to replating. After 24 h, the medium was exchanged with 2 µM CHIR99021 in RPMI 1640 medium supplemented with 2% B27 and exchanged every 48 h. After 7-10 days of expansion, cells were confluent and harvested for downstream applications by the collagenase digestion protocol as above. For dystrophin immunoblotting, cells were isolated by centrifugation and lysed in tissue lysis buffer [50 mM HEPES, pH 7.4, 150 mM NaCl, 2 mM EDTA, 10 mM NaF, 10 mM Na-pyrophosphate, 10% glycerol, 1% Triton X-100 with protease inhibitor (Roche, 11836170001) and PhosSTOP (Roche 04906837001)] for 15 min on ice. Protein amount was quantified by the Bradford assay (Bio-Rad, 5000205), 10 µg of protein was loaded and run on a 3-6% Tris-acetate gel (Invitrogen, EA03785BOX), and then transferred to a PVDF membrane. The membrane was then blocked with StartingBlock (Thermo Fisher Scientific, 37543), incubated with rabbit anti-dystrophin polyclonal antibody (1:1000, Invitrogen, PA1-37587), washed, incubated with the secondary antibody goat anti-rabbit IgG-HRP (1:2500, Jackson ImmunoResearch, 111-035-003), and then washed. Signals were detected on an iBright1500 Invitrogen system using SuperSignal West Femto Maximum Sensitivity Substrate (Thermo Fisher Scientific, 34577). Total protein was assessed with the Pierce Reversible Protein Stain Kit (Thermo Fisher Scientific, 34096).

### Preparation of flexible membranes and application of equibiaxial strain

Silanization of flexible membrane six-well plates (Bioflex culture plates, FlexCell International) was performed by adding 1 ml 5% (v/v) 3-aminopropyltriethoxysilane (Acros Organics, AC430941000) in 95% ethanol for 10 min. The solution was aspirated, and 1 ml 100% ethanol was added and immediately aspirated. Plates were incubated at 65°C for 20 min, washed once with 1 ml 95% ethanol, twice with 2 ml DPBS, and once with deionized water. Plates were then coated with 3 ml 1:400 Matrigel as per the hiPSC-CM expansion protocol. Expanded hiPSC-CMs were harvested by collagenase digestion as above and plated at a density of 1.5 million cells/well in RPMI 1640 medium supplemented with 2% B27, 10% fetal bovine serum (Gibco, 26140079) and 1% penicillin/streptomycin (Gibco, 15070063). The medium was exchanged with RPMI 1640 supplemented with 2% B27 and 1% penicillin/streptomycin every other day. On day 7 post replating, the medium was exchanged with fresh B27 in RPMI 1640, and cyclic sinusoidal equibiaxial strain at 1 Hz was applied using a FX-6000T Tension System (FlexCell International).

### HiPSC-CM troponin T staining and flow cytometry analysis

HiPSC-CMs were collected before or after enrichment in initial experiments and at the time of replating onto flexible membranes for all differentiations. All centrifugation steps in this protocol were performed at 600 ***g*** for 5 min. 500,000-1,000,000 cells were resuspended in 2 ml DPBS in fluorescence-activated cell sorting (FACS) tubes (Falcon, 352057), centrifuged and decanted. Cells were resuspended in 1 ml DPBS and 1 ml 8% paraformaldehyde (Electron Microscopy Sciences, 15710) in DPBS. Cells were incubated in a 37°C shaker for 10 min and then centrifuged and decanted. Cells were resuspended in 200 µl of ice-cold 90% methanol, 10% DPBS and stored at −20°C until staining for flow cytometry. Fresh incubation buffer containing 0.5% w/v bovine serum albumin (Sigma-Aldrich, A7906) in DPBS was prepared. Alexa Fluor 647 mouse anti-cardiac troponin T (1:200, BD Biosciences, 565744) and Alexa Fluor 647 mouse IgG1 κ isotype control (1:200, BD Biosciences, 557732) antibodies were prepared in the incubation buffer. Cells were split evenly into two FACS tubes and 2 ml incubation buffer was added to each tube before being centrifuged and decanted. Cells were incubated with 100 µl primary antibody or isotype control solution and incubated at room temperature in the dark for 1 h. 4 ml incubation buffer was added to each tube before being centrifuged and decanted. Cells were then resuspended in 100 µl DPBS and analyzed with a BD Accuri C6 Plus flow cytometer (BD Biosciences). If hiPSC-CM purity was <85%, cells were rejected for downstream applications.

### Biomarker measurement

LDH release and cardiac troponin T release were quantitated per manufacturer instructions using the Promega LDH-Glo Cytotoxicity Assay (Promega, J2380) and the human cardiac troponin T ELISA kit (Abcam, ab223860). Frozen culture media aliquots were sent to Somalogic (Boulder, CO, USA) for SomaScan analysis.

### Aptamer-based proteomics and analysis

The SomaScan assay reports 7322 aptamer-based proteomics results per sample in units of relative fluorescent units (RFU), which were read into R studio using the SomaDataIO R package (https://CRAN.R-project.org/package=SomaDataIO). For the study of individual protein levels, absolute RFUs are reported. The entire dataset is available in Table S3. A large proportion of measurements displayed non-normality (Shapiro–Wilk *P*-value<0.05); therefore, Wilcoxon rank-sum tests were used to assess differential serum biomarker levels across experimental groups. To account for multiple hypothesis testing, the Benjamini–Hochberg correction method was utilized using the p.adjust() function of the ‘stats’ R package (https://www.R-project.org/). Thresholds for significant differential biomarker levels were set at false discovery rate (FDR) <0.01 and an absolute value of log_2_FC>0.5. All statistical analysis was performed in R studio (R.4.0.2, 22 June 2020) with additional packages, and plots were generated using the ‘ggplot2’ and ‘gplots’ packages (https://ggplot2.tidyverse.org). Pathway enrichment was performed on differentially expressed biomarkers using the ‘clusterProfiler’ R package and the Gene Ontology database of terms (Wu et al., 2021). Analysis was performed on all differentially expressed terms, as well as a subset of the top 100 terms as stratified by log_2_FC.

### Recombinant annexin A6

HiPSC-CMs were treated with recombinant annexin A6 (produced and purified by Evotec, Princeton, NJ, USA) (Demonbreun et al., 2019) at a concentration of 10 µg/ml. In the case of binding studies, hiPSC-CMs were strained at 10% for 23 h, followed by addition of recombinant annexin A6 conjugated to Alexa Fluor 488 (produced and purified by Evotec), which were strained for one additional hour, incubated for an additional 2 h, washed twice with 2 ml Hanks’ balanced salt solution, collagenase-digested as described above, harvested after 2 h by quenching with an equal volume of medium, centrifuged for 10 min at 100 ***g***, resuspended in 100 µl DPBS and analyzed by flow cytometry as described above.

### Statistical methods

Data were analyzed using Prism 9.3.0. Where comparisons of two conditions were made, a Mann–Whitney test was used. Where comparisons of more than two conditions were made, the Kruskal–Wallis test with Dunn's multiple comparisons test was used. In all cases, *P*<0.05 was defined as statistically significant. Statistical data are reported as mean±s.e.m. Confidence intervals are reported as 95% (95% c.i.).

### Study approval

Written and informed consent was obtained from all human subjects included in this study, including for creation of hiPSC lines and deidentified medical image publication. All work conducted was approved by the Northwestern University Institutional Review Board.

## Supplementary Material

10.1242/dmm.050487_sup1 Supplementary information

Table S3. Full aptamer (Somalogics) dataset.
