# Supplementary material for: Physiological stress improves stem cell modeling of dystrophic cardiomyopathy
Source: Dis Model Mech. 2024 Feb 5;17(6):dmm050487. doi: 10.1242/dmm.050487 (PMC10820750; doi:10.1242/dmm.050487)
Supplement: Supplementary information [file dmm-17-050487-s1.pdf]

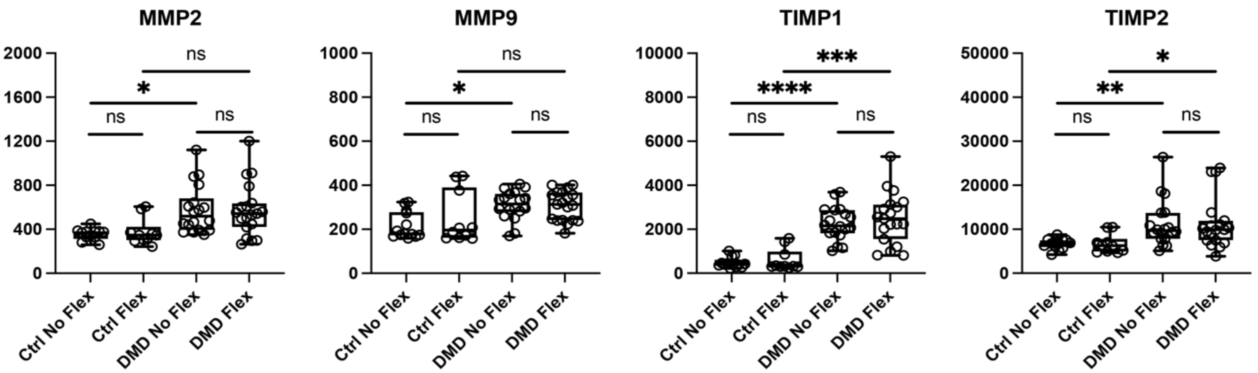

Fig. S1. **Aptamer measurements of selected matrix remodeling proteins.** Values given relative fluorescence intensity units (RFUs). Control no flex n = 13 from 4 differentiations, control flexed n = 10 from 3 differentiations, DMD no flex n = 18 from 6 differentiations, and DMD flexed n = 19 from 6 differentiations.

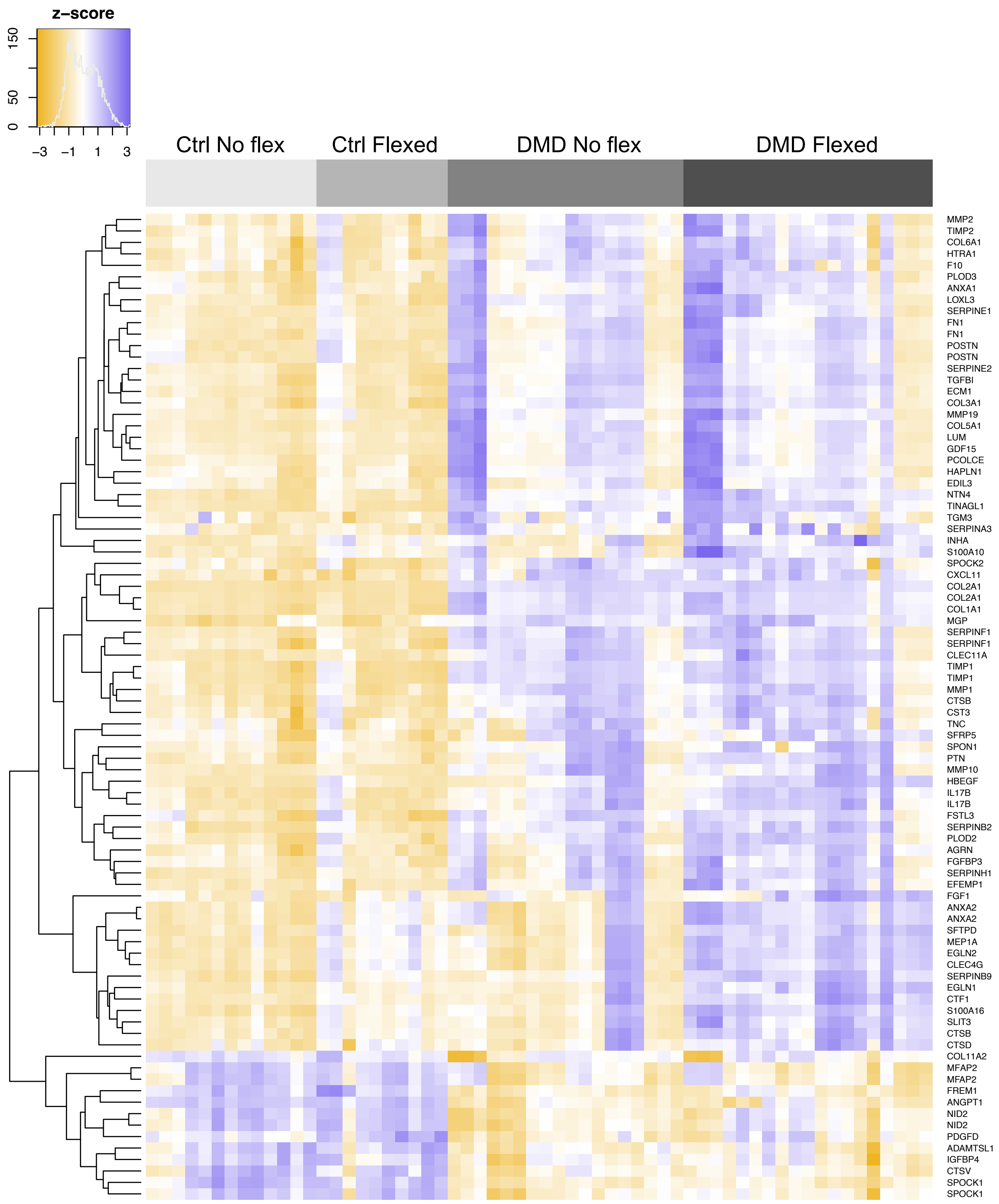

Fig. S2. Matrisome proteins that showed significant differences between DMD and control hiPSC-CMs with and without strain. Matrisome protein list from Naba et al cited in the main text. Control no flex n = 13 from 4 differentiations, control flexed n = 10 from 3 differentiations, DMD no flex n = 18 from 6 differentiations, and DMD flexed n = 19 from 6 differentiations.

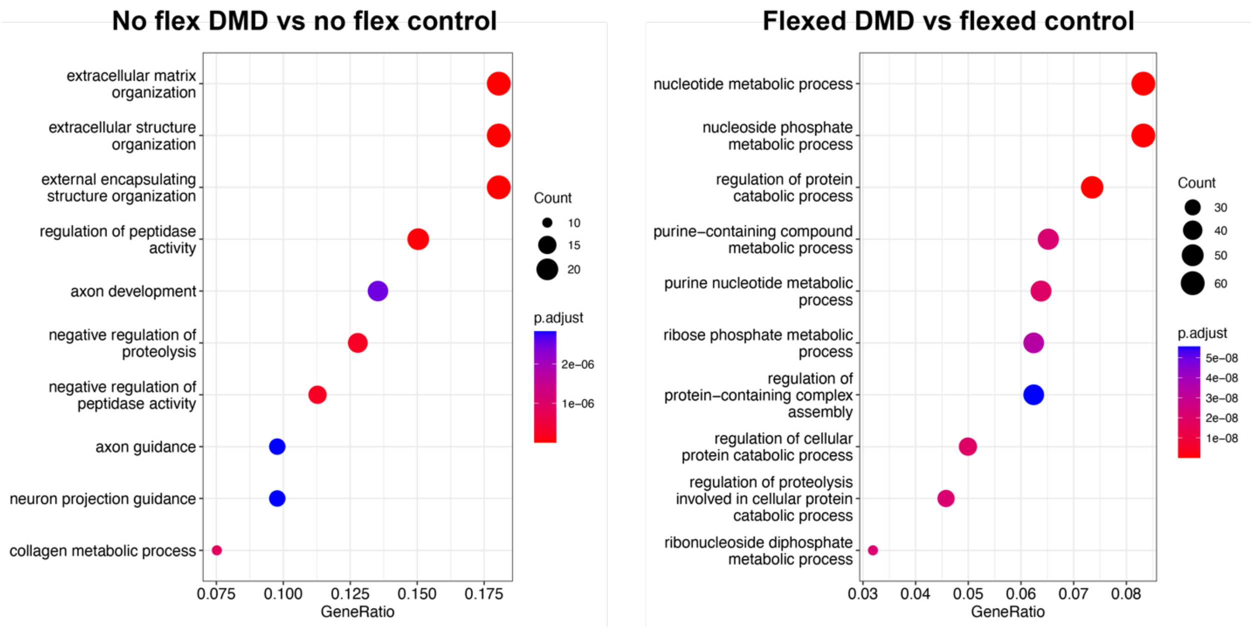

Fig. S3. Pathway enrichment analysis of Fig. 3C in main text of all significant changes. Control no flex n = 13 from 4 differentiations, control flexed n = 10 from 3 differentiations, DMD no flex n = 18 from 6 differentiations, and DMD flexed n = 19 from 6 differentiations.

Disease Models & Mechanisms • Supplementary information

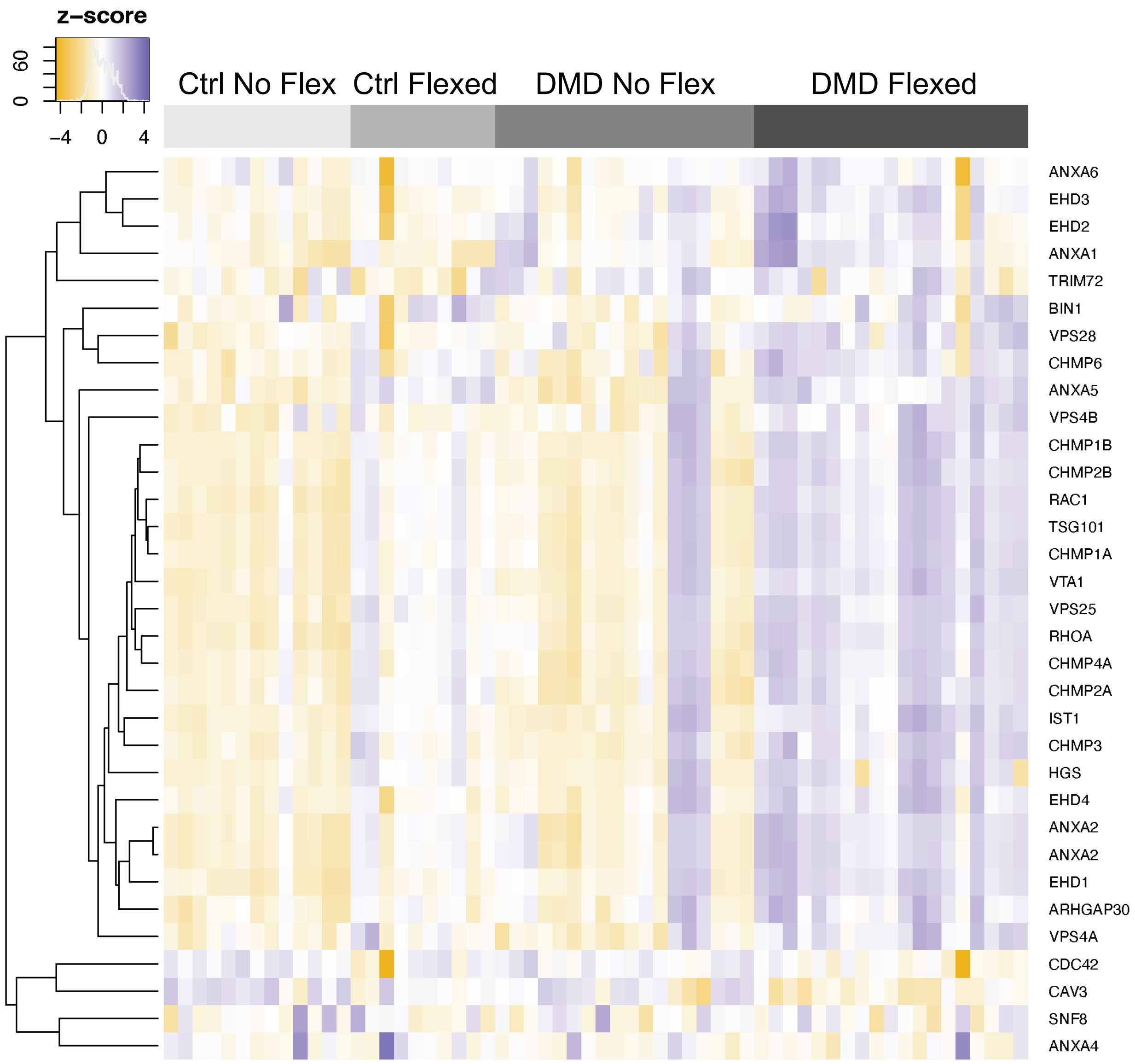

Fig. S4. Equibiaxial strain induces release of membrane repair proteins in DMD, but no control hiPSC-CMs. Control no flex n = 13 from 4 differentiations, control flexed n = 10 from 3 differentiations, DMD no flex n = 18 from 6 differentiations, and DMD flexed n = 19 from 6 differentiations.

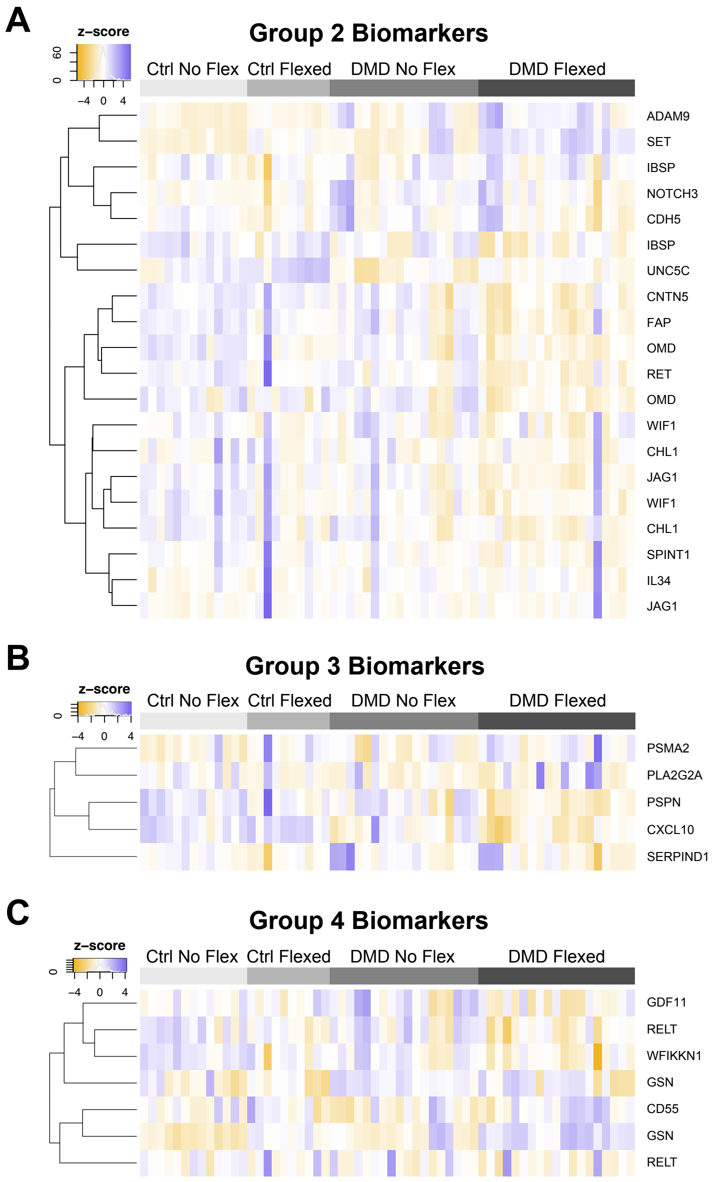

Fig. S5. Group 2, 3, and 4, biomarkers from patients with DMD (Hathout et al, referenced in main text) showed minimal baseline differences and did not respond to mechanical stress in DMD compared control hiPSC-CMs. (A) Group 2 proteins were defined by Hathout et al. as being lower in patients with DMD compared to otherwise healthy patients and not changing over time in patients with DMD. **(B)** Group 3 proteins were defined as those that did not change with age and were higher in patient with DMD compared to otherwise healthy patients. **(C)** Group 4 proteins were those that that were similar between otherwise healthy patients and patients with DMD at young age (ambulatory) but then increased in patients with DMD with age. Control no flex n = 13 from 4 differentiations, control flexed n = 10 from 3 differentiations, DMD no flex n = 18 from 6 differentiations, and DMD flexed n = 19 from 6 differentiations.

Disease Models & Mechanisms • Supplementary information

Table S1. Aptamer based profiling of hiPSC-CMs no flex DMD to no flex control.

Compiled list of no flex control to no flex DMD from Fig. 3C of the main text.

Aptamer Name	SeqId	Target	EntrezGeneSymbol	UniProt	p.val.adj	Log2(FC)
seq.10627.87	10627-87	Amyloid-like protein 2	APLP2	Q06481	3.20E-06	1.024666
seq.11140.56	11140-56	Collagen alpha-1(I) chain:C-term propeptide	COL1A1	P02452	3.20E-06	2.91646
seq.11192.168	11192-168	Tubulointerstitial nephritis antigen-like	TINAGL1	Q9GZM7	3.20E-06	1.048882
seq.11836.144	11836-144	Epithelial discoidin domain-containing receptor 1	DDR1	Q08345	3.20E-06	0.886405
seq.13122.19	13122-19	Leucine-rich repeat transmembrane protein FLRT2	FLRT2	O43155	3.20E-06	1.427612
seq.15569.15	15569-15	Collagen Type II	COL2A1	P02458	3.20E-06	1.893753
seq.18875.125	18875-125	Chondrocalcin	COL2A1	P02458	3.20E-06	2.466426
seq.20561.15	20561-15	Contactin-6	CNTN6	Q9UQ52	3.20E-06	1.881477
seq.20568.3	20568-3	FRAS1-related extracellular matrix protein 1	FREM1	Q5H8C1	3.20E-06	-0.65237
seq.22047.46	22047-46	Collagen alpha-1(V) chain	COL5A1	P20908	3.20E-06	1.622433
seq.2211.9	2211-9	Metalloproteinase inhibitor 1	TIMP1	P01033	3.20E-06	0.358115
seq.23173.3	23173-3	Metalloproteinase inhibitor 1	TIMP1	P01033	3.20E-06	2.23968
seq.2567.5	2567-5	Complement factor I	CFI	P05156	3.20E-06	1.946859
seq.2811.27	2811-27	Angiopoietin-1	ANGPT1	Q15389	3.20E-06	-1.38672
seq.2974.61	2974-61	Contactin-1	CNTN1	Q12860	3.20E-06	1.25414
seq.3327.27	3327-27	Netrin-4	NTN4	Q9HB63	3.20E-06	1.099552
seq.5660.51	5660-51	Extracellular superoxide dismutase [Cu-Zn]	SOD3	P08294	3.20E-06	2.687515
seq.6217.23	6217-23	Sulfhydryl oxidase 1	QSOX1	O00391	3.20E-06	1.065512
seq.6556.5	6556-5	Ectonucleotide pyrophosphatase/phosphodiesterase family member 5	ENPP5	Q9UJA9	3.20E-06	0.421315
seq.7871.16	7871-16	Transmembrane protein 132A	TMEM132A	Q24JP5	3.20E-06	1.495151
seq.8463.2	8463-2	Extracellular superoxide dismutase [Cu-Zn]	SOD3	P08294	3.20E-06	2.58064
seq.8479.4	8479-4	Stromelysin-2	MMP10	P09238	3.20E-06	1.016896
seq.9267.2	9267-2	Carboxypeptidase A4	CPA4	Q9UI42	3.20E-06	2.836446
seq.11117.2	11117-2	Spermatogenesis-associated protein 20	SPATA20	Q8TB22	5.46E-06	-0.32902
seq.25967.34	25967-34	Metalloproteinase inhibitor 1	TIMP1	P01033	5.46E-06	2.234414
seq.3485.28	3485-28	Beta-2-microglobulin	B2M	P61769	5.46E-06	1.071059
seq.7980.72	7980-72	N-acetyllactosaminide beta-1,3-N-acetylglucosaminyltransferase 2	B3GNT2	Q9NY97	5.46E-06	0.677922
seq.10379.19	10379-19	A disintegrin and metalloproteinase with thrombospondin motifs 1_MOUSE	Adamts1	P97857	8.42E-06	-1.17771
seq.11487.4	11487-4	Testican-1	SPOCK1	Q08629	8.42E-06	-0.82426
seq.14131.37	14131-37	Ephrin-B2:Extracellular domain	EFNB2	P52799	8.42E-06	0.64072
seq.3366.51	3366-51	Extracellular matrix protein 1	ECM1	Q16610	8.42E-06	2.292477
seq.4543.65	4543-65	Collagen alpha-1(XXIII) chain	COL23A1	Q86Y22	8.42E-06	-0.26492
seq.6373.54	6373-54	Protein delta homolog 1	DLK1	P80370	8.42E-06	0.539617
seq.6520.87	6520-87	Matrix Gla protein	MGP	P08493	8.42E-06	1.44113
seq.9211.19	9211-19	Pigment epithelium-derived factor	SERPINF1	P36955	8.42E-06	1.938319
seq.2609.59	2609-59	Cystatin-C	CST3	P01034	1.39E-05	0.826509
seq.4924.32	4924-32	Interstitial collagenase	MMP1	P03956	1.39E-05	2.035589
seq.15560.52	15560-52	Transcobalamin-2	TCN2	P20062	2.27E-05	-0.27502
seq.6504.65	6504-65	Lysyl oxidase homolog 2	LOXL2	Q9Y4K0	2.27E-05	0.327463
seq.15472.16	15472-16	Low-density lipoprotein receptor-related protein 11	LRP11	Q86VZ4	3.33E-05	0.574746
seq.18880.81	18880-81	Collagen Type III	COL3A1	P02461	3.33E-05	2.011685
seq.3079.62	3079-62	Retinoic acid receptor responder protein 2	RARRES2	Q99969	3.33E-05	0.612446
seq.15326.64	15326-64	Guanylate-binding protein 1	GBP1	P32455	5.14E-05	1.327358
seq.21676.17	21676-17	PAI-2	SERPINB2	P05120	7.37E-05	0.85358
seq.3283.21	3283-21	Transforming growth factor-beta-induced protein ig-h3	TGFBI	Q15582	7.37E-05	2.882615
seq.18225.13	18225-13	Heme-binding protein 1	HEBP1	Q9NRV9	9.14E-05	1.114467
seq.18871.24	18871-24	Allograft inflammatory factor 1-like	AIF1L	Q9BQI0	9.14E-05	0.654438
seq.19154.41	19154-41	Glia-derived nexin	SERPINE2	P07093	9.14E-05	3.299835
seq.3173.49	3173-49	N-acylethanolamine-hydrolyzing acid amidase	NAAA	Q02083	9.14E-05	0.508184
seq.3234.23	3234-23	Coiled-coil domain-containing protein 80	CCDC80	Q76M96	9.14E-05	1.308009
seq.5312.49	5312-49	Apolipoprotein E (isoform E2)	APOE	P02649	9.14E-05	1.624122
seq.6425.87	6425-87	Matrix metalloproteinase-19	MMP19	Q99542	9.14E-05	0.683939
seq.8480.29	8480-29	EGF-containing fibulin-like extracellular matrix protein 1	EFEMP1	Q12805	9.14E-05	0.557139
seq.9854.36	9854-36	Aldose reductase	AKR1B1	P15121	9.14E-05	2.337429
seq.2925.9	2925-9	Plasminogen activator inhibitor 1	SERPINE1	P05121	0.00013	0.997997
seq.12338.27	12338-27	Pikachurin	EGFLAM	Q63HQ2	0.000168	0.156074
seq.15475.4	15475-4	Phospholipid transfer protein	PLTP	P55058	0.000168	1.759828
seq.21737.20	21737-20	Beta-galactoside alpha-2,6-sialyltransferase 2	ST6GAL2	Q96JF0	0.000168	0.836708
seq.2654.19	2654-19	Tumor necrosis factor receptor superfamily member 1A	TNFRSF1A	P19438	0.000168	0.302626
seq.5542.22	5542-22	Neuropilin-1	NRP1	O14786	0.000168	0.755335
seq.6453.70	6453-70	Leukocyte immunoglobulin-like receptor subfamily B member 4	LILRB4	Q8NHJ6	0.000168	0.6816
seq.14022.17	14022-17	Interleukin-17B	IL17B	Q9UHF5	0.000218	1.226177
seq.4374.45	4374-45	Growth/differentiation factor 15	GDF15	Q99988	0.000218	1.303095
seq.5682.13	5682-13	Vasorin	VASN	Q6EMK4	0.000218	1.275382
seq.7628.40	7628-40	Cysteine-rich with EGF-like domain protein 1	CRELD1	Q96HD1	0.000218	0.409754
seq.8304.50	8304-50	Tumor necrosis factor receptor superfamily member 11B	TNFRSF11B	O00300	0.000218	0.964039
seq.14048.7	14048-7	Interleukin-1 Receptor accessory protein	IL1RAP	Q9NPH3	0.000286	0.508759
seq.15387.44	15387-44	Neuropilin-2	NRP2	O60462	0.000286	1.175352
seq.15521.4	15521-4	Calsyntenin-1	CLSTN1	O94985	0.000286	0.572814
seq.16781.2	16781-2	Cytosolic endo-beta-N-acetylglucosaminidase	ENGASE	Q8NFI3	0.000286	0.834449
seq.15486.126	15486-126	Amiloride-sensitive amine oxidase [copper-containing]	AOC1	P19801	0.000306	0.959984
seq.17331.138	17331-138	Kremen protein 1	KREMEN1	Q96MU8	0.000306	0.494934
seq.2966.65	2966-65	Stem cell growth factor-beta	CLEC11A	Q9Y240	0.000306	0.463982
seq.16872.248	16872-248	Maleylacetoacetate isomerase	GSTZ1	O43708	0.000306	-1.20293
seq.17783.9	17783-9	Cob(I)yrinic acid a,c-diamide adenosyltransferase, mitochondrial	MMAB	Q96EY8	0.000306	-0.26985
seq.4500.50	4500-50	Stem cell growth factor-alpha	CLEC11A	Q9Y240	0.000306	0.66535
seq.6496.60	6496-60	Protein delta homolog 1:Extracellular domain	DLK1	P80370	0.000306	0.319059
seq.16606.85	16606-85	Aldose reductase	AKR1B1	P15121	0.000339	2.461467
seq.5490.53	5490-53	Testican-1	SPOCK1	Q08629	0.000339	-0.57907

seq.5491.12	5491-12	Testican-2	SPOCK2	Q92563	0.000339	0.516765
seq.7161.25	7161-25	GDH/6PGL endoplasmic bifunctional protein	H6PD	O95479	0.000339	-0.30458
seq.9793.145	9793-145	Immunoglobulin superfamily DCC subclass member 4	IGDCC4	Q8TDY8	0.000348	0.471068
seq.21495.134	21495-134	Prosaposin receptor GPR37	GPR37	O15354	0.000407	0.254474
seq.7735.17	7735-17	Pigment epithelium-derived factor	SERPINF1	P36955	0.000407	0.936846
seq.8007.19	8007-19	Cathepsin B	CTSB	P07858	0.000407	0.888416
seq.11237.49	11237-49	Procollagen C-endopeptidase enhancer 1	PCOLCE	Q15113	0.00042	1.460701
seq.14094.29	14094-29	Heparin-binding EGF-like growth factor	HBEGF	Q99075	0.00042	2.730632
seq.2937.10	2937-10	Apolipoprotein E (isoform E3)	APOE	P02649	0.00042	1.468533
seq.5441.67	5441-67	Troponin I, cardiac muscle	TNNI3	P19429	0.00042	1.340642
seq.18284.77	18284-77	Protein KIBRA	WWC1	Q8IX03	0.000551	-0.07889
seq.16288.17	16288-17	Ephrin type-A receptor 4	EPHA4	P54764	0.000551	1.036969
seq.13697.51	13697-51	Glycerol-3-phosphate dehydrogenase [NAD(+)], cytoplasmic	GPD1	P21695	0.000719	1.125079
seq.16060.99	16060-99	Nidogen-2	NID2	Q14112	0.000719	-0.7186
seq.13114.50	13114-50	Lumican	LUM	P51884	0.000775	1.823841
seq.2620.4	2620-4	Interleukin-6 receptor subunit beta	IL6ST	P40189	0.000775	0.512562
seq.13639.101	13639-101	General vesicular transport factor p115	USO1	O60763	0.000868	0.162501
seq.13983.27	13983-27	Quinone oxidoreductase	CRYZ	Q08257	0.000868	-0.262
seq.17691.1	17691-1	Tripeptidyl-peptidase 1	TPP1	O14773	0.000868	0.498242
seq.20203.45	20203-45	Complement C1s subcomponent	C1S	P09871	0.000868	0.580284
seq.24236.46	24236-46	Synaptic vesicle membrane protein VAT-1 homolog-like	VAT1L	Q9HCJ6	0.000868	0.687051
seq.4254.6	4254-6	NudC domain-containing protein 3	NUDCD3	Q8IVD9	0.000868	-0.09737
seq.4498.62	4498-62	Neural cell adhesion molecule 1, 120 kDa isoform	NCAM1	P13591	0.000868	0.625224
seq.3038.9	3038-9	C-X-C motif chemokine 11	CXCL11	O14625	0.00094	0.471627
seq.17140.57	17140-57	Platelet-derived growth factor D	PDGFD	Q9GZP0	0.001092	-0.38692
seq.5930.54	5930-54	Troponin I, cardiac muscle	TNNI3	P19429	0.001092	1.151207
seq.7748.11	7748-11	NADH dehydrogenase [ubiquinone] flavoprotein 2, mitochondrial	NDUFV2	P19404	0.001092	-0.34106
seq.3814.63	3814-63	Interleukin-11 receptor subunit alpha	IL11RA	Q14626	0.001191	-0.2769
seq.12893.159	12893-159	Gamma-interferon-inducible protein 16:Isoform 2, Hematopoietic expression, interferon-inducible nature, and nuclear localization 2	IFI16	Q16666	0.001327	0.216965
seq.15427.35	15427-35	Lysyl oxidase homolog 3	LOXL3	P58215	0.001327	1.21681
seq.20578.10	20578-10	Latrophilin-3	ADGRL3	Q9HAR2	0.001327	0.849653
seq.23178.95	23178-95	Histamine N-methyltransferase	HNMT	P50135	0.001327	0.492282
seq.8295.16	8295-16	Noelin-2	OLFM2	O95897	0.001327	0.827374
seq.8403.18	8403-18	Fatty acid synthase	FASN	P49327	0.001327	0.60099
seq.3331.8	3331-8	RGM domain family member B	RGMB	Q6NW40	0.001327	0.742044
seq.9831.12	9831-12	Ras GTPase-activating protein-binding protein 2	G3BP2	Q9UN86	0.001327	1.423
seq.11104.13	11104-13	Chitinase-3-like protein 1	CHI3L1	P36222	0.001605	0.496486
seq.20161.41	20161-41	Neural cell adhesion molecule 1	NCAM1	P13591	0.001605	0.828691
seq.24268.21	24268-21	GRB2-associated-binding protein 1	GAB1	Q13480	0.001605	0.956526
seq.2950.57	2950-57	Insulin-like growth factor-binding protein 4	IGFBP4	P22692	0.001605	-0.5854
seq.3214.3	3214-3	Neuropilin-1	NRP1	O14786	0.001605	0.291581
seq.8840.61	8840-61	Complement C1s subcomponent	C1S	P09871	0.001605	0.469465
seq.8092.29	8092-29	Interleukin-6 receptor subunit alpha	IL6R	P08887	0.001618	0.231892
seq.10970.3	10970-3	Ecto-ADP-ribosyltransferase 3	ART3	Q13508	0.001618	0.422808
seq.13123.3	13123-3	Leucine-rich repeat transmembrane protein FLRT3:Extracellular domain	FLRT3	Q9NZU0	0.001747	0.340903
seq.12676.1	12676-1	Protein kinase C and casein kinase substrate in neurons protein 1	PACSIN1	Q9BY11	0.001929	0.192087
seq.13130.150	13130-150	Hexokinase-2	HK2	P52789	0.001929	0.590475
seq.21857.26	21857-26	Guanine nucleotide-binding protein subunit beta-2-like 1	RACK1	P63244	0.001929	1.182469
seq.3171.57	3171-57	Amyloid beta A4 protein	APP	P05067	0.001929	0.717398
seq.4240.31	4240-31	Pyruvate kinase PKM	PKM	P14618	0.001929	1.223428
seq.2212.69	2212-69	Tissue-type plasminogen activator	PLAT	P00750	0.002178	0.434572
seq.3499.77	3499-77	Interleukin-17B	IL17B	Q9UHF5	0.002282	0.645287
seq.11278.4	11278-4	Collagen alpha-2(XI) chain	COL11A2	P13942	0.002282	-0.66622
seq.15418.25	15418-25	Mitogen-activated protein kinase 10	MAPK10	P53779	0.002282	1.053475
seq.20525.200	20525-200	Protein disulfide-isomerase A4	PDIA4	P13667	0.002282	1.327416
seq.23662.10	23662-10	SH3 domain-binding protein 5	SH3BP5	O60239	0.002282	0.187993
seq.3438.10	3438-10	Follistatin-related protein 3	FSTL3	O95633	0.002282	1.222838
seq.3633.70	3633-70	Nidogen-2	NID2	Q14112	0.002282	-0.68964
seq.4131.72	4131-72	Fibronectin	FN1	P02751	0.002282	1.554488
seq.4160.49	4160-49	72 kDa type IV collagenase	MMP2	P08253	0.002282	0.721164
seq.10818.36	10818-36	Sphingomyelin phosphodiesterase	SMPD1	P17405	0.002388	0.45971
seq.21976.4	21976-4	I-kappa-B kinase gamma	IKBKG	Q9Y6K9	0.002388	0.170993
seq.10801.11	10801-11	Ephrin-A2	EFNA2	O43921	0.002714	0.387046
seq.11431.235	11431-235	ATP-dependent DNA helicase Q1	RECQL	P46063	0.002714	1.05022
seq.13126.52	13126-52	Desmocollin-2	DSC2	Q02487	0.002714	0.717748
seq.17836.17	17836-17	Protein S100-A16	S100A16	Q96FQ6	0.002714	1.006943
seq.19161.1	19161-1	Ubiquitin carboxyl-terminal hydrolase 15	USP15	Q9Y4E8	0.002714	0.725135
seq.19254.125	19254-125	GMP reductase 1	GMPR	P36959	0.002714	0.2145
seq.21147.9	21147-9	Fructosamine-3-kinase	FN3K	Q9H479	0.002714	1.529409
seq.24474.12	24474-12	Tetratricopeptide repeat protein 9A	TTC9	Q92623	0.002883	0.662366
seq.19277.4	19277-4	Thiosulfate sulfurtransferase/rhodanese-like domain-containing protein 1	TSTD1	Q8NFU3	0.002883	0.569479
seq.13634.209	13634-209	Pirin	PIR	O00625	0.002883	0.543083
seq.10479.18	10479-18	Stromelysin-2	MMP10	P09238	0.003112	0.253518
seq.12475.48	12475-48	Chloride intracellular channel protein 5	CLIC5	Q9NZA1	0.003223	1.633819
seq.14124.6	14124-6	Ephrin-A2	EFNA2	O43921	0.003223	0.73889
seq.6207.10	6207-10	Prosaposin	PSAP	P07602	0.003223	0.370726
seq.6923.1	6923-1	Procollagen-lysine,2-oxoglutarate 5-dioxygenase 2	PLOD2	O00469	0.003223	0.813302
seq.8039.41	8039-41	Protein FAM177A1	FAM177A1	Q8N128	0.003223	0.704989
seq.12714.38	12714-38	AP-1 complex subunit gamma-like 2	AP1G2	O75843	0.003263	0.445991
seq.15394.79	15394-79	Netrin receptor UNC5B	UNC5B	Q8IZJ1	0.003527	0.280118
seq.23528.199	23528-199	Phytanoyl-CoA hydroxylase-interacting protein-like	PHYHIPL	Q96FC7	0.003706	0.37946

seq.13937.75	13937-75	Cullin-associated NEDD8-dissociated protein 1	CAND1	Q86VP6	0.003706	1.14012
seq.15491.20	15491-20	Endosialin	CD248	Q9HCU0	0.003706	1.018484
seq.16890.37	16890-37	ADAMTS-like protein 1	ADAMTSL1	Q8N6G6	0.003706	-0.73791
seq.20550.38	20550-38	Neurotrimin	NTM	Q9P121	0.003706	1.217306
seq.21384.2	21384-2	Alpha-L-fucoside fucohydrolase	FUCA1	P04066	0.003706	0.599872
seq.22989.24	22989-24	Protein lin-7 homolog A	LIN7A	O14910	0.003706	0.972745
seq.6379.62	6379-62	ADAMTS-like protein 2	ADAMTSL2	Q86TH1	0.003706	0.365663
seq.6645.53	6645-53	Periostin	POSTN	Q15063	0.003706	0.967746
seq.6947.4	6947-4	Type 2 lactosamine alpha-2,3-sialyltransferase	ST3GAL6	Q9Y274	0.003706	0.562722
seq.8474.6	8474-6	Inactive tyrosine-protein kinase transmembrane receptor ROR1	ROR1	Q01973	0.003706	0.669711
seq.8901.40	8901-40	14-3-3 protein gamma	YWHAG	P61981	0.003706	1.209133
seq.13940.19	13940-19	Gamma-interferon-inducible protein 16:Isoform 2, Hematopoietic expression, interferon-inducible nature, and nuclear localization 1	IFI16	Q16666	0.003854	0.233135
seq.2837.3	2837-3	Hepatocyte growth factor receptor	MET	P08581	0.004139	0.193521
seq.13680.3	13680-3	NGFI-A-binding protein 2	NAB2	Q15742	0.004139	0.20201
seq.8094.20	8094-20	CDGSH iron-sulfur domain-containing protein 2	CISD2	Q8N5K1	0.004363	-0.07606
seq.10612.18	10612-18	Procollagen-lysine,2-oxoglutarate 5-dioxygenase 3	PLOD3	O60568	0.004363	0.979435
seq.13381.49	13381-49	Beta-1,4-galactosyltransferase 1	B4GALT1	P15291	0.004363	0.409518
seq.15316.262	15316-262	Thioredoxin-like protein 4B	TXNL4B	Q9NX01	0.004363	0.527342
seq.15566.10	15566-10	Calponin-1	CNN1	P51911	0.004363	0.860359
seq.23659.2	23659-2	Basic leucine zipper and W2 domain-containing protein 2	BZW2	Q9Y6E2	0.004363	1.457446
seq.2938.55	2938-55	Apolipoprotein E (isoform E4)	APOE	P02649	0.004363	0.479943
seq.23394.125	23394-125	Growth arrest-specific protein 2	GAS2	O43903	0.004665	1.05402
seq.5509.7	5509-7	Epidermal growth factor:Extracellular domain	EGF	P01133	0.005008	0.143577
seq.3315.15	3315-15	Glypican-2	GPC2	Q8N158	0.005008	0.206149
seq.17826.341	17826-341	Elongation factor 1-delta	EEF1D	P29692	0.005126	1.189328
seq.21221.67	21221-67	Serpin B9	SERPINB9	P50453	0.005126	1.195766
seq.21705.33	21705-33	Meteorin-like protein	METRNL	Q641Q3	0.005126	0.411455
seq.2700.56	2700-56	Vitamin K-dependent protein S	PROS1	P07225	0.005126	1.2534
seq.3435.53	3435-53	Fibronectin Fragment 4	FN1	P02751	0.005126	0.944981
seq.5012.67	5012-67	Adenylate kinase isoenzyme 1	AK1	P00568	0.005126	1.180752
seq.9018.38	9018-38	Protocadherin-10:Extracellular domain	PCDH10	Q9P2E7	0.005126	0.5364
seq.15468.14	15468-14	Complement factor H-related protein 1	CFHR1	Q03591	0.005952	0.34638
seq.4903.72	4903-72	Calcineurin	PPP3CA|PPP3R1	Q08209|P63098	0.005952	1.107997
seq.13552.7	13552-7	Switch-associated protein 70	SWAP70	Q9UH65	0.005952	0.444781
seq.15447.45	15447-45	Sorbitol dehydrogenase	SORD	Q00796	0.005952	0.949343
seq.15483.377	15483-377	Agrin	AGRN	O00468	0.005952	1.018618
seq.16828.8	16828-8	Collagen alpha-1(VI) chain	COL6A1	P12109	0.005952	0.582828
seq.20943.14	20943-14	N-alpha-acetyltransferase 50	NAA50	Q9GZZ1	0.005952	1.272779
seq.22527.4	22527-4	PDZ and LIM domain protein 3	PDLIM3	Q53GG5	0.005952	2.13274
seq.23521.29	23521-29	Nucleoside diphosphate kinase 7	NME7	Q9Y5B8	0.005952	1.167472
seq.3457.57	3457-57	Periostin	POSTN	Q15063	0.005952	0.778304
seq.7806.33	7806-33	Beta-1,4-galactosyltransferase 7	B4GALT7	Q9UBV7	0.005952	0.2248
seq.9870.17	9870-17	Tryptophan--tRNA ligase, cytoplasmic	WARS1	P23381	0.005952	1.42802
seq.19584.33	19584-33	Fibroblast growth factor 9	FGF9	P31371	0.007011	0.161856
seq.21109.1	21109-1	Cilia- and flagella-associated protein 36	CFAP36	Q96G28	0.007011	1.164653
seq.5675.6	5675-6	Tenascin	TNC	P24821	0.007011	0.373952
seq.7096.30	7096-30	Regulator of microtubule dynamics protein 1	RMDN1	Q96DB5	0.007011	-0.69625
seq.7788.1	7788-1	ATP synthase-coupling factor 6, mitochondrial	ATP5PF	P18859	0.007011	-0.71258
seq.8974.172	8974-172	Collagen alpha-1(XV) chain	COL15A1	P39059	0.007011	0.15505
seq.9244.27	9244-27	Palmitoyl-protein thioesterase 1	PPT1	P50897	0.007011	0.761126
seq.9294.45	9294-45	Microfibrillar-associated protein 2	MFAP2	P55001	0.007011	-0.92664
seq.13658.31	13658-31	Platelet-derived growth factor C	PDGFC	Q9NRA1	0.007011	0.248161
seq.9090.9	9090-9	Synaptotagmin-like protein 4:Ca2+-dependent membrane-targeting module 1	SYTL4	Q96C24	0.007011	0.189045
seq.22967.15	22967-15	Growth/differentiation factor 7	GDF7	Q7Z4P5	0.007448	0.205036
seq.3298.52	3298-52	Contactin-4	CNTN4	Q8IWV2	0.007448	0.178533
seq.6557.50	6557-50	Leucine-rich repeat-containing protein 15	LRRC15	Q8TF66	0.007448	0.254188
seq.9482.110	9482-110	ADP-ribose pyrophosphatase, mitochondrial	NUDT9	Q9BW91	0.007448	1.235452
seq.23695.1	23695-1	Fibronectin type III and SPRY domain-containing protein 1	FSD1	Q9BTV5	0.007961	0.367639
seq.10451.11	10451-11	Nucleobindin-1	NUCB1	Q02818	0.007961	0.461889
seq.10800.15	10800-15	Serpin H1	SERPINH1	P50454	0.007961	1.175353
seq.12022.12	12022-12	Mothers against decapentaplegic homolog 4	SMAD4	Q13485	0.007961	0.503957
seq.16756.30	16756-30	Growth/differentiation factor 7	GDF7	Q7Z4P5	0.007961	0.206431
seq.17156.72	17156-72	Serine/threonine-protein kinase DCLK1	DCLK1	O15075	0.007961	0.385957
seq.20091.138	20091-138	Ephrin-A1	EFNA1	P20827	0.007961	0.849122
seq.23566.6	23566-6	Apolipoprotein-L2	APOL2	Q9BQE5	0.007961	0.901873
seq.3077.66	3077-66	Coagulation factor Xa	F10	P00742	0.007961	0.650039
seq.6927.7	6927-7	Bifunctional heparan sulfate N-deacetylase/N-sulfotransferase 1	NDST1	P52848	0.007961	0.193448
seq.8428.102	8428-102	Neurotrimin	NTM	Q9P121	0.007961	0.421955
seq.17436.193	17436-193	Casein kinase I isoform gamma-2	CSNK1G2	P78368	0.008176	0.192834
seq.7049.2	7049-2	Disintegrin and metalloproteinase domain-containing protein 23	ADAM23	O75077	0.008187	0.165199
seq.8300.82	8300-82	Peroxisomal membrane protein PEX14:N-term	PEX14	O75381	0.008187	-0.4137
seq.4842.62	4842-62	Glypican-3	GPC3	P51654	0.008763	0.484878
seq.19639.53	19639-53	Islet amyloid polypeptide	IAPP	P10997	0.008763	-0.1248
seq.7227.75	7227-75	Cochlin	COCH	O43405	0.009133	-0.1351
seq.22969.12	22969-12	C-C motif chemokine 7	CCL7	P80098	0.009133	0.187672
seq.14273.19	14273-19	Prolyl endopeptidase	PREP	P48147	0.009133	1.859581
seq.4878.3	4878-3	Coagulation Factor X	F10	P00742	0.009133	0.386352
seq.12869.68	12869-68	Probable ATP-dependent RNA helicase DDX6	DDX6	P26196	0.009133	1.195876
seq.13986.6	13986-6	LanC-like protein 1	LANCL1	O43813	0.009133	0.872519
seq.15594.47	15594-47	Serine protease HTRA1	HTRA1	Q92743	0.009133	0.739386

seq.18910.45	18910-45	Protein lin-7 homolog A	LIN7A	O14910	0.009133	0.873095
seq.2278.61	2278-61	Metalloproteinase inhibitor 2	TIMP2	P16035	0.009133	0.755264
seq.15620.4	15620-4	Neuroligin-1	NLGN1	Q8N2Q7	0.009783	0.12333
seq.8859.51	8859-51	Carbonic anhydrase-related protein 11	CA11	O75493	0.009783	0.274628
seq.16307.22	16307-22	Netrin receptor UNC5D	UNC5D	Q6UXZ4	0.010479	0.207027
seq.20387.277	20387-277	Coiled-coil-helix-coiled-coil-helix domain-containing protein 7	CHCHD7	Q9BUK0	0.010479	0.364264
seq.12703.6	12703-6	Serine/threonine-protein kinase Nek7	NEK7	Q8TDX7	0.010766	1.223541
seq.3045.72	3045-72	Pleiotrophin	PTN	P21246	0.010766	1.93917
seq.3196.6	3196-6	Hyaluronan and proteoglycan link protein 1	HAPLN1	P10915	0.010766	1.860653
seq.6909.40	6909-40	Alpha-1,6-mannosyl-glycoprotein 2-beta-N-acetylglucosaminyltransferase	MGAT2	Q10469	0.011043	0.136188
seq.19560.23	19560-23	Plexin-A4	PLXNA4	Q9HCM2	0.011043	-0.17116
seq.17777.31	17777-31	Serine dehydratase-like	SDSL	Q96GA7	0.011043	0.164787
seq.8811.24	8811-24	BMP and activin membrane-bound inhibitor homolog:Extracellular domain	BAMBI	Q13145	0.011869	0.093717
seq.12628.31	12628-31	LanC-like protein 2	LANCL2	Q9NS86	0.012223	0.994739
seq.15441.6	15441-6	Ganglioside GM2 activator	GM2A	P17900	0.012223	0.482889
seq.17332.3	17332-3	Poly(ADP-ribose) glycohydrolase ARH3	ADPRS	Q9NX46	0.012223	1.086154
seq.17722.5	17722-5	Peptidyl-prolyl cis-trans isomerase FKBP4	FKBP4	Q02790	0.012223	1.168012
seq.18233.10	18233-10	Aspartate aminotransferase, mitochondrial	GOT2	P00505	0.012223	-0.57036
seq.23300.3	23300-3	RNA-binding protein with multiple splicing 2	RBPMS2	Q6ZRY4	0.012223	0.812328
seq.23903.3	23903-3	Aspartate aminotransferase, mitochondrial	GOT2	P00505	0.012223	-0.72652
seq.25256.23	25256-23	Elongation factor 1-alpha 1	EEF1A1	P68104	0.012223	1.074046
seq.2615.60	2615-60	Ephrin-A5	EFNA5	P52803	0.012223	0.535216
seq.5489.18	5489-18	Stress-induced-phosphoprotein 1	STIP1	P31948	0.012223	0.805534
seq.5496.49	5496-49	Spondin-1	SPON1	Q9HCB6	0.012223	1.506187
seq.12656.1	12656-1	Kinesin light chain 1	KLC1	Q07866	0.012223	0.285444
seq.18403.25	18403-25	AMP deaminase 2	AMPD2	Q01433	0.012983	0.38476
seq.6531.29	6531-29	Protein FAM162A	FAM162A	Q96A26	0.012983	-0.12809
seq.4874.3	4874-3	Angiogenin	ANG	P03950	0.012983	0.182955
seq.5129.12	5129-12	Scavenger receptor class F member 1	SCARF1	Q14162	0.012983	0.166289
seq.17676.13	17676-13	Cdc42-interacting protein 4	TRIP10	Q15642	0.014224	0.719738
seq.3336.50	3336-50	Tissue factor pathway inhibitor	TFPI	P10646	0.014224	1.451734
seq.3607.71	3607-71	Dickkopf-related protein 3	DKK3	Q9UBP4	0.014224	0.45903
seq.3889.64	3889-64	Lamin-B1	LMNB1	P20700	0.014224	-0.06751
seq.6400.33	6400-33	Tumor protein p53-inducible protein 13	TP53I13	Q8NBR0	0.014224	0.901277
seq.8687.26	8687-26	Transmembrane protein 106B	TMEM106B	Q9NUM4	0.014676	-0.1727
seq.23597.11	23597-11	Arf-GAP domain and FG repeat-containing protein 2	AGFG2	O95081	0.014676	0.292756
seq.10569.28	10569-28	Microfibrillar-associated protein 2	MFAP2	P55001	0.015997	-0.8239
seq.11150.3	11150-3	Collagen alpha-1(VI) chain	COL6A1	P12109	0.015997	0.31438
seq.12630.8	12630-8	Arfaptin-2	ARFIP2	P53365	0.015997	0.484367
seq.12643.4	12643-4	Beta-arrestin-1	ARRB1	P49407	0.015997	1.025056
seq.17675.17	17675-17	Acyl-coenzyme A thioesterase 13	ACOT13	Q9NPJ3	0.015997	-0.67868
seq.18186.15	18186-15	Threonine--tRNA ligase, cytoplasmic	TARS1	P26639	0.015997	1.200713
seq.18823.52	18823-52	Histone H2B type 3-B	H2BU1	Q8N257	0.015997	-0.58742
seq.24226.30	24226-30	Fascin	FSCN1	Q16658	0.015997	1.294412
seq.2579.17	2579-17	Matrix metalloproteinase-9	MMP9	P14780	0.015997	0.517696
seq.2625.53	2625-53	Hsp90alpha	HSP90AA1	P07900	0.015997	1.325122
seq.3858.5	3858-5	Low molecular weight phosphotyrosine protein phosphatase	ACP1	P24666	0.015997	0.288764
seq.4258.15	4258-15	Proliferation-associated protein 2G4	PA2G4	Q9UQ80	0.015997	1.238025
seq.5139.32	5139-32	Netrin receptor UNC5C	UNC5C	O95185	0.015997	-0.74853
seq.5699.19	5699-19	Protein FAM189A2	FAM189A2	Q15884	0.015997	0.155772
seq.11514.196	11514-196	CD59 glycoprotein	CD59	P13987	0.015997	0.108708
seq.15446.25	15446-25	Neuregulin-1, sensory and motor neuron-derived factor isoform	NRG1	Q02297	0.015997	0.192049
seq.2860.19	2860-19	Importin subunit alpha-1	KPNA2	P52292	0.015997	0.633361
seq.3174.2	3174-2	A disintegrin and metalloproteinase with thrombospondin motifs 1	ADAMTS1	Q9UHI8	0.015997	-0.08855
seq.8035.6	8035-6	Uncharacterized protein C1orf198	C1orf198	Q9H425	0.0171	0.132511
seq.5465.32	5465-32	Heparan-sulfate 6-O-sulfotransferase 1	HS6ST1	O60243	0.0171	0.867703
seq.11365.17	11365-17	Teneurin-4	TENM4	Q6N022	0.017779	-0.08327
seq.11830.48	11830-48	Tyrosine-protein phosphatase non-receptor type 11	PTPN11	Q06124	0.017779	1.253099
seq.12456.5	12456-5	6-phosphofructo-2-kinase/fructose-2,6-bisphosphatase 3	PFKFB3	Q16875	0.017779	0.336854
seq.13636.20	13636-20	Nucleosome assembly protein 1-like 1	NAP1L1	P55209	0.017779	1.168642
seq.14143.8	14143-8	Histone H2B type 2-E	H2BC21	Q16778	0.017779	-0.56582
seq.15522.2	15522-2	Golgi-associated plant pathogenesis-related protein 1	GLIPR2	Q9H4G4	0.017779	0.698739
seq.15545.13	15545-13	Calcineurin subunit B type 1	PPP3R1	P63098	0.017779	0.533291
seq.17686.27	17686-27	Tubulin-folding cofactor B	TBCB	Q99426	0.017779	1.033622
seq.17781.191	17781-191	Microtubule-associated proteins 1A/1B light chain 3A	MAP1LC3A	Q9H492	0.017779	0.938316
seq.18188.12	18188-12	Glycine amidinotransferase, mitochondrial	GATM	P50440	0.017779	-0.35297
seq.18819.21	18819-21	Peptidyl-prolyl cis-trans isomerase C	PPIC	P45877	0.017779	0.61087
seq.21383.37	21383-37	Asparaginyl-tRNA synthetase, cytoplasmic	NARS1	O43776	0.017779	1.128335
seq.21945.4	21945-4	Protein Wnt-5b	WNT5B	Q9H1J7	0.017779	-0.73848
seq.22402.12	22402-12	Histone H2A type 1-A	H2AC1	Q96QV6	0.017779	-0.49177
seq.4159.130	4159-130	Complement factor H	CFH	P08603	0.017779	0.217458
seq.7770.25	7770-25	NFU1 iron-sulfur cluster scaffold homolog, mitochondrial	NFU1	Q9UMS0	0.017779	-0.1088
seq.7928.183	7928-183	Protein-tyrosine sulfotransferase 1	TPST1	O60507	0.017779	0.28691
seq.24685.12	24685-12	Sorting nexin-11	SNX11	Q9Y5W9	0.01859	0.694039
seq.12688.115	12688-115	Ribosomal protein S6 kinase alpha-6	RPS6KA6	Q9UK32	0.019734	0.419959
seq.16583.8	16583-8	Bis(5'-nucleosyl)-tetraphosphatase [asymmetrical]	NUDT2	P50583	0.019734	0.965387
seq.24655.23	24655-23	RILP-like protein 2	RILPL2	Q969X0	0.019734	0.06618
seq.21595.8	21595-8	Coiled-coil domain-containing protein 43	CCDC43	Q96MW1	0.019734	0.38295
seq.10746.24	10746-24	Dickkopf-related protein 3	DKK3	Q9UBP4	0.019957	0.50553
seq.11692.21	11692-21	SHC-transforming protein 4	SHC4	Q6S5L8	0.019957	-0.06917
seq.12617.2	12617-2	Serine/threonine-protein kinase 24	STK24	Q9Y6E0	0.019957	0.83994
seq.15534.26	15534-26	Malate dehydrogenase, mitochondrial	MDH2	P40926	0.019957	-0.70929

seq.20458.22	20458-22	NADH dehydrogenase [ubiquinone] 1 alpha subcomplex subunit 2	NDUFA2	O43678	0.019957	-0.42039
seq.20590.13	20590-13	Neuropeptide Y	NPY	P01303	0.019957	0.358995
seq.23278.13	23278-13	39S ribosomal protein L12, mitochondrial	MRPL12	P52815	0.019957	-0.75864
seq.24717.26	24717-26	Serine/threonine-protein phosphatase 2A regulatory subunit B'	PTPA	Q15257	0.019957	0.962351
seq.3284.75	3284-75	Biglycan	BGN	P21810	0.019957	1.117344
seq.3364.76	3364-76	Cathepsin L2	CTSV	O60911	0.019957	-0.80492
seq.3388.58	3388-58	Serine/threonine-protein kinase PAK 5	PAK5	Q9P286	0.019957	-0.05644
seq.7141.21	7141-21	Alpha-1,3-mannosyl-glycoprotein 4-beta-N-acetylglucosaminyltransferase B	MGAT4B	Q9UQ53	0.019957	0.178876
seq.9952.57	9952-57	Transcription factor A, mitochondrial	TFAM	Q00059	0.019957	-0.66181
seq.9986.14	9986-14	Neuropeptide W	NPW	Q8N729	0.019957	0.219359
seq.21891.31	21891-31	Fibulin-7	FBLN7	Q53RD9	0.020133	0.240903
seq.12670.15	12670-15	Cell cycle checkpoint protein RAD1	RAD1	O60671	0.020133	0.633261
seq.2889.37	2889-37	Cardiotrophin-1	CTF1	Q16619	0.020133	0.351374
seq.16892.23	16892-23	Ectonucleotide pyrophosphatase/phosphodiesterase family member 2	ENPP2	Q13822	0.021563	-0.16827
seq.3469.74	3469-74	Ribosomal protein S6 kinase alpha-3	RPS6KA3	P51812	0.022531	0.708635
seq.19372.7	19372-7	MAM domain-containing glycosylphosphatidylinositol anchor protein 2	MDGA2	Q7Z553	0.022531	0.322752
seq.8565.160	8565-160	Plasmalemma vesicle-associated protein	PLVAP	Q9BX97	0.022531	-0.07696
seq.3422.4	3422-4	Cyclin-dependent kinase 1:G2/mitotic-specific cyclin-B1 complex	CDK1|CCNB1	P06493|P14635	0.022531	-0.05262
seq.12436.84	12436-84	Glutathione S-transferase omega-1	GSTO1	P78417	0.022531	0.388961
seq.15526.33	15526-33	Glutathione synthetase	GSS	P48637	0.022531	0.7051
seq.18821.9	18821-9	C4a anaphylatoxin	C4A|C4B	P0C0L4|P0C0L5	0.022531	-0.23556
seq.22974.25	22974-25	Histone H2B type 2-E	H2BC21	Q16778	0.022531	-0.47842
seq.24235.2	24235-2	EH domain-containing protein 1	EHD1	Q9H4M9	0.022531	0.851569
seq.2443.10	2443-10	Fibroblast growth factor 8 isoform B	FGF8	P55075	0.022531	-0.09981
seq.5888.29	5888-29	Eukaryotic translation initiation factor 5A-1	EIF5A	P63241	0.022531	0.769914
seq.9233.71	9233-71	Tissue factor pathway inhibitor 2	TFPI2	P48307	0.022531	0.853035
seq.9278.9	9278-9	Stromal cell-derived factor 1	CXCL12	P48061	0.022531	1.424387
seq.5701.81	5701-81	Tetranectin	CLEC3B	P05452	0.023641	-0.05107
seq.7251.64	7251-64	Complement C1q tumor necrosis factor-related protein 3	C1QTNF3	Q9BXJ4	0.023727	-0.06993
seq.8095.213	8095-213	Peptidyl-prolyl cis-trans isomerase F, mitochondrial	PPIF	P30405	0.023727	-0.69282
seq.8089.173	8089-173	Nuclear receptor subfamily 4 group A member 1	NR4A1	P22736	0.025273	-0.07945
seq.20367.6	20367-6	L-amino-acid oxidase	IL4I1	Q96RQ9	0.025273	-0.08046
seq.12649.80	12649-80	Malate dehydrogenase, mitochondrial	MDH2	P40926	0.025273	-0.64179
seq.13132.14	13132-14	Semaphorin-5A	SEMA5A	Q13591	0.025273	0.724276
seq.13622.16	13622-16	Serine/threonine-protein phosphatase 2A 56 kDa regulatory subunit alpha isoform	PPP2R5A	Q15172	0.025273	0.954573
seq.17729.20	17729-20	Ubiquitin-conjugating enzyme E2 S	UBE2S	Q16763	0.025273	0.393579
seq.18348.89	18348-89	Heterogeneous nuclear ribonucleoprotein D0	HNRNPD	Q14103	0.025273	0.872952
seq.19267.14	19267-14	Hydroxyacylglutathione hydrolase, mitochondrial	HAGH	Q16775	0.025273	-0.27068
seq.19311.15	19311-15	Beta-crystallin S	CRYGS	P22914	0.025273	0.49062
seq.22403.13	22403-13	Histone H2B type 1-K	H2BC12	O60814	0.025273	-0.52154
seq.22468.54	22468-54	Histone H2A type 1	H2AC11	P0C0S8	0.025273	-0.49283
seq.25285.14	25285-14	Kelch repeat and BTB domain-containing protein 11	KBTBD11	O94819	0.025273	0.641246
seq.4775.34	4775-34	Gelsolin	GSN	P06396	0.025273	0.936088
seq.4880.21	4880-21	Growth/differentiation factor 2	GDF2	Q9UK05	0.025273	-0.04324
seq.5467.15	5467-15	Heat shock protein HSP 90-beta	HSP90AB1	P08238	0.025273	0.805957
seq.5654.70	5654-70	Protein disulfide-isomerase TMX3	TMX3	Q96JJ7	0.025273	0.696276
seq.16599.38	16599-38	GPN-loop GTPase 1	GPN1	Q9HCN4	0.026052	1.000648
seq.13580.2	13580-2	UDP-N-acetylhexosamine pyrophosphorylase	UAP1	Q16222	0.027634	0.267582
seq.5742.14	5742-14	Lysophosphatidic acid phosphatase type 6	ACP6	Q9NPH0	0.027634	0.35781
seq.7970.315	7970-315	Ecto-ADP-ribosyltransferase 3	ART3	Q13508	0.027634	0.624617
seq.11355.10	11355-10	Eukaryotic translation initiation factor 5A-2	EIF5A2	Q9GZV4	0.028548	0.748324
seq.12621.55	12621-55	Serine/threonine-protein phosphatase 2A 65 kDa regulatory subunit A alpha isoform	PPP2R1A	P30153	0.028548	0.796793
seq.13967.14	13967-14	Thioredoxin reductase 1, cytoplasmic	TXNRD1	Q16881	0.028548	0.715075
seq.13969.24	13969-24	Importin subunit alpha-7	KPNA6	O60684	0.028548	0.400934
seq.16616.137	16616-137	Beta-enolase	ENO3	P13929	0.028548	0.7754
seq.16885.49	16885-49	Microtubule-associated protein RP/EB family member 3	MAPRE3	Q9UPY8	0.028548	1.206366
seq.18884.22	18884-22	DnaJ homolog subfamily B member 4	DNAJB4	Q9UDY4	0.028548	0.704653
seq.19189.2	19189-2	Ubiquitin carboxyl-terminal hydrolase 14	USP14	P54578	0.028548	1.142796
seq.4143.74	4143-74	Lymphotactin	XCL1	P47992	0.028548	-0.07751
seq.5000.52	5000-52	Galectin-3-binding protein	LGALS3BP	Q08380	0.028548	0.271356
seq.6049.64	6049-64	Receptor-type tyrosine-protein phosphatase S	PTPRS	Q13332	0.028548	1.006993
seq.19360.22	19360-22	Leucine-rich repeat transmembrane neuronal protein 1	LRRTM1	Q86UE6	0.028548	0.068108
seq.10014.31	10014-31	Zinc finger protein SNAI2	SNAI2	O43623	0.028548	-0.05778
seq.7853.19	7853-19	Protein SCO1 homolog, mitochondrial	SCO1	O75880	0.028548	-0.13527
seq.23361.20	23361-20	RNA-binding protein 4	RBM4	Q9BWF3	0.030508	0.399059
seq.12449.16	12449-16	Peptidyl-prolyl cis-trans isomerase H	PPIH	O43447	0.03222	0.800657
seq.21120.3	21120-3	Mimitin, mitochondrial	NDUFAF2	Q8N183	0.03222	-0.2664
seq.24670.1	24670-1	PIH1 domain-containing protein 1	PIH1D1	Q9NWS0	0.03222	0.192095
seq.13059.33	13059-33	Riboflavin kinase	RFK	Q969G6	0.03222	0.373158
seq.17748.21	17748-21	Quinone oxidoreductase PIG3	TP53I3	Q53FA7	0.03222	-0.2464
seq.21963.48	21963-48	Disabled homolog 2	DAB2	P98082	0.03222	0.503655
seq.23542.8	23542-8	Golgi phosphoprotein 3-like	GOLPH3L	Q9H4A5	0.03222	0.729351
seq.23705.42	23705-42	CTP synthase 1	CTPS1	P17812	0.03222	0.678992
seq.24425.8	24425-8	Methionyl-tRNA synthetase, cytoplasmic	MARS1	P56192	0.03222	1.134549
seq.24710.1	24710-1	Microtubule-associated tumor suppressor candidate 2	MTUS2	Q5JR59	0.03222	-0.21521
seq.25126.19	25126-19	E3 ubiquitin-protein ligase HECW2	HECW2	Q9P2P5	0.03222	0.428099
seq.9453.12	9453-12	Vascular endothelial growth factor B	VEGFB	P49765	0.03222	0.191939
seq.14615.46	14615-46	Keratin-associated protein 2-4	KRTAP2-4	Q9BYR9	0.033631	0.091567
seq.6575.79	6575-79	ADAMTS-like protein 1	ADAMTSL1	Q8N6G6	0.033764	-0.10289
seq.24446.65	24446-65	Muscleblind-like protein 2	MBNL2	Q5VZF2	0.035778	0.618836

seq.13710.6	13710-6	Plasma protease C1 inhibitor	SERPING1	P05155	0.035778	0.245087
seq.17742.2	17742-2	Ras-related protein R-Ras	RRAS	P10301	0.035778	0.193521
seq.7196.21	7196-21	Interferon omega-1	IFNW1	P05000	0.035778	-0.07515
seq.12438.127	12438-127	DNA-3-methyladenine glycosylase	MPG	P29372	0.036131	0.278934
seq.14144.3	14144-3	Histone H2A type 3	H2AW	Q7L7L0	0.036131	-0.43018
seq.18833.76	18833-76	Astrocytic phosphoprotein PEA-15	PEA15	Q15121	0.036131	0.748342
seq.21162.30	21162-30	Tubulin-specific chaperone cofactor E-like protein	TBCEL	Q5QJ74	0.036131	1.175945
seq.21180.16	21180-16	PI-PLC X domain-containing protein 3	PLCXD3	Q63HM9	0.036131	1.347151
seq.23241.27	23241-27	Mitochondrial import inner membrane translocase subunit Tim10 B	TIMM10B	Q9Y5J6	0.036131	-0.22421
seq.2949.6	2949-6	Group 10 secretory phospholipase A2	PLA2G10	O15496	0.036131	-0.07232
seq.3170.6	3170-6	Methionine aminopeptidase 2	METAP2	P50579	0.036131	1.106795
seq.4471.50	4471-50	Protein-glutamine gamma-glutamyltransferase E	TGM3	Q08188	0.036131	0.680592
seq.5018.68	5018-68	Peroxiredoxin-6	PRDX6	P30041	0.036131	0.661896
seq.6285.71	6285-71	Malectin	MLEC	Q14165	0.036131	0.940772
seq.9762.14	9762-14	Vasodilator-stimulated phosphoprotein	VASP	P50552	0.036131	0.765675
seq.8273.84	8273-84	Interleukin-31 receptor subunit alpha	IL31RA	Q8NI17	0.03709	-0.08858
seq.15511.37	15511-37	Neuronal pentraxin receptor	NPTXR	O95502	0.039013	0.105611
seq.23363.41	23363-41	Poly(rC)-binding protein 2	PCBP2	Q15366	0.039013	0.486526
seq.21436.56	21436-56	Cysteine protease ATG4A	ATG4A	Q8WYN0	0.039013	0.153159
seq.15318.75	15318-75	Protein S100-A10	S100A10	P60903	0.039013	0.381624
seq.16591.71	16591-71	Acidic mammalian chitinase	CHIA	Q9BZP6	0.039013	-0.14767
seq.17752.24	17752-24	Arginase-2, mitochondrial	ARG2	P78540	0.039013	-0.07325
seq.6260.14	6260-14	Tenascin	TNC	P24821	0.039013	0.971797
seq.11608.5	11608-5	Microtubule-associated proteins 1A/1B light chain 3B	MAP1LC3B	Q9GZQ8	0.04038	0.927177
seq.12340.17	12340-17	Alanine--tRNA ligase, cytoplasmic	AARS1	P49588	0.04038	0.841335
seq.18317.111	18317-111	AN1-type zinc finger protein 5	ZFAND5	O76080	0.04038	0.742656
seq.19188.21	19188-21	nucleosome assembly protein 1-like 4	NAP1L4	Q99733	0.04038	1.102422
seq.19297.4	19297-4	Glucose-6-phosphate 1-dehydrogenase	G6PD	P11413	0.04038	0.937504
seq.23595.6	23595-6	Laccase domain-containing protein 1	LACC1	Q8IV20	0.04038	0.196326
seq.24459.15	24459-15	Coatomer subunit beta'	COPB2	P35606	0.04038	1.11823
seq.25286.33	25286-33	Rab GTPase-activating protein 1-like	RABGAP1L	Q5R372	0.04038	0.757025
seq.9796.4	9796-4	Bile salt-activated lipase	CEL	P19835	0.04038	0.285327
seq.4145.58	4145-58	Neurotrophin-3	NTF3	P20783	0.04038	-0.12509
seq.15323.112	15323-112	Protein argonaute-3	AGO3	Q9H9G7	0.04038	0.491464
seq.21713.11	21713-11	Reelin	RELN	P78509	0.04038	0.339411
seq.2730.58	2730-58	MHC class I polypeptide-related sequence A	MICA	Q29983	0.04038	-0.22382
seq.3516.60	3516-60	Stromal cell-derived factor 1	CXCL12	P48061	0.04038	0.407438
seq.10082.251	10082-251	Neurofilament light polypeptide	NEFL	P07196	0.042858	0.47461
seq.9760.13	9760-13	Mitogen-activated protein kinase 9	MAPK9	P45984	0.042858	0.890591
seq.14287.6	14287-6	Ras-related protein Rab-5C	RAB5C	P51148	0.042858	0.415047
seq.10554.23	10554-23	Beta-galactosidase	GLB1	P16278	0.044015	0.245398
seq.21184.1	21184-1	Biogenesis of lysosome-related organelles complex 1 subunit 2	BLOC1S2	Q6QNY1	0.044591	0.197848
seq.7878.2	7878-2	Protein transport protein Sec61 subunit beta	SEC61B	P60468	0.044591	-0.07568
seq.12569.25	12569-25	T-complex protein 1 subunit epsilon	CCT5	P48643	0.044591	0.780973
seq.12575.30	12575-30	C-1-tetrahydrofolate synthase, cytoplasmic	MTHFD1	P11586	0.044591	0.822814
seq.12620.3	12620-3	Septin-11	SEPTIN11	Q9NVA2	0.044591	0.796711
seq.12747.89	12747-89	RNA-binding protein 3	RBM3	P98179	0.044591	0.944276
seq.18175.65	18175-65	Synaptic vesicle membrane protein VAT-1 homolog	VAT1	Q99536	0.044591	1.192814
seq.23638.3	23638-3	Carnosine N-methyltransferase	CARNMT1	Q8N4J0	0.044591	0.502651
seq.23666.35	23666-35	Bifunctional purine biosynthesis protein PURH	ATIC	P31939	0.044591	0.956377
seq.2879.9	2879-9	Alpha-1-antichymotrypsin	SERPINA3	P01011	0.044591	0.496971
seq.3340.53	3340-53	Thrombospondin-4	THBS4	P35443	0.044591	0.354769
seq.4314.12	4314-12	dCTP pyrophosphatase 1	DCTPP1	Q9H773	0.044591	1.082315
seq.5918.5	5918-5	Proteasome activator complex subunit 1	PSME1	Q06323	0.044591	0.566353
seq.6304.8	6304-8	Complement C1q tumor necrosis factor-related protein 1	C1QTNF1	Q9BXJ1	0.044591	0.299408
seq.8019.73	8019-73	Stathmin-3	STMN3	Q9NZ72	0.044591	0.667622
seq.9394.19	9394-19	Carboxypeptidase Q	CPQ	Q9Y646	0.044591	0.14357
seq.9468.8	9468-8	Vesicular integral-membrane protein VIP36	LMAN2	Q12907	0.044591	0.634715
seq.9864.38	9864-38	Tetratricopeptide repeat protein 1	TTC1	Q99614	0.046877	0.07253
seq.4459.68	4459-68	Proprotein convertase subtilisin/kexin type 7	PCSK7	Q16549	0.049853	-0.07254
seq.12563.2	12563-2	Tumor necrosis factor alpha-induced protein 8	TNFAIP8	O95379	0.049853	-0.12982
seq.13450.49	13450-49	Ubiquitin carboxyl-terminal hydrolase 8	USP8	P40818	0.049853	0.38056
seq.13939.14	13939-14	UTP--glucose-1-phosphate uridylyltransferase	UGP2	Q16851	0.049853	0.959841
seq.16043.30	16043-30	SHC-transforming protein 1:Phosphotyrosine Interaction Domain	SHC1	P29353	0.049853	0.488511
seq.17148.7	17148-7	Flavin reductase (NADPH)	BLVRB	P30043	0.049853	1.527072
seq.17513.11	17513-11	Annexin A11	ANXA11	P50995	0.049853	-0.0555
seq.21445.40	21445-40	Ubiquitin-like protein Nedd8	NEDD8	Q15843	0.049853	1.198868
seq.22381.1	22381-1	Endoribonuclease LACTB2	LACTB2	Q53H82	0.049853	0.947717
seq.23656.9	23656-9	Pseudouridylate synthase 7 homolog	PUS7	Q96PZ0	0.049853	0.473134
seq.2848.2	2848-2	Wnt inhibitory factor 1	WIF1	Q9Y5W5	0.049853	-0.09354
seq.3379.29	3379-29	Protein kinase C iota type	PRKCI	P41743	0.049853	0.9565
seq.3474.19	3474-19	Thrombospondin-1	THBS1	P07996	0.049853	0.317619
seq.3887.90	3887-90	Importin subunit beta-1	KPNB1	Q14974	0.049853	1.035238
seq.8358.30	8358-30	Thioredoxin-dependent peroxide reductase, mitochondrial	PRDX3	P30048	0.049853	-0.28648
seq.9744.139	9744-139	DnaJ homolog subfamily A member 4	DNAJA4	Q8WW22	0.049853	0.678821
seq.9886.28	9886-28	DNA repair protein XRCC4	XRCC4	Q13426	0.049853	0.895617

Table S2. Aptamer based profiling of hiPSC-CMs flex DMD to flex control.

Compiled list of flex control to flex DMD from Fig. 3C of the main text.

Aptamer Name	SeqId	Target	EntrezGeneSymbol	UniProt	p.val.adj	Log_2(FC)
seq.11117.2	11117-2	Spermatogenesis-associated protein 20	SPATA20	Q8TB22	2.23E-05	-0.56643
seq.11140.56	11140-56	Collagen alpha-1(I) chain:C-term propeptide	COL1A1	P02452	2.23E-05	2.86258
seq.11836.144	11836-144	Epithelial discoidin domain-containing receptor 1	DDR1	Q08345	2.23E-05	1.049218
seq.12475.48	12475-48	Chloride intracellular channel protein 5	CLIC5	Q9NZA1	2.23E-05	1.913663
seq.12620.3	12620-3	Septin-11	SEPTIN11	Q9NVA2	2.23E-05	1.092246
seq.12714.38	12714-38	AP-1 complex subunit gamma-like 2	AP1G2	O75843	2.23E-05	0.696728
seq.13122.19	13122-19	Leucine-rich repeat transmembrane protein FLRT2	FLRT2	O43155	2.23E-05	1.590462
seq.13130.150	13130-150	Hexokinase-2	HK2	P52789	2.23E-05	0.983606
seq.13634.209	13634-209	Pirin	PIR	O00625	2.23E-05	0.752086
seq.15522.2	15522-2	Golgi-associated plant pathogenesis-related protein 1	GLIPR2	Q9H4G4	2.23E-05	1.120494
seq.15569.15	15569-15	Collagen Type II	COL2A1	P02458	2.23E-05	1.93501
seq.16606.85	16606-85	Aldose reductase	AKR1B1	P15121	2.23E-05	2.081314
seq.16781.2	16781-2	Cytosolic endo-beta-N-acetylglucosaminidase	ENGASE	Q8NFI3	2.23E-05	1.213826
seq.16872.248	16872-248	Maleylacetoacetate isomerase	GSTZ1	O43708	2.23E-05	-2.0471
seq.18225.13	18225-13	Heme-binding protein 1	HEBP1	Q9NRV9	2.23E-05	1.502514
seq.18417.3	18417-3	Arsenite methyltransferase	AS3MT	Q9HBK9	2.23E-05	0.830762
seq.18871.24	18871-24	Allograft inflammatory factor 1-like	AIF1L	Q9BQI0	2.23E-05	1.197424
seq.18875.125	18875-125	Chondrocalcin	COL2A1	P02458	2.23E-05	2.484122
seq.19254.125	19254-125	GMP reductase 1	GMPR	P36959	2.23E-05	0.501785
seq.19277.4	19277-4	Thiosulfate sulfurtransferase/rhodanese-like domain-containing protein 1	TSTD1	Q8NFU3	2.23E-05	0.916811
seq.20561.15	20561-15	Contactin-6	CNTN6	Q9UQ52	2.23E-05	2.196365
seq.21180.16	21180-16	PI-PLC X domain-containing protein 3	PLCXD3	Q63HM9	2.23E-05	1.480405
seq.21643.8	21643-8	40S ribosomal protein S20	RPS20	P60866	2.23E-05	1.364622
seq.23178.95	23178-95	Histamine N-methyltransferase	HNMT	P50135	2.23E-05	0.813253
seq.23595.6	23595-6	Laccase domain-containing protein 1	LACC1	Q8IV20	2.23E-05	0.573627
seq.2860.19	2860-19	Importin subunit alpha-1	KPNA2	P52292	2.23E-05	0.949396
seq.3045.72	3045-72	Pleiotrophin	PTN	P21246	2.23E-05	1.881119
seq.3327.27	3327-27	Netrin-4	NTN4	Q9HB63	2.23E-05	1.169175
seq.7227.75	7227-75	Cochlin	COCH	O43405	2.23E-05	-0.1825
seq.8403.18	8403-18	Fatty acid synthase	FASN	P49327	2.23E-05	1.112222
seq.9267.2	9267-2	Carboxypeptidase A4	CPA4	Q9UI42	2.23E-05	2.889551
seq.9385.4	9385-4	Lysosomal alpha-glucosidase	GAA	P10253	2.23E-05	1.426766
seq.9793.145	9793-145	Immunoglobulin superfamily DCC subclass member 4	IGDCC4	Q8TDY8	2.23E-05	0.629559
seq.9854.36	9854-36	Aldose reductase	AKR1B1	P15121	2.23E-05	2.769499
seq.12569.25	12569-25	T-complex protein 1 subunit epsilon	CCT5	P48643	3.37E-05	1.88067
seq.12617.2	12617-2	Serine/threonine-protein kinase 24	STK24	Q9Y6E0	3.37E-05	1.038993
seq.13697.51	13697-51	Glycerol-3-phosphate dehydrogenase [NAD(+)], cytoplasmic	GPD1	P21695	3.37E-05	1.405293
seq.18408.26	18408-26	ADP-ribosylation factor 4	ARF4	P18085	3.37E-05	0.897602
seq.18916.25	18916-25	Inosine triphosphate pyrophosphatase	ITPA	Q9BY32	3.37E-05	1.063006
seq.19166.15	19166-15	40S ribosomal protein S19	RPS19	P39019	3.37E-05	0.934137
seq.21109.1	21109-1	Cilia- and flagella-associated protein 36	CFAP36	Q96G28	3.37E-05	1.256229
seq.25050.82	25050-82	Tripeptidyl-peptidase 2	TPP2	P29144	3.37E-05	0.64201
seq.3814.63	3814-63	Interleukin-11 receptor subunit alpha	IL11RA	Q14626	3.37E-05	-0.42215
seq.5312.49	5312-49	Apolipoprotein E (isoform E2)	APOE	P02649	3.37E-05	1.847578
seq.7871.16	7871-16	Transmembrane protein 132A	TMEM132A	Q24JP5	3.37E-05	1.40502
seq.10398.110	10398-110	Ankyrin repeat domain-containing protein 1	ANKRD1	Q15327	4.82E-05	1.519646
seq.11192.168	11192-168	Tubulointerstitial nephritis antigen-like	TINAGL1	Q9GZM7	4.82E-05	1.277504
seq.12694.28	12694-28	Probable dimethyladenosine transferase	DIMT1	Q9UNQ2	4.82E-05	0.7386
seq.12699.28	12699-28	T-complex protein 1 subunit theta	CCT8	P50990	4.82E-05	1.805417
seq.15326.64	15326-64	Guanylate-binding protein 1	GBP1	P32455	4.82E-05	1.764098
seq.17764.108	17764-108	Rho-related GTP-binding protein RhoC	RHOC	P08134	4.82E-05	1.057333
seq.18175.65	18175-65	Synaptic vesicle membrane protein VAT-1 homolog	VAT1	Q99536	4.82E-05	1.40828
seq.18317.111	18317-111	AN1-type zinc finger protein 5	ZFAND5	O76080	4.82E-05	0.981149
seq.19129.15	19129-15	Methenyltetrahydrofolate synthase domain-containing protein	MTHFSD	Q2M296	4.82E-05	0.355656
seq.19175.18	19175-18	MARCKS-related protein	MARCKSL1	P49006	4.82E-05	0.689814
seq.19563.3	19563-3	Seizure 6-like protein	SEZ6L	Q9BYH1	4.82E-05	-0.12065
seq.20943.14	20943-14	N-alpha-acetyltransferase 50	NAA50	Q9GZZ1	4.82E-05	1.391174
seq.23638.3	23638-3	Carnosine N-methyltransferase	CARNMT1	Q8N4J0	4.82E-05	0.793692
seq.23656.9	23656-9	Pseudouridylate synthase 7 homolog	PUS7	Q96PZ0	4.82E-05	0.753011
seq.23659.2	23659-2	Basic leucine zipper and W2 domain-containing protein 2	BZW2	Q9Y6E2	4.82E-05	1.388439
seq.2974.61	2974-61	Contactin-1	CNTN1	Q12860	4.82E-05	1.258116
seq.3858.5	3858-5	Low molecular weight phosphotyrosine protein phosphatase	ACP1	P24666	4.82E-05	0.748651
seq.6520.87	6520-87	Matrix Gla protein	MGP	P08493	4.82E-05	1.647601
seq.12621.55	12621-55	Serine/threonine-protein phosphatase 2A 65 kDa regulatory subunit A alpha isoform	PPP2R1A	P30153	7.37E-05	1.074909
seq.12643.4	12643-4	Beta-arrestin-1	ARRB1	P49407	7.37E-05	1.128238
seq.14273.19	14273-19	Prolyl endopeptidase	PREP	P48147	7.37E-05	1.476057
seq.20948.16	20948-16	U8 snoRNA-decapping enzyme	NUDT16	Q96DE0	7.37E-05	1.36772
seq.21147.9	21147-9	Fructosamine-3-kinase	FN3K	Q9H479	7.37E-05	1.291647
seq.24235.2	24235-2	EH domain-containing protein 1	EHD1	Q9H4M9	7.37E-05	1.160939
seq.25949.3	25949-3	1-phosphatidylinositol 4,5-bisphosphate phosphodiesterase delta-3	PLCD3	Q8N3E9	7.37E-05	0.53503
seq.4282.3	4282-3	GTP-binding nuclear protein Ran	RAN	P62826	7.37E-05	1.343056
seq.4450.26	4450-26	Heterogeneous nuclear ribonucleoprotein A/B	HNRNPAB	Q99729	7.37E-05	1.320488
seq.13597.20	13597-20	Ras-related protein Rab-31	RAB31	Q13636	0.000105	0.617134
seq.13636.20	13636-20	Nucleosome assembly protein 1-like 1	NAP1L1	P55209	0.000105	1.058932
seq.15418.25	15418-25	Mitogen-activated protein kinase 10	MAPK10	P53779	0.000105	1.06882
seq.16616.137	16616-137	Beta-enolase	ENO3	P13929	0.000105	1.181231
seq.17384.110	17384-110	ATP-dependent 6-phosphofructokinase, muscle type	PFKM	P08237	0.000105	1.139234
seq.17836.17	17836-17	Protein S100-A16	S100A16	Q96FQ6	0.000105	1.171812
seq.18214.2	18214-2	Glutamate--cysteine ligase regulatory subunit	GCLM	P48507	0.000105	0.594974
seq.21172.11	21172-11	Mesoderm development candidate 1	TLNRD1	Q9H1K6	0.000105	0.372591

Disease Models & Mechanisms • Supplementary information

seq.21929.53	21929-53	UB2D3/PolyUbiquitin K48	UBE2D3|UBB	P61077|P0C G47	0.000105	1.218588
seq.2567.5	2567-5	Complement factor I	CFI	P05156	0.000105	2.001857
seq.2811.27	2811-27	Angiopoietin-1	ANGPT1	Q15389	0.000105	-1.31186
seq.2889.37	2889-37	Cardiotrophin-1	CTF1	Q16619	0.000105	0.589272
seq.9482.110	9482-110	ADP-ribose pyrophosphatase, mitochondrial	NUDT9	Q9BW91	0.000105	1.206546
seq.9831.12	9831-12	Ras GTPase-activating protein-binding protein 2	G3BP2	Q9UN86	0.000105	1.258375
seq.9901.28	9901-28	Egl nine homolog 1	EGLN1	Q9GZT9	0.000105	0.94728
seq.10800.15	10800-15	Serpin H1	SERPINH1	P50454	0.000139	1.187959
seq.11487.4	11487-4	Testican-1	SPOCK1	Q08629	0.000139	-0.73833
seq.11830.48	11830-48	Tyrosine-protein phosphatase non-receptor type 11	PTPN11	Q06124	0.000139	1.399248
seq.12456.5	12456-5	6-phosphofructo-2-kinase/fructose-2,6-bisphosphatase 3	PFKFB3	Q16875	0.000139	0.627043
seq.13680.3	13680-3	NGFI-A-binding protein 2	NAB2	Q15742	0.000139	0.325996
seq.13937.75	13937-75	Cullin-associated NEDD8-dissociated protein 1	CAND1	Q86VP6	0.000139	1.005029
seq.16043.30	16043-30	SHC-transforming protein 1:Phosphotyrosine Interaction Domain	SHC1	P29353	0.000139	0.719777
seq.18184.28	18184-28	GMP synthase [glutamine-hydrolyzing]	GMPS	P49915	0.000139	1.359182
seq.21857.26	21857-26	Guanine nucleotide-binding protein subunit beta-2-like 1	RACK1	P63244	0.000139	1.368194
seq.23394.125	23394-125	Growth arrest-specific protein 2	GAS2	O43903	0.000139	1.266463
seq.23923.26	23923-26	Protein phosphatase 1F	PPM1F	P49593	0.000139	1.051595
seq.2855.49	2855-49	Mitogen-activated protein kinase 3	MAPK3	P27361	0.000139	1.062793
seq.2966.65	2966-65	Stem cell growth factor-beta	CLEC11A	Q9Y240	0.000139	0.476608
seq.2986.49	2986-49	Gro-gamma	CXCL3	P19876	0.000139	0.454881
seq.3169.70	3169-70	Alpha-L-iduronidase	IDUA	P35475	0.000139	1.160087
seq.3469.74	3469-74	Ribosomal protein S6 kinase alpha-3	RPS6KA3	P51812	0.000139	0.97024
seq.6556.5	6556-5	Ectonucleotide pyrophosphatase/phosphodiesterase family member 5	ENPP5	Q9UJA9	0.000139	0.641913
seq.11355.10	11355-10	Eukaryotic translation initiation factor 5A-2	EIF5A2	Q9GZV4	0.000185	0.740908
seq.11382.5	11382-5	Biliverdin reductase A	BLVRA	P53004	0.000185	0.833795
seq.12478.15	12478-15	60S ribosomal protein L30	RPL30	P62888	0.000185	1.176437
seq.12575.30	12575-30	C-1-tetrahydrofolate synthase, cytoplasmic	MTHFD1	P11586	0.000185	1.013173
seq.12869.68	12869-68	Probable ATP-dependent RNA helicase DDX6	DDX6	P26196	0.000185	1.206781
seq.13552.7	13552-7	Switch-associated protein 70	SWAP70	Q9UH65	0.000185	0.6286
seq.14131.37	14131-37	Ephrin-B2:Extracellular domain	EFNB2	P52799	0.000185	0.608966
seq.15318.75	15318-75	Protein S100-A10	S100A10	P60903	0.000185	0.584018
seq.17794.6	17794-6	Phosphomannomutase 2	PMM2	O15305	0.000185	1.37173
seq.17854.33	17854-33	N-alpha-acetyltransferase 10	NAA10	P41227	0.000185	0.681885
seq.18348.89	18348-89	Heterogeneous nuclear ribonucleoprotein D0	HNRNPD	Q14103	0.000185	1.278835
seq.21757.49	21757-49	Ubiquitin carboxyl-terminal hydrolase 22	USP22	Q9UPT9	0.000185	0.751155
seq.22047.46	22047-46	Collagen alpha-1(V) chain	COL5A1	P20908	0.000185	1.478881
seq.3170.6	3170-6	Methionine aminopeptidase 2	METAP2	P50579	0.000185	1.105159
seq.3441.64	3441-64	Glycogen synthase kinase-3 alpha	GSK3A	P49840	0.000185	0.751626
seq.4187.49	4187-49	6-phosphogluconate dehydrogenase, decarboxylating	PGD	P52209	0.000185	1.293583
seq.5888.29	5888-29	Eukaryotic translation initiation factor 5A-1	EIF5A	P63241	0.000185	0.843513
seq.8901.40	8901-40	14-3-3 protein gamma	YWHAG	P61981	0.000185	1.327432
seq.9870.17	9870-17	Tryptophan--tRNA ligase, cytoplasmic	WARS1	P23381	0.000185	1.260598
seq.11431.235	11431-235	ATP-dependent DNA helicase Q1	RECQL	P46063	0.000242	1.083986
seq.11448.34	11448-34	Galactokinase	GALK1	P51570	0.000242	0.750466
seq.13931.22	13931-22	26S proteasome non-ATPase regulatory subunit 9	PSMD9	O00233	0.000242	0.739817
seq.14124.6	14124-6	Ephrin-A2	EFNA2	O43921	0.000242	0.580628
seq.15316.262	15316-262	Thioredoxin-like protein 4B	TXNL4B	Q9NX01	0.000242	0.630367
seq.16885.49	16885-49	Microtubule-associated protein RP/EB family member 3	MAPRE3	Q9UPY8	0.000242	1.170883
seq.17332.3	17332-3	Poly(ADP-ribose) glycohydrolase ARH3	ADPRS	Q9NX46	0.000242	1.068176
seq.18186.15	18186-15	Threonine--tRNA ligase, cytoplasmic	TARS1	P26639	0.000242	1.121376
seq.19189.2	19189-2	Ubiquitin carboxyl-terminal hydrolase 14	USP14	P54578	0.000242	1.117234
seq.21739.7	21739-7	Bifunctional UDP-N-acetylglucosamine 2-epimerase/N-acetylmannosamine kinase	GNE	Q9Y223	0.000242	0.302431
seq.22041.26	22041-26	Dual specificity mitogen-activated protein kinase kinase 5	MAP2K5	Q13163	0.000242	1.445586
seq.22989.24	22989-24	Protein lin-7 homolog A	LIN7A	O14910	0.000242	1.124472
seq.23666.35	23666-35	Bifunctional purine biosynthesis protein PURH	ATIC	P31939	0.000242	1.040335
seq.23695.1	23695-1	Fibronectin type III and SPRY domain-containing protein 1	FSD1	Q9BTV5	0.000242	0.656016
seq.25126.19	25126-19	E3 ubiquitin-protein ligase HECW2	HECW2	Q9P2P5	0.000242	0.668925
seq.3845.51	3845-51	Dynein light chain roadblock-type 1	DYNLRB1	Q9NP97	0.000242	1.462944
seq.4240.31	4240-31	Pyruvate kinase PKM	PKM	P14618	0.000242	1.332089
seq.4985.11	4985-11	Fatty acid-binding protein, epidermal	FABP5	Q01469	0.000242	-0.0838
seq.12703.6	12703-6	Serine/threonine-protein kinase Nek7	NEK7	Q8TDX7	0.000297	1.509691
seq.13967.14	13967-14	Thioredoxin reductase 1, cytoplasmic	TXNRD1	Q16881	0.000297	0.823643
seq.15361.37	15361-37	Ankyrin repeat domain-containing protein 1	ANKRD1	Q15327	0.000297	0.641962
seq.15387.44	15387-44	Neuropilin-2	NRP2	O60462	0.000297	1.107427
seq.15486.126	15486-126	Amiloride-sensitive amine oxidase [copper-containing]	AOC1	P19801	0.000297	1.297481
seq.15566.10	15566-10	Calponin-1	CNN1	P51911	0.000297	1.363526
seq.17722.5	17722-5	Peptidyl-prolyl cis-trans isomerase FKBP4	FKBP4	Q02790	0.000297	1.066535
seq.18884.22	18884-22	DnaJ homolog subfamily B member 4	DNAJB4	Q9UDY4	0.000297	0.940167
seq.19188.21	19188-21	nucleosome assembly protein 1-like 4	NAP1L4	Q99733	0.000297	0.878263
seq.19297.4	19297-4	Glucose-6-phosphate 1-dehydrogenase	G6PD	P11413	0.000297	1.111619
seq.19523.215	19523-215	Protein DJ-1	PARK7	Q99497	0.000297	0.939862
seq.21173.25	21173-25	ELAV-like protein 2	ELAVL2	Q12926	0.000297	0.557726
seq.21221.67	21221-67	Serpin B9	SERPINB9	P50453	0.000297	1.114978
seq.21349.2	21349-2	Serine/threonine/tyrosine-interacting protein	STYX	Q8WUJ0	0.000297	0.34845
seq.23337.54	23337-54	BTB/POZ domain-containing adapter for CUL3-mediated RhoA degradation protein 3	KCTD10	Q9H3F6	0.000297	0.468484
seq.23389.28	23389-28	Fumarylacetoacetate hydrolase domain-containing protein 2A	FAHD2A	Q96GK7	0.000297	0.621183
seq.23678.132	23678-132	Transcription factor SOX-10	SOX10	P56693	0.000297	0.450557
seq.24436.23	24436-23	Disks large-associated protein 4	DLGAP4	Q9Y2H0	0.000297	1.034504
seq.24685.12	24685-12	Sorting nexin-11	SNX11	Q9Y5W9	0.000297	0.872738
seq.25913.17	25913-17	Ferritin heavy chain	FTH1	P02794	0.000297	0.496524
seq.2937.10	2937-10	Apolipoprotein E (isoform E3)	APOE	P02649	0.000297	1.696784

Disease Models & Mechanisms • Supplementary information

seq.4500.50	4500-50	Stem cell growth factor-alpha	CLEC11A	Q9Y240	0.000297	0.691133
seq.4903.72	4903-72	Calcineurin	PPP3CA|PPP3R1	Q08209|P63098	0.000297	1.057881
seq.5012.67	5012-67	Adenylate kinase isoenzyme 1	AK1	P00568	0.000297	0.990928
seq.5229.90	5229-90	Inosine-5'-monophosphate dehydrogenase 1	IMPDH1	P20839	0.000297	0.70841
seq.5660.51	5660-51	Extracellular superoxide dismutase [Cu-Zn]	SOD3	P08294	0.000297	2.594572
seq.6217.23	6217-23	Sulfhydryl oxidase 1	QSOX1	O00391	0.000297	1.044669
seq.6285.71	6285-71	Malectin	MLEC	Q14165	0.000297	0.965485
seq.8463.2	8463-2	Extracellular superoxide dismutase [Cu-Zn]	SOD3	P08294	0.000297	2.441591
seq.9744.139	9744-139	DnaJ homolog subfamily A member 4	DNAJA4	Q8WW22	0.000297	0.806319
seq.10627.87	10627-87	Amyloid-like protein 2	APLP2	Q06481	0.000336	0.969817
seq.10900.272	10900-272	Stathmin-2	STMN2	Q93045	0.000336	0.923087
seq.11103.24	11103-24	Heat shock protein beta-1	HSPB1	P04792	0.000336	1.014958
seq.11122.97	11122-97	Maspardin	SPG21	Q9NZD8	0.000336	0.450516
seq.12436.84	12436-84	Glutathione S-transferase omega-1	GSTO1	P78417	0.000336	0.763444
seq.12628.31	12628-31	LanC-like protein 2	LANCL2	Q9NS86	0.000336	1.145724
seq.12630.8	12630-8	Arfaptin-2	ARFIP2	P53365	0.000336	0.62867
seq.12688.115	12688-115	Ribosomal protein S6 kinase alpha-6	RPS6KA6	Q9UK32	0.000336	0.602035
seq.12707.26	12707-26	Dihydropyrimidinase-related protein 3	DPYSL3	Q14195	0.000336	1.044811
seq.12740.55	12740-55	Protein FEV	FEV	Q99581	0.000336	-0.14085
seq.13104.32	13104-32	Ephrin-B1	EFNB1	P98172	0.000336	0.30059
seq.15483.377	15483-377	Agrin	AGRN	O00468	0.000336	1.196991
seq.17364.8	17364-8	U2 small nuclear ribonucleoprotein B''	SNRPB2	P08579	0.000336	1.003519
seq.17686.27	17686-27	Tubulin-folding cofactor B	TBCB	Q99426	0.000336	1.309288
seq.18910.45	18910-45	Protein lin-7 homolog A	LIN7A	O14910	0.000336	1.045892
seq.19222.124	19222-124	Ras-related protein Rab-5B	RAB5B	P61020	0.000336	1.033088
seq.19265.9	19265-9	T-complex protein 1 subunit alpha	TCP1	P17987	0.000336	0.919954
seq.20130.144	20130-144	Casein kinase I isoform gamma-1	CSNK1G1	Q9HCP0	0.000336	0.832673
seq.21162.30	21162-30	Tubulin-specific chaperone cofactor E-like protein	TBCEL	Q5QJ74	0.000336	1.050697
seq.21163.21	21163-21	TBC1 domain family member 13	TBC1D13	Q9NVG8	0.000336	0.65089
seq.21676.17	21676-17	PAI-2	SERPINB2	P05120	0.000336	0.909225
seq.21862.145	21862-145	LIM and SH3 domain protein 1	LASP1	Q14847	0.000336	0.939332
seq.21985.61	21985-61	Integrin beta-1-binding protein 2	ITGB1BP2	Q9UKP3	0.000336	0.884481
seq.23290.3	23290-3	Survival motor neuron protein	SMN1	Q16637	0.000336	0.627541
seq.23302.19	23302-19	Proline-rich AKT1 substrate 1	AKT1S1	Q96B36	0.000336	0.5887
seq.24432.3	24432-3	GRIP1-associated protein 1	GRIPAP1	Q4V328	0.000336	0.478366
seq.24674.22	24674-22	Egl nine homolog 2	EGLN2	Q96KS0	0.000336	0.781791
seq.25256.23	25256-23	Elongation factor 1-alpha 1	EEF1A1	P68104	0.000336	1.081111
seq.25274.2	25274-2	Ankyrin repeat domain-containing protein 2	ANKRD2	Q9GZV1	0.000336	0.989091
seq.25465.42	25465-42	Muscular LMNA-interacting protein	MLIP	Q5VWP3	0.000336	1.058764
seq.25967.34	25967-34	Metalloproteinase inhibitor 1	TIMP1	P01033	0.000336	1.980864
seq.3234.23	3234-23	Coiled-coil domain-containing protein 80	CCDC80	Q76M96	0.000336	1.427258
seq.3379.29	3379-29	Protein kinase C iota type	PRKCI	P41743	0.000336	1.005225
seq.3887.90	3887-90	Importin subunit beta-1	KPNB1	Q14974	0.000336	0.983417
seq.4314.12	4314-12	dCTP pyrophosphatase 1	DCTPP1	Q9H773	0.000336	1.023951
seq.5020.50	5020-50	Phosphoglycerate kinase 1	PGK1	P00558	0.000336	0.950667
seq.6369.82	6369-82	Deformed epidermal autoregulatory factor 1 homolog	DEAF1	O75398	0.000336	0.324502
seq.6392.7	6392-7	WNT1-inducible-signaling pathway protein 2	CCN5	O76076	0.000336	1.736262
seq.6609.22	6609-22	2',3'-cyclic-nucleotide 3'-phosphodiesterase	CNP	P09543	0.000336	0.368323
seq.7070.25	7070-25	Kell blood group glycoprotein	KEL	P23276	0.000336	-0.14186
seq.7765.15	7765-15	Integral membrane protein 2A	ITM2A	O43736	0.000336	0.386054
seq.8019.73	8019-73	Stathmin-3	STMN3	Q9NZ72	0.000336	0.757682
seq.9065.28	9065-28	Alpha-taxilin	TXLNA	P40222	0.000336	0.773927
seq.9757.29	9757-29	Protein enabled homolog	ENAH	Q8N8S7	0.000336	0.812108
seq.9762.14	9762-14	Vasodilator-stimulated phosphoprotein	VASP	P50552	0.000336	0.90227
seq.9790.28	9790-28	Serine/threonine-protein kinase BRSK2	BRSK2	Q8IWQ3	0.000336	0.178224
seq.9969.8	9969-8	Solute carrier family 22 member 16	SLC22A16	Q86VW1	0.000336	0.487418
seq.10390.21	10390-21	E3 ubiquitin-protein ligase ZNRF3	ZNRF3	Q9ULT6	0.000404	0.617564
seq.10961.15	10961-15	Retinoic acid receptor responder protein 3	PLAAT4	Q9UL19	0.000404	0.28047
seq.11098.1	11098-1	Pyridoxal kinase	PDXK	O00764	0.000404	0.739755
seq.12670.15	12670-15	Cell cycle checkpoint protein RAD1	RAD1	O60671	0.000404	0.792804
seq.13580.2	13580-2	UDP-N-acetylhexosamine pyrophosphorylase	UAP1	Q16222	0.000404	0.365923
seq.13622.16	13622-16	Serine/threonine-protein phosphatase 2A 56 kDa regulatory subunit alpha isoform	PPP2R5A	Q15172	0.000404	1.00468
seq.14670.1	14670-1	Ski-like protein	SKIL	P12757	0.000404	0.906007
seq.15475.4	15475-4	Phospholipid transfer protein	PLTP	P55058	0.000404	1.800542
seq.15576.158	15576-158	Eosinophil cationic protein	RNASE3	P12724	0.000404	-0.09097
seq.16583.8	16583-8	Bis(5'-nucleosyl)-tetraphosphatase [asymmetrical]	NUDT2	P50583	0.000404	0.899872
seq.16599.38	16599-38	GPN-loop GTPase 1	GPN1	Q9HCN4	0.000404	0.994007
seq.17196.5	17196-5	ATP-dependent Clp protease proteolytic subunit, mitochondrial	CLPP	Q16740	0.000404	-0.0955
seq.17826.341	17826-341	Elongation factor 1-delta	EEF1D	P29692	0.000404	1.134111
seq.18299.13	18299-13	Calpain small subunit 1	CAPNS1	P04632	0.000404	0.712122
seq.18435.40	18435-40	UBX domain-containing protein 2B	UBXN2B	Q14CS0	0.000404	0.664734
seq.18877.15	18877-15	Calponin-2	CNN2	Q99439	0.000404	0.92974
seq.19154.41	19154-41	Glia-derived nexin	SERPINE2	P07093	0.000404	3.25517
seq.19278.19	19278-19	Ras-related protein Rab-1B	RAB1B	Q9H0U4	0.000404	0.848485
seq.20161.41	20161-41	Neural cell adhesion molecule 1	NCAM1	P13591	0.000404	0.788732
seq.21104.37	21104-37	Ubiquitin-like protein 5	UBL5	Q9BZL1	0.000404	0.99967
seq.21241.1	21241-1	N-acetyltransferase 6	NAA80	Q93015	0.000404	0.652551
seq.21383.37	21383-37	Asparaginyl-tRNA synthetase, cytoplasmic	NARS1	O43776	0.000404	1.126033
seq.21384.2	21384-2	Alpha-L-fucoside fucohydrolase	FUCA1	P04066	0.000404	0.758391
seq.23300.3	23300-3	RNA-binding protein with multiple splicing 2	RBPMS2	Q6ZRY4	0.000404	0.894841
seq.23329.52	23329-52	Ras-related protein Rab-4B	RAB4B	P61018	0.000404	1.048781
seq.23416.47	23416-47	Sorting nexin-15	SNX15	Q9NRS6	0.000404	0.992115
seq.23521.29	23521-29	Nucleoside diphosphate kinase 7	NME7	Q9Y5B8	0.000404	1.060125
seq.23566.6	23566-6	Apolipoprotein-L2	APOL2	Q9BQE5	0.000404	1.129743
seq.24215.8	24215-8	Paired mesoderm homeobox protein 1	PRRX1	P54821	0.000404	0.773122

seq.24260.4	24260-4	Histone-lysine N-methyltransferase setd3	SETD3	Q86TU7	0.000404	0.907297
seq.24425.8	24425-8	Methionyl-tRNA synthetase, cytoplasmic	MARS1	P56192	0.000404	1.14129
seq.25232.4	25232-4	EH domain-containing protein 3	EHD3	Q9NZN3	0.000404	0.49902
seq.3848.14	3848-14	Glyceraldehyde-3-phosphate dehydrogenase	GAPDH	P04406	0.000404	0.874105
seq.4374.45	4374-45	Growth/differentiation factor 15	GDF15	Q99988	0.000404	1.384075
seq.8007.19	8007-19	Cathepsin B	CTSB	P07858	0.000404	0.926046
seq.8039.41	8039-41	Protein FAM177A1	FAM177A1	Q8N128	0.000404	0.861632
seq.8480.29	8480-29	EGF-containing fibulin-like extracellular matrix protein 1	EFEMP1	Q12805	0.000404	0.663083
seq.8986.2	8986-2	D-3-phosphoglycerate dehydrogenase	PHGDH	O43175	0.000404	0.866339
seq.9211.19	9211-19	Pigment epithelium-derived factor	SERPINF1	P36955	0.000404	1.917588
seq.9244.27	9244-27	Palmitoyl-protein thioesterase 1	PPT1	P50897	0.000404	0.797837
seq.9760.13	9760-13	Mitogen-activated protein kinase 9	MAPK9	P45984	0.000404	0.926497
seq.15560.52	15560-52	Transcobalamin-2	TCN2	P20062	0.000413	-0.33619
seq.24236.46	24236-46	Synaptic vesicle membrane protein VAT-1 homolog-like	VAT1L	Q9HCJ6	0.000413	1.198889
seq.24474.12	24474-12	Tetratricopeptide repeat protein 9A	TTC9	Q92623	0.000413	1.197582
seq.3173.49	3173-49	N-acylethanolamine-hydrolyzing acid amidase	NAAA	Q02083	0.000413	0.65881
seq.9940.35	9940-35	Dual specificity phosphatase 28	DUSP28	Q4G0W2	0.000413	0.947241
seq.4543.65	4543-65	Collagen alpha-1(XXIII) chain	COL23A1	Q86Y22	0.000475	-0.36886
seq.9004.24	9004-24	Low-density lipoprotein receptor class A domain-containing protein 4	LDLRAD4	O15165	0.000475	0.774072
seq.10363.13	10363-13	Mothers against decapentaplegic homolog 3	SMAD3	P84022	0.000475	0.615717
seq.12498.12	12498-12	Tax1-binding protein 3	TAX1BP3	O14907	0.000475	0.9005
seq.12504.26	12504-26	Leiomodin-1	LMOD1	P29536	0.000475	0.60057
seq.12656.1	12656-1	Kinesin light chain 1	KLC1	Q07866	0.000475	0.504069
seq.13576.15	13576-15	Glutathione S-transferase P	GSTP1	P09211	0.000475	0.508565
seq.13602.6	13602-6	NHP2-like protein 1	SNU13	P55769	0.000475	0.874998
seq.13954.9	13954-9	Glucosamine 6-phosphate N-acetyltransferase	GNPNAT1	Q96EK6	0.000475	0.687969
seq.14029.42	14029-42	COP9 signalosome complex subunit 2	COPS2	P61201	0.000475	0.485523
seq.14094.29	14094-29	Heparin-binding EGF-like growth factor	HBEGF	Q99075	0.000475	2.368799
seq.16057.6	16057-6	Cation-independent mannose-6-phosphate receptor	IGF2R	P11717	0.000475	0.403149
seq.17156.72	17156-72	Serine/threonine-protein kinase DCLK1	DCLK1	O15075	0.000475	0.604953
seq.17852.5	17852-5	Protein phosphatase methylesterase 1	PPME1	Q9Y570	0.000475	0.934855
seq.18257.64	18257-64	Signal recognition particle 19 kDa protein	SRP19	P09132	0.000475	0.337098
seq.18301.10	18301-10	Nuclear apoptosis-inducing factor 1	NAIF1	Q69YI7	0.000475	0.941856
seq.18342.2	18342-2	Phosphoserine aminotransferase	PSAT1	Q9Y617	0.000475	1.022751
seq.18422.41	18422-41	Diphosphomevalonate decarboxylase	MVD	P53602	0.000475	0.511962
seq.19196.73	19196-73	Homeodomain-only protein	HOPX	Q9BPY8	0.000475	0.707813
seq.21105.23	21105-23	Protein unc-119 homolog B	UNC119B	A6NIH7	0.000475	0.547695
seq.21583.14	21583-14	60S ribosomal protein L26-like 1	RPL26L1	Q9UNX3	0.000475	1.038205
seq.21600.10	21600-10	Beta-catenin-interacting protein 1	CTNNBIP1	Q9NSA3	0.000475	0.872714
seq.21727.15	21727-15	Ubiquitin-conjugating enzyme E2 Q1	UBE2Q1	Q7Z7E8	0.000475	0.289013
seq.22009.1	22009-1	Protein phosphatase 1B	PPM1B	O75688	0.000475	0.835143
seq.23173.3	23173-3	Metalloproteinase inhibitor 1	TIMP1	P01033	0.000475	2.045136
seq.23298.148	23298-148	Acyl-coenzyme A thioesterase THEM4	THEM4	Q5T1C6	0.000475	0.254049
seq.23318.60	23318-60	Phosphatidylcholine transfer protein	PCTP	Q9UKL6	0.000475	0.33309
seq.23542.8	23542-8	Golgi phosphoprotein 3-like	GOLPH3L	Q9H4A5	0.000475	0.85334
seq.23669.20	23669-20	Glutamine--fructose-6-phosphate aminotransferase [isomerizing] 2	GFPT2	O94808	0.000475	0.306
seq.24111.10	24111-10	Elongation factor 1-gamma	EEF1G	P26641	0.000475	0.914911
seq.24268.21	24268-21	GRB2-associated-binding protein 1	GAB1	Q13480	0.000475	0.974253
seq.24459.15	24459-15	Coatomer subunit beta'	COPB2	P35606	0.000475	1.072314
seq.25060.18	25060-18	NADP-dependent malic enzyme	ME1	P48163	0.000475	0.737549
seq.25083.26	25083-26	Protein diaphanous homolog 1	DIAPH1	O60610	0.000475	0.584046
seq.25094.9	25094-9	Acetyl-coenzyme A synthetase, cytoplasmic	ACSS2	Q9NR19	0.000475	0.432352
seq.3366.51	3366-51	Extracellular matrix protein 1	ECM1	Q16610	0.000475	2.201907
seq.4179.57	4179-57	14-3-3 protein gamma	YWHAG	P61981	0.000475	1.094956
seq.4258.15	4258-15	Proliferation-associated protein 2G4	PA2G4	Q9UQ80	0.000475	1.190368
seq.4460.8	4460-8	3-phosphoinositide-dependent protein kinase 1	PDPK1	O15530	0.000475	0.3607
seq.5021.13	5021-13	Inorganic pyrophosphatase	PPA1	Q15181	0.000475	0.95261
seq.6372.7	6372-7	Y-box-binding protein 2	YBX2	Q9Y2T7	0.000475	0.839139
seq.9171.11	9171-11	Cysteine and glycine-rich protein 3	CSRP3	P50461	0.000475	1.775067
seq.9751.72	9751-72	Nuclease-sensitive element-binding protein 1	YBX1	P67809	0.000475	0.807036
seq.9842.2	9842-2	Catenin beta-1	CTNNB1	P35222	0.000475	0.63952
seq.6400.33	6400-33	Tumor protein p53-inducible protein 13	TP53I13	Q8NBR0	0.000535	0.923516
seq.12701.1	12701-1	Eukaryotic translation initiation factor 1b	EIF1B	O60739	0.000565	1.282307
seq.10818.36	10818-36	Sphingomyelin phosphodiesterase	SMPD1	P17405	0.000565	0.526429
seq.12340.17	12340-17	Alanine--tRNA ligase, cytoplasmic	AARS1	P49588	0.000565	0.791422
seq.12632.14	12632-14	Arylamine N-acetyltransferase 1	NAT1	P18440	0.000565	0.408026
seq.12724.81	12724-81	Cold-inducible RNA-binding protein	CIRBP	Q14011	0.000565	1.057148
seq.12747.89	12747-89	RNA-binding protein 3	RBM3	P98179	0.000565	1.084064
seq.12956.40	12956-40	KIF1-binding protein	KIFBP	Q96EK5	0.000565	0.965247
seq.13374.4	13374-4	Beta-defensin 113	DEFB113	Q30KQ7	0.000565	0.450637
seq.13986.6	13986-6	LanC-like protein 1	LANCL1	O43813	0.000565	0.933912
seq.15627.83	15627-83	RAC-alpha serine/threonine-protein kinase	AKT1	P31749	0.000565	0.985313
seq.17367.5	17367-5	Stathmin	STMN1	P16949	0.000565	0.870541
seq.17676.13	17676-13	Cdc42-interacting protein 4	TRIP10	Q15642	0.000565	0.833922
seq.17802.4	17802-4	Sialic acid synthase	NANS	Q9NR45	0.000565	0.964542
seq.18174.79	18174-79	Programmed cell death 6-interacting protein	PDCD6IP	Q8WUM4	0.000565	0.955862
seq.18224.11	18224-11	Charged multivesicular body protein 1a	CHMP1A	Q9HD42	0.000565	0.973167
seq.18322.15	18322-15	Bifunctional arginine demethylase and lysyl-hydroxylase JMJD6	JMJD6	Q6NYC1	0.000565	0.352901
seq.18880.81	18880-81	Collagen Type III	COL3A1	P02461	0.000565	1.813412
seq.19161.1	19161-1	Ubiquitin carboxyl-terminal hydrolase 15	USP15	Q9Y4E8	0.000565	0.849944
seq.19173.5	19173-5	AN1-type zinc finger protein 1	ZFAND1	Q8TCF1	0.000565	0.885261
seq.20221.26	20221-26	cAMP-specific 3',5'-cyclic phosphodiesterase 4C	PDE4C	Q08493	0.000565	0.959698
seq.20525.200	20525-200	Protein disulfide-isomerase A4	PDIA4	P13667	0.000565	1.425709
seq.20952.15	20952-15	Ethylmalonyl-CoA decarboxylase	ECHDC1	Q9NTX5	0.000565	0.929315

Disease Models & Mechanisms • Supplementary information

seq.20964.13	20964-13	40S ribosomal protein S14	RPS14	P62263	0.000565	0.238727
seq.21002.1	21002-1	NudC domain-containing protein 2	NUDCD2	Q8WVJ2	0.000565	0.794415
seq.21445.40	21445-40	Ubiquitin-like protein Nedd8	NEDD8	Q15843	0.000565	1.136996
seq.21885.196	21885-196	Nucleolysin TIA-1 isoform p40	TIA1	P31483	0.000565	0.868801
seq.21926.24	21926-24	UB2D1/PolyUbiquitin K48	UBE2D1|UBB	P51668|P0C G47	0.000565	0.788513
seq.22381.1	22381-1	Endoribonuclease LACTB2	LACTB2	Q53H82	0.000565	0.906345
seq.22485.1	22485-1	BTB/POZ domain-containing protein KCTD7	KCTD7	Q96MP8	0.000565	0.320969
seq.23265.15	23265-15	Tumor protein D54	TPD52L2	O43399	0.000565	0.998059
seq.23363.41	23363-41	Poly(rC)-binding protein 2	PCBP2	Q15366	0.000565	0.813419
seq.23535.3	23535-3	Protein FAM45A	DENND10	Q8TCE6	0.000565	0.73521
seq.23705.42	23705-42	CTP synthase 1	CTPS1	P17812	0.000565	0.869483
seq.24408.30	24408-30	Muscleblind-like protein 1	MBNL1	Q9NR56	0.000565	0.789198
seq.25033.194	25033-194	Transitional endoplasmic reticulum ATPase	VCP	P55072	0.000565	0.947978
seq.25105.87	25105-87	Eukaryotic translation initiation factor 2A	EIF2A	Q9BY44	0.000565	0.940622
seq.25468.5	25468-5	SOX-9	SOX9	P48436	0.000565	0.353916
seq.25951.17	25951-17	Preferentially expressed antigen of melanoma	PRAME	P78395	0.000565	0.247328
seq.3283.21	3283-21	Transforming growth factor-beta-induced protein ig-h3	TGFBI	Q15582	0.000565	2.844687
seq.3868.8	3868-8	Small glutamine-rich tetratricopeptide repeat-containing protein alpha	SGTA	O43765	0.000565	0.920841
seq.3903.49	3903-49	Sorting nexin-4	SNX4	O95219	0.000565	0.535147
seq.4292.5	4292-5	Alpha-soluble NSF attachment protein	NAPA	P54920	0.000565	0.846361
seq.4924.32	4924-32	Interstitial collagenase	MMP1	P03956	0.000565	1.828061
seq.4962.52	4962-52	Cerebral dopamine neurotrophic factor	CDNF	Q49AH0	0.000565	-0.15398
seq.5918.5	5918-5	Proteasome activator complex subunit 1	PSME1	Q06323	0.000565	0.749599
seq.7841.84	7841-84	Endothelial cell-selective adhesion molecule	ESAM	Q96AP7	0.000565	-0.10646
seq.8894.80	8894-80	Heterogeneous nuclear ribonucleoprotein A/B	HNRNPAB	Q99729	0.000565	0.610479
seq.8942.2	8942-2	39S ribosomal protein L21, mitochondrial	MRPL21	Q7Z2W9	0.000565	0.529524
seq.9053.16	9053-16	Cysteine-rich protein 2	CRIP2	P52943	0.000565	0.874502
seq.9942.2	9942-2	NTF2-related export protein 1	NXT1	Q9UKK6	0.000565	0.670859
seq.6919.3	6919-3	Hemoglobin subunit zeta	HBZ	P02008	0.000623	0.200797
seq.20568.3	20568-3	FRAS1-related extracellular matrix protein 1	FREM1	Q5H8C1	0.000674	-0.69172
seq.24981.8	24981-8	Cytoplasmic dynein 1 light intermediate chain 1	DYNC1LI1	Q9Y6G9	0.000674	0.747308
seq.3174.2	3174-2	A disintegrin and metalloproteinase with thrombospondin motifs 1	ADAMTS1	Q9UHI8	0.000674	-0.16048
seq.10346.5	10346-5	Signal transducer and activator of transcription 3	STAT3	P40763	0.000674	1.077108
seq.10354.57	10354-57	Signal transducer and activator of transcription 3	STAT3	P40763	0.000674	0.889971
seq.10447.18	10447-18	Polyadenylate-binding protein 3	PABPC3	Q9H361	0.000674	0.703907
seq.10866.60	10866-60	Serine/threonine-protein phosphatase 4 regulatory subunit 3A	PPP4R3A	Q6IN85	0.000674	0.882045
seq.11307.33	11307-33	NEDD4-like E3 ubiquitin-protein ligase WWP1	WWP1	Q9H0M0	0.000674	0.885113
seq.11438.6	11438-6	DnaJ homolog subfamily B member 2	DNAJB2	P25686	0.000674	0.382868
seq.11458.30	11458-30	Poly(rC)-binding protein 1	PCBP1	Q15365	0.000674	0.946411
seq.12448.246	12448-246	Glycylpeptide N-tetradecanoyltransferase 1	NMT1	P30419	0.000674	0.967414
seq.12499.108	12499-108	Endophilin-A1	SH3GL2	Q99962	0.000674	0.924095
seq.12854.3	12854-3	Transcriptional enhancer factor TEF-5	TEAD3	Q99594	0.000674	0.748005
seq.13125.45	13125-45	Vitronectin	VTN	P04004	0.000674	0.499504
seq.13566.2	13566-2	RNA polymerase II subunit A C-terminal domain phosphatase SSU72	SSU72	Q9NP77	0.000674	0.604104
seq.13573.5	13573-5	Actin-related protein 2/3 complex subunit 3	ARPC3	O15145	0.000674	0.283156
seq.13933.276	13933-276	NGFI-A-binding protein 1	NAB1	Q13506	0.000674	0.453962
seq.13963.7	13963-7	Toll-interacting protein	TOLLIP	Q9H0E2	0.000674	0.755982
seq.14022.17	14022-17	Interleukin-17B	IL17B	Q9UHF5	0.000674	1.256105
seq.14287.6	14287-6	Ras-related protein Rab-5C	RAB5C	P51148	0.000674	0.628843
seq.15617.8	15617-8	Cytokine SCM-1 beta	XCL2	Q9UBD3	0.000674	0.407325
seq.15669.7	15669-7	Serine/threonine-protein kinase B-raf	BRAF	P15056	0.000674	0.397636
seq.17337.1	17337-1	Transcriptional repressor protein YY1	YY1	P25490	0.000674	0.983348
seq.17849.6	17849-6	Developmentally-regulated GTP-binding protein 1	DRG1	Q9Y295	0.000674	0.545747
seq.18172.71	18172-71	Histone chaperone ASF1A	ASF1A	Q9Y294	0.000674	0.30287
seq.18833.76	18833-76	Astrocytic phosphoprotein PEA-15	PEA15	Q15121	0.000674	0.835374
seq.19219.71	19219-71	Nuclear transport factor 2	NUTF2	P61970	0.000674	0.488748
seq.19240.265	19240-265	28 kDa heat- and acid-stable phosphoprotein	PDAP1	Q13442	0.000674	0.379179
seq.19797.4	19797-4	Histone acetyltransferase KAT2A	KAT2A	Q92830	0.000674	0.628382
seq.20932.10	20932-10	Coiled-coil domain-containing protein 69	CCDC69	A6NI79	0.000674	0.546194
seq.21129.95	21129-95	MAD2L1-binding protein	MAD2L1BP	Q15013	0.000674	0.457215
seq.21380.77	21380-77	Queuine tRNA-ribosyltransferase	QTRT1	Q9BXR0	0.000674	0.396951
seq.21387.64	21387-64	Meprin A subunit alpha	MEP1A	Q16819	0.000674	0.980111
seq.21911.17	21911-17	Norrin	NDP	Q00604	0.000674	0.703205
seq.22527.4	22527-4	PDZ and LIM domain protein 3	PDLIM3	Q53GG5	0.000674	2.368873
seq.23606.18	23606-18	Sterile alpha motif domain-containing protein 4B	SAMD4B	Q5PRF9	0.000674	0.929983
seq.25256.153	25256-153	Elongation factor 1-alpha 1	EEF1A1	P68104	0.000674	0.966564
seq.25285.14	25285-14	Kelch repeat and BTB domain-containing protein 11	KBTBD11	O94819	0.000674	0.828708
seq.25444.29	25444-29	Type I inositol 1,4,5-trisphosphate 5-phosphatase	INPP5A	Q14642	0.000674	0.914448
seq.2668.70	2668-70	Calpain I	CAPN1|CAPNS1	P07384|P04 632	0.000674	0.487427
seq.3210.1	3210-1	Methionine aminopeptidase 1	METAP1	P53582	0.000674	1.061751
seq.4209.60	4209-60	Vacuolar protein sorting-associated protein VTA1 homolog	VTA1	Q9NP79	0.000674	0.778075
seq.4965.27	4965-27	ATP synthase subunit beta, mitochondrial	ATP5F1B	P06576	0.000674	0.250101
seq.5124.62	5124-62	Intercellular adhesion molecule 5	ICAM5	Q9UMF0	0.000674	0.405061
seq.5930.54	5930-54	Troponin I, cardiac muscle	TNNI3	P19429	0.000674	1.774282
seq.6304.8	6304-8	Complement C1q tumor necrosis factor-related protein 1	C1QTNF1	Q9BXJ1	0.000674	0.367521
seq.6923.1	6923-1	Procollagen-lysine,2-oxoglutarate 5-dioxygenase 2	PLOD2	O00469	0.000674	0.865139
seq.7886.26	7886-26	Serine palmitoyltransferase 1	SPTLC1	O15269	0.000674	0.272853
seq.8307.47	8307-47	UPF0696 protein C11orf68	C11orf68	Q9H3H3	0.000674	0.766274
seq.9883.29	9883-29	Lactoylglutathione lyase	GLO1	Q04760	0.000674	0.917387
seq.23528.199	23528-199	Phytanoyl-CoA hydroxylase-interacting protein-like	PHYHIPL	Q96FC7	0.000732	0.610568
seq.23700.42	23700-42	ATP-binding cassette sub-family F member 3	ABCF3	Q9NUQ8	0.000732	0.76813
seq.4232.19	4232-19	Insulin-like growth factor 1 receptor	IGF1R	P08069	0.000732	0.489713

Disease Models & Mechanisms • Supplementary information

seq.4254.6	4254-6	NudC domain-containing protein 3	NUDCD3	Q8IVD9	0.000732	-0.18196
seq.21357.12	21357-12	Filamin-binding LIM protein 1	FBLIM1	Q8WUP2	0.000804	0.676637
seq.17199.43	17199-43	Hypoxia-inducible factor 1-alpha inhibitor	HIF1AN	Q9NWT6	0.000804	0.17361
seq.24945.16	24945-16	Sorting nexin-27	SNX27	Q96L92	0.000804	0.680191
seq.10012.5	10012-5	SAM pointed domain-containing Ets transcription factor	SPDEF	O95238	0.000804	0.14867
seq.10080.9	10080-9	Translation initiation factor eIF-2B subunit alpha	EIF2B1	Q14232	0.000804	0.918311
seq.10336.3	10336-3	E3 ubiquitin-protein ligase CHIP	STUB1	Q9UNE7	0.000804	1.072141
seq.11572.4	11572-4	Dynamin-2	DNM2	P50570	0.000804	0.448058
seq.12332.7	12332-7	Eukaryotic elongation factor 2 kinase	EEF2K	O00418	0.000804	0.233499
seq.12438.127	12438-127	DNA-3-methyladenine glycosylase	MPG	P29372	0.000804	0.467417
seq.12454.105	12454-105	N-terminal Xaa-Pro-Lys N-methyltransferase 1	NTMT1	Q9BV86	0.000804	0.459544
seq.12491.23	12491-23	Chloride intracellular channel protein 4	CLIC4	Q9Y696	0.000804	0.85269
seq.13700.10	13700-10	Annexin A2	ANXA2	P07355	0.000804	0.845873
seq.13744.37	13744-37	Interleukin-3 receptor subunit alpha	IL3RA	P26951	0.000804	-0.10666
seq.14098.28	14098-28	Cysteine--tRNA ligase, cytoplasmic	CARS1	P49589	0.000804	0.726857
seq.15312.14	15312-14	Protein argonaute-1	AGO1	Q9UL18	0.000804	0.494977
seq.15474.7	15474-7	Peptidyl-prolyl cis-trans isomerase NIMA-interacting 1	PIN1	Q13526	0.000804	0.672003
seq.15526.33	15526-33	Glutathione synthetase	GSS	P48637	0.000804	0.737897
seq.15693.9	15693-9	Progesterone receptor	PGR	P06401	0.000804	0.824152
seq.17205.21	17205-21	Ras-related protein Rab-5A	RAB5A	P20339	0.000804	0.833516
seq.17347.80	17347-80	Axin interactor, dorsalization-associated protein	AIDA	Q96BJ3	0.000804	0.952998
seq.17777.31	17777-31	Serine dehydratase-like	SDSL	Q96GA7	0.000804	0.28431
seq.18318.98	18318-98	Endophilin-A3	SH3GL3	Q99963	0.000804	0.888466
seq.18336.31	18336-31	Cytidine deaminase	CDA	P32320	0.000804	0.203822
seq.18931.40	18931-40	Slit homolog 3 protein	SLIT3	O75094	0.000804	0.746969
seq.19187.21	19187-21	STAM-binding protein	STAMBP	O95630	0.000804	0.204362
seq.19223.6	19223-6	Ras-related protein Rab-1A	RAB1A	P62820	0.000804	0.690062
seq.19224.5	19224-5	Eukaryotic translation initiation factor 1A, Y-chromosomal	EIF1AY	O14602	0.000804	0.967984
seq.20982.29	20982-29	U11/U12 small nuclear ribonucleoprotein 25 kDa protein	SNRNP25	Q9BV90	0.000804	0.44658
seq.21579.35	21579-35	Myosin-binding protein C, cardiac-type	MYBPC3	Q14896	0.000804	1.27544
seq.21827.7	21827-7	Rho-related GTP-binding protein RhoQ	RHOQ	P17081	0.000804	0.66128
seq.22984.10	22984-10	Eukaryotic initiation factor 4A-II	EIF4A2	Q14240	0.000804	0.853234
seq.22992.6	22992-6	Microtubule-associated protein RP/EB family member 3	MAPRE3	Q9UPY8	0.000804	0.782544
seq.23296.29	23296-29	SARP-3	SFRP5	Q5T4F7	0.000804	1.088694
seq.23312.16	23312-16	Rab-like protein 3	RABL3	Q5HYI8	0.000804	0.749855
seq.23398.1	23398-1	DNA-directed RNA polymerase II subunit GRINL1A	POLR2M	P0CAP2	0.000804	0.759721
seq.23401.3	23401-3	DAZ-associated protein 1	DAZAP1	Q96EP5	0.000804	0.970757
seq.23915.18	23915-18	Ras-related protein Rab-7	RAB7A	P51149	0.000804	0.929728
seq.24226.30	24226-30	Fascin	FSCN1	Q16658	0.000804	1.230399
seq.24446.65	24446-65	Muscleblind-like protein 2	MBNL2	Q5VZF2	0.000804	0.761578
seq.24717.26	24717-26	Serine/threonine-protein phosphatase 2A regulatory subunit B'	PTPA	Q15257	0.000804	0.854448
seq.24934.96	24934-96	Opioid growth factor receptor	OGFR	Q9NZT2	0.000804	0.957162
seq.3419.49	3419-49	Calcium/calmodulin-dependent protein kinase type II subunit delta	CAMK2D	Q13557	0.000804	0.769743
seq.3480.7	3480-7	Dual specificity protein phosphatase 3	DUSP3	P51452	0.000804	0.599917
seq.4459.68	4459-68	Proprotein convertase subtilisin/kexin type 7	PCSK7	Q16549	0.000804	-0.1296
seq.5139.32	5139-32	Netrin receptor UNC5C	UNC5C	O95185	0.000804	-1.1892
seq.5364.7	5364-7	Protein SET	SET	Q01105	0.000804	0.698942
seq.5460.60	5460-60	ATP-dependent RNA helicase DDX19B	DDX19B	Q9UMR2	0.000804	0.657465
seq.6283.60	6283-60	Mast cell-expressed membrane protein 1	MCEMP1	Q8IX19	0.000804	0.600267
seq.7735.17	7735-17	Pigment epithelium-derived factor	SERPINF1	P36955	0.000804	0.929149
seq.8921.139	8921-139	Polyadenylate-binding protein 2	PABPN1	Q86U42	0.000804	0.969849
seq.20387.277	20387-277	Coiled-coil-helix-coiled-coil-helix domain-containing protein 7	CHCHD7	Q9BUK0	0.000876	0.586693
seq.12697.30	12697-30	Phosphatidylinositol 5-phosphate 4-kinase type-2 alpha	PIP4K2A	P48426	0.000958	0.438988
seq.3038.9	3038-9	C-X-C motif chemokine 11	CXCL11	O14625	0.000958	0.509743
seq.16293.1	16293-1	Somatoliberin	GHRH	P01286	0.000958	0.349577
seq.18215.5	18215-5	Probable tRNA(His) guanylyltransferase	THG1L	Q9NWX6	0.000958	0.553224
seq.8035.6	8035-6	Uncharacterized protein C1orf198	C1orf198	Q9H425	0.000963	0.279879
seq.12338.27	12338-27	Pikachurin	EGFLAM	Q63HQ2	0.000963	0.182918
seq.12591.27	12591-27	Oligophrenin-1	OPHN1	O60890	0.000963	0.374182
seq.12599.10	12599-10	Fermitin family homolog 3	FERMT3	Q86UX7	0.000963	0.404011
seq.12706.2	12706-2	Serine/threonine-protein kinase MRCK alpha	CDC42BPA	Q5VT25	0.000963	0.474524
seq.13022.20	13022-20	Tumor protein p53-inducible protein 11	TP53I11	O14683	0.000963	0.461931
seq.13567.1	13567-1	Dihydropyrimidinase-related protein 2	DPYSL2	Q16555	0.000963	0.609191
seq.13610.9	13610-9	Melanoma-associated antigen 10	MAGEA10	P43363	0.000963	0.669672
seq.13743.56	13743-56	Cullin-4B	CUL4B	Q13620	0.000963	0.360327
seq.15323.112	15323-112	Protein argonaute-3	AGO3	Q9H9G7	0.000963	0.558301
seq.15465.79	15465-79	Protein canopy homolog 4	CNPY4	Q8N129	0.000963	0.697182
seq.15612.5	15612-5	Protein lin-7 homolog B	LIN7B	Q9HAP6	0.000963	0.923893
seq.17148.7	17148-7	Flavin reductase (NADPH)	BLVRB	P30043	0.000963	1.060019
seq.17331.138	17331-138	Kremen protein 1	KREMEN1	Q96MU8	0.000963	0.46714
seq.17694.32	17694-32	Proteasome activator complex subunit 2	PSME2	Q9UL46	0.000963	0.784131
seq.17757.86	17757-86	Calcium-binding protein 39	CAB39	Q9Y376	0.000963	1.001407
seq.17804.102	17804-102	Pyridoxine-5'-phosphate oxidase	PNPO	Q9NVS9	0.000963	0.324859
seq.18207.6	18207-6	Charged multivesicular body protein 1b	CHMP1B	Q7LBR1	0.000963	0.637909
seq.19259.176	19259-176	Eukaryotic translation initiation factor 2 subunit 1	EIF2S1	P05198	0.000963	0.886895
seq.19296.51	19296-51	Myosin regulatory light chain 2, atrial isoform	MYL7	Q01449	0.000963	1.081691
seq.20999.12	20999-12	60S ribosomal protein L5	RPL5	P46777	0.000963	1.153508
seq.21141.9	21141-9	Protein FAM84B	LRATD2	Q96KN1	0.000963	1.118592
seq.21281.13	21281-13	Calcium/calmodulin-dependent protein kinase II inhibitor 2	CAMK2N2	Q96S95	0.000963	0.508118
seq.21590.9	21590-9	Ankyrin repeat family A protein 2	ANKRA2	Q9H9E1	0.000963	0.285825
seq.21595.8	21595-8	Coiled-coil domain-containing protein 43	CCDC43	Q96MW1	0.000963	0.589437
seq.21829.8	21829-8	40S ribosomal protein S12	RPS12	P25398	0.000963	0.731077
seq.21913.5	21913-5	Transcription factor YY2	YY2	O15391	0.000963	0.888572
seq.21939.6	21939-6	ULA1/UBA3	NAE1|UBA3	Q13564|Q8TBC4	0.000963	0.76855
seq.21963.48	21963-48	Disabled homolog 2	DAB2	P98082	0.000963	0.600022

Disease Models & Mechanisms • Supplementary information

seq.2212.69	2212-69	Tissue-type plasminogen activator	PLAT	P00750	0.000963	0.46483
seq.22824.53	22824-53	V-type proton ATPase subunit C 2	ATP6V1C2	Q8NEY4	0.000963	0.926528
seq.23426.1	23426-1	Variable charge X-linked protein 1	VCX	Q9H320	0.000963	0.258778
seq.23663.28	23663-28	Vacuolar ATP synthase subunit C 1	ATP6V1C1	P21283	0.000963	0.556396
seq.24417.22	24417-22	Rab GTPase-binding effector protein 1	RABEP1	Q15276	0.000963	0.615535
seq.24637.3	24637-3	Transcription elongation factor A protein-like 5	TCEAL5	Q5H9L2	0.000963	0.93952
seq.24670.1	24670-1	PIH1 domain-containing protein 1	PIH1D1	Q9NWS0	0.000963	0.56065
seq.24676.105	24676-105	Uncharacterized protein C21orf59	CFAP298	P57076	0.000963	0.425598
seq.25108.6	25108-6	Arginyl-tRNA--protein transferase 1	ATE1	O95260	0.000963	0.648566
seq.3061.61	3061-61	Cathepsin B	CTSB	P07858	0.000963	0.76774
seq.3397.7	3397-7	Tyrosine-protein phosphatase non-receptor type 11	PTPN11	Q06124	0.000963	1.203013
seq.3485.28	3485-28	Beta-2-microglobulin	B2M	P61769	0.000963	0.957103
seq.4230.1	4230-1	Eukaryotic translation initiation factor 4 gamma 2	EIF4G2	P78344	0.000963	0.790055
seq.4961.17	4961-17	Annexin A2	ANXA2	P07355	0.000963	0.737812
seq.5441.67	5441-67	Troponin I, cardiac muscle	TNNI3	P19429	0.000963	1.725636
seq.6966.144	6966-144	Syntaxin-1B	STX1B	P61266	0.000963	-0.08988
seq.9855.10	9855-10	Transforming protein RhoA	RHOA	P61586	0.000963	0.760527
seq.9875.107	9875-107	THO complex subunit 1	THOC1	Q96FV9	0.000963	0.201842
seq.22148.135	22148-135	Forkhead box protein P3	FOXP3	Q9BZS1	0.001049	0.40934
seq.3044.3	3044-3	C-C motif chemokine 18	CCL18	P55774	0.001049	-0.1187
seq.4141.79	4141-79	C-X-C motif chemokine 10	CXCL10	P02778	0.001049	-0.11662
seq.8479.4	8479-4	Stromelysin-2	MMP10	P09238	0.001049	0.963777
seq.3189.61	3189-61	Enteropeptidase	TMPRSS15	P98073	0.001133	-0.13213
seq.10977.55	10977-55	Unique cartilage matrix-associated protein	UCMA	Q8WVF2	0.001133	0.157109
seq.21156.5	21156-5	Putative glutathione-specific gamma-glutamylcyclotransferase 2	CHAC2	Q8WUX2	0.001133	0.23102
seq.10053.5	10053-5	Integrin-linked protein kinase	ILK	Q13418	0.001133	0.443553
seq.11608.5	11608-5	Microtubule-associated proteins 1A/1B light chain 3B	MAP1LC3B	Q9GZQ8	0.001133	0.981878
seq.12583.77	12583-77	Serine/threonine-protein kinase A-Raf	ARAF	P10398	0.001133	0.379398
seq.13639.101	13639-101	General vesicular transport factor p115	USO1	O60763	0.001133	0.274867
seq.13659.36	13659-36	Manganese-transporting ATPase 13A1	ATP13A1	Q9HD20	0.001133	0.65646
seq.13673.21	13673-21	T-complex protein 1 subunit eta	CCT7	Q99832	0.001133	1.242381
seq.15427.35	15427-35	Lysyl oxidase homolog 3	LOXL3	P58215	0.001133	1.221498
seq.15441.6	15441-6	Ganglioside GM2 activator	GM2A	P17900	0.001133	0.526032
seq.15447.45	15447-45	Sorbitol dehydrogenase	SORD	Q00796	0.001133	0.954266
seq.15604.18	15604-18	Mitogen-activated protein kinase 9	MAPK9	P45984	0.001133	0.647329
seq.18177.49	18177-49	NEDD8-activating enzyme E1 regulatory subunit	NAE1	Q13564	0.001133	0.573109
seq.18237.29	18237-29	Nuclear receptor-interacting protein 3	NRIP3	Q9NQ35	0.001133	-0.1227
seq.18327.6	18327-6	Sorting nexin-5	SNX5	Q9Y5X3	0.001133	0.465275
seq.18950.13	18950-13	Ras-related C3 botulinum toxin substrate 2	RAC2	P15153	0.001133	0.703615
seq.19273.3	19273-3	Glutathione reductase, mitochondrial	GSR	P00390	0.001133	0.724704
seq.19371.18	19371-18	Microtubule-associated protein RP/EB family member 1	MAPRE1	Q15691	0.001133	0.505613
seq.19446.1	19446-1	GMP reductase 2	GMPR2	Q9P2T1	0.001133	0.453252
seq.19503.2	19503-2	Sorting nexin-3	SNX3	O60493	0.001133	0.771289
seq.19748.3	19748-3	Diphosphomevalonate decarboxylase	MVD	P53602	0.001133	0.855694
seq.20590.13	20590-13	Neuropeptide Y	NPY	P01303	0.001133	0.617238
seq.20913.27	20913-27	Eukaryotic translation initiation factor 1	EIF1	P41567	0.001133	0.995464
seq.21131.109	21131-109	tRNA wybutosine-synthesizing protein 5	TYW5	A2RUC4	0.001133	0.684556
seq.21351.8	21351-8	Glycyl t-RNA synthetase	GARS1	P41250	0.001133	0.908098
seq.21802.53	21802-53	Endonuclease 8-like 1	NEIL1	Q96FI4	0.001133	0.793592
seq.22154.37	22154-37	GATA zinc finger domain-containing protein 1	GATAD1	Q8WUU5	0.001133	0.382521
seq.22970.8	22970-8	PDZ domain-containing protein GIPC1	GIPC1	O14908	0.001133	0.728802
seq.23631.1	23631-1	N-acetylgalactosamine kinase	GALK2	Q01415	0.001133	0.412849
seq.23703.8	23703-8	PDZ and LIM domain protein 5	PDLIM5	Q96HC4	0.001133	0.881403
seq.24443.8	24443-8	Mortality factor 4-like protein 2	MORF4L2	Q15014	0.001133	0.472532
seq.24472.28	24472-28	Sharpin	SHARPIN	Q9H0F6	0.001133	0.716271
seq.25036.10	25036-10	Ran GTPase-activating protein 1	RANGAP1	P46060	0.001133	0.390798
seq.25048.30	25048-30	Protein kinase C and casein kinase substrate in neurons protein 2	PACSIN2	Q9UNF0	0.001133	0.780877
seq.25053.1	25053-1	Cytoplasmic dynein 1 light intermediate chain 2	DYNC1LI2	O43237	0.001133	0.45072
seq.25089.21	25089-21	Transcription intermediary factor 1-beta	TRIM28	Q13263	0.001133	0.751214
seq.25116.11	25116-11	Coiled-coil domain-containing protein 9	CCDC9	Q9Y3X0	0.001133	0.478022
seq.2515.14	2515-14	GDNF family receptor alpha-2	GFRA2	O00451	0.001133	0.433001
seq.2859.69	2859-69	Histone deacetylase 8	HDAC8	Q9BY41	0.001133	0.382143
seq.3168.8	3168-8	A disintegrin and metalloproteinase with thrombospondin motifs 5	ADAMTS5	Q9UNA0	0.001133	-0.15227
seq.3197.70	3197-70	Insulin-degrading enzyme	IDE	P14735	0.001133	0.484876
seq.3331.8	3331-8	RGM domain family member B	RGMB	Q6NW40	0.001133	0.683538
seq.3392.68	3392-68	Protein kinase B gamma	AKT3	Q9Y243	0.001133	1.011212
seq.3894.15	3894-15	N-acetyl-D-glucosamine kinase	NAGK	Q9UJ70	0.001133	0.752777
seq.4498.62	4498-62	Neural cell adhesion molecule 1, 120 kDa isoform	NCAM1	P13591	0.001133	0.617155
seq.4696.2	4696-2	Fatty acid-binding protein, heart	FABP3	P05413	0.001133	0.801369
seq.5023.23	5023-23	Adenylosuccinate lyase	ADSL	P30566	0.001133	0.641006
seq.5360.9	5360-9	RAC-beta serine/threonine-protein kinase	AKT2	P31751	0.001133	0.741831
seq.5383.14	5383-14	Tumor necrosis factor receptor superfamily member 13C	TNFRSF13C	Q96RJ3	0.001133	0.308452
seq.5885.55	5885-55	Eukaryotic translation initiation factor 4H	EIF4H	Q15056	0.001133	1.107003
seq.7628.40	7628-40	Cysteine-rich with EGF-like domain protein 1	CRELD1	Q96HD1	0.001133	0.427887
seq.8885.6	8885-6	Voltage-dependent calcium channel subunit alpha-2/delta-3	CACNA2D3	Q8IZS8	0.001133	-0.08771
seq.9544.24	9544-24	Uncharacterized protein C7orf69	C7orf69	Q9H7B7	0.001133	0.293973
seq.9925.56	9925-56	Scavenger receptor class F member 2:Cytoplasmic domain	SCARF2	Q96GP6	0.001133	-0.11225
seq.8219.14	8219-14	Zymogen granule membrane protein 16	ZG16	O60844	0.001133	-0.12913
seq.12022.12	12022-12	Mothers against decapentaplegic homolog 4	SMAD4	Q13485	0.001133	0.628857
seq.12748.6	12748-6	Bromodomain testis-specific protein	BRDT	Q58F21	0.001133	-0.09726
seq.15394.79	15394-79	Netrin receptor UNC5B	UNC5B	Q8IZJ1	0.001133	0.439051
seq.17742.2	17742-2	Ras-related protein R-Ras	RRAS	P10301	0.001133	0.332541
seq.18284.77	18284-77	Protein KIBRA	WWC1	Q8IX03	0.001133	-0.18164
seq.20367.6	20367-6	L-amino-acid oxidase	IL4I1	Q96RQ9	0.001133	-0.10117

seq.21286.29	21286-29	60S ribosomal protein L11	RPL11	P62913	0.001133	0.482684
seq.11350.30	11350-30	E3 ubiquitin-protein ligase CHIP	STUB1	Q9UNE7	0.00136	0.786238
seq.25248.28	25248-28	Protein numb homolog	NUMB	P49757	0.00136	0.364516
seq.2705.5	2705-5	C-C motif chemokine 25	CCL25	O15444	0.00136	-0.12878
seq.3390.72	3390-72	PIK3CA/PIK3R1	PIK3CA|PIK3R1	P42336|P27986	0.00136	0.603906
seq.11105.171	11105-171	Alpha-enolase	ENO1	P06733	0.001371	0.824084
seq.11440.58	11440-58	Suppressor of cytokine signaling 3	SOCS3	O14543	0.001371	0.341362
seq.11493.169	11493-169	Dynein light chain 2, cytoplasmic	DYNLL2	Q96FJ2	0.001371	0.819541
seq.12351.25	12351-25	Signal transducer and activator of transcription 1-alpha/beta	STAT1	P42224	0.001371	0.86263
seq.12392.30	12392-30	ADP-ribosylation factor-like protein 1	ARL1	P40616	0.001371	0.275357
seq.12494.99	12494-99	Gamma-aminobutyric acid receptor-associated protein-like 2	GABARAPL2	P60520	0.001371	0.809686
seq.12993.21	12993-21	Nuclear protein localization protein 4 homolog	NPLOC4	Q8TAT6	0.001371	0.77258
seq.13059.33	13059-33	Riboflavin kinase	RFK	Q969G6	0.001371	0.596554
seq.13501.10	13501-10	Solute carrier family 35 member G2	SLC35G2	Q8TBE7	0.001371	0.257886
seq.13522.20	13522-20	Visinin-like protein 1	VSNL1	P62760	0.001371	0.240766
seq.13530.5	13530-5	Urotensin-2 receptor	UTS2R	Q9UKP6	0.001371	-0.11476
seq.13572.43	13572-43	26S proteasome non-ATPase regulatory subunit 11	PSMD11	O00231	0.001371	0.681744
seq.13594.158	13594-158	ADP-ribosylation factor-binding protein GGA1	GGA1	Q9UJY5	0.001371	0.710759
seq.17224.12	17224-12	Mimecan	OGN	P20774	0.001371	-0.09903
seq.17350.13	17350-13	Charged multivesicular body protein 2b	CHMP2B	Q9UQN3	0.001371	0.696022
seq.17776.15	17776-15	Peroxisomal trans-2-enoyl-CoA reductase	PECR	Q9BY49	0.001371	-0.07725
seq.17816.58	17816-58	Neurocalcin-delta	NCALD	P61601	0.001371	0.248212
seq.19150.20	19150-20	Phosphoribosyl pyrophosphate synthase-associated protein 2	PRPSAP2	O60256	0.001371	0.879421
seq.19586.89	19586-89	Ras-related protein Rab-3C	RAB3C	Q96E17	0.001371	0.276118
seq.20167.6	20167-6	Ras-related protein Rab-8B	RAB8B	Q92930	0.001371	1.340607
seq.20370.6	20370-6	Acylphosphatase-1	ACYP1	P07311	0.001371	0.488031
seq.20550.38	20550-38	Neurotrimin	NTM	Q9P121	0.001371	1.369263
seq.20935.4	20935-4	Phosducin-like protein 3	PDCL3	Q9H2J4	0.001371	0.758901
seq.20960.47	20960-47	Far upstream element-binding protein 1	FUBP1	Q96AE4	0.001371	0.754935
seq.21369.15	21369-15	RNA 3'-terminal phosphate cyclase	RTCA	O00442	0.001371	1.189067
seq.21475.137	21475-137	Thioredoxin-like protein 1	TXNL1	O43396	0.001371	0.261066
seq.21504.41	21504-41	Eukaryotic translation initiation factor 2C 2	AGO2	Q9UKV8	0.001371	0.724537
seq.21743.1	21743-1	WD repeat-containing protein 48	WDR48	Q8TAF3	0.001371	0.919692
seq.22037.47	22037-47	EF-hand domain-containing protein D2	EFHD2	Q96C19	0.001371	0.386309
seq.25085.37	25085-37	Tether containing UBX domain for GLUT4	ASPSCR1	Q9BZE9	0.001371	0.440518
seq.25217.35	25217-35	Sperm protein associated with the nucleus on the X chromosome A	SPANXA1	Q9NS26	0.001371	0.241858
seq.2870.29	2870-29	Ras-related C3 botulinum toxin substrate 1	RAC1	P63000	0.001371	1.021145
seq.3825.18	3825-18	Mitogen-activated protein kinase 8	MAPK8	P45983	0.001371	0.390258
seq.5464.52	5464-52	Growth factor receptor-bound protein 2	GRB2	P62993	0.001371	0.682851
seq.5489.18	5489-18	Stress-induced-phosphoprotein 1	STIP1	P31948	0.001371	0.825659
seq.5682.13	5682-13	Vasorin	VASN	Q6EMK4	0.001371	0.96563
seq.5934.1	5934-1	Ferritin	FTH1|FTL	P02794|P02792	0.001371	0.788032
seq.9843.5	9843-5	Alpha-actinin-1	ACTN1	P12814	0.001371	0.914292
seq.9846.32	9846-32	Rho GDP-dissociation inhibitor 2	ARHGDIB	P52566	0.001371	0.719545
seq.9849.13	9849-13	DNA-(apurinic or apyrimidinic site) lyase	APEX1	P27695	0.001371	0.731339
seq.12384.92	12384-92	COP9 signalosome complex subunit 7b	COPS7B	Q9H9Q2	0.001392	0.159381
seq.23255.7	23255-7	Ras-related protein Rab-33A	RAB33A	Q14088	0.001392	0.295387
seq.21117.18	21117-18	Zinc finger C4H2 domain-containing protein	ZC4H2	Q9NQZ6	0.001393	0.406704
seq.10953.14	10953-14	C-type lectin domain family 2 member A	CLEC2A	Q6UVW9	0.001522	-0.16414
seq.18403.25	18403-25	AMP deaminase 2	AMPD2	Q01433	0.001522	0.532861
seq.19180.38	19180-38	PTB domain-containing engulfment adapter protein 1	GULP1	Q9UBP9	0.001522	0.391279
seq.18268.5	18268-5	G antigen 2A	GAGE2A	Q6NT46	0.00164	0.322603
seq.11096.57	11096-57	HemK methyltransferase family member 2	N6AMT1	Q9Y5N5	0.00164	0.257099
seq.11226.16	11226-16	Ubiquitin-protein ligase E3A	UBE3A	Q05086	0.00164	0.614607
seq.11237.49	11237-49	Procollagen C-endopeptidase enhancer 1	PCOLCE	Q15113	0.00164	1.493962
seq.11633.89	11633-89	Activator of 90 kDa heat shock protein ATPase homolog 1	AHSA1	O95433	0.00164	0.487928
seq.12358.6	12358-6	Immunoglobulin-binding protein 1	IGBP1	P78318	0.00164	0.699272
seq.12432.23	12432-23	Calcyclin-binding protein	CACYBP	Q9HB71	0.00164	0.618102
seq.12603.87	12603-87	Polyadenylate-binding protein 4	PABPC4	Q13310	0.00164	0.913817
seq.13044.5	13044-5	Tumor susceptibility gene 101 protein	TSG101	Q99816	0.00164	0.795468
seq.13434.172	13434-172	Alpha-parvin	PARVA	Q9NVD7	0.00164	0.931526
seq.14072.9	14072-9	Translationally-controlled tumor protein	TPT1	P13693	0.00164	0.629989
seq.15301.24	15301-24	Acrosomal protein SP-10	ACRV1	P26436	0.00164	-0.12527
seq.17380.2	17380-2	Ubiquitin-conjugating enzyme E2 K	UBE2K	P61086	0.00164	0.820278
seq.17697.2	17697-2	Esterase OVCA2	OVCA2	Q8WZ82	0.00164	0.781109
seq.17729.20	17729-20	Ubiquitin-conjugating enzyme E2 S	UBE2S	Q16763	0.00164	0.57822
seq.17781.191	17781-191	Microtubule-associated proteins 1A/1B light chain 3A	MAP1LC3A	Q9H492	0.00164	1.014409
seq.17818.22	17818-22	Protein SGT1 homolog	SUGT1	Q9Y2Z0	0.00164	0.882666
seq.18331.3	18331-3	Glucosylceramidase	GBA	P04062	0.00164	0.376782
seq.18383.9	18383-9	Peptidyl-prolyl cis-trans isomerase FKBP3	FKBP3	Q00688	0.00164	0.9499
seq.19239.5	19239-5	ADP-ribosylation factor 1	ARF1	P84077	0.00164	0.451752
seq.20386.15	20386-15	Putative peptidyl-tRNA hydrolase PTRHD1	PTRHD1	Q6GMV3	0.00164	0.811399
seq.20432.6	20432-6	Pyroglutamyl-peptidase 1	PGPEP1	Q9NXJ5	0.00164	0.843052
seq.20915.68	20915-68	Adaptin ear-binding coat-associated protein 2	NECAP2	Q9NVZ3	0.00164	0.669443
seq.21188.38	21188-38	RWD domain-containing protein 4	RWDD4	Q6NW29	0.00164	0.868659
seq.21290.66	21290-66	Checkpoint protein HUS1	HUS1	O60921	0.00164	0.215243
seq.21373.8	21373-8	Queuine tRNA-ribosyltransferase subunit QTRTD1	QTRT2	Q9H974	0.00164	0.287163
seq.21737.20	21737-20	Beta-galactoside alpha-2,6-sialyltransferase 2	ST6GAL2	Q96JF0	0.00164	0.847692
seq.22564.5	22564-5	Low-density lipoprotein receptor class A domain-containing protein 3	LDLRAD3	Q86YD5	0.00164	0.237558
seq.23387.18	23387-18	TIP41-like protein	TIPRL	O75663	0.00164	0.796146
seq.24475.45	24475-45	Sorting nexin-12	SNX12	Q9UMY4	0.00164	0.772061
seq.25286.33	25286-33	Rab GTPase-activating protein 1-like	RABGAP1L	Q5R372	0.00164	0.76246
seq.25298.53	25298-53	Lysine-specific demethylase 4C	KDM4C	Q9H3R0	0.00164	0.745235

seq.3079.62	3079-62	Retinoic acid receptor responder protein 2	RARRES2	Q99969	0.00164	0.668706
seq.3405.6	3405-6	Ubiquitin-fold modifier-conjugating enzyme 1	UFC1	Q9Y3C8	0.00164	0.654015
seq.3836.51	3836-51	Ubiquitin-fold modifier 1	UFM1	P61960	0.00164	0.401252
seq.4145.58	4145-58	Neurotrophin-3	NTF3	P20783	0.00164	-0.2051
seq.4250.23	4250-23	NSFL1 cofactor p47	NSFL1C	Q9UNZ2	0.00164	0.581788
seq.5006.71	5006-71	Mitogen-activated protein kinase 13	MAPK13	O15264	0.00164	-0.18701
seq.5008.51	5008-51	Superoxide dismutase [Mn], mitochondrial	SOD2	P04179	0.00164	0.497932
seq.5095.21	5095-21	Killer cell immunoglobulin-like receptor 2DL4	KIR2DL4	Q99706	0.00164	0.357529
seq.5490.53	5490-53	Testican-1	SPOCK1	Q08629	0.00164	-0.52819
seq.5861.78	5861-78	3-hydroxyanthranilate 3,4-dioxygenase	HAAO	P46952	0.00164	0.723323
seq.7768.10	7768-10	Carboxypeptidase M	CPM	P14384	0.00164	0.296284
seq.7859.21	7859-21	Protocadherin gamma-C3	PCDHGC3	Q9UN70	0.00164	0.338418
seq.8328.9	8328-9	Ethanolamine kinase 1	ETNK1	Q9HBU6	0.00164	-0.10538
seq.9456.34	9456-34	Interleukin-22 receptor subunit alpha-2	IL22RA2	Q969J5	0.00164	-0.10384
seq.9756.6	9756-6	Transgelin	TAGLN	Q01995	0.00164	1.161371
seq.9815.5	9815-5	Rho GTPase-activating protein 1:Cellular retinaldehyde-TRIO domain	ARHGAP1	Q07960	0.00164	0.901896
seq.3209.69	3209-69	Matrix extracellular phosphoglycoprotein	MEPE	Q9NQ76	0.001684	-0.13853
seq.10382.1	10382-1	Angiopoietin-related protein 3	ANGPTL3	Q9Y5C1	0.001684	-0.15821
seq.12751.26	12751-26	Zinc fingers and homeoboxes protein 1	ZHX1	Q9UKY1	0.001684	-0.12385
seq.12853.112	12853-112	Tropomodulin-2	TMOD2	Q9NZR1	0.001684	0.383643
seq.21111.49	21111-49	Hsp70-binding protein 1	HSPBP1	Q9NZL4	0.001684	0.730348
seq.23691.2	23691-2	Tubulin--tyrosine ligase-like protein 12	TTLL12	Q14166	0.001684	0.38442
seq.3235.50	3235-50	WAP, Kazal, immunoglobulin, Kunitz and NTR domain-containing protein 2	WFIKKN2	Q8TEU8	0.001684	-0.11757
seq.5465.32	5465-32	Heparan-sulfate 6-O-sulfotransferase 1	HS6ST1	O60243	0.001684	0.900365
seq.22141.59	22141-59	6-phosphofructo-2-kinase/fructose-2,6-bisphosphatase 1	PFKFB1	P16118	0.00183	0.219095
seq.6606.61	6606-61	Metastasis-suppressor KiSS-1	KISS1	Q15726	0.001831	0.387884
seq.6570.1	6570-1	Collagen alpha-1(XIII) chain	COL13A1	Q5TAT6	0.001831	-0.14634
seq.12398.15	12398-15	Paired box protein Pax-4	PAX4	O43316	0.001831	-0.17667
seq.6380.23	6380-23	Peptidyl-tRNA hydrolase ICT1, mitochondrial	MRPL58	Q14197	0.001831	0.397004
seq.24923.27	24923-27	Secernin-3	SCRN3	Q0VDG4	0.001906	-0.20901
seq.18275.5	18275-5	Cysteine-rich protein 1	CRIP1	P50238	0.001906	0.41406
seq.20139.57	20139-57	Endophilin-A1	SH3GL2	Q99962	0.001906	0.87892
seq.23597.11	23597-11	Arf-GAP domain and FG repeat-containing protein 2	AGFG2	O95081	0.001906	0.535221
seq.3499.77	3499-77	Interleukin-17B	IL17B	Q9UHF5	0.001906	0.751035
seq.7856.51	7856-51	Protein FAM151A	FAM151A	Q8WW52	0.001906	-0.10069
seq.8891.7	8891-7	Probable serine carboxypeptidase CPVL	CPVL	Q9H3G5	0.001906	-0.09646
seq.10339.48	10339-48	Gamma-enolase	ENO2	P09104	0.001906	0.812055
seq.10479.18	10479-18	Stromelysin-2	MMP10	P09238	0.001906	0.200781
seq.11205.10	11205-10	Integrin beta-7	ITGB7	P26010	0.001906	0.205512
seq.11421.10	11421-10	EH domain-containing protein 4	EHD4	Q9H223	0.001906	0.361771
seq.11560.76	11560-76	Nuclear factor of activated T-cells, cytoplasmic 4	NFATC4	Q14934	0.001906	-0.11749
seq.11649.3	11649-3	Stromal membrane-associated protein 1	SMAP1	Q8IYB5	0.001906	0.603267
seq.12449.16	12449-16	Peptidyl-prolyl cis-trans isomerase H	PPIH	O43447	0.001906	0.80224
seq.12562.1	12562-1	Serine/threonine-protein kinase N1	PKN1	Q16512	0.001906	0.146912
seq.12812.25	12812-25	Acylphosphatase-2	ACYP2	P14621	0.001906	0.795722
seq.13123.3	13123-3	Leucine-rich repeat transmembrane protein FLRT3:Extracellular domain	FLRT3	Q9NZU0	0.001906	0.341185
seq.13236.25	13236-25	Protein Wnt-3a	WNT3A	P56704	0.001906	0.249599
seq.13939.14	13939-14	UTP--glucose-1-phosphate uridylyltransferase	UGP2	Q16851	0.001906	0.912852
seq.15468.14	15468-14	Complement factor H-related protein 1	CFHR1	Q03591	0.001906	0.449888
seq.15491.20	15491-20	Endosialin	CD248	Q9HCU0	0.001906	1.099074
seq.15521.4	15521-4	Calsyntenin-1	CLSTN1	O94985	0.001906	0.559623
seq.15523.9	15523-9	Delta-aminolevulinic acid dehydratase	ALAD	P13716	0.001906	0.465961
seq.16292.288	16292-288	Gastric inhibitory polypeptide	GIP	P09681	0.001906	0.178941
seq.17761.2	17761-2	ADP-sugar pyrophosphatase	NUDT5	Q9UKK9	0.001906	0.481027
seq.17769.28	17769-28	PEST proteolytic signal-containing nuclear protein	PCNP	Q8WW12	0.001906	0.712622
seq.17783.9	17783-9	Cob(I)yrinic acid a,c-diamide adenosyltransferase, mitochondrial	MMAB	Q96EY8	0.001906	-0.34872
seq.18216.22	18216-22	Interleukin-11 receptor subunit alpha	IL11RA	Q14626	0.001906	-0.13643
seq.18396.10	18396-10	Amino-terminal enhancer of split	TLE5	Q08117	0.001906	0.266562
seq.18813.15	18813-15	ATP-dependent RNA helicase DDX19A	DDX19A	Q9NUU7	0.001906	0.635213
seq.20116.30	20116-30	Serine/threonine-protein kinase OSR1	OXSR1	O95747	0.001906	0.950314
seq.20950.159	20950-159	Phosphoribosyltransferase domain-containing protein 1	PRTFDC1	Q9NRG1	0.001906	0.479947
seq.21326.28	21326-28	Tubulin-specific chaperone C	TBCC	Q15814	0.001906	0.556927
seq.21393.62	21393-62	Adenosylhomocysteinase	AHCY	P23526	0.001906	0.955601
seq.21724.22	21724-22	N-acetylgalactosamine-6-sulfatase	GALNS	P34059	0.001906	0.528805
seq.21747.8	21747-8	UBE2N (Ubc13)/Uev1a Complex	UBE2N|UBE2V1	P61088|Q13404	0.001906	0.773305
seq.21756.5	21756-5	UBE2N/UBE2V2 Complex	UBE2N|UBE2V2	P61088|Q15819	0.001906	0.700917
seq.22019.21	22019-21	SH2B adapter protein 3	SH2B3	Q9UQQ2	0.001906	-0.08565
seq.22371.46	22371-46	Protein FAM110A	FAM110A	Q9BQ89	0.001906	0.42715
seq.22572.19	22572-19	Pepsin A-4	PGA4	P0DJD7	0.001906	0.206316
seq.23319.6	23319-6	AN1-type zinc finger protein 2B	ZFAND2B	Q8WV99	0.001906	0.509358
seq.23668.281	23668-281	Mitochondrial dynamics protein MID51	MIEF1	Q9NQG6	0.001906	0.24641
seq.23680.1	23680-1	MCP-1 Induced Protein	ZC3H12A	Q5D1E8	0.001906	0.188475
seq.24426.15	24426-15	Catenin alpha-1	CTNNA1	P35221	0.001906	0.539408
seq.24476.18	24476-18	Activating signal cointegrator 1 complex subunit 2	ASCC2	Q9H1I8	0.001906	0.569578
seq.2625.53	2625-53	Hsp90alpha	HSP90AA1	P07900	0.001906	0.864116
seq.2642.4	2642-4	Platelet-activating factor acetylhydrolase IB subunit beta	PAFAH1B2	P68402	0.001906	0.454235
seq.3864.5	3864-5	40S ribosomal protein S7	RPS7	P62081	0.001906	0.291392
seq.3905.62	3905-62	Ubiquitin-conjugating enzyme E2 N	UBE2N	P61088	0.001906	0.711217
seq.5124.69	5124-69	Intercellular adhesion molecule 5	ICAM5	Q9UMF0	0.001906	-0.12928
seq.5491.12	5491-12	Testican-2	SPOCK2	Q92563	0.001906	0.598698
seq.5609.92	5609-92	Protein FAM19A5	TAFA5	Q7Z5A7	0.001906	-0.08462

seq.6900.30	6900-30	Ubiquitin-conjugating enzyme E2 J1	UBE2J1	Q9Y385	0.001906	0.106828
seq.7842.52	7842-52	Small EDRK-rich factor 1	SERF1A	O75920	0.001906	-0.09648
seq.7878.2	7878-2	Protein transport protein Sec61 subunit beta	SEC61B	P60468	0.001906	-0.11674
seq.8474.6	8474-6	Inactive tyrosine-protein kinase transmembrane receptor ROR1	ROR1	Q01973	0.001906	0.700849
seq.8907.11	8907-11	UDP-glucuronosyltransferase 2A1	UGT2A1	Q9Y4X1	0.001906	0.123114
seq.9391.60	9391-60	ProSAAS	PCSK1N	Q9UHG2	0.001906	0.463176
seq.9296.15	9296-15	Receptor-type tyrosine-protein phosphatase delta	PTPRD	P23468	0.002011	-0.0806
seq.4123.60	4123-60	Fibroblast growth factor 4	FGF4	P08620	0.002011	-0.15198
seq.7784.1	7784-1	Kininogen-1	KNG1	P01042	0.002011	-0.10636
seq.2654.19	2654-19	Tumor necrosis factor receptor superfamily member 1A	TNFRSF1A	P19438	0.002012	0.412638
seq.24263.6	24263-6	Occludin	OCLN	Q16625	0.002163	-0.11126
seq.7194.36	7194-36	Neuroplastin	NPTN	Q9Y639	0.002163	0.271106
seq.12803.9	12803-9	Zinc finger protein 329	ZNF329	Q86UD4	0.002163	-0.11762
seq.4584.5	4584-5	Melittin_VESMG	MELT	P68408	0.002163	-0.13041
seq.8074.32	8074-32	Transmembrane protein 70, mitochondrial	TMEM70	Q9BUB7	0.002163	-0.1359
seq.12676.1	12676-1	Protein kinase C and casein kinase substrate in neurons protein 1	PACSIN1	Q9BY11	0.002163	0.307683
seq.21755.27	21755-27	Ubiquitin carboxyl-terminal hydrolase 12	USP12	O75317	0.002163	0.433628
seq.21799.15	21799-15	Homer protein homolog 3	HOMER3	Q9NSC5	0.002163	0.381415
seq.3212.30	3212-30	Neutral ceramidase	ASAH2	Q9NR71	0.002163	-0.14346
seq.4128.27	4128-27	C-C motif chemokine 24	CCL24	O00175	0.002163	-0.12497
seq.4143.74	4143-74	Lymphotactin	XCL1	P47992	0.002163	-0.11714
seq.7045.4	7045-4	BCL2/adenovirus E1B 19 kDa protein-interacting protein 3	BNIP3	Q12983	0.002163	0.171481
seq.12893.159	12893-159	Gamma-interferon-inducible protein 16:Isoform 2, Hematopoietic expression, interferon-inducible nature, and nuclear localization 2	IFI16	Q16666	0.002193	0.247407
seq.6237.11	6237-11	Fibroblast growth factor receptor-like 1	FGFRL1	Q8N441	0.002193	0.338517
seq.6234.74	6234-74	Vitrin	VIT	Q6UXI7	0.002193	-0.14159
seq.11270.17	11270-17	Coiled-coil-helix-coiled-coil-helix domain-containing protein 10, mitochondrial	CHCHD10	Q8WYQ3	0.002193	0.653168
seq.11667.29	11667-29	Tensin-2	TNS2	Q63HR2	0.002193	-0.12589
seq.12476.50	12476-50	Fructose-2,6-bisphosphatase TIGAR	TIGAR	Q9NQ88	0.002193	0.595388
seq.12516.13	12516-13	Transcriptional enhancer factor TEF-3	TEAD4	Q15561	0.002193	0.457137
seq.12530.14	12530-14	Cyclin-dependent kinases regulatory subunit 1	CKS1B	P61024	0.002193	0.549584
seq.12571.14	12571-14	ADP-ribosylation factor-like protein 3	ARL3	P36405	0.002193	0.731102
seq.12830.4	12830-4	Histone deacetylase complex subunit SAP18	SAP18	O00422	0.002193	0.28398
seq.15620.4	15620-4	Neuroligin-1	NLGN1	Q8N2Q7	0.002193	0.196285
seq.15640.54	15640-54	Transgelin	TAGLN	Q01995	0.002193	1.077036
seq.16060.99	16060-99	Nidogen-2	NID2	Q14112	0.002193	-0.54071
seq.17151.84	17151-84	Interferon regulatory factor 3	IRF3	Q14653	0.002193	0.81704
seq.17450.51	17450-51	Histidine--tRNA ligase, cytoplasmic	HARS1	P12081	0.002193	0.764906
seq.17756.69	17756-69	Deoxycytidylate deaminase	DCTD	P32321	0.002193	0.494608
seq.17765.3	17765-3	Snurportin-1	SNUPN	O95149	0.002193	0.661524
seq.19295.32	19295-32	Serine/threonine-protein phosphatase PP1-gamma catalytic subunit	PPP1CC	P36873	0.002193	0.86002
seq.19333.4	19333-4	Heterogeneous nuclear ribonucleoprotein K	HNRNPK	P61978	0.002193	0.964603
seq.19568.17	19568-17	Interleukin-15	IL15	P40933	0.002193	0.497713
seq.20141.42	20141-42	Transcription elongation factor A protein 2	TCEA2	Q15560	0.002193	0.572449
seq.20385.21	20385-21	Ceramide-1-phosphate transfer protein	CPTP	Q5TA50	0.002193	0.300717
seq.21190.4	21190-4	Ubiquitin-conjugating enzyme E2 W	UBE2W	Q96B02	0.002193	0.466959
seq.21210.33	21210-33	DNA polymerase beta	POLB	P06746	0.002193	0.511421
seq.21219.7	21219-7	RPA-interacting protein	RPAIN	Q86UA6	0.002193	-0.08079
seq.21321.2	21321-2	U2 small nuclear ribonucleoprotein AAE	SNRPA1	P09661	0.002193	0.501437
seq.21976.4	21976-4	I-kappa-B kinase gamma	IKBKG	Q9Y6K9	0.002193	0.242278
seq.21996.28	21996-28	NEDD8 ultimate buster 1	NUB1	Q9Y5A7	0.002193	0.472772
seq.22958.6	22958-6	Destrin	DSTN	P60981	0.002193	0.80721
seq.23396.21	23396-21	Twinfilin-2	TWF2	Q6IBS0	0.002193	0.777506
seq.23660.112	23660-112	Kelch-like protein 41	KLHL41	O60662	0.002193	0.948277
seq.23686.44	23686-44	CWF19-like protein 1	CWF19L1	Q69YN2	0.002193	0.728977
seq.24426.191	24426-191	Catenin alpha-1	CTNNA1	P35221	0.002193	0.580166
seq.24671.15	24671-15	Apoptosis-associated speck-like protein containing a CARD	PYCARD	Q9ULZ3	0.002193	0.433085
seq.24723.58	24723-58	Eukaryotic translation initiation factor 2 subunit 2	EIF2S2	P20042	0.002193	0.665766
seq.24926.9	24926-9	Far upstream element-binding protein 2	KHSRP	Q92945	0.002193	0.707418
seq.25087.11	25087-11	Dystrobrevin alpha	DTNA	Q9Y4J8	0.002193	0.569195
seq.25245.22	25245-22	Synembryn-A	RIC8A	Q9NPQ8	0.002193	0.798853
seq.25257.162	25257-162	Oligodendrocyte transcription factor 1	OLIG1	Q8TAK6	0.002193	-0.12084
seq.25918.60	25918-60	HLA class I histocompatibility antigen, alpha chain E	HLA-E	P13747	0.002193	0.415096
seq.25948.18	25948-18	Serine/threonine-protein kinase N2	PKN2	Q16513	0.002193	0.342561
seq.2615.60	2615-60	Ephrin-A5	EFNA5	P52803	0.002193	0.420254
seq.2878.66	2878-66	Tyrosine-protein kinase Yes	YES1	P07947	0.002193	0.372164
seq.2938.55	2938-55	Apolipoprotein E (isoform E4)	APOE	P02649	0.002193	0.638357
seq.3115.64	3115-64	Mitogen-activated protein kinase 1	MAPK1	P28482	0.002193	0.650528
seq.3336.50	3336-50	Tissue factor pathway inhibitor	TFPI	P10646	0.002193	1.482526
seq.3633.70	3633-70	Nidogen-2	NID2	Q14112	0.002193	-0.52986
seq.3714.49	3714-49	Creatine kinase M-type:Creatine kinase B-type heterodimer	CKB|CKM	P12277|P06732	0.002193	1.410045
seq.3872.2	3872-2	Translationally-controlled tumor protein	TPT1	P13693	0.002193	0.754037
seq.4192.10	4192-10	Alcohol dehydrogenase [NADP(+)]	AKR1A1	P14550	0.002193	0.735582
seq.4276.10	4276-10	Phosphatidylethanolamine-binding protein 1	PEBP1	P30086	0.002193	0.672469
seq.4978.54	4978-54	Drebrin-like protein	DBNL	Q9UJU6	0.002193	0.648229
seq.5193.51	5193-51	GTPase KRas	KRAS	P01116	0.002193	-0.09746
seq.5654.70	5654-70	Protein disulfide-isomerase TMX3	TMX3	Q96JJ7	0.002193	0.946967
seq.6651.74	6651-74	PolyUbiquitin K48-linked	UBB	P0CG47	0.002193	0.49854
seq.6925.26	6925-26	Sorting nexin-8	SNX8	Q9Y5X2	0.002193	0.742982
seq.8749.194	8749-194	Antigen-presenting glycoprotein CD1d	CD1D	P15813	0.002193	-0.1158
seq.8791.151	8791-151	Carbonic anhydrase 5A, mitochondrial	CA5A	P35218	0.002193	-0.10046

seq.8887.21	8887-21	Nuclear migration protein nudC:CS domain	NUDC	Q9Y266	0.002193	0.782425
seq.9021.1	9021-1	Hepatitis A virus cellular receptor 1	HAVCR1	Q96D42	0.002193	-0.12291
seq.9525.1	9525-1	Inactive tyrosine-protein kinase 7	PTK7	Q13308	0.002193	1.007065
seq.9742.59	9742-59	Spliceosome RNA helicase DDX39B	DDX39B	Q13838	0.002193	0.784914
seq.9877.28	9877-28	Crk-like protein	CRKL	P46109	0.002193	0.760336
seq.9886.28	9886-28	DNA repair protein XRCC4	XRCC4	Q13426	0.002193	0.8559
seq.13940.19	13940-19	Gamma-interferon-inducible protein 16:Isoform 2, Hematopoietic expression, interferon-inducible nature, and nuclear localization 1	IFI16	Q16666	0.002355	0.235838
seq.18332.17	18332-17	Complexin-1	CPLX1	O14810	0.002355	0.495464
seq.18914.188	18914-188	Platelet-activating factor acetylhydrolase 2, cytoplasmic	PAFAH2	Q99487	0.002355	0.260465
seq.10801.11	10801-11	Ephrin-A2	EFNA2	O43921	0.002355	0.308902
seq.13929.27	13929-27	Peroxisomal carnitine O-octanoyltransferase	CROT	Q9UKG9	0.002355	-0.13143
seq.14090.23	14090-23	Differentially expressed in FDCP 6 homolog	DEF6	Q9H4E7	0.002355	-0.10636
seq.22086.2	22086-2	Breast cancer metastasis-suppressor 1-like protein	BRMS1L	Q5PSV4	0.002355	-0.20228
seq.23376.56	23376-56	Actin-related protein 2/3 complex subunit 2	ARPC2	O15144	0.002355	0.445854
seq.24687.18	24687-18	Alpha-tocopherol transfer protein-like	TTPAL	Q9BTX7	0.002355	0.457587
seq.3236.12	3236-12	Glycogen synthase kinase-3 beta	GSK3B	P49841	0.002355	1.294374
seq.6940.18	6940-18	Junctophilin-1	JPH1	Q9HDC5	0.002355	-0.09546
seq.19120.33	19120-33	D-amino-acid oxidase	DAO	P14920	0.002556	-0.12512
seq.12437.18	12437-18	Serine/threonine-protein kinase ULK3	ULK3	Q6PHR2	0.002556	-0.10566
seq.10045.47	10045-47	Cullin-3	CUL3	Q13618	0.002556	0.408888
seq.16825.20	16825-20	Ataxin-3	ATXN3	P54252	0.002556	0.607556
seq.18232.42	18232-42	Phosphatidylinositol 5-phosphate 4-kinase type-2 beta	PIP4K2B	P78356	0.002556	0.336758
seq.8059.1	8059-1	Thrombopoietin	THPO	P40225	0.002556	-0.15969
seq.10058.1	10058-1	UV excision repair protein RAD23 homolog A	RAD23A	P54725	0.002587	0.746145
seq.11160.56	11160-56	RING finger protein 122	RNF122	Q9H9V4	0.002587	0.138054
seq.11444.49	11444-49	DNA-directed RNA polymerase III subunit RPC6	POLR3F	Q9H1D9	0.002587	-0.06692
seq.11833.83	11833-83	Frataxin, mitochondrial	FXN	Q16595	0.002587	-0.08906
seq.12458.79	12458-79	Calcineurin B homologous protein 1	CHP1	Q99653	0.002587	0.234213
seq.12758.47	12758-47	Glutamate receptor ionotropic, delta-2	GRID2	O43424	0.002587	-0.13134
seq.12795.2	12795-2	Zinc finger protein 566	ZNF566	Q969W8	0.002587	-0.09049
seq.12843.6	12843-6	Zinc finger protein 410	ZNF410	Q86VK4	0.002587	-0.09828
seq.12861.13	12861-13	Tropomodulin-3	TMOD3	Q9NYL9	0.002587	0.681139
seq.13114.50	13114-50	Lumican	LUM	P51884	0.002587	1.784777
seq.13590.1	13590-1	Oligoribonuclease, mitochondrial	REXO2	Q9Y3B8	0.002587	0.811772
seq.13618.15	13618-15	Mitotic spindle assembly checkpoint protein MAD1	MAD1L1	Q9Y6D9	0.002587	-0.1585
seq.15529.33	15529-33	Cysteine and glycine-rich protein 1	CSRP1	P21291	0.002587	0.831877
seq.15545.13	15545-13	Calcineurin subunit B type 1	PPP3R1	P63098	0.002587	0.575757
seq.17320.19	17320-19	L-aminoadipate-semialdehyde dehydrogenase-phosphopantetheinyl transferase	AASDHPPT	Q9NRN7	0.002587	0.763787
seq.18181.2	18181-2	Protein TSSC4	TSSC4	Q9Y5U2	0.002587	0.591209
seq.19110.6	19110-6	Protein MEMO1	MEMO1	Q9Y316	0.002587	0.261094
seq.19124.9	19124-9	Ubiquitin-like domain-containing CTD phosphatase 1	UBLCP1	Q8WVY7	0.002587	0.800589
seq.19134.66	19134-66	DNA-directed RNA polymerases I, II, and III subunit RPABC4	POLR2K	P53803	0.002587	0.375571
seq.19176.27	19176-27	Protein FAM49B	CYRIB	Q9NUQ9	0.002587	0.656035
seq.19249.18	19249-18	Lysine--tRNA ligase	KARS1	Q15046	0.002587	0.867499
seq.20057.177	20057-177	Histone chaperone ASF1B	ASF1B	Q9NVP2	0.002587	0.533951
seq.20578.10	20578-10	Latrophilin-3	ADGRL3	Q9HAR2	0.002587	0.746367
seq.20947.50	20947-50	Protein Dr1	DR1	Q01658	0.002587	0.517384
seq.21436.56	21436-56	Cysteine protease ATG4A	ATG4A	Q8WYN0	0.002587	0.225513
seq.21717.44	21717-44	Ubiquitin-conjugating enzyme E2 Q2	UBE2Q2	Q8WVN8	0.002587	0.548068
seq.21754.5	21754-5	Ubiquitin thioesterase ZRANB1	ZRANB1	Q9UGI0	0.002587	0.255829
seq.21935.16	21935-16	Ubiquitin-conjugating enzyme E2 H	UBE2H	P62256	0.002587	-0.12909
seq.22043.174	22043-174	Heat shock-related 70 kDa protein 2	HSPA2	P54652	0.002587	0.56822
seq.22155.44	22155-44	Gem-associated protein 7	GEMIN7	Q9H840	0.002587	0.174021
seq.23584.2	23584-2	Nucleotide triphosphate diphosphatase NUDT15	NUDT15	Q9NV35	0.002587	0.862235
seq.23662.10	23662-10	SH3 domain-binding protein 5	SH3BP5	O60239	0.002587	0.29264
seq.25272.17	25272-17	Cadherin-23	CDH23	Q9H251	0.002587	0.615372
seq.2643.57	2643-57	Cadherin-3	CDH3	P22223	0.002587	0.35313
seq.2658.27	2658-27	NT-3 growth factor receptor	NTRK3	Q16288	0.002587	-0.11483
seq.2846.24	2846-24	Ubiquitin+1, truncated mutation for UbB	RPS27A	P62979	0.002587	0.721755
seq.3347.9	3347-9	beta-adrenergic receptor kinase 1	GRK2	P25098	0.002587	0.665308
seq.3351.1	3351-1	Calcium/calmodulin-dependent protein kinase type II subunit beta	CAMK2B	Q13554	0.002587	0.69311
seq.4237.70	4237-70	Leucine carboxyl methyltransferase 1	LCMT1	Q9UIC8	0.002587	0.313118
seq.5018.68	5018-68	Peroxiredoxin-6	PRDX6	P30041	0.002587	0.63147
seq.6172.7	6172-7	PolyUbiquitin K63-linked	UBC	P0CG48	0.002587	0.527901
seq.6274.15	6274-15	WSC domain-containing protein 2	WSCD2	Q2TBF2	0.002587	0.810777
seq.6377.54	6377-54	Lysozyme-like protein 2	LYZL2	Q7Z4W2	0.002587	0.483721
seq.6986.17	6986-17	Heparan sulfate glucosamine 3-O-sulfotransferase 3B1	HS3ST3B1	Q9Y662	0.002587	-0.07112
seq.7787.25	7787-25	Leukocyte immunoglobulin-like receptor subfamily A member 5	LILRA5	A6NI73	0.002587	-0.10127
seq.13450.49	13450-49	Ubiquitin carboxyl-terminal hydrolase 8	USP8	P40818	0.002636	0.511232
seq.13615.60	13615-60	Cytoplasmic protein NCK2	NCK2	O43639	0.002636	0.625065
seq.17712.7	17712-7	Isopentenyl-diphosphate Delta-isomerase 1	IDI1	Q13907	0.002636	1.027449
seq.24277.22	24277-22	Copine-7	CPNE7	Q9UBL6	0.002636	0.310294
seq.7204.1	7204-1	Carcinoembryonic antigen-related cell adhesion molecule 21	CEACAM21	Q3KPI0	0.002636	-0.10422
seq.8445.54	8445-54	Melittin_VESMG	MELT	P68408	0.002636	-0.13758
seq.12532.28	12532-28	Ubiquitin-conjugating enzyme E2 R1	CDC34	P49427	0.00286	0.387081
seq.7161.25	7161-25	GDH/6PGL endoplasmic bifunctional protein	H6PD	O95479	0.00286	-0.31565
seq.9829.91	9829-91	Bile salt sulfotransferase	SULT2A1	Q06520	0.00286	-0.82975
seq.11607.15	11607-15	Bromodomain-containing protein 1	BRD1	O95696	0.00286	-0.10658
seq.13393.46	13393-46	Derlin-1	DERL1	Q9BUN8	0.00286	0.404277
seq.9078.207	9078-207	Beta-1-syntrophin	SNTB1	Q13884	0.00286	0.143161
seq.18411.83	18411-83	ADP-ribosylation factor-like protein 15	ARL15	Q9NXU5	0.002993	0.159276
seq.10848.137	10848-137	Butyrophilin-like protein 3	BTNL3	Q6UXE8	0.002993	-0.0996
seq.9245.1	9245-1	Killer cell immunoglobulin-like receptor 2DL4	KIR2DL4	Q99706	0.002993	-0.10651

seq.11104.13	11104-13	Chitinase-3-like protein 1	CHI3L1	P36222	0.002993	0.499882
seq.20181.17	20181-17	Integrin alpha-11	ITGA11	Q9UKX5	0.002993	-0.14114
seq.5626.20	5626-20	Chymotrypsin-C	CTRC	Q99895	0.002993	-0.11235
seq.12385.4	12385-4	Calpain-3	CAPN3	P20807	0.002993	0.195377
seq.13256.21	13256-21	SH3 and multiple ankyrin repeat domains protein 1	SHANK1	Q9Y566	0.002993	-0.10655
seq.13992.12	13992-12	Vesicle-fusing ATPase	NSF	P46459	0.002993	0.435079
seq.15433.4	15433-4	Proto-oncogene tyrosine-protein kinase Src	SRC	P12931	0.002993	0.982782
seq.17850.42	17850-42	Krueppel-like factor 4	KLF4	O43474	0.002993	-0.16357
seq.18817.50	18817-50	Chromobox protein homolog 1	CBX1	P83916	0.002993	0.614273
seq.2211.9	2211-9	Metalloproteinase inhibitor 1	TIMP1	P01033	0.002993	0.322883
seq.22385.2	22385-2	PWWP domain-containing protein MUM1	PWWP3A	Q2TAK8	0.002993	0.221202
seq.23342.4	23342-4	Arrestin domain-containing protein 5	ARRDC5	A6NEK1	0.002993	0.20806
seq.8858.21	8858-21	Basic leucine zipper transcriptional factor ATF-like 3	BATF3	Q9NR55	0.002993	-0.1311
seq.9957.9	9957-9	E3 ubiquitin-protein ligase RNF114	RNF114	Q9Y508	0.002993	0.736763
seq.10364.6	10364-6	Mothers against decapentaplegic homolog 2	SMAD2	Q15796	0.002993	0.240774
seq.10870.32	10870-32	Spastin:Microtubule interacting and trafficking domain	SPAST	Q9UBP0	0.002993	0.635486
seq.11360.39	11360-39	Ribonucleoside-diphosphate reductase large subunit	RRM1	P23921	0.002993	0.991122
seq.11664.32	11664-32	ADP-ribosylation factor GTPase-activating protein 2	ARFGAP2	Q8N6H7	0.002993	0.710513
seq.11955.1	11955-1	Rho GTPase-activating protein 1:Rho-GTPase activating protein domain	ARHGAP1	Q07960	0.002993	0.78079
seq.12616.45	12616-45	Nuclear receptor-binding protein	NRBP1	Q9UHY1	0.002993	0.34172
seq.12661.44	12661-44	Gamma-aminobutyric acid receptor-associated protein-like 1	GABARAPL1	Q9H0R8	0.002993	0.68383
seq.13632.10	13632-10	Zyxin	ZYX	Q15942	0.002993	0.557881
seq.14623.26	14623-26	Small ubiquitin-related modifier 3	SUMO3	P55854	0.002993	0.711899
seq.15370.5	15370-5	BolA-like protein 1	BOLA1	Q9Y3E2	0.002993	0.558264
seq.15472.16	15472-16	Low-density lipoprotein receptor-related protein 11	LRP11	Q86VZ4	0.002993	0.564529
seq.16863.47	16863-47	N-acylneuraminate-9-phosphatase	NANP	Q8TBE9	0.002993	-0.09921
seq.16882.27	16882-27	14 kDa phosphohistidine phosphatase	PHPT1	Q9NRX4	0.002993	0.7233
seq.17404.5	17404-5	ADP-ribosylation factor-like protein 5B	ARL5B	Q96KC2	0.002993	0.614511
seq.17758.79	17758-79	L-xylulose reductase	DCXR	Q7Z4W1	0.002993	0.437852
seq.18204.1	18204-1	Transcription elongation factor A protein 1	TCEA1	P23193	0.002993	0.652334
seq.18307.71	18307-71	Inorganic pyrophosphatase 2, mitochondrial	PPA2	Q9H2U2	0.002993	0.657458
seq.18316.75	18316-75	Neuronal-specific septin-3	SEPTIN3	Q9UH03	0.002993	-0.07896
seq.19229.92	19229-92	Homer protein homolog 1	HOMER1	Q86YM7	0.002993	0.645056
seq.19233.75	19233-75	Copper transport protein ATOX1	ATOX1	O00244	0.002993	0.679405
seq.19250.50	19250-50	C-Myc-binding protein	MYCBP	Q99417	0.002993	0.660033
seq.19280.29	19280-29	Ubiquitin-conjugating enzyme E2 D3	UBE2D3	P61077	0.002993	0.248501
seq.20243.26	20243-26	Profilin-1	PFN1	P07737	0.002993	0.584058
seq.20401.19	20401-19	Protein BUD31 homolog	BUD31	P41223	0.002993	0.334994
seq.21235.11	21235-11	Importin subunit alpha-6	KPNA5	O15131	0.002993	0.877826
seq.21567.214	21567-214	Complement C1q tumor necrosis factor-related protein 4	C1QTNF4	Q9BXJ3	0.002993	-0.11441
seq.22049.24	22049-24	14-3-3 protein eta	YWHAH	Q04917	0.002993	0.761927
seq.23350.66	23350-66	Protein FAM118B	FAM118B	Q9BPY3	0.002993	0.545092
seq.23402.147	23402-147	Ganglioside-induced differentiation-associated protein 1-like 1	GDAP1L1	Q96MZ0	0.002993	0.455507
seq.23586.32	23586-32	Cancer/testis antigen 1B	CTAG1A	P78358	0.002993	-0.11416
seq.23672.10	23672-10	26S proteasome non-ATPase regulatory subunit 6	PSMD6	Q15008	0.002993	0.317941
seq.24656.6	24656-6	Sesquipedalian-2	PHETA2	Q6ICB4	0.002993	-0.11015
seq.24725.4	24725-4	COP9 signalosome complex subunit 5	COPS5	Q92905	0.002993	0.768675
seq.24941.14	24941-14	Clustered mitochondria protein homolog	CLUH	O75153	0.002993	0.16041
seq.24973.11	24973-11	MICAL-like protein 1	MICALL1	Q8N3F8	0.002993	0.31045
seq.25055.56	25055-56	Oxysterol-binding protein 1	OSBP	P22059	0.002993	0.383141
seq.25459.3	25459-3	O-acetyl-ADP-ribose deacetylase MACROD2	MACROD2	A1Z1Q3	0.002993	0.716113
seq.2609.59	2609-59	Cystatin-C	CST3	P01034	0.002993	0.764177
seq.3298.52	3298-52	Contactin-4	CNTN4	Q8IWV2	0.002993	0.202989
seq.3373.5	3373-5	Granzyme H	GZMH	P20718	0.002993	-0.1224
seq.3593.72	3593-72	Caspase-3	CASP3	P42574	0.002993	0.956525
seq.4124.24	4124-24	Heat shock 70 kDa protein 1A	HSPA1A	P0DMV8	0.002993	0.210459
seq.4224.7	4224-7	Heterogeneous nuclear ribonucleoprotein Q	SYNCRIP	O60506	0.002993	0.228081
seq.4911.49	4911-49	Glutathione S-transferase P	GSTP1	P09211	0.002993	0.993236
seq.4976.57	4976-57	Adapter molecule crk	CRK	P46108	0.002993	0.597151
seq.5722.78	5722-78	Lysosomal Pro-X carboxypeptidase	PRCP	P42785	0.002993	0.823104
seq.5903.91	5903-91	Heat shock cognate 71 kDa protein	HSPA8	P11142	0.002993	0.337485
seq.6504.65	6504-65	Lysyl oxidase homolog 2	LOXL2	Q9Y4K0	0.002993	0.268817
seq.9180.6	9180-6	Interferon gamma receptor 2:Extracellular domain	IFNGR2	P38484	0.002993	-0.15052
seq.9294.45	9294-45	Microfibrillar-associated protein 2	MFAP2	P55001	0.002993	-0.90275
seq.9468.8	9468-8	Vesicular integral-membrane protein VIP36	LMAN2	Q12907	0.002993	0.651128
seq.9934.29	9934-29	Phosphatidylinositol transfer protein alpha isoform	PITPNA	Q00169	0.002993	0.607663
seq.7059.14	7059-14	Leukocyte immunoglobulin-like receptor subfamily A member 6	LILRA6	Q6PI73	0.003137	-0.17845
seq.8248.222	8248-222	Sialic acid-binding Ig-like lectin 14	SIGLEC14	Q08ET2	0.003144	-0.14022
seq.10716.35	10716-35	26S proteasome non-ATPase regulatory subunit 5	PSMD5	Q16401	0.003144	-0.09915
seq.13985.12	13985-12	E3 ubiquitin-protein ligase SMURF2	SMURF2	Q9HAU4	0.003144	0.193704
seq.16915.153	16915-153	Semaphorin-4A	SEMA4A	Q9H3S1	0.003144	-0.12345
seq.21763.46	21763-46	Ubiquitin carboxyl-terminal hydrolase 4	USP4	Q13107	0.003144	0.352715
seq.23352.9	23352-9	Dysbindin	DTNBP1	Q96EV8	0.003354	-0.11798
seq.21897.4	21897-4	IL-17/IL-17F	IL17A|IL17F	Q16552|Q9 6PD4	0.003354	-0.06132
seq.25278.41	25278-41	Rho GTPase-activating protein 6	ARHGAP6	O43182	0.003354	-0.14404
seq.3195.50	3195-50	Granulysin	GNLY	P22749	0.003354	-0.1567
seq.11566.48	11566-48	Keratin, type II cytoskeletal 72	KRT72	Q14CN4	0.003354	-0.11445
seq.13733.5	13733-5	Interleukin-12 subunit beta	IL12B	P29460	0.003354	-0.14403
seq.2730.58	2730-58	MHC class I polypeptide-related sequence A	MICA	Q29983	0.003354	-0.33232
seq.5605.77	5605-77	Beta-1,3-N-acetylglucosaminyltransferase manic fringe	MFNG	O00587	0.003354	0.331876
seq.11562.9	11562-9	DNA polymerase epsilon subunit 2	POLE2	P56282	0.003354	-0.11555
seq.18191.23	18191-23	Ran-specific GTPase-activating protein	RANBP1	P43487	0.003354	0.207624
seq.20389.36	20389-36	Mitochondrial inner membrane protease subunit 2	IMMP2L	Q96T52	0.003354	-0.16159
seq.20441.35	20441-35	DNA-directed RNA polymerases I, II, and III subunit RPABC1	POLR2E	P19388	0.003354	0.636465
seq.20586.18	20586-18	Contactin-3	CNTN3	Q9P232	0.003354	-0.14535

Disease Models & Mechanisms • Supplementary information

seq.25118.45	25118-45	Protein bicaudal D homolog 1	BICD1	Q96G01	0.003354	0.244518
seq.25413.80	25413-80	G antigen 2	GAGE2B	Q13066	0.003354	0.219315
seq.3311.27	3311-27	Low affinity immunoglobulin gamma Fc region receptor III-B	FCGR3B	O75015	0.003354	-0.11463
seq.7193.98	7193-98	Protocadherin alpha-C1	PCDHAC1	Q9H158	0.003354	-0.09504
seq.8772.5	8772-5	Ephrin-B2:Cytoplasmic domain	EFNB2	P52799	0.003354	-0.10603
seq.9017.58	9017-58	Lactase-phlorizin hydrolase	LCT	P09848	0.003354	-0.11932
seq.9110.2	9110-2	Synaptotagmin-17	SYT17	Q9BSW7	0.003354	-0.12242
seq.10612.18	10612-18	Procollagen-lysine,2-oxoglutarate 5-dioxygenase 3	PLOD3	O60568	0.00349	1.019308
seq.11428.31	11428-31	PDZ and LIM domain protein 1	PDLIM1	O00151	0.00349	0.454914
seq.11672.17	11672-17	Kinesin-like protein KIF16B	KIF16B	Q96L93	0.00349	0.238605
seq.11683.19	11683-19	ADP-ribosylation factor-binding protein GGA3	GGA3	Q9NZ52	0.00349	0.481505
seq.12414.31	12414-31	14-3-3 protein beta/alpha	YWHAB	P31946	0.00349	0.606331
seq.12469.19	12469-19	Microtubule-associated protein RP/EB family member 1	MAPRE1	Q15691	0.00349	0.74041
seq.12585.39	12585-39	DNA excision repair protein ERCC-1	ERCC1	P07992	0.00349	0.299758
seq.12720.71	12720-71	Ubiquilin-4	UBQLN4	Q9NRR5	0.00349	0.49195
seq.12775.6	12775-6	High mobility group protein B3	HMGB3	O15347	0.00349	0.507075
seq.14271.23	14271-23	Ras-related protein Rab-6B	RAB6B	Q9NRW1	0.00349	0.435574
seq.15324.58	15324-58	Ferritin light chain	FTL	P02792	0.00349	0.616724
seq.15336.7	15336-7	Selenoprotein M	SELENOM	Q8WWX9	0.00349	0.273712
seq.16288.17	16288-17	Ephrin type-A receptor 4	EPHA4	P54764	0.00349	0.899644
seq.17751.68	17751-68	Beta-crystallin B1	CRYBB1	P53674	0.00349	3.132124
seq.18387.7	18387-7	Hsc70-interacting protein	ST13	P50502	0.00349	0.731194
seq.18897.31	18897-31	Histone deacetylase 2	HDAC2	Q92769	0.00349	0.743965
seq.19111.10	19111-10	NEDD8-conjugating enzyme Ubc12	UBE2M	P61081	0.00349	0.468394
seq.19131.184	19131-184	Nucleolysin TIAR	TIAL1	Q01085	0.00349	0.42049
seq.19383.131	19383-131	Cyclin-dependent kinase 2-interacting protein	CINP	Q9BW66	0.00349	0.298908
seq.19560.23	19560-23	Plexin-A4	PLXNA4	Q9HCM2	0.00349	-0.21177
seq.21110.5	21110-5	Ubiquitin-conjugating enzyme E2 Z	UBE2Z	Q9H832	0.00349	0.524296
seq.21126.27	21126-27	Glutamine--fructose-6-phosphate aminotransferase [isomerizing] 1	GFPT1	Q06210	0.00349	0.522907
seq.21839.3	21839-3	POU domain class 2-associating factor 1	POU2AF1	Q16633	0.00349	0.415936
seq.22488.17	22488-17	Krueppel-like factor 9	KLF9	Q13886	0.00349	0.478362
seq.22585.5	22585-5	Frizzled-4	FZD4	Q9ULV1	0.00349	0.154761
seq.22808.59	22808-59	Thyroid hormone receptor beta	THRB	P10828	0.00349	0.512574
seq.23272.8	23272-8	Protein DPCD	DPCD	Q9BVM2	0.00349	0.722732
seq.23546.9	23546-9	Hydroxyacylglutathione hydrolase-like protein	HAGHL	Q6PII5	0.00349	0.43332
seq.24455.2	24455-2	Arf-GAP with SH3 domain, ANK repeat and PH domain-containing protein 3	ASAP3	Q8TDY4	0.00349	0.335779
seq.25308.8	25308-8	LIM domain kinase 1	LIMK1	P53667	0.00349	0.218746
seq.3435.53	3435-53	Fibronectin Fragment 4	FN1	P02751	0.00349	1.041398
seq.4131.72	4131-72	Fibronectin	FN1	P02751	0.00349	1.554126
seq.4471.50	4471-50	Protein-glutamine gamma-glutamyltransferase E	TGM3	Q08188	0.00349	1.112094
seq.4712.28	4712-28	Apolipoprotein D	APOD	P05090	0.00349	0.390458
seq.4960.72	4960-72	Annexin A1	ANXA1	P04083	0.00349	1.133964
seq.5315.22	5315-22	Troponin T, cardiac muscle	TNNT2	P45379	0.00349	0.747709
seq.6425.87	6425-87	Matrix metalloproteinase-19	MMP19	Q99542	0.00349	0.695462
seq.7838.27	7838-27	Submaxillary gland androgen-regulated protein 3A	SMR3A	Q99954	0.00349	-0.0843
seq.7980.72	7980-72	N-acetyllactosaminide beta-1,3-N-acetylglucosaminyltransferase 2	B3GNT2	Q9NY97	0.00349	0.50958
seq.8252.2	8252-2	Palmitoleoyl-protein carboxylesterase NOTUM	NOTUM	Q6P988	0.00349	0.292115
seq.8409.3	8409-3	R-spondin-2	RSPO2	Q6UXX9	0.00349	-0.10053
seq.9360.33	9360-33	EGF-like repeat and discoidin I-like domain-containing protein 3	EDIL3	O43854	0.00349	2.038778
seq.12573.80	12573-80	Tripartite motif-containing protein 3	TRIM3	O75382	0.00349	-0.13154
seq.22569.55	22569-55	TGF-beta receptor type-1	TGFBR1	P36897	0.00349	-0.12153
seq.24648.8	24648-8	Histone RNA hairpin-binding protein	SLBP	Q14493	0.00349	0.345547
seq.24957.6	24957-6	Espin	None	B1AK53	0.00349	-0.12927
seq.9971.5	9971-5	CUB and sushi domain-containing protein 2	CSMD2	Q7Z408	0.00349	0.351877
seq.6546.41	6546-41	Dickkopf-related protein 2	DKK2	Q9UBU2	0.003749	-0.1266
seq.15608.5	15608-5	Ribosomal protein S6 kinase beta-1	RPS6KB1	P23443	0.003749	-0.07669
seq.12668.7	12668-7	Vacuolar protein sorting-associated protein 4B	VPS4B	O75351	0.003749	0.197809
seq.21770.18	21770-18	Galactosylgalactosylxylosylprotein 3-beta-glucuronosyltransferase 1	B3GAT1	Q9P2W7	0.003749	-0.1481
seq.3299.29	3299-29	Contactin-5	CNTN5	O94779	0.003749	-0.13609
seq.11178.21	11178-21	Sushi, von Willebrand factor type A, EGF and pentraxin domain-containing protein 1:EGF-like domains 4-6	SVEP1	Q4LDE5	0.003749	-0.19373
seq.13496.19	13496-19	Hydroxymethylglutaryl-CoA synthase, cytoplasmic	HMGCS1	Q01581	0.003749	1.371002
seq.14039.33	14039-33	Kallikrein-5	KLK5	Q9Y337	0.003749	-0.11629
seq.15436.40	15436-40	Receptor activity-modifying protein 1	RAMP1	O60894	0.003749	-0.08948
seq.15471.29	15471-29	Pancreatic lipase-related protein 2	PNLIPRP2	P54317	0.003749	0.24323
seq.23038.63	23038-63	Netrin receptor UNC5B	UNC5B	Q8IZJ1	0.003749	0.10175
seq.2879.9	2879-9	Alpha-1-antichymotrypsin	SERPINA3	P01011	0.003749	1.063409
seq.3489.9	3489-9	Ciliary neurotrophic factor	CNTF	P26441	0.003749	-0.12751
seq.4534.10	4534-10	Brain-specific serine protease 4	PRSS22	Q9GZN4	0.003749	-0.16537
seq.7951.146	7951-146	Fc receptor-like protein 2	FCRL2	Q96LA5	0.003749	-0.1227
seq.9183.7	9183-7	Interferon alpha/beta receptor 1	IFNAR1	P17181	0.004037	-0.10254
seq.7778.104	7778-104	Caspase recruitment domain-containing protein 19	CARD19	Q96LW7	0.004037	-0.09023
seq.14123.34	14123-34	V-type immunoglobulin domain-containing suppressor of T-cell activation:Extracellular domain	VSIR	Q9H7M9	0.004037	0.130302
seq.17138.8	17138-8	Glutathione S-transferase A1	GSTA1	P08263	0.004037	-0.0811
seq.17828.3	17828-3	Protein S100-A14	S100A14	Q9HCY8	0.004037	-0.09241
seq.23553.1	23553-1	ABI gene family member 3	ABI3	Q9P2A4	0.004037	-0.13248
seq.24013.6	24013-6	Metabotropic glutamate receptor 4	GRM4	Q14833	0.004037	-0.15836
seq.4707.50	4707-50	14-3-3 protein eta	YWHAH	Q04917	0.004037	0.550058
seq.10379.19	10379-19	A disintegrin and metalloproteinase with thrombospondin motifs 1_MOUSE	Adamts1	P97857	0.004037	-1.19289
seq.10557.6	10557-6	Testis-expressed sequence 29 protein	TEX29	Q8N6K0	0.004037	0.242871
seq.11606.22	11606-22	DnaJ homolog subfamily B member 6	DNAJB6	O75190	0.004037	-0.09949

Disease Models & Mechanisms • Supplementary information

seq.12417.46	12417-46	EKC/KEOPS complex subunit TPRKB	TPRKB	Q9Y3C4	0.004037	0.511326
seq.12659.13	12659-13	Obg-like ATPase 1	OLA1	Q9NTK5	0.004037	0.759373
seq.13488.3	13488-3	Arfaptin-1	ARFIP1	P53367	0.004037	0.216291
seq.13693.5	13693-5	Cerebral dopamine neurotrophic factor	CDNF	Q49AH0	0.004037	-0.08046
seq.14005.2	14005-2	Chromodomain-helicase-DNA-binding protein 7	CHD7	Q9P2D1	0.004037	-0.10016
seq.14083.25	14083-25	Selenide, water dikinase 1	SEPHS1	P49903	0.004037	0.701109
seq.14107.1	14107-1	5-formyltetrahydrofolate cyclo-ligase	MTHFS	P49914	0.004037	0.706691
seq.14283.12	14283-12	Ras-related protein Rab-14	RAB14	P61106	0.004037	0.238531
seq.15314.49	15314-49	Chloride intracellular channel protein 4	CLIC4	Q9Y696	0.004037	0.461017
seq.15322.35	15322-35	Death domain-containing protein CRADD	CRADD	P78560	0.004037	0.578467
seq.15594.47	15594-47	Serine protease HTRA1	HTRA1	Q92743	0.004037	0.826076
seq.17140.57	17140-57	Platelet-derived growth factor D	PDGFD	Q9GZP0	0.004037	-0.52288
seq.17372.5	17372-5	Eukaryotic translation initiation factor 4E-binding protein 1	EIF4EBP1	Q13541	0.004037	0.196268
seq.17691.1	17691-1	Tripeptidyl-peptidase 1	TPP1	O14773	0.004037	0.501575
seq.18165.181	18165-181	Ubiquitin-conjugating enzyme E2 variant 2	UBE2V2	Q15819	0.004037	0.29559
seq.18235.16	18235-16	Glycerol-3-phosphate phosphatase	PGP	A6NDG6	0.004037	0.481684
seq.18415.16	18415-16	ADP-ribosylation factor-like protein 6	ARL6	Q9H0F7	0.004037	0.346302
seq.18883.4	18883-4	Destrin	DSTN	P60981	0.004037	0.181937
seq.19620.16	19620-16	Disks large homolog 2	DLG2	Q15700	0.004037	0.606188
seq.20054.28	20054-28	Cytoplasmic aconitate hydratase	ACO1	P21399	0.004037	0.347117
seq.20086.5	20086-5	Ras-related protein Rab-11B	RAB11B	Q15907	0.004037	0.789032
seq.20090.63	20090-63	Ubiquitin-conjugating enzyme E2 E3	UBE2E3	Q969T4	0.004037	0.693285
seq.20398.60	20398-60	Ribonuclease P protein subunit p20	POP7	O75817	0.004037	0.24147
seq.21128.2	21128-2	CDK2	CDK2	P24941	0.004037	0.559895
seq.21189.9	21189-9	Nucleotide-binding protein 1	NUBP1	P53384	0.004037	0.55531
seq.21437.77	21437-77	Methylated-DNA--protein-cysteine methyltransferase	MGMT	P16455	0.004037	0.85124
seq.21483.155	21483-155	cAMP-dependent protein kinase type I-alpha regulatory subunit	PRKAR1A	P10644	0.004037	0.531068
seq.22517.106	22517-106	E3 SUMO-protein ligase NSE2	NSMCE2	Q96MF7	0.004037	0.196474
seq.23176.17	23176-17	Beta-dystroglycan	DAG1	Q14118	0.004037	0.189197
seq.23547.21	23547-21	Transcription elongation factor A protein 3	TCEA3	O75764	0.004037	0.47835
seq.23560.154	23560-154	Centrosomal protein of 41 kDa	CEP41	Q9BYV8	0.004037	0.741696
seq.23615.4	23615-4	TRM112-like protein	TRMT112	Q9UI30	0.004037	0.481822
seq.24493.10	24493-10	Myotubularin-related protein 7	MTMR7	Q9Y216	0.004037	0.172454
seq.24979.17	24979-17	Eukaryotic translation initiation factor 3 subunit B	EIF3B	P55884	0.004037	0.788568
seq.25480.21	25480-21	Ubiquitin-associated domain-containing protein 1	UBAC1	Q9BSL1	0.004037	0.291841
seq.2620.4	2620-4	Interleukin-6 receptor subunit beta	IL6ST	P40189	0.004037	0.463367
seq.2741.22	2741-22	Sialic acid-binding Ig-like lectin 6	SIGLEC6	O43699	0.004037	-0.09946
seq.3364.76	3364-76	Cathepsin L2	CTSV	O60911	0.004037	-0.69654
seq.4992.49	4992-49	Granulins	GRN	P28799	0.004037	0.295196
seq.5638.23	5638-23	Procollagen galactosyltransferase 1	COLGALT1	Q8NBJ5	0.004037	0.762513
seq.5690.49	5690-49	Tuftelin	TUFT1	Q9NNX1	0.004037	0.344882
seq.5695.5	5695-5	BPI fold-containing family A member 2	BPIFA2	Q96DR5	0.004037	-0.11421
seq.5747.67	5747-67	Osteocalcin	BGLAP	P02818	0.004037	-0.12778
seq.6641.60	6641-60	PolyUbiquitin K48-linked	UBB	P0CG47	0.004037	0.589346
seq.7893.19	7893-19	Oxidized low-density lipoprotein receptor 1	OLR1	P78380	0.004037	-0.09215
seq.8991.115	8991-115	Fc receptor-like protein 4:Cytoplasmic domain	FCRL4	Q96PJ5	0.004037	0.267962
seq.9018.38	9018-38	Protocadherin-10:Extracellular domain	PCDH10	Q9P2E7	0.004037	0.614563
seq.9357.4	9357-4	Protein CREG1	CREG1	O75629	0.004037	0.361307
seq.9764.79	9764-79	Heterogeneous nuclear ribonucleoprotein F	HNRNPF	P52597	0.004037	0.868506
seq.13665.35	13665-35	Serine/threonine-protein phosphatase 2A regulatory subunit B'' subunit alpha	PPP2R3A	Q06190	0.004161	-0.0933
seq.15417.3	15417-3	Serpin B5	SERPINB5	P36952	0.004163	-0.10545
seq.22085.86	22085-86	Polycomb complex protein BMI-1	BMI1	P35226	0.004163	-0.08411
seq.2611.72	2611-72	Tyrosine-protein kinase receptor TYRO3	TYRO3	Q06418	0.004163	-0.16928
seq.12968.2	12968-2	Cysteine and glycine-rich protein 2	CSRP2	Q16527	0.004163	0.819001
seq.13688.2	13688-2	Calcyphosin-like protein	CAPSL	Q8WWF8	0.004163	-0.11855
seq.17200.50	17200-50	Killer cell immunoglobulin-like receptor 2DL1	KIR2DL1	P43626	0.004163	0.176315
seq.19294.26	19294-26	Cysteine-rich PDZ-binding protein	CRIPT	Q9P021	0.004163	0.235268
seq.20105.7	20105-7	Myosin light polypeptide 6	MYL6	P60660	0.004163	0.538593
seq.20447.11	20447-11	Homeobox protein TGIF2LY	TGIF2LY	Q8IUE0	0.004163	-0.13581
seq.8298.8	8298-8	Serine-rich single-pass membrane protein 1	SSMEM1	Q8WWF3	0.004163	0.257253
seq.6993.8	6993-8	5'-Nucleotidase	NT5E	P21589	0.004481	-0.1317
seq.9030.56	9030-56	Uncharacterized protein C17orf89	NDUFAF8	A1L188	0.004481	-0.08968
seq.10008.43	10008-43	Guanylyl cyclase-activating protein 1	GUCA1A	P43080	0.004481	-0.1141
seq.23384.19	23384-19	BTB/POZ domain-containing protein KCTD17	KCTD17	Q8N5Z5	0.004481	0.736974
seq.23570.87	23570-87	Zinc finger protein with KRAB and SCAN domains 7	ZKSCAN7	Q9P0L1	0.004481	-0.12301
seq.5028.59	5028-59	Scavenger receptor cysteine-rich type 1 protein M130	CD163	Q86VB7	0.004481	-0.11796
seq.7265.32	7265-32	Osteocrin	OSTN	P61366	0.004481	-0.11687
seq.15462.28	15462-28	T-cell surface glycoprotein CD8 alpha chain	CD8A	P01732	0.004481	-0.13819
seq.18926.7	18926-7	Proteasome subunit alpha type-3	PSMA3	P25788	0.004481	-0.14446
seq.19317.114	19317-114	Prostatic acid phosphatase	ACP3	P15309	0.004481	-0.11284
seq.4413.3	4413-3	Antileukoproteinase	SLPI	P03973	0.004481	-0.09851
seq.4979.34	4979-34	Dermatopontin	DPT	Q07507	0.004625	-0.14254
seq.11480.1	11480-1	Aldehyde dehydrogenase, dimeric NADP-preferring	ALDH3A1	P30838	0.004625	-0.12077
seq.13452.113	13452-113	Small integral membrane protein 13	SMIM13	P0DJ93	0.004625	-0.09537
seq.17454.15	17454-15	Epidermal growth factor-like protein 6	EGFL6	Q8IUX8	0.004625	0.136379
seq.18398.1	18398-1	3-oxo-5-beta-steroid 4-dehydrogenase	AKR1D1	P51857	0.004625	-0.12158
seq.20430.8	20430-8	C-type natriuretic peptide	NPPC	P23582	0.004625	-0.11197
seq.21533.51	21533-51	Lymphocyte-specific protein 1	LSP1	P33241	0.004625	-0.13311
seq.2500.2	2500-2	Angiopoietin-4	ANGPT4	Q9Y264	0.004625	-0.10349
seq.8107.12	8107-12	Transmembrane protein 234	TMEM234	Q8WY98	0.004625	-0.10544
seq.8899.75	8899-75	UDP-glucuronosyltransferase 1-8	UGT1A8	Q9HAW9	0.004625	-0.13445
seq.19347.37	19347-37	Carbonic anhydrase 12	CA12	O43570	0.004625	-0.1325
seq.23403.64	23403-64	Cdc42 effector protein 4	CDC42EP4	Q9H3Q1	0.004625	0.26397
seq.2558.51	2558-51	Beta-endorphin	POMC	P01189	0.004625	-0.10406
seq.3194.36	3194-36	Platelet glycoprotein VI	GP6	Q9HCN6	0.004625	-0.10662

seq.4440.15	4440-15	Fc receptor-like protein 3	FCRL3	Q96P31	0.004625	-0.11141
seq.10575.31	10575-31	Poly(U)-binding-splicing factor PUF60	PUF60	Q9UHX1	0.004625	0.594387
seq.11241.8	11241-8	Argininosuccinate lyase	ASL	P04424	0.004625	0.597267
seq.11530.37	11530-37	Porphobilinogen deaminase	HMBS	P08397	0.004625	0.583378
seq.11616.9	11616-9	Heat shock factor protein 1	HSF1	Q00613	0.004625	0.56659
seq.11626.7	11626-7	Ubiquitin-conjugating enzyme E2 variant 1	UBE2V1	Q13404	0.004625	0.5375
seq.12497.29	12497-29	Tudor-interacting repair regulator protein	NUDT16L1	Q9BRJ7	0.004625	0.677004
seq.12646.2	12646-2	Ribulose-phosphate 3-epimerase	RPE	Q96AT9	0.004625	0.228983
seq.14708.59	14708-59	Complement component C8 gamma chain	C8G	P07360	0.004625	0.417157
seq.15562.24	15562-24	Beta-glucuronidase	GUSB	P08236	0.004625	0.453032
seq.16890.37	16890-37	ADAMTS-like protein 1	ADAMTSL1	Q8N6G6	0.004625	-0.71956
seq.17231.1	17231-1	Plastin-2	LCP1	P13796	0.004625	0.137152
seq.17325.10	17325-10	Guanylate kinase	GUK1	Q16774	0.004625	0.598887
seq.17411.55	17411-55	Ubiquitin recognition factor in ER-associated degradation protein 1	UFD1	Q92890	0.004625	0.575207
seq.17755.5	17755-5	UDP-glucose 6-dehydrogenase	UGDH	O60701	0.004625	0.596117
seq.18308.30	18308-30	Syntaxin-binding protein 6	STXBP6	Q8NFX7	0.004625	0.300702
seq.18405.117	18405-117	AP-1 complex subunit sigma-2	AP1S2	P56377	0.004625	0.208799
seq.18824.7	18824-7	Eukaryotic initiation factor 4A-II	EIF4A2	Q14240	0.004625	0.735017
seq.19170.25	19170-25	DNA polymerase epsilon subunit 3	POLE3	Q9NRF9	0.004625	0.138334
seq.19238.12	19238-12	Glutamine synthetase	GLUL	P15104	0.004625	0.415294
seq.19341.36	19341-36	Acyl-CoA-binding domain-containing protein 6	ACBD6	Q9BR61	0.004625	0.750677
seq.21178.8	21178-8	Probable cytosolic iron-sulfur protein assembly protein CIAO1	CIAO1	O76071	0.004625	0.988864
seq.21249.115	21249-115	DNA fragmentation factor subunit alpha	DFFA	O00273	0.004625	0.408681
seq.21317.25	21317-25	NF-kappa-B inhibitor-interacting Ras-like protein 1	NKIRAS1	Q9NYS0	0.004625	0.176853
seq.21331.19	21331-19	B-cell CLL/lymphoma 7 protein family member A	BCL7A	Q4VC05	0.004625	0.478975
seq.21345.93	21345-93	Retinol dehydrogenase 12	RDH12	Q96NR8	0.004625	0.200359
seq.21382.70	21382-70	Zinc phosphodiesterase ELAC protein 1	ELAC1	Q9H777	0.004625	0.588347
seq.21537.33	21537-33	Selenocysteine lyase	SCLY	Q96I15	0.004625	0.497148
seq.22817.126	22817-126	E3 ubiquitin-protein ligase TRIM9	TRIM9	Q9C026	0.004625	0.176289
seq.23320.11	23320-11	Multivesicular body subunit 12B	MVB12B	Q9H7P6	0.004625	0.479052
seq.23408.1	23408-1	Sorting nexin 16	SNX16	P57768	0.004625	0.399988
seq.24496.26	24496-26	RILP-like protein 1	RILPL1	Q5EBL4	0.004625	0.784645
seq.24910.18	24910-18	Non-POU domain-containing octamer-binding protein	NONO	Q15233	0.004625	0.776201
seq.24983.119	24983-119	ELKS/RAB6-interacting/CAST family member 1	ERC1	Q8IUD2	0.004625	0.259516
seq.25284.47	25284-47	Ubiquitin-like modifier-activating enzyme 6	UBA6	A0AVT1	0.004625	0.889437
seq.25299.11	25299-11	Nuclear receptor coactivator 7	NCOA7	Q8NI08	0.004625	0.194412
seq.2835.1	2835-1	X-ray repair cross-complementing protein 6	XRCC6	P12956	0.004625	0.618061
seq.3122.6	3122-6	Diablo homolog, mitochondrial	DIABLO	Q9NR28	0.004625	0.092806
seq.3293.2	3293-2	CD5 antigen-like	CD5L	O43866	0.004625	-0.11584
seq.4272.46	4272-46	Glucose-6-phosphate isomerase	GPI	P06744	0.004625	0.685756
seq.4474.19	4474-19	Ubiquitin	RPS27A	P62979	0.004625	0.397584
seq.4834.61	4834-61	Ephrin type-A receptor 2	EPHA2	P29317	0.004625	0.604024
seq.5007.1	5007-1	Mitogen-activated protein kinase 14	MAPK14	Q16539	0.004625	0.554299
seq.5091.28	5091-28	Leukocyte immunoglobulin-like receptor subfamily B member 2	LILRB2	Q8N423	0.004625	-0.10094
seq.5467.15	5467-15	Heat shock protein HSP 90-beta	HSP90AB1	P08238	0.004625	0.506957
seq.5509.7	5509-7	Epidermal growth factor:Extracellular domain	EGF	P01133	0.004625	0.144091
seq.5599.88	5599-88	Prenylcysteine oxidase-like	PCYOX1L	Q8NBM8	0.004625	0.585679
seq.6207.10	6207-10	Prosaposin	PSAP	P07602	0.004625	0.427666
seq.8925.25	8925-25	Ribonucleoside-diphosphate reductase subunit M2 B	RRM2B	Q7LG56	0.004625	0.603515
seq.9111.40	9111-40	Junctophilin-4	JPH4	Q96JJ6	0.004625	-0.08355
seq.9836.20	9836-20	Deoxycytidine kinase	DCK	P27707	0.004625	0.191049
seq.9937.7	9937-7	Gap junction alpha-1 protein	GJA1	P17302	0.004625	-0.06502
seq.25433.25	25433-25	Putative methyltransferase NSUN6	NSUN6	Q8TEA1	0.004981	-0.12083
seq.24695.8	24695-8	Pleckstrin homology domain-containing family B member 1	PLEKHB1	Q9UF11	0.004981	-0.09806
seq.6245.4	6245-4	Nectin-2	NECTIN2	Q92692	0.004985	0.143306
seq.12394.53	12394-53	Transmembrane protein C16orf54	C16orf54	Q6UWD8	0.005327	-0.19754
seq.5741.55	5741-55	Eosinophil cationic protein	RNASE3	P12724	0.005327	-0.14846
seq.11388.75	11388-75	WAP four-disulfide core domain protein 2	WFDC2	Q14508	0.005327	-0.11454
seq.17357.33	17357-33	O-phosphoseryl-tRNA(Sec) selenium transferase	SEPSECS	Q9HD40	0.005327	-0.13196
seq.3206.4	3206-4	Lymphatic vessel endothelial hyaluronic acid receptor 1	LYVE1	Q9Y5Y7	0.005327	-0.17226
seq.7822.11	7822-11	HRAS-like suppressor 2	PLAAT2	Q9NWW9	0.005327	-0.13784
seq.8052.115	8052-115	Neuroligin-1	NLGN1	Q8N2Q7	0.005327	0.229767
seq.19639.53	19639-53	Islet amyloid polypeptide	IAPP	P10997	0.005327	-0.15342
seq.9451.20	9451-20	Uromodulin	UMOD	P07911	0.005327	-0.15257
seq.12378.71	12378-71	Tapasin	TAPBP	O15533	0.005327	-0.10536
seq.12434.25	12434-25	IST1 homolog	IST1	P53990	0.005327	0.26933
seq.21971.47	21971-47	Friend leukemia integration 1 transcription factor	FLI1	Q01543	0.005327	0.168493
seq.22783.40	22783-40	Transcription factor PU.1	SPI1	P17947	0.005327	-0.13725
seq.2449.1	2449-1	Calcium-dependent phospholipase A2	PLA2G5	P39877	0.005327	-0.1169
seq.3082.9	3082-9	NKG2D ligand 2	ULBP2	Q9BZM5	0.005327	-0.09439
seq.5098.79	5098-79	Killer cell lectin-like receptor subfamily F member 1	KLRF1	Q9NZS2	0.005327	-0.10045
seq.9302.90	9302-90	Growth arrest and DNA damage-inducible proteins-interacting protein 1	GADD45GIP1	Q8TAE8	0.005327	-0.12918
seq.9963.19	9963-19	Protocadherin beta-10	PCDHB10	Q9UN67	0.005327	0.104824
seq.10569.28	10569-28	Microfibrillar-associated protein 2	MFAP2	P55001	0.005358	-0.78215
seq.10583.1	10583-1	Transmembrane protein 108	TMEM108	Q6UXF1	0.005358	-0.12768
seq.10781.19	10781-19	C-type lectin domain family 4 member G	CLEC4G	Q6UXB4	0.005358	0.550516
seq.11450.110	11450-110	Protein disulfide-isomerase	P4HB	P07237	0.005358	0.934241
seq.11454.87	11454-87	Eukaryotic translation initiation factor 3 subunit G	EIF3G	O75821	0.005358	0.783445
seq.13719.19	13719-19	Serine/threonine-protein kinase PAK 4	PAK4	O96013	0.005358	0.457908
seq.14156.33	14156-33	14-3-3 protein beta/alpha	YWHAB	P31946	0.005358	0.582768
seq.14309.8	14309-8	Heterogeneous nuclear ribonucleoprotein H	HNRNPH1	P31943	0.005358	0.323806
seq.14597.5	14597-5	Semaphorin-6C, cytoplasmic	SEMA6C	Q9H3T2	0.005358	-0.13206
seq.16758.96	16758-96	Hepatoma-derived growth factor	HDGF	P51858	0.005358	0.561934
seq.16907.3	16907-3	Cell adhesion molecule 2	CADM2	Q8N3J6	0.005358	-0.10987
seq.18178.13	18178-13	Serine--tRNA ligase, cytoplasmic	SARS1	P49591	0.005358	0.421146

Disease Models & Mechanisms • Supplementary information

seq.18193.165	18193-165	Mortality factor 4-like protein 1	MORF4L1	Q9UBU8	0.005358	0.25029
seq.18300.39	18300-39	Transcriptional activator protein Pur-beta	PURB	Q96QR8	0.005358	0.454342
seq.18373.13	18373-13	Radixin	RDX	P35241	0.005358	0.586229
seq.19121.3	19121-3	Ubiquilin-2	UBQLN2	Q9UHD9	0.005358	0.519196
seq.19152.4	19152-4	Protein phosphatase inhibitor 2	PPP1R2	P41236	0.005358	0.349587
seq.20083.1	20083-1	Ras-related protein Rab-6A	RAB6A	P20340	0.005358	0.705258
seq.20378.110	20378-110	Leucine zipper transcription factor-like protein 1	LZTFL1	Q9NQ48	0.005358	0.427524
seq.20576.71	20576-71	Frizzled-7	FZD7	O75084	0.005358	0.517484
seq.21289.36	21289-36	Sperm-associated antigen 7	SPAG7	O75391	0.005358	0.512877
seq.21392.15	21392-15	Melanoma-associated antigen D1	MAGED1	Q9Y5V3	0.005358	0.263389
seq.21916.82	21916-82	Ubiquitin thioesterase otulin	OTULIN	Q96BN8	0.005358	0.299805
seq.24427.33	24427-33	Type II inositol-3,4-bisphosphate 4-phosphatase	INPP4B	O15327	0.005358	0.481289
seq.25287.7	25287-7	Eukaryotic translation initiation factor 4 gamma 1	EIF4G1	Q04637	0.005358	0.578516
seq.25921.3	25921-3	Inhibin a subunit	INHA	P05111	0.005358	0.523189
seq.2647.66	2647-66	Rab GDP dissociation inhibitor beta	GDI2	P50395	0.005358	0.510628
seq.2700.56	2700-56	Vitamin K-dependent protein S	PROS1	P07225	0.005358	1.0463
seq.3196.6	3196-6	Hyaluronan and proteoglycan link protein 1	HAPLN1	P10915	0.005358	2.00905
seq.3315.15	3315-15	Glypican-2	GPC2	Q8N158	0.005358	0.207709
seq.3486.58	3486-58	Fibroblast growth factor 1	FGF1	P05230	0.005358	2.073759
seq.4309.59	4309-59	Triosephosphate isomerase	TPI1	P60174	0.005358	0.516526
seq.4563.61	4563-61	1-phosphatidylinositol 4,5-bisphosphate phosphodiesterase gamma-1	PLCG1	P19174	0.005358	0.534007
seq.5125.6	5125-6	Sialic acid-binding Ig-like lectin 14	SIGLEC14	Q08ET2	0.005358	-0.12493
seq.5225.50	5225-50	Casein kinase II 2-alpha:2-beta heterotetramer	CSNK2A1|CSNK2B	P68400|P67870	0.005358	0.728375
seq.5238.26	5238-26	Peptidyl-prolyl cis-trans isomerase E	PPIE	Q9UNP9	0.005358	0.372315
seq.5437.63	5437-63	Fatty acid-binding protein, heart	FABP3	P05413	0.005358	0.544101
seq.5542.22	5542-22	Neuropilin-1	NRP1	O14786	0.005358	0.683822
seq.5618.50	5618-50	Protein FAM3B	FAM3B	P58499	0.005358	0.20946
seq.6290.3	6290-3	Urotensin-2B	UTS2B	Q765I0	0.005358	0.4344
seq.6568.18	6568-18	HEPACAM family member 2:Isoform 2, Extracellular domain	HEPACAM2	A8MVW5	0.005358	-0.10721
seq.6947.4	6947-4	Type 2 lactosamine alpha-2,3-sialyltransferase	ST3GAL6	Q9Y274	0.005358	0.556059
seq.7175.4	7175-4	Protocadherin gamma-A2	PCDHGA2	Q9Y5H1	0.005358	0.362655
seq.7970.315	7970-315	Ecto-ADP-ribosyltransferase 3	ART3	Q13508	0.005358	0.828489
seq.8016.19	8016-19	DnaJ homolog subfamily C member 4:N-term	DNAJC4	Q9NNZ3	0.005358	-0.11872
seq.8274.64	8274-64	Syntaxin-7	STX7	O15400	0.005358	0.440453
seq.8304.50	8304-50	Tumor necrosis factor receptor superfamily member 11B	TNFRSF11B	O00300	0.005358	0.943951
seq.8994.65	8994-65	SLAM family member 8	SLAMF8	Q9P0V8	0.005358	-0.09143
seq.9039.47	9039-47	Torsin-1A-interacting protein 1:Nuclear domain	TOR1AIP1	Q5JTV8	0.005358	0.789466
seq.9541.15	9541-15	Beta-1,3-galactosyltransferase 1	B3GALT1	Q9Y5Z6	0.005358	-0.1374
seq.9841.197	9841-197	Multifunctional protein ADE2	PAICS	P22234	0.005358	0.421273
seq.9999.1	9999-1	Interferon regulatory factor 6	IRF6	O14896	0.005358	0.434103
seq.9204.33	9204-33	Pro-opiomelanocortin	POMC	P01189	0.005456	-0.13297
seq.7130.4	7130-4	Leukocyte-associated immunoglobulin-like receptor 2	LAIR2	Q6ISS4	0.005514	-0.09044
seq.9227.15	9227-15	Programmed cell death protein 1	PDCD1	Q15116	0.005514	-0.13084
seq.11310.8	11310-8	Desmoglein-3	DSG3	P32926	0.005514	-0.11529
seq.5596.75	5596-75	T-cell surface glycoprotein CD5:Extracellular domain	CD5	P06127	0.005514	-0.13496
seq.13447.42	13447-42	Shadow of prion protein	SPRN	Q5BIV9	0.005514	0.149948
seq.17436.193	17436-193	Casein kinase I isoform gamma-2	CSNK1G2	P78368	0.005514	0.212343
seq.4437.56	4437-56	Ectonucleoside triphosphate diphosphohydrolase 5	ENTPD5	O75356	0.005514	0.182822
seq.15692.300	15692-300	Nodal homolog	NODAL	Q96S42	0.005871	-0.10118
seq.2665.26	2665-26	Tumor necrosis factor receptor superfamily member 17	TNFRSF17	Q02223	0.005871	-0.07399
seq.14102.6	14102-6	Granulysin	GNLY	P22749	0.005871	-0.10846
seq.9991.112	9991-112	EMILIN-3:region 2	EMILIN3	Q9NT22	0.005871	0.28284
seq.10816.150	10816-150	Paired immunoglobulin-like type 2 receptor alpha isoform FDF03-M14	PILRA	Q9UKJ1	0.005871	-0.0922
seq.12681.63	12681-63	Ubiquitin carboxyl-terminal hydrolase 21	USP21	Q9UK80	0.005871	0.14585
seq.13494.6	13494-6	Ceramide synthase 5	CERS5	Q8N5B7	0.005871	-0.13272
seq.20213.82	20213-82	Heat shock 70 kDa protein 6	HSPA6	P17066	0.005871	0.242418
seq.9728.4	9728-4	E3 ubiquitin-protein ligase CCNB1IP1	CCNB1IP1	Q9NPC3	0.005871	-0.0907
seq.10876.300	10876-300	BRCA1-associated ATM activator 1	BRAT1	Q6PJG6	0.005871	-0.1132
seq.12509.115	12509-115	COMM domain-containing protein 1	COMMD1	Q8N668	0.005871	0.103998
seq.20912.10	20912-10	Pseudouridine-5'-phosphatase	PUDP	Q08623	0.005871	0.514568
seq.2418.55	2418-55	Apolipoprotein E	APOE	P02649	0.005871	0.421666
seq.24707.6	24707-6	Uncharacterized protein CXorf38	CXorf38	Q8TB03	0.005871	0.251843
seq.3070.1	3070-1	Interleukin-2	IL2	P60568	0.005871	-0.14003
seq.3795.6	3795-6	Disintegrin and metalloproteinase domain-containing protein 9	ADAM9	Q13443	0.005871	0.304682
seq.3879.50	3879-50	Hsp90 co-chaperone Cdc37	CDC37	Q16543	0.005871	0.297033
seq.4842.62	4842-62	Glypican-3	GPC3	P51654	0.005871	0.467152
seq.5103.30	5103-30	Cell surface glycoprotein CD200 receptor 1:Isoform 4, Extracellular Domain	CD200R1	Q8TD46	0.005871	-0.13147
seq.5223.59	5223-59	Glucokinase regulatory protein	GCKR	Q14397	0.005871	-0.08673
seq.6527.1	6527-1	TLR4 interactor with leucine rich repeats:Extracellular domain	TRIL	Q7L0X0	0.005871	-0.1465
seq.9265.10	9265-10	Glioma pathogenesis-related protein 1	GLIPR1	P48060	0.005871	-0.14468
seq.9340.17	9340-17	Peptidyl-prolyl cis-trans isomerase FKBP14	FKBP14	Q9NWM8	0.005871	0.786776
seq.10010.10	10010-10	Beclin-1	BECN1	Q14457	0.006114	-0.11155
seq.12963.1	12963-1	Protein GPR107	GPR107	Q5VW38	0.006114	-0.17391
seq.19325.21	19325-21	Protein ABHD14B	ABHD14B	Q96IU4	0.006114	-0.10803
seq.5009.11	5009-11	Moesin	MSN	P26038	0.006114	-0.117
seq.9524.46	9524-46	Putative spermatogenesis-associated protein 31D4	SPATA31D4	Q6ZUB0	0.006114	0.096911
seq.10970.3	10970-3	Ecto-ADP-ribosyltransferase 3	ART3	Q13508	0.006114	0.50982
seq.10990.21	10990-21	Leucine-rich repeat serine/threonine-protein kinase 2	LRRK2	Q5S007	0.006114	0.155539
seq.11208.15	11208-15	N-acetylglucosamine-1-phosphodiester alpha-N-acetylglucosaminidase	NAGPA	Q9UK23	0.006114	-0.11828
seq.11657.86	11657-86	Suppressor of cytokine signaling 7	SOCS7	O14512	0.006114	-0.08233
seq.12016.60	12016-60	E3 ubiquitin-protein ligase CBL	CBL	P22681	0.006114	0.594399
seq.12466.7	12466-7	Heterogeneous nuclear ribonucleoprotein A1	HNRNPA1	P09651	0.006114	0.664761

seq.12522.6	12522-6	UV excision repair protein RAD23 homolog B	RAD23B	P54727	0.006114	0.717128
seq.12593.33	12593-33	p53 and DNA damage-regulated protein 1	PDRG1	Q9NUG6	0.006114	0.344359
seq.13629.25	13629-25	Cysteine protease ATG4B	ATG4B	Q9Y4P1	0.006114	0.398934
seq.13658.31	13658-31	Platelet-derived growth factor C	PDGFC	Q9NRA1	0.006114	0.230626
seq.14034.22	14034-22	Tumor-associated calcium signal transducer 2	TACSTD2	P09758	0.006114	-0.07738
seq.14151.4	14151-4	Ubiquitin-like protein ISG15	ISG15	P05161	0.006114	0.705445
seq.14203.3	14203-3	Annexin A7	ANXA7	P20073	0.006114	0.462591
seq.14713.46	14713-46	Azurocidin	AZU1	P20160	0.006114	-0.11356
seq.15524.30	15524-30	Phosphoglycerate mutase 2	PGAM2	P15259	0.006114	0.773195
seq.16926.44	16926-44	Alkaline phosphatase, tissue-nonspecific isozyme	ALPL	P05186	0.006114	0.445463
seq.17682.1	17682-1	Membrane cofactor protein	CD46	P15529	0.006114	0.323645
seq.17726.3	17726-3	GTP-binding protein SAR1a	SAR1A	Q9NR31	0.006114	0.376984
seq.17735.130	17735-130	Gamma-aminobutyric acid receptor-associated protein	GABARAP	O95166	0.006114	0.610717
seq.18243.9	18243-9	UMP-CMP kinase	CMPK1	P30085	0.006114	0.38271
seq.18416.3	18416-3	ADP-ribosylation factor-like protein 9	ARL9	Q6T311	0.006114	-0.10879
seq.19183.164	19183-164	60S ribosomal protein L12	RPL12	P30050	0.006114	0.915743
seq.19195.85	19195-85	40S ribosomal protein S5	RPS5	P46782	0.006114	0.719035
seq.20137.49	20137-49	Heterogeneous nuclear ribonucleoprotein A1	HNRNPA1	P09651	0.006114	0.645045
seq.21136.1	21136-1	Proteasome assembly chaperone 2	PSMG2	Q969U7	0.006114	0.310858
seq.21323.2	21323-2	RWD domain-containing protein 1	RWDD1	Q9H446	0.006114	0.593063
seq.21546.20	21546-20	Adhesion G-protein coupled receptor D1	ADGRD1	Q6QNK2	0.006114	0.247645
seq.21647.9	21647-9	Spermidine synthase	SRM	P19623	0.006114	0.553881
seq.21713.11	21713-11	Reelin	RELN	P78509	0.006114	0.455232
seq.21734.36	21734-36	C1GLT/C1GLC Complex	C1GALT1|C1GALT1C1	Q9NS00|Q96EU7	0.006114	-0.09209
seq.21747.114	21747-114	UBE2N (Ubc13)/Uev1a Complex	UBE2N|UBE2V1	P61088|Q13404	0.006114	0.543448
seq.21766.50	21766-50	N-sulphoglucosamine sulphohydrolase	SGSH	P51688	0.006114	0.41619
seq.22430.15	22430-15	N-chimaerin	CHN1	P15882	0.006114	0.714985
seq.22547.17	22547-17	RNA-binding motif, single-stranded-interacting protein 1	RBMS1	P29558	0.006114	0.33033
seq.22589.3	22589-3	Frizzled-2	FZD2	Q14332	0.006114	0.473627
seq.22793.110	22793-110	Transcription initiation factor IIA subunit 2	GTF2A2	P52657	0.006114	0.194811
seq.23307.7	23307-7	Clusterin-associated protein 1	CLUAP1	Q96AJ1	0.006114	0.393486
seq.23366.15	23366-15	Ubiquitin-like protein 7	UBL7	Q96S82	0.006114	0.585547
seq.24244.81	24244-81	Rab-interacting lysosomal protein	RILP	Q96NA2	0.006114	0.525457
seq.24908.19	24908-19	Amphiphysin	AMPH	P49418	0.006114	0.248611
seq.25451.39	25451-39	Transcriptional coactivator YAP1	YAP1	P46937	0.006114	0.61833
seq.2864.2	2864-2	Dual specificity mitogen-activated protein kinase kinase 1	MAP2K1	Q02750	0.006114	0.418138
seq.3026.5	3026-5	Calpastatin	CAST	P20810	0.006114	0.289172
seq.3143.3	3143-3	T-cell surface glycoprotein CD4	CD4	P01730	0.006114	-0.14796
seq.3350.53	3350-53	Calcium/calmodulin-dependent protein kinase type II subunit alpha	CAMK2A	Q9UQM7	0.006114	0.374804
seq.3416.2	3416-2	Tyrosine-protein kinase BTK	BTK	Q06187	0.006114	-0.09269
seq.3854.24	3854-24	Nascent polypeptide-associated complex subunit alpha	NACA	Q13765	0.006114	0.275539
seq.4706.17	4706-17	Protein 4.1	EPB41	P11171	0.006114	0.755103
seq.5030.52	5030-52	NAD-dependent protein deacetylase sirtuin-2	SIRT2	Q8IXJ6	0.006114	0.426973
seq.5731.1	5731-1	Serine protease inhibitor Kazal-type 6	SPINK6	Q6UWN8	0.006114	-0.08066
seq.6049.64	6049-64	Receptor-type tyrosine-protein phosphatase S	PTPRS	Q13332	0.006114	0.679142
seq.6373.54	6373-54	Protein delta homolog 1	DLK1	P80370	0.006114	0.562588
seq.6458.6	6458-6	Dolichyl-diphosphooligosaccharide--protein glycosyltransferase subunit 1:Lumenal domain	RPN1	P04843	0.006114	-0.07506
seq.8295.16	8295-16	Noelin-2	OLFM2	O95897	0.006114	0.796998
seq.8378.3	8378-3	Protein LDOC1	LDOC1	O95751	0.006114	-0.07616
seq.8811.24	8811-24	BMP and activin membrane-bound inhibitor homolog:Extracellular domain	BAMBI	Q13145	0.006114	0.118672
seq.5854.60	5854-60	Microtubule-associated protein tau	MAPT	P10636	0.006522	-0.17087
seq.10571.14	10571-14	Alpha-1,3-mannosyl-glycoprotein 2-beta-N-acetylglucosaminyltransferase	MGAT1	P26572	0.006522	-0.14413
seq.15446.25	15446-25	Neuregulin-1, sensory and motor neuron-derived factor isoform	NRG1	Q02297	0.006522	0.265179
seq.4880.21	4880-21	Growth/differentiation factor 2	GDF2	Q9UK05	0.006522	-0.05864
seq.5627.53	5627-53	Progonadoliberin-1	GNRH1	P01148	0.006522	-0.10664
seq.16923.20	16923-20	Profilin-2	PFN2	P35080	0.006522	-0.0724
seq.9369.174	9369-174	Leucine-rich repeat-containing protein 4C	LRRC4C	Q9HCJ2	0.006522	-0.1692
seq.12034.28	12034-28	Adenylyl cyclase-associated protein 1	CAP1	Q01518	0.006522	1.164269
seq.13518.5	13518-5	Arf-GAP with SH3 domain, ANK repeat and PH domain-containing protein 2	ASAP2	O43150	0.006522	0.516055
seq.2192.63	2192-63	C-C motif chemokine 27	CCL27	Q9Y4X3	0.006522	-0.1246
seq.22394.8	22394-8	Transcription factor 21	TCF21	O43680	0.006522	-0.11034
seq.24902.84	24902-84	Gephyrin	GPHN	Q9NQX3	0.006522	0.342667
seq.4922.13	4922-13	C-C motif chemokine 19	CCL19	Q99731	0.006522	-0.13575
seq.7092.7	7092-7	Protein disulfide-isomerase-like protein of the testis	PDILT	Q8N807	0.006522	-0.09558
seq.9170.24	9170-24	Interleukin-17A	IL17A	Q16552	0.006522	-0.13947
seq.11911.13	11911-13	Leucine-rich repeat-containing protein 4B:Cytoplasmic domain	LRRC4B	Q9NT99	0.007011	-0.1659
seq.19575.4	19575-4	Erythropoietin receptor	EPOR	P19235	0.007011	-0.1144
seq.17827.53	17827-53	Dynein light chain roadblock-type 2	DYNLRB2	Q8TF09	0.007011	-0.02708
seq.2598.9	2598-9	Tumor necrosis factor receptor superfamily member 9	TNFRSF9	Q07011	0.007011	-0.1441
seq.7196.21	7196-21	Interferon omega-1	IFNW1	P05000	0.007011	-0.10126
seq.13472.35	13472-35	Haloacid dehalogenase-like hydrolase domain-containing protein 2	HDHD2	Q9H0R4	0.007011	0.638544
seq.14755.4	14755-4	Lactotransferrin	LTF	P02788	0.007011	-0.197
seq.16914.104	16914-104	Monocyte differentiation antigen CD14, soluble	CD14	P08571	0.007011	-0.08974
seq.2915.6	2915-6	Proliferating cell nuclear antigen	PCNA	P12004	0.007011	-0.13171
seq.9565.6	9565-6	PRKC apoptosis WT1 regulator protein	PAWR	Q96IZ0	0.007011	0.569485
seq.11186.12	11186-12	Transmembrane protein 52	TMEM52	Q8NDY8	0.007077	-0.13092
seq.11275.94	11275-94	Low-density lipoprotein receptor-related protein 1B	LRP1B	Q9NZR2	0.007077	0.266794
seq.11538.216	11538-216	Malonyl-CoA decarboxylase, mitochondrial	MLYCD	O95822	0.007077	-0.10973
seq.12518.289	12518-289	Protein polybromo-1	PBRM1	Q86U86	0.007077	-0.09854

Disease Models & Mechanisms • Supplementary information

seq.12682.5	12682-5	Kynurenine--oxoglutarate transaminase 3	KYAT3	Q6YP21	0.007077	0.217737
seq.12988.49	12988-49	RNA-binding protein EWS	EWSR1	Q01844	0.007077	0.542375
seq.13475.10	13475-10	Ubiquitin-conjugating enzyme E2 D4	UBE2D4	Q9Y2X8	0.007077	0.652592
seq.13678.169	13678-169	Complement factor D	CFD	P00746	0.007077	-0.14985
seq.13969.24	13969-24	Importin subunit alpha-7	KPNA6	O60684	0.007077	0.855136
seq.14148.2	14148-2	Ubiquitin-like protein ISG15	ISG15	P05161	0.007077	0.674047
seq.14326.4	14326-4	Ubiquitin-conjugating enzyme E2 E1	UBE2E1	P51965	0.007077	0.570267
seq.16055.3	16055-3	Complement factor H-related protein 5	CFHR5	Q9BXR6	0.007077	-0.14446
seq.16621.77	16621-77	NAD(P)H-hydrate epimerase	NAXE	Q8NCW5	0.007077	0.296198
seq.16919.1	16919-1	Acyl-CoA-binding protein	DBI	P07108	0.007077	0.601291
seq.17155.1	17155-1	Vacuolar protein sorting-associated protein 28 homolog	VPS28	Q9UK41	0.007077	0.27242
seq.17744.31	17744-31	Ras-related protein Rab-11A	RAB11A	P62491	0.007077	0.644363
seq.17843.30	17843-30	Phosphopantothenate--cysteine ligase	PPCS	Q9HAB8	0.007077	0.697137
seq.18189.12	18189-12	Vacuolar protein-sorting-associated protein 25	VPS25	Q9BRG1	0.007077	0.772738
seq.18244.1	18244-1	Annexin A7	ANXA7	P20073	0.007077	0.414788
seq.18829.4	18829-4	Eukaryotic initiation factor 4A-I	EIF4A1	P60842	0.007077	0.865967
seq.18909.11	18909-11	Exosome complex component RRP43	EXOSC8	Q96B26	0.007077	0.395304
seq.19290.5	19290-5	Hypoxanthine-guanine phosphoribosyltransferase	HPRT1	P00492	0.007077	0.577991
seq.19329.31	19329-31	Homeodomain-interacting protein kinase 3	HIPK3	Q9H422	0.007077	0.11279
seq.19332.1	19332-1	MOB-like protein phocein	MOB4	Q9Y3A3	0.007077	0.421429
seq.20449.72	20449-72	Josephin-1	JOSD1	Q15040	0.007077	-0.11447
seq.21116.13	21116-13	Polycomb protein EED	EED	O75530	0.007077	0.403994
seq.22393.130	22393-130	Thyroid transcription factor 1-associated protein 26	CCDC59	Q9P031	0.007077	-0.11785
seq.22966.20	22966-20	Gamma-aminobutyric acid receptor-associated protein	GABARAP	O95166	0.007077	0.370533
seq.23351.59	23351-59	Protein arginine N-methyltransferase 1	PRMT1	Q99873	0.007077	0.285448
seq.23410.46	23410-46	Eukaryotic translation initiation factor 3 subunit M	EIF3M	Q7L2H7	0.007077	0.629883
seq.23611.16	23611-16	MARVEL domain-containing protein 2	MARVELD2	Q8N4S9	0.007077	0.26123
seq.23617.15	23617-15	Huntingtin-interacting protein K	HYPK	Q9NX55	0.007077	0.205138
seq.24237.115	24237-115	Voltage-dependent L-type calcium channel subunit beta-3	CACNB3	P54284	0.007077	0.497486
seq.24895.25	24895-25	F-BAR and double SH3 domains protein 1	FCHSD1	Q86WN1	0.007077	0.290963
seq.2632.5	2632-5	Interleukin-12 receptor subunit beta-1	IL12RB1	P42701	0.007077	-0.15613
seq.2728.62	2728-62	Tenascin	TNC	P24821	0.007077	0.466196
seq.5013.2	5013-2	Chloride intracellular channel protein 1	CLIC1	O00299	0.007077	0.240936
seq.5226.36	5226-36	Casein kinase II 2-alpha':2-beta heterotetramer	CSNK2A2|CSNK2B	P19784|P67870	0.007077	0.320637
seq.5384.67	5384-67	Transcription factor IIIB 90 kDa subunit	BRF1	Q92994	0.007077	0.173733
seq.5508.62	5508-62	Cathepsin D	CTSD	P07339	0.007077	0.54994
seq.5858.6	5858-6	14-3-3 protein zeta/delta	YWHAZ	P63104	0.007077	0.408667
seq.5870.23	5870-23	Bcl2-associated agonist of cell death	BAD	Q92934	0.007077	0.29116
seq.6334.9	6334-9	Inactive gamma-glutamyltranspeptidase 2	GGT2	P36268	0.007077	0.184141
seq.8309.12	8309-12	Hyaluronidase-1	HYAL1	Q12794	0.007077	-0.09214
seq.9370.69	9370-69	Gamma-glutamyl hydrolase	GGH	Q92820	0.007077	0.338315
seq.9755.19	9755-19	Drebrin-like protein	DBNL	Q9UJU6	0.007077	0.664704
seq.9859.180	9859-180	Diamine acetyltransferase 1	SAT1	P21673	0.007077	-0.06747
seq.22503.24	22503-24	Homeobox protein Mohawk	MKX	Q8IYA7	0.007208	-0.11941
seq.23152.49	23152-49	BTB/POZ domain-containing adapter for CUL3-mediated RhoA degradation protein 2	TNFAIP1	Q13829	0.007208	-0.179
seq.25280.2	25280-2	LIM domain-containing protein 1	LIMD1	Q9UGP4	0.007208	0.325875
seq.5743.82	5743-82	Cocaine- and amphetamine-regulated transcript protein	CARTPT	Q16568	0.007208	-0.08484
seq.8106.15	8106-15	Translocon-associated protein subunit alpha	SSR1	P43307	0.007208	-0.09738
seq.11827.7	11827-7	Nuclear receptor ROR-gamma	RORC	P51449	0.007208	-0.15465
seq.13741.36	13741-36	Insulin-like growth factor-binding protein 1	IGFBP1	P08833	0.007208	-0.15659
seq.14056.4	14056-4	Glycoprotein hormones alpha chain	CGA	P01215	0.007208	-0.09587
seq.23531.79	23531-79	Hematopoietic SH2 domain-containing protein	HSH2D	Q96JZ2	0.007208	-0.09699
seq.3452.17	3452-17	Tyrosine-protein kinase Lck	LCK	P06239	0.007208	-0.11546
seq.5359.65	5359-65	Serine/threonine-protein kinase pim-1	PIM1	P11309	0.007208	-0.06584
seq.7200.4	7200-4	Leucine-rich repeat and fibronectin type-III domain-containing protein 2	LRFN2	Q9ULH4	0.007208	0.190461
seq.8380.244	8380-244	Protein delta homolog 1:Cytoplasmic domain	DLK1	P80370	0.007208	-0.07291
seq.10618.190	10618-190	Low-density lipoprotein receptor-related protein 2	LRP2	P98164	0.007208	0.171772
seq.11556.19	11556-19	ADP-ribosylation factor GTPase-activating protein 1	ARFGAP1	Q8N6T3	0.007208	0.115932
seq.13993.20	13993-20	Band 4.1-like protein 1	EPB41L1	Q9H4G0	0.007208	0.325676
seq.14049.17	14049-17	Interleukin-7	IL7	P13232	0.007208	-0.09507
seq.15583.18	15583-18	Fc receptor-like B	FCRLB	Q6BAA4	0.007208	-0.14836
seq.16614.27	16614-27	R-spondin-1	RSPO1	Q2MKA7	0.007208	-0.08517
seq.20374.41	20374-41	Ubiquinone biosynthesis protein COQ9, mitochondrial	COQ9	O75208	0.007208	0.48261
seq.2867.52	2867-52	RAC-alpha serine/threonine-protein kinase	AKT1	P31749	0.007208	0.306895
seq.3332.57	3332-57	Hemojuvelin	HJV	Q6ZVN8	0.007208	-0.11404
seq.4990.87	4990-87	Platelet glycoprotein Ib alpha chain	GP1BA	P07359	0.007208	-0.10086
seq.5738.25	5738-25	Tenascin	TNC	P24821	0.007208	0.384113
seq.5939.42	5939-42	Tumor necrosis factor ligand superfamily member 12	TNFSF12	O43508	0.007208	-0.15972
seq.6497.10	6497-10	Retinoschisin	RS1	O15537	0.007208	-0.11245
seq.8606.39	8606-39	Transmembrane glycoprotein NMB:Cytoplasmic domain	GPNMB	Q14956	0.007208	-0.15354
seq.6399.52	6399-52	Beta-defensin 107	DEFB107A	Q8IZN7	0.00775	-0.0934
seq.6530.63	6530-63	Opiorphin prepropeptide	OPRPN	Q99935	0.00775	-0.09493
seq.13076.4	13076-4	Fragile X mental retardation syndrome-related protein 1	FXR1	P51114	0.00775	0.358533
seq.14111.15	14111-15	Thrombospondin-2	THBS2	P35442	0.00775	-0.1003
seq.22496.21	22496-21	LYR motif-containing protein 1	LYRM1	O43325	0.00775	0.240893
seq.3676.15	3676-15	Cation-independent mannose-6-phosphate receptor	IGF2R	P11717	0.00775	0.271917
seq.5742.14	5742-14	Lysophosphatidic acid phosphatase type 6	ACP6	Q9NPH0	0.00775	0.422783
seq.6276.16	6276-16	Furin	FURIN	P09958	0.00775	0.091641
seq.9057.19	9057-19	Proteasomal ubiquitin receptor ADRM1	ADRM1	Q16186	0.00775	-0.07538
seq.2939.10	2939-10	Artemin	ARTN	Q5T4W7	0.008085	-0.14223
seq.11187.11	11187-11	C-type lectin domain family 12 member A	CLEC12A	Q5QGZ9	0.008085	-0.13486
seq.12678.66	12678-66	U1 small nuclear ribonucleoprotein A	SNRPA	P09012	0.008085	0.51014
seq.12790.10	12790-10	Coiled-coil domain-containing protein 51	CCDC51	Q96ER9	0.008085	-0.07456
seq.13126.52	13126-52	Desmocollin-2	DSC2	Q02487	0.008085	0.646876

Disease Models & Mechanisms • Supplementary information

seq.13408.23	13408-23	WAP, Kazal, immunoglobulin, Kunitz and NTR domain-containing protein 2	WFIKKN2	Q8TEU8	0.008085	0.134626
seq.13621.31	13621-31	AP-2 complex subunit alpha-2	AP2A2	O94973	0.008085	0.491998
seq.13983.27	13983-27	Quinone oxidoreductase	CRYZ	Q08257	0.008085	-0.18541
seq.15303.63	15303-63	Protein S100-A5	S100A5	P33763	0.008085	0.381463
seq.16596.25	16596-25	Glutaredoxin-3	GLRX3	O76003	0.008085	1.383842
seq.17514.48	17514-48	Ras-related protein Rab-21	RAB21	Q9UL25	0.008085	1.549318
seq.18280.29	18280-29	Putative D-tyrosyl-tRNA(Tyr) deacylase 2	DTD2	Q96FN9	0.008085	0.566729
seq.18901.26	18901-26	Heat shock 70 kDa protein 1B	HSPA1B	P0DMV9	0.008085	0.595124
seq.19257.11	19257-11	Clathrin light chain A	CLTA	P09496	0.008085	0.745793
seq.19601.15	19601-15	Ankyrin repeat and SOCS box protein 9	ASB9	Q96DX5	0.008085	0.869537
seq.20091.138	20091-138	Ephrin-A1	EFNA1	P20827	0.008085	0.709909
seq.20376.64	20376-64	Putative deoxyribonuclease TATDN1	TATDN1	Q6P1N9	0.008085	0.579834
seq.20439.14	20439-14	Proline synthase co-transcribed bacterial homolog protein	PLPBP	O94903	0.008085	0.750655
seq.21341.19	21341-19	Charged multivesicular body protein 4a	CHMP4A	Q9BY43	0.008085	0.56259
seq.21975.22	21975-22	Islet cell autoantigen 1	ICA1	Q05084	0.008085	0.452972
seq.22777.46	22777-46	S-phase kinase-associated protein 2	SKP2	Q13309	0.008085	0.277908
seq.23242.2	23242-2	Sjoegren syndrome nuclear autoantigen 1	SSNA1	O43805	0.008085	0.294603
seq.23559.10	23559-10	DDRGK domain-containing protein 1	DDRGK1	Q96HY6	0.008085	0.436603
seq.25117.17	25117-17	PGM5	PGM5	Q15124	0.008085	0.586392
seq.2906.55	2906-55	Interleukin-4	IL4	P05112	0.008085	-0.10971
seq.3171.57	3171-57	Amyloid beta A4 protein	APP	P05067	0.008085	0.599324
seq.4414.69	4414-69	Pulmonary surfactant-associated protein D	SFTPD	P35247	0.008085	0.660865
seq.4807.13	4807-13	Collagen alpha-1(VIII) chain	COL8A1	P27658	0.008085	-0.08672
seq.5272.55	5272-55	SHC-transforming protein 1:Src Homology domain	SHC1	P29353	0.008085	0.359284
seq.6259.60	6259-60	Tenascin	TNC	P24821	0.008085	0.56144
seq.6453.70	6453-70	Leukocyte immunoglobulin-like receptor subfamily B member 4	LILRB4	Q8NHJ6	0.008085	0.55592
seq.6645.53	6645-53	Periostin	POSTN	Q15063	0.008085	0.957114
seq.6955.68	6955-68	Sorting nexin-1	SNX1	Q13596	0.008085	0.206042
seq.8273.84	8273-84	Interleukin-31 receptor subunit alpha	IL31RA	Q8NI17	0.008085	-0.06684
seq.8340.9	8340-9	Beta-defensin 110	DEFB110	Q30KQ9	0.008085	-0.07717
seq.8351.17	8351-17	Serine protease 57	PRSS57	Q6UWY2	0.008085	-0.117
seq.8356.88	8356-88	Oxytocin-neurophysin 1	OXT	P01178	0.008085	-0.10266
seq.9199.6	9199-6	Ubiquitin-conjugating enzyme E2 G2	UBE2G2	P60604	0.008085	0.199306
seq.9895.77	9895-77	DNA repair endonuclease XPF	ERCC4	Q92889	0.008085	-0.07595
seq.5031.10	5031-10	Spectrin alpha chain, non-erythrocytic 1	SPTAN1	Q13813	0.008085	-0.09995
seq.12876.39	12876-39	Sperm flagellar protein 1	SPEF1	Q9Y4P9	0.008085	-0.12327
seq.9000.177	9000-177	Neutral and basic amino acid transport protein rBAT	SLC3A1	Q07837	0.008085	-0.08492
seq.9550.153	9550-153	Tetratricopeptide repeat protein 17:Tetratricopeptide repeat 2	TTC17	Q96AE7	0.008085	-0.1221
seq.9933.49	9933-49	Protein ATP1B4	ATP1B4	Q9UN42	0.008085	-0.09253
seq.10043.31	10043-31	Bromodomain-containing protein 4	BRD4	O60885	0.008085	-0.07845
seq.13745.10	13745-10	Polycystin-2:Cytoplasmic domain 4	PKD2	Q13563	0.008085	-0.0997
seq.14624.51	14624-51	Transcriptional repressor CTCF	CTCF	P49711	0.008085	-0.12451
seq.20382.8	20382-8	Motile sperm domain-containing protein 1	MOSPD1	Q9UJG1	0.008085	-0.12485
seq.21495.134	21495-134	Prosaposin receptor GPR37	GPR37	O15354	0.008085	0.230438
seq.23596.17	23596-17	Coiled-coil domain-containing protein 89	CCDC89	Q8N998	0.008085	-0.14726
seq.6228.58	6228-58	Sperm acrosome membrane-associated protein 1	SPACA1	Q9HBV2	0.008085	-0.1008
seq.6575.79	6575-79	ADAMTS-like protein 1	ADAMTSL1	Q8N6G6	0.008085	-0.12089
seq.6599.5	6599-5	Protein APCDD1	APCDD1	Q8J025	0.008085	-0.16454
seq.7551.33	7551-33	Leucine-rich repeat-containing protein 32	LRRC32	Q14392	0.008085	-0.09845
seq.9384.17	9384-17	Cathelicidin antimicrobial peptide	CAMP	P49913	0.008085	-0.10185
seq.5719.66	5719-66	Protein FAM189A2	FAM189A2	Q15884	0.008665	-0.07277
seq.19570.12	19570-12	Fibroblast growth factor 8	FGF8	P55075	0.008665	-0.15375
seq.3401.8	3401-8	Tyrosine-protein phosphatase non-receptor type 2	PTPN2	P17706	0.008665	-0.09816
seq.11219.95	11219-95	Fibroblast growth factor-binding protein 3	FGFBP3	Q8TAT2	0.008665	0.553245
seq.11324.3	11324-3	Peroxidasin-like protein	PXDNL	A1KZ92	0.008665	-0.09862
seq.25081.6	25081-6	Protein FAM160B1	FAM160B1	Q5W0V3	0.008665	0.373793
seq.4304.18	4304-18	Ligand-dependent nuclear receptor corepressor-like protein	LCORL	Q8N3X6	0.008665	-0.13667
seq.4560.34	4560-34	Tyrosine-protein kinase Lck	LCK	P06239	0.008665	-0.13294
seq.6168.11	6168-11	Cellular tumor antigen p53 R175H mutant	TP53	P04637	0.008665	0.119043
seq.7737.76	7737-76	Integrin beta-6	ITGB6	P18564	0.008665	-0.12396
seq.9090.9	9090-9	Synaptotagmin-like protein 4:Ca2+-dependent membrane-targeting module 1	SYTL4	Q96C24	0.008665	0.199805
seq.21750.25	21750-25	Ubiquitin carboxyl-terminal hydrolase BAP1	BAP1	Q92560	0.009254	-0.1289
seq.11351.233	11351-233	Non-homologous end-joining factor 1	NHEJ1	Q9H9Q4	0.009254	-0.10234
seq.13513.174	13513-174	E3 SUMO-protein ligase PIAS3	PIAS3	Q9Y6X2	0.009254	-0.12046
seq.22563.4	22563-4	Kin of IRRE-like protein 1	KIRREL1	Q96J84	0.009254	-0.11643
seq.24445.38	24445-38	Actin filament-associated protein 1-like 2	AFAP1L2	Q8N4X5	0.009254	0.077256
seq.10473.2	10473-2	Trefoil factor 2	TFF2	Q03403	0.009254	-0.15957
seq.11544.39	11544-39	PHD finger protein 3	PHF3	Q92576	0.009254	-0.16058
seq.22512.18	22512-18	Neuroguidin	NGDN	Q8NEJ9	0.009254	-0.12552
seq.24679.35	24679-35	Transcription factor ETV7	ETV7	Q9Y603	0.009254	-0.10697
seq.11617.1	11617-1	Integrin alpha-L	ITGAL	P20701	0.009254	-0.07688
seq.12439.67	12439-67	Interferon regulatory factor 9	IRF9	Q00978	0.009254	-0.10015
seq.17343.6	17343-6	Syntaphilin	SNPH	O15079	0.009254	-0.11389
seq.19225.11	19225-11	B-cell linker protein	BLNK	Q8WV28	0.009254	-0.11734
seq.4874.3	4874-3	Angiogenin	ANG	P03950	0.009254	0.160778
seq.5087.5	5087-5	Interleukin-22 receptor subunit alpha-2	IL22RA2	Q969J5	0.009254	-0.13116
seq.5242.37	5242-37	Dual specificity mitogen-activated protein kinase kinase 4	MAP2K4	P45985	0.009254	0.200087
seq.7780.34	7780-34	Marginal zone B- and B1-cell-specific protein	MZB1	Q8WU39	0.009254	-0.11128
seq.7865.126	7865-126	HRAS-like suppressor 3	PLAAT3	P53816	0.009254	-0.08011
seq.8850.5	8850-5	Putative inactive group IIC secretory phospholipase A2	PLA2G2C	Q5R387	0.009254	-0.11476
seq.11163.7	11163-7	Protein FAM162B	FAM162B	Q5T6X4	0.009289	-0.0732
seq.11248.43	11248-43	Uroporphyrinogen-III synthase	UROS	P10746	0.009289	0.123951
seq.11614.29	11614-29	Enhancer of rudimentary homolog	ERH	P84090	0.009289	0.514532
seq.12517.52	12517-52	Programmed cell death protein 5	PDCD5	O14737	0.009289	0.604694
seq.12560.9	12560-9	5'(3')-deoxyribonucleotidase, cytosolic type	NT5C	Q8TCD5	0.009289	0.531719

Disease Models & Mechanisms • Supplementary information

seq.12760.34	12760-34	Zinc finger protein 774	ZNF774	Q6NX45	0.009289	-0.1388
seq.13640.5	13640-5	B-cell lymphoma 6 protein	BCL6	P41182	0.009289	-0.08603
seq.14048.7	14048-7	Interleukin-1 Receptor accessory protein	IL1RAP	Q9NPH3	0.009289	0.484081
seq.15321.8	15321-8	Complexin-2	CPLX2	Q6PUV4	0.009289	-0.14446
seq.15492.1	15492-1	Cysteine-rich motor neuron 1 protein:Extracellular domain	CRIM1	Q9NZV1	0.009289	-0.10572
seq.16756.30	16756-30	Growth/differentiation factor 7	GDF7	Q7Z4P5	0.009289	0.283579
seq.16875.13	16875-13	Protein mago nashi homolog 2	MAGOHB	Q96A72	0.009289	0.450239
seq.17158.17	17158-17	Ubiquitin-conjugating enzyme E2 G1	UBE2G1	P62253	0.009289	0.269643
seq.19135.5	19135-5	7-methylguanosine phosphate-specific 5'-nucleotidase	NT5C3B	Q969T7	0.009289	0.617634
seq.19558.10	19558-10	Low-density lipoprotein receptor-related protein 4	LRP4	O75096	0.009289	0.204474
seq.20445.29	20445-29	AP-1 complex-associated regulatory protein	AP1AR	Q63HQ0	0.009289	0.22788
seq.20936.8	20936-8	Putative deoxyribonuclease TATDN3	TATDN3	Q17R31	0.009289	0.474344
seq.21203.14	21203-14	MORF4 family-associated protein 1-like 1	MRFAP1L1	Q96HT8	0.009289	-0.0843
seq.21761.213	21761-213	Ubiquitin carboxyl-terminal hydrolase 46	USP46	P62068	0.009289	0.152364
seq.21923.24	21923-24	SAE1/SAE2	SAE1|UBA2	Q9UBE0|Q9 UBT2	0.009289	0.419041
seq.22013.6	22013-6	Ras association domain-containing protein 2	RASSF2	P50749	0.009289	0.411625
seq.22059.56	22059-56	Palmdelphin	PALMD	Q9NP74	0.009289	0.582082
seq.22068.5	22068-5	Serine/threonine-protein phosphatase 2A 56 kDa regulatory subunit delta isoform	PPP2R5D	Q14738	0.009289	0.336206
seq.22404.4	22404-4	Inositol hexakisphosphate kinase 1	IP6K1	Q92551	0.009289	0.247755
seq.23380.16	23380-16	Trans-3-hydroxy-L-proline dehydratase	L3HYPDH	Q96EM0	0.009289	0.475815
seq.23554.2	23554-2	SH2 domain-containing adapter protein D	SHD	Q96IW2	0.009289	0.512317
seq.24669.12	24669-12	DnaJ homolog subfamily C member 5B	DNAJC5B	Q9UF47	0.009289	0.402263
seq.24901.4	24901-4	Villin-1	VIL1	P09327	0.009289	0.3112
seq.24909.40	24909-40	1-phosphatidylinositol-4,5-bisphosphate phosphodiesterase delta-1	PLCD1	P51178	0.009289	0.212969
seq.2837.3	2837-3	Hepatocyte growth factor receptor	MET	P08581	0.009289	0.216363
seq.2877.3	2877-3	SUMO-conjugating enzyme UBC9	UBE2I	P63279	0.009289	0.597525
seq.2925.9	2925-9	Plasminogen activator inhibitor 1	SERPINE1	P05121	0.009289	1.083265
seq.5011.11	5011-11	Nicotinamide phosphoribosyltransferase	NAMPT	P43490	0.009289	0.44148
seq.5496.49	5496-49	Spondin-1	SPON1	Q9HCB6	0.009289	1.513547
seq.6256.9	6256-9	Lymphocyte antigen 6 complex locus protein G6c	LY6G6C	O95867	0.009289	0.921895
seq.6281.51	6281-51	CMP-N-acetylneuraminate-beta-galactosamide-alpha-2,3-sialyltransferase 2	ST3GAL2	Q16842	0.009289	0.578114
seq.6420.4	6420-4	Succinate dehydrogenase assembly factor 2, mitochondrial	SDHAF2	Q9NX18	0.009289	0.288025
seq.6511.17	6511-17	Protein FAM19A4	TAFA4	Q96LR4	0.009289	-0.07209
seq.7732.45	7732-45	Vesicle-associated membrane protein 4	VAMP4	O75379	0.009289	-0.10641
seq.8903.1	8903-1	Cytochrome c oxidase subunit 6C	COX6C	P09669	0.009289	0.230648
seq.9303.9	9303-9	ICOS ligand	ICOSLG	O75144	0.009289	-0.09735
seq.10705.14	10705-14	Alpha-N-acetylgalactosaminide alpha-2,6-sialyltransferase 3	ST6GALNAC3	Q8NDV1	0.009694	-0.20208
seq.10966.1	10966-1	Alpha-2-HS-glycoprotein	AHSG	P02765	0.009694	-0.15355
seq.23658.1	23658-1	Probable asparagine--tRNA ligase, mitochondrial	NARS2	Q96I59	0.009694	-0.12355
seq.3388.58	3388-58	Serine/threonine-protein kinase PAK 5	PAK5	Q9P286	0.009694	-0.07651
seq.12347.29	12347-29	Cerebral cavernous malformations 2 protein	CCM2	Q9BSQ5	0.009694	0.126619
seq.12800.5	12800-5	Tubulin polymerization-promoting protein family member 2	TPPP2	P59282	0.009694	0.26376
seq.14035.13	14035-13	T-lymphoma invasion and metastasis-inducing protein 1	TIAM1	Q13009	0.009694	-0.1416
seq.3344.60	3344-60	Antithrombin-III	SERPINC1	P01008	0.010298	-0.15194
seq.20564.53	20564-53	Thy-1 membrane glycoprotein	THY1	P04216	0.010298	0.664962
seq.8485.7	8485-7	Kelch-like ECH-associated protein 1	KEAP1	Q14145	0.010298	0.286736
seq.10695.12	10695-12	Protein WFDC10B	WFDC10B	Q8IUB3	0.010298	-0.11976
seq.12786.61	12786-61	Glycerophosphocholine phosphodiesterase GPCPD1	GPCPD1	Q9NPB8	0.010298	-0.09027
seq.12880.1	12880-1	Synaptic vesicle glycoprotein 2A	SV2A	Q7L0J3	0.010298	-0.07618
seq.25464.1	25464-1	Cytochrome P450 2C19	CYP2C19	P33261	0.010298	-0.13547
seq.4981.6	4981-6	Desmocollin-3	DSC3	Q14574	0.010298	-0.11301
seq.5752.63	5752-63	Sushi domain-containing protein 3	SUSD3	Q96L08	0.010298	-0.10323
seq.7203.125	7203-125	Beta-1,3-N-acetylglucosaminyltransferase radical fringe	RFNG	Q9Y644	0.010298	-0.05812
seq.7890.68	7890-68	Inactive dipeptidyl peptidase 10	DPP10	Q8N608	0.010298	-0.09854
seq.8222.49	8222-49	Cadherin-related family member 3	CDHR3	Q6ZTQ4	0.010298	-0.14355
seq.9561.21	9561-21	Versican core protein	VCAN	P13611	0.010298	-0.09937
seq.10880.38	10880-38	Protein FAM163B	FAM163B	P0C2L3	0.010298	0.106801
seq.11215.6	11215-6	Cadherin-15:Cytoplasmic domain	CDH15	P55291	0.010298	-0.08822
seq.14129.1	14129-1	Interferon alpha-7	IFNA7	P01567	0.010298	-0.11214
seq.17202.37	17202-37	Programmed cell death protein 4	PDCD4	Q53EL6	0.010298	-0.09548
seq.20519.7	20519-7	CD6	CD6	P30203	0.010298	-0.10346
seq.23339.46	23339-46	Transcription factor 24	TCF24	Q7RTU0	0.010298	-0.10017
seq.24318.13	24318-13	FYVE, RhoGEF and PH domain-containing protein 2	FGD2	Q7Z6J4	0.010298	0.208073
seq.24713.4	24713-4	PDZ domain-containing protein MAGIX	MAGIX	Q9H6Y5	0.010298	-0.07388
seq.2949.6	2949-6	Group 10 secretory phospholipase A2	PLA2G10	O15496	0.010298	-0.09273
seq.5598.3	5598-3	Gremlin-2	GREM2	Q9H772	0.010298	-0.12865
seq.6440.31	6440-31	Microfibrillar-associated protein 5	MFAP5	Q13361	0.010298	0.179247
seq.8428.102	8428-102	Neurotrimin	NTM	Q9P121	0.010298	0.556093
seq.8905.20	8905-20	Calcium-activated potassium channel subunit beta-3	KCNMB3	Q9NPA1	0.010298	0.368566
seq.11449.22	11449-22	F-actin-capping protein subunit alpha-1	CAPZA1	P52907	0.010635	0.547761
seq.11592.1	11592-1	ELAV-like protein 1	ELAVL1	Q15717	0.010635	0.248293
seq.12460.18	12460-18	Proteasome subunit alpha type-7	PSMA7	O14818	0.010635	0.437253
seq.12479.50	12479-50	cAMP-dependent protein kinase type I-beta regulatory subunit	PRKAR1B	P31321	0.010635	0.333372
seq.12635.9	12635-9	tRNA (cytosine(38)-C(5))-methyltransferase	TRDMT1	O14717	0.010635	-0.08002
seq.12690.33	12690-33	Septin-10	SEPTIN10	Q9P0V9	0.010635	0.132379
seq.14324.52	14324-52	Structural maintenance of chromosomes protein 3	SMC3	Q9UQE7	0.010635	0.606409
seq.16760.2	16760-2	Interleukin-26	IL26	Q9NPH9	0.010635	0.197852
seq.16780.6	16780-6	Heat shock 70 kDa protein 1A	HSPA1A	P0DMV8	0.010635	0.415072
seq.16807.35	16807-35	Troponin T, fast skeletal muscle	TNNT3	P45378	0.010635	0.189681
seq.16865.62	16865-62	TATA-binding protein-associated factor 2N	TAF15	Q92804	0.010635	0.378036
seq.17366.6	17366-6	DCN1-like protein 1	DCUN1D1	Q96GG9	0.010635	0.295623
seq.17775.8	17775-8	Transgelin-3	TAGLN3	Q9UI15	0.010635	0.605852
seq.18206.18	18206-18	Alcohol dehydrogenase 6	ADH6	P28332	0.010635	0.596313

Disease Models & Mechanisms • Supplementary information

seq.18261.34	18261-34	Protein NATD1	NATD1	Q8N6N6	0.010635	-0.0879
seq.18295.102	18295-102	Glyoxylate reductase/hydroxypyruvate reductase	GRHPR	Q9UBQ7	0.010635	0.407209
seq.18338.26	18338-26	Isocitrate dehydrogenase [NADP] cytoplasmic	IDH1	O75874	0.010635	0.718969
seq.19293.6	19293-6	Vacuolar protein sorting-associated protein 26A	VPS26A	O75436	0.010635	0.652848
seq.21577.35	21577-35	FK506-binding protein 5	FKBP5	Q13451	0.010635	0.219963
seq.21592.8	21592-8	Cytosolic beta-glucosidase	GBA3	Q9H227	0.010635	-0.11998
seq.21942.14	21942-14	Protein Wnt-16	WNT16	Q9UBV4	0.010635	-0.15175
seq.22779.75	22779-75	Single-pass membrane and coiled-coil domain-containing protein 1	SMCO1	Q147U7	0.010635	-0.10258
seq.22978.13	22978-13	High mobility group protein B2	HMGB2	P26583	0.010635	0.448488
seq.23386.38	23386-38	T-complex protein 10A homolog 2	None	B9ZVM9	0.010635	0.315722
seq.23649.6	23649-6	Kelch-like protein 2	KLHL2	O95198	0.010635	0.448786
seq.24971.2	24971-2	Phosphatidylinositide phosphatase SAC2	INPP5F	Q9Y2H2	0.010635	0.201386
seq.25438.288	25438-288	5'-nucleotidase domain-containing protein 1	NT5DC1	Q5TFE4	0.010635	-0.06043
seq.2849.49	2849-49	Allograft inflammatory factor 1	AIF1	P55008	0.010635	0.250859
seq.3342.76	3342-76	Abelson tyrosine-protein kinase 2	ABL2	P42684	0.010635	0.218414
seq.3822.54	3822-54	MAP kinase-activated protein kinase 3	MAPKAPK3	Q16644	0.010635	0.572025
seq.3897.61	3897-61	Pyridoxal phosphate phosphatase	PDXP	Q96GD0	0.010635	0.237062
seq.4159.130	4159-130	Complement factor H	CFH	P08603	0.010635	0.249089
seq.4249.64	4249-64	Nucleoside diphosphate kinase B	NME2	P22392	0.010635	0.444047
seq.5864.10	5864-10	Fructose-bisphosphate aldolase A	ALDOA	P04075	0.010635	0.46351
seq.6441.62	6441-62	A disintegrin and metalloproteinase with thrombospondin motifs 6	ADAMTS6	Q9UKP5	0.010635	0.398575
seq.8269.327	8269-327	Arylsulfatase K	ARSK	Q6UWY0	0.010635	0.352503
seq.8346.9	8346-9	Dipeptidyl peptidase 2	DPP7	Q9UHL4	0.010635	-0.11676
seq.8445.184	8445-184	Melittin_VESMG	MELT	P68408	0.010635	-0.1265
seq.8696.15	8696-15	Galactosylceramide sulfotransferase	GAL3ST1	Q99999	0.010635	0.498893
seq.9401.57	9401-57	Metalloprotease TIKI1	TRABD2A	Q86V40	0.010635	0.336691
seq.9426.73	9426-73	DNA-binding protein inhibitor ID-2	ID2	Q02363	0.010635	-0.06448
seq.9748.31	9748-31	Glutathione S-transferase Mu 3	GSTM3	P21266	0.010635	0.500409
seq.9864.38	9864-38	Tetratricopeptide repeat protein 1	TTC1	Q99614	0.010635	0.445982
seq.9970.7	9970-7	UBX domain-containing protein 4:Cytoplasmic domain 2	UBXN4	Q92575	0.010635	0.245506
seq.4891.50	4891-50	Glucagon	GCG	P01275	0.010736	-0.13531
seq.8813.160	8813-160	Thioredoxin	TXN	P10599	0.010736	-0.13248
seq.10835.25	10835-25	Alpha-1,4-N-acetylglucosaminyltransferase	A4GNT	Q9UNA3	0.010736	-0.08375
seq.19606.28	19606-28	Indian hedgehog protein	IHH	Q14623	0.010736	-0.06854
seq.20534.6	20534-6	Scavenger receptor class B member 1	SCARB1	Q8WTV0	0.010736	-0.12641
seq.22083.60	22083-60	Protein BEX5	BEX5	Q5H9J7	0.010736	-0.08432
seq.6933.20	6933-20	39S ribosomal protein L34, mitochondrial	MRPL34	Q9BQ48	0.010736	-0.078
seq.9557.5	9557-5	MANSC domain-containing protein 1	MANSC1	Q9H8J5	0.010736	0.311825
seq.11699.16	11699-16	Protein tyrosine phosphatase type IVA 2	PTP4A2	Q12974	0.010736	0.20332
seq.12423.38	12423-38	APOBEC1 complementation factor	A1CF	Q9NQ94	0.010736	-0.07049
seq.19602.36	19602-36	Transcription factor jun-D	JUND	P17535	0.010736	0.152078
seq.21905.10	21905-10	Integrin alpha-2/b1	ITGA2|ITGB1	P17301|P05556	0.010736	-0.12799
seq.7181.17	7181-17	Vesicle-associated membrane protein-associated protein B/C	VAPB	O95292	0.010736	-0.11276
seq.3280.49	3280-49	Aggrecan core protein	ACAN	P16112	0.011377	-0.10834
seq.16558.2	16558-2	Myocilin	MYOC	Q99972	0.011377	-0.23819
seq.6430.36	6430-36	Protein FAM19A2	TAFA2	Q8N3H0	0.011377	-0.133
seq.24304.3	24304-3	EF-hand calcium-binding domain-containing protein 4B	CRACR2A	Q9BSW2	0.011377	-0.08221
seq.11432.11	11432-11	Polyglutamine-binding protein 1	PQBP1	O60828	0.011377	0.20131
seq.13936.24	13936-24	Phosphoglycerate kinase 2	PGK2	P07205	0.011377	-0.09372
seq.15412.40	15412-40	Baculoviral IAP repeat-containing protein 7	BIRC7	Q96CA5	0.011377	-0.0936
seq.22129.55	22129-55	RNA polymerase II elongation factor ELL3	ELL3	Q9HB65	0.011377	-0.10608
seq.24489.34	24489-34	Transcription factor HES-3	HES3	Q5TGS1	0.011377	-0.10492
seq.3400.49	3400-49	Serine/threonine-protein kinase TBK1	TBK1	Q9UHD2	0.011377	-0.10732
seq.5066.134	5066-134	CMRF35-like molecule 6	CD300C	Q08708	0.011377	-0.12164
seq.5355.69	5355-69	Tumor necrosis factor ligand superfamily member 14	TNFSF14	O43557	0.011377	-0.11509
seq.7201.5	7201-5	Iron-sulfur cluster assembly enzyme ISCU, mitochondrial	ISCU	Q9H1K1	0.011377	-0.10652
seq.7224.11	7224-11	T-cell receptor-associated transmembrane adapter 1	TRAT1	Q6PIZ9	0.011377	-0.11731
seq.8794.13	8794-13	Dipeptidase 1	DPEP1	P16444	0.011377	-0.12255
seq.12715.30	12715-30	mRNA-decapping enzyme 1B	DCP1B	Q8IZD4	0.011377	-0.0979
seq.12793.4	12793-4	Piwi-like protein 1	PIWIL1	Q96J94	0.011377	-0.12848
seq.13423.94	13423-94	Redox-regulatory protein FAM213A	PRXL2A	Q9BRX8	0.011377	-0.08073
seq.13561.5	13561-5	5-hydroxytryptamine receptor 6	HTR6	P50406	0.011377	-0.03876
seq.14250.115	14250-115	Bleomycin hydrolase	BLMH	Q13867	0.011377	-0.10467
seq.24456.3	24456-3	Visual system homeobox 1	VSX1	Q9NZR4	0.011377	-0.08983
seq.24704.38	24704-38	COMM domain-containing protein 5	COMMD5	Q9GZQ3	0.011377	-0.10878
seq.4890.10	4890-10	Corticotropin	POMC	P01189	0.011377	-0.10458
seq.5017.19	5017-19	Peroxiredoxin-5, mitochondrial	PRDX5	P30044	0.011377	-0.12252
seq.5897.58	5897-58	Gastrin-releasing peptide	GRP	P07492	0.011377	-0.16686
seq.6248.68	6248-68	Tumor necrosis factor receptor superfamily member 6	FAS	P25445	0.011377	-0.137
seq.6603.18	6603-18	Anosmin-1	ANOS1	P23352	0.011377	-0.11494
seq.7840.64	7840-64	Legumain	LGMN	Q99538	0.011377	0.569205
seq.8833.20	8833-20	Tumor necrosis factor ligand superfamily member 10	TNFSF10	P50591	0.011377	-0.14948
seq.12705.9	12705-9	Probable E3 ubiquitin-protein ligase HERC1	HERC1	Q15751	0.012046	-0.13619
seq.20245.13	20245-13	Retinoic acid receptor RXR-alpha	RXRA	P19793	0.012046	-0.08315
seq.20538.71	20538-71	Sialic acid-binding Ig-like lectin 11:Extracellular domain, Isoform 1	SIGLEC11	Q96RL6	0.012046	-0.14737
seq.2992.59	2992-59	Interleukin-17 receptor A	IL17RA	Q96F46	0.012046	-0.19217
seq.2696.87	2696-87	Persephin	PSPN	O60542	0.012046	-0.11734
seq.3479.71	3479-71	Trypsin-3	PRSS3	P35030	0.012046	-0.13888
seq.6526.77	6526-77	Odorant-binding protein 2a	OBP2A	Q9NY56	0.012046	-0.11209
seq.8462.18	8462-18	Somatotropin	GH1	P01241	0.012046	-0.09768
seq.8756.41	8756-41	Potassium voltage-gated channel subfamily E regulatory beta subunit 5:N-term, opposite side of membrane	KCNE5	Q9UJ90	0.012046	-0.13103
seq.13441.30	13441-30	Dixin	DIXDC1	Q155Q3	0.012046	-0.08126

seq.24693.5	24693-5	Chloride intracellular channel protein 3	CLIC3	O95833	0.012046	0.112973
seq.3728.52	3728-52	Secretin	SCT	P09683	0.012046	-0.10627
seq.7189.55	7189-55	Carbohydrate sulfotransferase 3	CHST3	Q7LGC8	0.012046	-0.1327
seq.8369.102	8369-102	Dystroglycan	DAG1	Q14118	0.012046	-0.1041
seq.8825.4	8825-4	Paired immunoglobulin-like type 2 receptor alpha	PILRA	Q9UKJ1	0.012046	-0.13602
seq.9594.30	9594-30	Zinc transporter 5	SLC30A5	Q8TAD4	0.012046	0.386912
seq.10830.5	10830-5	EF-hand calcium-binding domain-containing protein 14:N-term	EFCAB14	O75071	0.012046	-0.13879
seq.12486.8	12486-8	Glutaredoxin-2, mitochondrial	GLRX2	Q9NS18	0.012046	0.310574
seq.12644.63	12644-63	Adenylosuccinate synthetase isozyme 2	ADSS2	P30520	0.012046	0.203075
seq.12794.6	12794-6	NACHT, LRR and PYD domains-containing protein 4	NLRP4	Q96MN2	0.012046	-0.08943
seq.13083.18	13083-18	Valine--tRNA ligase	VARS1	P26640	0.012046	0.131608
seq.13654.1	13654-1	Rho-associated protein kinase 2	ROCK2	O75116	0.012046	0.170953
seq.14603.51	14603-51	Uncharacterized protein KIAA0040	KIAA0040	Q15053	0.012046	0.244749
seq.15313.28	15313-28	Mitotic spindle assembly checkpoint protein MAD2A	MAD2L1	Q13257	0.012046	0.212835
seq.17326.44	17326-44	Dual specificity protein phosphatase 23	DUSP23	Q9BVJ7	0.012046	-0.1311
seq.17419.17	17419-17	Testin	TES	Q9UGI8	0.012046	0.458999
seq.19193.18	19193-18	Prefoldin subunit 3	VBP1	P61758	0.012046	0.369868
seq.19364.163	19364-163	Proliferating cell nuclear antigen	PCNA	P12004	0.012046	1.038395
seq.19392.6	19392-6	N(G),N(G)-dimethylarginine dimethylaminohydrolase 1	DDAH1	O94760	0.012046	0.209198
seq.20402.11	20402-11	Trafficking protein particle complex subunit 2	TRAPPC2	P0DI81	0.012046	0.490999
seq.20923.10	20923-10	Magnesium-dependent phosphatase 1	MDP1	Q86V88	0.012046	0.625519
seq.21008.113	21008-113	Protein mago nashi homolog	MAGOH	P61326	0.012046	0.595463
seq.21121.31	21121-31	NEDD8-activating enzyme E1 catalytic subunit	UBA3	Q8TBC4	0.012046	0.658963
seq.21569.49	21569-49	Serine/threonine kinase 10	STK10	O94804	0.012046	0.266104
seq.22816.4	22816-4	Tripartite motif-containing protein 54	TRIM54	Q9BYV2	0.012046	0.299823
seq.24898.39	24898-39	Sec1 family domain-containing protein 1	SCFD1	Q8WVM8	0.012046	0.379469
seq.25051.104	25051-104	Fat mass and obesity-associated protein	FTO	Q9C0B1	0.012046	0.714068
seq.25473.62	25473-62	Protein farnesyltransferase/geranylgeranyltransferase type-1 subunit alpha	FNTA	P49354	0.012046	0.414523
seq.25479.8	25479-8	Leucine-rich repeat flightless-interacting protein 2	LRRFIP2	Q9Y608	0.012046	0.280074
seq.2765.4	2765-4	Growth/differentiation factor 11/8	GDF11|MSTN	O95390|O14793	0.012046	0.123823
seq.3284.75	3284-75	Biglycan	BGN	P21810	0.012046	1.101657
seq.3855.56	3855-56	Peroxiredoxin-1	PRDX1	Q06830	0.012046	0.556707
seq.5245.40	5245-40	AMP Kinase (alpha2beta2gamma1)	PRKAA2|PRKAB2|PRKAG1	P54646|O43741|P54619	0.012046	0.478668
seq.5351.52	5351-52	Heterogeneous nuclear ribonucleoproteins A2/B1	HNRNPA2B1	P22626	0.012046	0.512008
seq.6450.8	6450-8	Gastrotropin	FABP6	P51161	0.012046	-0.09572
seq.6605.17	6605-17	Insulin-like growth factor-binding protein complex acid labile subunit	IGFALS	P35858	0.012046	0.191927
seq.8702.42	8702-42	Osteoclast-associated immunoglobulin-like receptor	OSCAR	Q8IYS5	0.012046	-0.08257
seq.8841.65	8841-65	Cartilage intermediate layer protein 2	CILP2	Q8IUL8	0.012046	-0.13707
seq.9758.17	9758-17	40S ribosomal protein S4, X isoform	RPS4X	P62701	0.012046	0.946324
seq.9808.41	9808-41	Trinucleotide repeat-containing gene 6B protein	TNRC6B	Q9UPQ9	0.012046	0.20951
seq.5648.28	5648-28	Chymotrypsinogen B2	CTRB2	Q6GPI1	0.012632	-0.09335
seq.21239.31	21239-31	Cytohesin-1	CYTH1	Q15438	0.012632	0.187047
seq.20544.103	20544-103	Glutamate receptor ionotropic, kainate 2	GRIK2	Q13002	0.012632	-0.17797
seq.5656.53	5656-53	Poly(U)-specific endoribonuclease	ENDOU	P21128	0.012632	-0.10807
seq.6413.79	6413-79	Lipase member K	LIPK	Q5VXJ0	0.012632	-0.09308
seq.8237.56	8237-56	Sclerostin domain-containing protein 1	SOSTDC1	Q6X4U4	0.012632	-0.21638
seq.8800.14	8800-14	Tetratricopeptide repeat protein 17:Tetratricopeptide repeat 3	TTC17	Q96AE7	0.012632	-0.14406
seq.16308.14	16308-14	B- and T-lymphocyte attenuator	BTLA	Q7Z6A9	0.012632	0.115713
seq.22561.3	22561-3	Uncharacterized protein UNQ511/PRO1026	LYPD8	Q6UX82	0.012632	-0.14149
seq.5587.3	5587-3	Coiled-coil domain-containing protein 134	CCDC134	Q9H6E4	0.012632	-0.05985
seq.7813.6	7813-6	Alkaline phosphatase, placental type	ALPP	P05187	0.012632	-0.14929
seq.8093.13	8093-13	Uncharacterized family 31 glucosidase KIAA1161:Cytoplasmic domain	MYORG	Q6NSJ0	0.012632	-0.1175
seq.9049.2	9049-2	Monocarboxylate transporter 4	SLC16A3	O15427	0.012632	-0.10842
seq.11293.14	11293-14	Leucine-rich repeat neuronal protein 1:Cytoplasmic domain	LRRN1	Q6UXK5	0.012632	-0.08789
seq.11716.28	11716-28	Leucine-rich repeat, immunoglobulin-like domain and transmembrane domain-containing protein 2	LRIT2	A6NDA9	0.012632	-0.09008
seq.13504.147	13504-147	Heterogeneous nuclear ribonucleoprotein R	HNRNPR	O43390	0.012632	0.401669
seq.14052.26	14052-26	Protein unc-13 homolog A	UNC13A	Q9UPW8	0.012632	-0.11161
seq.15432.1	15432-1	Otoraplin	OTOR	Q9NRC9	0.012632	-0.07758
seq.16612.28	16612-28	Syndecan-3	SDC3	O75056	0.012632	0.142151
seq.19640.2	19640-2	Parathyroid Hormone1-34	PTH	P01270	0.012632	-0.14551
seq.22511.28	22511-28	Nuclear transcription factor Y subunit alpha	NFYA	P23511	0.012632	-0.11034
seq.22967.15	22967-15	Growth/differentiation factor 7	GDF7	Q7Z4P5	0.012632	0.203262
seq.23006.19	23006-19	Phosphatidylinositol 5-phosphate 4-kinase type-2 beta	PIP4K2B	P78356	0.012632	0.19295
seq.24471.2	24471-2	SET-binding protein	SETBP1	Q9Y6X0	0.012632	-0.11031
seq.24905.22	24905-22	Protein CXorf40A	EOLA1	Q8TE69	0.012632	0.132517
seq.3374.49	3374-49	Tyrosine-protein kinase HCK	HCK	P08631	0.012632	-0.12566
seq.4548.4	4548-4	Galactoside 3(4)-L-fucosyltransferase	FUT3	P21217	0.012632	-0.0983
seq.5807.77	5807-77	CD70 antigen	CD70	P32970	0.012632	-0.1052
seq.9040.144	9040-144	Mammalian ependymin-related protein 1	EPDR1	Q9UM22	0.012632	-0.09783
seq.21491.7	21491-7	Vascular adhesion protein-1	AOC3	Q16853	0.013488	-0.14066
seq.24465.28	24465-28	synovial sarcoma, X breakpoint 4	SSX4	O60224	0.013488	-0.10118
seq.5701.81	5701-81	Tetranectin	CLEC3B	P05452	0.013488	-0.05856
seq.10563.13	10563-13	LysM and putative peptidoglycan-binding domain-containing protein 3	LYSMD3	Q7Z3D4	0.013488	-0.24373
seq.18171.25	18171-25	C-X-C motif chemokine 11	CXCL11	O14625	0.013488	-0.116
seq.4987.17	4987-17	Immunoglobulin alpha Fc receptor	FCAR	P24071	0.013488	-0.09053
seq.9884.8	9884-8	Peptidyl-prolyl cis-trans isomerase-like 1	PPIL1	Q9Y3C6	0.013488	0.392652
seq.18337.4	18337-4	GDP-mannose 4,6 dehydratase	GMDS	O60547	0.013488	0.250764
seq.20990.48	20990-48	Thyrotroph embryonic factor	TEF	Q10587	0.013488	-0.19925
seq.22961.7	22961-7	Ficolin-1	FCN1	O00602	0.013488	-0.06537
seq.6496.60	6496-60	Protein delta homolog 1:Extracellular domain	DLK1	P80370	0.013488	0.286209

seq.6561.77	6561-77	Ig Kappa chain V-I region HK102- like	IGKV1-5	P01602	0.013488	0.256102
seq.8366.19	8366-19	Uncharacterized protein C1orf115	C1orf115	Q9H7X2	0.013488	-0.06974
seq.9319.59	9319-59	Transmembrane emp24 domain-containing protein 4	TMED4	Q7Z7H5	0.013488	-0.11155
seq.11212.7	11212-7	Thioredoxin domain-containing protein 5	TXNDC5	Q8NBS9	0.013767	0.783008
seq.11243.90	11243-90	Adhesion G-protein coupled receptor F1	ADGRF1	Q5T601	0.013767	0.254751
seq.11288.26	11288-26	Cytosolic purine 5'-nucleotidase	NT5C2	P49902	0.013767	0.390497
seq.11352.42	11352-42	Titin	TTN	Q8WZ42	0.013767	-0.08685
seq.12501.10	12501-10	Tubulin-specific chaperone A	TBCA	O75347	0.013767	0.149148
seq.12718.43	12718-43	Peptidyl-prolyl cis-trans isomerase NIMA-interacting 4	PIN4	Q9Y237	0.013767	0.386298
seq.12746.4	12746-4	Cytohesin-4	CYTH4	Q9UIA0	0.013767	0.227279
seq.14082.56	14082-56	Talin-2	TLN2	Q9Y4G6	0.013767	0.229118
seq.14254.27	14254-27	Tyrosine-protein phosphatase non-receptor type 4	PTPN4	P29074	0.013767	0.142228
seq.16754.40	16754-40	Peripheral plasma membrane protein CASK	CASK	O14936	0.013767	0.233507
seq.19207.119	19207-119	Adenosine kinase	ADK	P55263	0.013767	0.29121
seq.20195.13	20195-13	Interferon-induced helicase C domain-containing protein 1	IFIH1	Q9BYX4	0.013767	-0.0686
seq.21945.4	21945-4	Protein Wnt-5b	WNT5B	Q9H1J7	0.013767	-0.45683
seq.2201.17	2201-17	Endostatin	COL18A1	P39060	0.013767	0.284314
seq.22128.8	22128-8	ETS domain-containing protein Elk-4	ELK4	P28324	0.013767	0.191467
seq.22431.164	22431-164	Serum response factor-binding protein 1	SRFBP1	Q8NEF9	0.013767	-0.08731
seq.23543.92	23543-92	Probable tRNA pseudouridine synthase 1	TRUB1	Q8WWH5	0.013767	0.545307
seq.23683.79	23683-79	Tripartite motif-containing protein 55	TRIM55	Q9BYV6	0.013767	0.737031
seq.2634.2	2634-2	Cytokine receptor common subunit gamma	IL2RG	P31785	0.013767	0.145034
seq.3010.53	3010-53	Thymic stromal lymphopoietin	TSLP	Q969D9	0.013767	-0.1354
seq.3457.57	3457-57	Periostin	POSTN	Q15063	0.013767	0.807211
seq.3739.72	3739-72	Integrin alpha-IIb: beta-3 complex	ITGA2B|ITGB3	P08514|P05 106	0.013767	0.165784
seq.4163.5	4163-5	Histone H2A.z	H2AZ1	P0C0S5	0.013767	-0.12334
seq.4297.62	4297-62	Spondin-1	SPON1	Q9HCB6	0.013767	0.492043
seq.7100.31	7100-31	T-cell surface antigen CD2	CD2	P06729	0.013767	-0.11075
seq.8697.38	8697-38	Glypican-1	GPC1	P35052	0.013767	0.334083
seq.8775.61	8775-61	Protein FAM24B	FAM24B	Q8N5W8	0.013767	-0.09661
seq.8953.47	8953-47	Hepatoma-derived growth factor	HDGF	P51858	0.013767	0.496247
seq.8956.96	8956-96	Scavenger receptor class F member 2:Extracellular domain	SCARF2	Q96GP6	0.013767	0.294214
seq.8982.65	8982-65	Thrombospondin-3	THBS3	P49746	0.013767	1.358468
seq.9910.9	9910-9	S-methyl-5'-thioadenosine phosphorylase	MTAP	Q13126	0.013767	0.211835
seq.13431.74	13431-74	Membrane protein FAM159A	SHISAL2A	Q6UWV7	0.014076	-0.10268
seq.8776.10	8776-10	Erlin-1	ERLIN1	O75477	0.014076	-0.07924
seq.22977.18	22977-18	Protein HEXIM1	HEXIM1	O94992	0.014076	-0.07326
seq.11112.18	11112-18	Protein kish-A	TMEM167A	Q8TBQ9	0.014076	-0.12036
seq.12651.21	12651-21	[Pyruvate dehydrogenase (acetyl-transferring)] kinase isozyme 2, mitochondrial	PDK2	Q15119	0.014076	-0.11394
seq.22445.2	22445-2	Calcium-binding protein 5	CABP5	Q9NP86	0.014076	-0.15232
seq.22787.39	22787-39	Sperm protein associated with the nucleus on the X chromosome N3	SPANXN3	Q5MJ09	0.014076	-0.13211
seq.8834.58	8834-58	Calnexin	CANX	P27824	0.014076	-0.10504
seq.11534.6	11534-6	Leucine-rich repeat, immunoglobulin-like domain and transmembrane domain-containing protein 3	LRIT3	Q3SXY7	0.014076	-0.1294
seq.12814.17	12814-17	RNA-binding protein 40	RNPC3	Q96LT9	0.014076	-0.07885
seq.18841.1	18841-1	Serpin B13	SERPINB13	Q9UIV8	0.014076	-0.15535
seq.21361.8	21361-8	Protein TXNRD3NB	TXNRD3NB	Q6F5E7	0.014076	0.309054
seq.5476.66	5476-66	Protein kinase C gamma type	PRKCG	P05129	0.014076	-0.0954
seq.7050.5	7050-5	Neuronal growth regulator 1	NEGR1	Q7Z3B1	0.014076	-0.09981
seq.9518.95	9518-95	Phospholipase A1 member A	PLA1A	Q53H76	0.014076	-0.09251
seq.12811.55	12811-55	Zinc finger protein 415	ZNF415	Q09FC8	0.014076	-0.09966
seq.13411.21	13411-21	E3 ubiquitin-protein ligase NRDP1	RNF41	Q9H4P4	0.014076	0.190192
seq.14012.17	14012-17	Probable ATP-dependent RNA helicase DHX58	DHX58	Q96C10	0.014076	-0.05722
seq.21355.4	21355-4	Upstream stimulatory factor 1	USF1	P22415	0.014076	0.377164
seq.22092.43	22092-43	Caspase-4	CASP4	P49662	0.014076	-0.10777
seq.22103.25	22103-25	Cyclin-dependent kinase 20	CDK20	Q8IZL9	0.014076	-0.07173
seq.25424.234	25424-234	Synaptotagmin-12	SYT12	Q8IV01	0.014076	-0.08115
seq.6264.9	6264-9	Protein CYR61	CCN1	O00622	0.014076	0.522674
seq.6593.5	6593-5	Polypeptide N-acetylgalactosaminyltransferase 3	GALNT3	Q14435	0.014076	-0.09514
seq.7776.20	7776-20	Netrin receptor UNC5B	UNC5B	Q8IZJ1	0.014076	-0.16007
seq.7839.99	7839-99	Protein FAM19A3	TAFA3	Q7Z5A8	0.014076	-0.08891
seq.23202.78	23202-78	Ecto-ADP-ribosyltransferase 5	ART5	Q96L15	0.015036	-0.1766
seq.24931.9	24931-9	Epidermal growth factor receptor kinase substrate 8-like protein 3	EPS8L3	Q8TE67	0.015036	-0.10225
seq.5029.3	5029-3	Prolyl endopeptidase FAP	FAP	Q12884	0.015036	-0.08565
seq.10531.18	10531-18	GTPase NRas	NRAS	P01111	0.015036	0.086824
seq.13651.54	13651-54	E3 ubiquitin-protein ligase ZFP91	ZFP91	Q96JP5	0.015036	-0.1884
seq.21440.9	21440-9	ADAM 8	ADAM8	P78325	0.015036	-0.11087
seq.3187.52	3187-52	Cysteine-rich secretory protein 3	CRISP3	P54108	0.015036	-0.122
seq.7089.42	7089-42	Synaptotagmin-11	SYT11	Q9BT88	0.015036	-0.1041
seq.8236.8	8236-8	UPF0729 protein C18orf32	C18orf32	Q8TCD1	0.015036	-0.13817
seq.17797.1	17797-1	Cullin-1	CUL1	Q13616	0.015036	0.121701
seq.19163.26	19163-26	Leucine-rich repeat-containing protein 59	LRRC59	Q96AG4	0.015036	0.338468
seq.8048.9	8048-9	Ferritin, mitochondrial	FTMT	Q8N4E7	0.015036	0.146141
seq.9290.8	9290-8	Regulator of microtubule dynamics protein 3	RMDN3	Q96TC7	0.015036	-0.08937
seq.10637.50	10637-50	UPF0577 protein KIAA1324:Extracellular domain	ELAPOR1	Q6UXG2	0.015639	-0.13766
seq.10749.18	10749-18	Heat shock 70 kDa protein 1A	HSPA1A	P0DMV8	0.015639	0.578363
seq.11161.5	11161-5	Spartin	SPART	Q8N0X7	0.015639	0.087834
seq.11263.57	11263-57	Calsequestrin-1	CASQ1	P31415	0.015639	0.193926
seq.12399.194	12399-194	Coiled-coil domain-containing protein 50	CCDC50	Q8IVM0	0.015639	0.249087
seq.12427.8	12427-8	M-phase inducer phosphatase 2	CDC25B	P30305	0.015639	0.109117
seq.12647.52	12647-52	Histone-lysine N-methyltransferase SETD2	SETD2	Q9BYW2	0.015639	-0.17211
seq.12788.6	12788-6	SAGA-associated factor 29 homolog	SGF29	Q96ES7	0.015639	-0.10806
seq.13482.14	13482-14	CCR4-NOT transcription complex subunit 1	CNOT1	A5YKK6	0.015639	0.166362

Disease Models & Mechanisms • Supplementary information

seq.13979.3	13979-3	Anion exchange transporter	SLC26A7	Q8TE54	0.015639	0.20723
seq.13998.26	13998-26	Adenylosuccinate synthetase isozyme 1	ADSS1	Q8N142	0.015639	0.784587
seq.14051.54	14051-54	Forkhead box protein C2	FOXC2	Q99958	0.015639	-0.08677
seq.15567.2	15567-2	T-cell surface glycoprotein CD3 zeta chain	CD247	P20963	0.015639	-0.13958
seq.18183.3	18183-3	Low density lipoprotein receptor adapter protein 1	LDLRAP1	Q5SW96	0.015639	0.228048
seq.18286.3	18286-3	ZW10 interactor	ZWINT	O95229	0.015639	0.25995
seq.19504.22	19504-22	Thymidylate kinase	DTYMK	P23919	0.015639	0.516893
seq.21933.7	21933-7	Ubiquitin-like modifier-activating enzyme 7	UBA7	P41226	0.015639	0.26794
seq.22075.16	22075-16	Cyclic AMP-dependent transcription factor ATF-3	ATF3	P18847	0.015639	-0.11874
seq.22583.47	22583-47	NKG2D ligand 2	ULBP2	Q9BZM5	0.015639	-0.11557
seq.22786.57	22786-57	Spermatogenesis-associated protein 46	SPATA46	Q5T0L3	0.015639	0.185396
seq.23267.5	23267-5	BTB/POZ domain-containing protein KCTD6	KCTD6	Q8NC69	0.015639	0.165911
seq.24413.96	24413-96	Peroxisomal coenzyme A diphosphatase NUDT7	NUDT7	P0C024	0.015639	-0.13219
seq.24698.12	24698-12	Pre-mRNA-splicing factor 18	PRPF18	Q99633	0.015639	0.276696
seq.24891.54	24891-54	Epidermal growth factor receptor kinase substrate 8-like protein 2	EPS8L2	Q9H6S3	0.015639	0.193203
seq.25061.8	25061-8	Engulfment and cell motility protein 2	ELMO2	Q96JJ3	0.015639	0.335507
seq.2670.67	2670-67	Creatine kinase M-type	CKM	P06732	0.015639	0.680217
seq.3438.10	3438-10	Follistatin-related protein 3	FSTL3	O95633	0.015639	0.964163
seq.4567.82	4567-82	SH2 domain-containing protein 1A	SH2D1A	O60880	0.015639	-0.1061
seq.4984.83	4984-83	S-formylglutathione hydrolase	ESD	P10768	0.015639	0.667821
seq.5350.14	5350-14	Glypican-6	GPC6	Q9Y625	0.015639	0.263099
seq.5667.3	5667-3	Semaphorin-3B	SEMA3B	Q13214	0.015639	-0.08492
seq.6260.14	6260-14	Tenascin	TNC	P24821	0.015639	1.090477
seq.6382.17	6382-17	Beta-mannosidase	MANBA	O00462	0.015639	0.334022
seq.7267.2	7267-2	Serine palmitoyltransferase 2	SPTLC2	O15270	0.015639	-0.0807
seq.9450.18	9450-18	Cyclin-dependent kinase 2-associated protein 1	CDK2AP1	O14519	0.015639	-0.12628
seq.9878.3	9878-3	Estrogen sulfotransferase	SULT1E1	P49888	0.015639	0.284634
seq.21327.12	21327-12	Modulator of retrovirus infection homolog	CYREN	Q9BWK5	0.015639	0.115641
seq.24970.117	24970-117	Serrate RNA effector molecule homolog	SRRT	Q9BXP5	0.015639	-0.12137
seq.12885.42	12885-42	Nuclear receptor subfamily 1 group D member 2	NR1D2	Q14995	0.015639	-0.0797
seq.13487.24	13487-24	Protein unc-93 homolog B1	UNC93B1	Q9H1C4	0.015639	-0.10074
seq.14079.14	14079-14	Interleukin-18 receptor 1	IL18R1	Q13478	0.015639	-0.08658
seq.15631.18	15631-18	Pregnancy-specific beta-1-glycoprotein 1	PSG1	P11464	0.015639	-0.12184
seq.19142.39	19142-39	DNA (cytosine-5)-methyltransferase 3-like	DNMT3L	Q9UJW3	0.015639	-0.0817
seq.23381.8	23381-8	Septin-1	SEPTIN1	Q8WYJ6	0.015639	0.250091
seq.23650.2	23650-2	Dematin	DMTN	Q08495	0.015639	-0.19383
seq.4261.55	4261-55	Serum paraoxonase/arylesterase 1	PON1	P27169	0.015639	-0.08354
seq.7185.29	7185-29	Platelet glycoprotein V	GP5	P40197	0.015639	-0.09402
seq.9343.16	9343-16	Interleukin-2 receptor subunit beta	IL2RB	P14784	0.015639	-0.07149
seq.10432.3	10432-3	Uncharacterized protein KIAA1644	SHISAL1	Q3SXP7	0.015639	-0.12575
seq.10710.23	10710-23	Zona pellucida-binding protein 2	ZPBP2	Q6X784	0.015639	-0.15253
seq.13747.9	13747-9	Carbonic anhydrase 6	CA6	P23280	0.015639	-0.09304
seq.17449.23	17449-23	CD9 antigen	CD9	P21926	0.015639	-0.07952
seq.22579.93	22579-93	Neurexin-1-beta	NRXN1	P58400	0.015639	-0.10408
seq.23529.11	23529-11	StAR-related lipid transfer protein 7, mitochondrial	STARD7	Q9NQZ5	0.015639	0.200215
seq.24419.3	24419-3	Molybdenum cofactor sulfurase	MOCOS	Q96EN8	0.015639	-0.12644
seq.25306.51	25306-51	DAXX	DAXX	Q9UER7	0.015639	-0.1274
seq.2681.23	2681-23	Hepatocyte growth factor	HGF	P14210	0.015639	0.116638
seq.2762.30	2762-30	Fibroblast growth factor 19	FGF19	O95750	0.015639	-0.21804
seq.3186.2	3186-2	Complement C2	C2	P06681	0.015639	-0.08868
seq.4154.57	4154-57	P-selectin	SELP	P16109	0.015639	-0.13104
seq.8784.7	8784-7	BRCA1-A complex subunit Abraxas	ABRAXAS1	Q6UWZ7	0.015639	-0.08518
seq.8854.59	8854-59	Ribonucleoside-diphosphate reductase subunit M2 B	RRM2B	Q7LG56	0.015639	0.229467
seq.9275.2	9275-2	Sialic acid-binding Ig-like lectin 5	SIGLEC5	O15389	0.015639	-0.11432
seq.9283.8	9283-8	CD44 antigen	CD44	P16070	0.015639	-0.08714
seq.17170.15	17170-15	Calcitonin gene-related peptide 2	CALCB	P10092	0.016637	-0.15278
seq.21742.43	21742-43	OTU domain-containing protein 7B	OTUD7B	Q6GQQ9	0.016637	0.172178
seq.14711.27	14711-27	Cystatin-M	CST6	Q15828	0.016637	-0.07256
seq.7887.57	7887-57	Cytochrome c oxidase subunit 5B, mitochondrial	COX5B	P10606	0.016637	-0.09228
seq.7968.15	7968-15	Cytotoxic and regulatory T-cell molecule	CRTAM	O95727	0.016637	-0.14026
seq.7991.54	7991-54	IQ domain-containing protein F1	IQCF1	Q8N6M8	0.016637	0.173043
seq.8762.38	8762-38	CD70 antigen	CD70	P32970	0.016637	-0.10657
seq.10367.62	10367-62	Interleukin-12	IL12A|IL12B	P29459|P29460	0.016637	-0.12899
seq.13399.33	13399-33	RELT-like protein 1	RELL1	Q8IUW5	0.016637	-0.13668
seq.17346.61	17346-61	Follicular dendritic cell secreted peptide	FDCSP	Q8NFU4	0.016637	-0.14762
seq.23268.15	23268-15	Protein FRG1	FRG1	Q14331	0.016637	0.159146
seq.25279.44	25279-44	Nuclear valosin-containing protein-like	NVL	O15381	0.016637	-0.13321
seq.9568.289	9568-289	High affinity immunoglobulin alpha and immunoglobulin mu Fc receptor	FCAMR	Q8WWV6	0.016637	-0.13105
seq.12529.32	12529-32	Inactive peptidyl-prolyl cis-trans isomerase FKBP6	FKBP6	O75344	0.016637	0.22723
seq.16836.1	16836-1	Complement factor H-related protein 3	CFHR3	Q02985	0.016637	-0.15214
seq.3473.78	3473-78	Thrombopoietin Receptor	MPL	P40238	0.016637	-0.11767
seq.5063.12	5063-12	Natural killer cell receptor 2B4	CD244	Q9BZW8	0.016637	-0.11883
seq.8243.55	8243-55	Serine protease inhibitor Kazal-type 1	SPINK1	P00995	0.016637	-0.07769
seq.8565.160	8565-160	Plasmalemma vesicle-associated protein	PLVAP	Q9BX97	0.016637	-0.08215
seq.9638.2	9638-2	T-cell immunoreceptor with Ig and ITIM domains	TIGIT	Q495A1	0.017515	-0.13098
seq.20126.19	20126-19	Glial fibrillary acidic protein	GFAP	P14136	0.017515	-0.20994
seq.21903.6	21903-6	Integrin alpha L beta 2	ITGAL|ITGB2	P20701|P05107	0.017515	-0.13135
seq.2839.2	2839-2	Tumor necrosis factor ligand superfamily member 4	TNFSF4	P23510	0.017515	-0.08747
seq.18928.10	18928-10	Protein S100-Z	S100Z	Q8WXG8	0.017515	-0.14348
seq.5178.5	5178-5	High affinity cAMP-specific 3',5'-cyclic phosphodiesterase 7A	PDE7A	Q13946	0.017515	-0.04813
seq.5834.18	5834-18	Interleukin-9	IL9	P15248	0.017515	-0.10588
seq.14076.74	14076-74	Cystatin-S	CST4	P01036	0.017515	-0.10793
seq.19145.4	19145-4	Melanoregulin	MREG	Q8N565	0.017515	-0.09176

seq.6357.83	6357-83	Chymotrypsin-like elastase family member 3B	CELA3B	P08861	0.017515	-0.0771
seq.8481.44	8481-44	Malic dehydrogenase_THETH	mdh	P10584	0.017515	-0.07821
seq.8750.46	8750-46	Vinculin	VCL	P18206	0.017515	-0.08698
seq.12879.5	12879-5	Retinoblastoma-like protein 1	RBL1	P28749	0.017515	-0.1025
seq.24953.27	24953-27	Kinesin-like protein KIF3B	KIF3B	O15066	0.017515	-0.10982
seq.3367.8	3367-8	Fetuin-B	FETUB	Q9UGM5	0.017515	-0.1305
seq.6223.5	6223-5	Guanylate cyclase activator 2B	GUCA2B	Q16661	0.017515	-0.19218
seq.16892.23	16892-23	Ectonucleotide pyrophosphatase/phosphodiesterase family member 2	ENPP2	Q13822	0.017515	-0.17271
seq.18876.77	18876-77	Carbohydrate sulfotransferase 4	CHST4	Q8NCG5	0.017515	-0.1277
seq.18881.7	18881-7	CD97 antigen	ADGRE5	P48960	0.017515	-0.0718
seq.21487.20	21487-20	Biogenesis of lysosome-related organelles complex 1 subunit 6	BLOC1S6	Q9UL45	0.017515	-0.06689
seq.21909.10	21909-10	Integrin a5b1	ITGA5|ITGB1	P08648|P05556	0.017515	-0.11826
seq.23622.128	23622-128	SOSS complex subunit C	INIP	Q9NRY2	0.017515	0.21959
seq.25886.11	25886-11	ATP-binding cassette sub-family D member 4	ABCD4	O14678	0.017515	-0.09086
seq.2638.12	2638-12	Macrophage colony-stimulating factor 1 receptor	CSF1R	P07333	0.017515	-0.17363
seq.4394.71	4394-71	Fibroblast growth factor 8 isoform A	FGF8	P55075	0.017515	-0.08268
seq.7240.2	7240-2	Membrane-bound transcription factor site-1 protease	MBTPS1	Q14703	0.017515	-0.10397
seq.9830.109	9830-109	Guanine nucleotide exchange factor VAV3	VAV3	Q9UKW4	0.017515	0.092815
seq.10040.63	10040-63	Tumor protein 63	TP63	Q9H3D4	0.017515	-0.08989
seq.10668.5	10668-5	Syntaxin-4	STX4	Q12846	0.017515	0.669738
seq.10803.22	10803-22	Heat shock 70 kDa protein 1A	HSPA1A	P0DMV8	0.017515	0.322118
seq.11681.8	11681-8	Arf-GAP domain and FG repeat-containing protein 1	AGFG1	P52594	0.017515	0.363376
seq.12400.25	12400-25	Ubiquitin-conjugating enzyme E2 T	UBE2T	Q9NPD8	0.017515	0.357697
seq.13509.5	13509-5	Secretory carrier-associated membrane protein 5	SCAMP5	Q8TAC9	0.017515	-0.12368
seq.13960.15	13960-15	Arf-GAP with GTPase, ANK repeat and PH domain-containing protein 3	AGAP3	Q96P47	0.017515	0.165938
seq.17176.13	17176-13	PC4 and SFRS1-interacting protein	PSIP1	O75475	0.017515	0.471365
seq.18389.11	18389-11	Interferon alpha-1/13	IFNA1	P01562	0.017515	-0.11616
seq.19108.50	19108-50	Methyl-CpG-binding protein 2	MECP2	P51608	0.017515	-0.08941
seq.19194.9	19194-9	D-tyrosyl-tRNA(Tyr) deacylase 1	DTD1	Q8TEA8	0.017515	0.39551
seq.19616.100	19616-100	EF-hand domain-containing protein D1	EFHD1	Q9BUP0	0.017515	0.735834
seq.21107.5	21107-5	Complex I intermediate-associated protein 30, mitochondrial	NDUFAF1	Q9Y375	0.017515	-0.0903
seq.21498.3	21498-3	Alpha-aminoadipic semialdehyde dehydrogenase	ALDH7A1	P49419	0.017515	0.303596
seq.21524.14	21524-14	Ubiquitin carboxyl-terminal hydrolase 5	USP5	P45974	0.017515	0.263368
seq.21780.15	21780-15	Protein ABHD4	ABHD4	Q8TB40	0.017515	-0.10761
seq.21849.2	21849-2	Ubiquilin-3	UBQLN3	Q9H347	0.017515	0.34288
seq.21858.25	21858-25	E3 ubiquitin-protein ligase SIAH1	SIAH1	Q8IUQ4	0.017515	0.236202
seq.21955.36	21955-36	Large proline-rich protein BAT3	BAG6	P46379	0.017515	0.282373
seq.22952.28	22952-28	Cyclin-dependent kinase 2-associated protein 2	CDK2AP2	O75956	0.017515	-0.16388
seq.23008.4	23008-4	Ras-related protein Rab-2A	RAB2A	P61019	0.017515	0.403479
seq.24255.38	24255-38	Epsin-1	EPN1	Q9Y6I3	0.017515	0.402483
seq.25220.8	25220-8	Cysteine-rich tail protein 1	CYSRT1	A8MQ03	0.017515	-0.08039
seq.3470.1	3470-1	E-selectin	SELE	P16581	0.017515	-0.11165
seq.5105.2	5105-2	Reticulon-4 receptor	RTN4R	Q9BZR6	0.017515	0.218286
seq.5754.76	5754-76	Insulin-like peptide INSL6	INSL6	Q9Y581	0.017515	-0.09234
seq.5963.9	5963-9	Dermokine	DMKN	Q6E0U4	0.017515	0.180291
seq.6022.57	6022-57	Protein kinase C-binding protein NELL2	NELL2	Q99435	0.017515	-0.32831
seq.6069.71	6069-71	Sulfatase-modifying factor 2	SUMF2	Q8NBJ7	0.017515	0.174782
seq.6468.37	6468-37	Tachykinin-4	TAC4	Q86UU9	0.017515	-0.08892
seq.6907.17	6907-17	Transmembrane and coiled-coil domain-containing protein 5A	TMCO5A	Q8N6Q1	0.017515	-0.1067
seq.7007.24	7007-24	Trafficking protein particle complex subunit 4	TRAPPC4	Q9Y296	0.017515	0.098849
seq.9083.35	9083-35	Tyrosine-protein phosphatase non-receptor type substrate 1	SIRPA	P78324	0.017515	-0.12134
seq.9278.9	9278-9	Stromal cell-derived factor 1	CXCL12	P48061	0.017515	1.366797
seq.13457.33	13457-33	ETS-related transcription factor Elf-5	ELF5	Q9UKW6	0.018372	-0.13648
seq.2468.62	2468-62	C-C motif chemokine 20	CCL20	P78556	0.018372	-0.11857
seq.11838.130	11838-130	Piezo-type mechanosensitive ion channel component 1	PIEZO1	Q92508	0.018372	-0.09337
seq.13392.13	13392-13	Sodium/potassium-transporting ATPase subunit beta-1	ATP1B1	P05026	0.018372	0.117983
seq.24664.3	24664-3	UPF0510 protein INM02	EMC10	Q5UCC4	0.018372	-0.07477
seq.11573.3	11573-3	Serine/arginine-rich splicing factor 6	SRSF6	Q13247	0.018372	-0.14294
seq.18160.2	18160-2	Protein-L-isoaspartate(D-aspartate) O-methyltransferase	PCMT1	P22061	0.018372	-0.07584
seq.21138.2	21138-2	Nucleoplasmin-2	NPM2	Q86SE8	0.018372	-0.11804
seq.21931.27	21931-27	UB2L3/PolyUbiquitin K48	UBE2L3|UBB	P68036|P0CG47	0.018372	-0.08627
seq.23270.14	23270-14	Spermatogenesis-associated protein 33	SPATA33	Q96N06	0.018372	-0.09291
seq.24649.11	24649-11	Protein N-terminal glutamine amidohydrolase	NTAQ1	Q96HA8	0.018372	0.461983
seq.24912.40	24912-40	WD repeat-containing protein 26	WDR26	Q9H7D7	0.018372	-0.07076
seq.3220.40	3220-40	Proto-oncogene tyrosine-protein kinase receptor Ret	RET	P07949	0.018372	-0.12065
seq.5337.64	5337-64	T-lymphocyte activation antigen CD86	CD86	P42081	0.018372	-0.15163
seq.5734.13	5734-13	Nectin-4	NECTIN4	Q96NY8	0.018372	-0.11493
seq.7881.244	7881-244	Mono [ADP-ribose] polymerase PARP16	PARP16	Q8N5Y8	0.018372	0.092417
seq.11596.47	11596-47	Zinc finger protein 75D	ZNF75D	P51815	0.018372	-0.08672
seq.14103.12	14103-12	Tryptase gamma	TPSG1	Q9NRR2	0.018372	-0.06602
seq.15633.6	15633-6	Retinol-binding protein 4	RBP4	P02753	0.018372	-0.09572
seq.18898.36	18898-36	Hepatoma-derived growth factor-like protein 1	HDGFL1	Q5TGJ6	0.018372	-0.1683
seq.3184.25	3184-25	Coagulation factor VII	F7	P08709	0.018372	-0.08943
seq.7073.69	7073-69	Uncharacterized protein C22orf15	C22orf15	Q8WYQ4	0.018372	-0.147
seq.8932.1	8932-1	Ectonucleoside triphosphate diphosphohydrolase 6	ENTPD6	O75354	0.018372	0.136083
seq.9114.84	9114-84	Uromodulin-like 1	UMODL1	Q5DID0	0.018372	-0.12205
seq.9800.20	9800-20	NADH dehydrogenase [ubiquinone] 1 beta subcomplex subunit 8, mitochondrial	NDUFB8	O95169	0.018372	-0.09099
seq.11242.33	11242-33	Protein-glutamine gamma-glutamyltransferase K	TGM1	P22735	0.019527	-0.06726
seq.10455.196	10455-196	Interleukin-31	IL31	Q6EBC2	0.019527	-0.12559
seq.11144.10	11144-10	Beta-defensin 116	DEFB116	Q30KQ4	0.019527	-0.09579
seq.13984.23	13984-23	ATP-dependent RNA helicase DDX25	DDX25	Q9UHL0	0.019527	-0.08151

Disease Models & Mechanisms • Supplementary information

seq.5749.53	5749-53	Colipase	CLPS	P04118	0.019527	-0.0862
seq.15404.3	15404-3	Interferon alpha-21	IFNA21	P01568	0.019527	-0.1528
seq.25242.12	25242-12	Ciliogenesis-associated TTC17-interacting protein	CATIP	Q7Z7H3	0.019527	0.123768
seq.6557.50	6557-50	Leucine-rich repeat-containing protein 15	LRRC15	Q8TF66	0.019527	0.271492
seq.11223.1	11223-1	Transmembrane protein 154	TMEM154	Q6P9G4	0.019527	-0.09097
seq.12856.14	12856-14	Transmembrane protein 237	TMEM237	Q96Q45	0.019527	-0.05639
seq.17356.34	17356-34	Interleukin-1 family member 10	IL1F10	Q8WWZ1	0.019527	-0.11937
seq.20453.9	20453-9	Non-structural maintenance of chromosomes element 1 homolog	NSMCE1	Q8WV22	0.019527	0.108305
seq.22490.16	22490-16	Lipocalin-like 1 protein	LCNL1	Q6ZST4	0.019527	-0.08718
seq.4245.80	4245-80	E3 ubiquitin-protein ligase Mdm2	MDM2	Q00987	0.019527	-0.10679
seq.4991.12	4991-12	Glypican-5	GPC5	P78333	0.019527	-0.14505
seq.5280.68	5280-68	Mitochondrial glutamate carrier 2	SLC25A18	Q9H1K4	0.019527	-0.15123
seq.15365.41	15365-41	Adhesion G protein-coupled receptor B3	ADGRB3	O60242	0.019527	-0.08546
seq.20411.52	20411-52	Tetratricopeptide repeat protein 33	TTC33	Q6PID6	0.019527	0.196833
seq.7888.58	7888-58	Cytochrome c oxidase assembly factor 3 homolog, mitochondrial	COA3	Q9Y2R0	0.019527	0.17323
seq.11347.9	11347-9	Transaldolase	TALDO1	P37837	0.019838	0.542876
seq.12020.39	12020-39	Bisphosphoglycerate mutase	BPGM	P07738	0.019838	0.149306
seq.12508.9	12508-9	Charged multivesicular body protein 3	CHMP3	Q9Y3E7	0.019838	0.242686
seq.12807.89	12807-89	Rho GTPase-activating protein 30	ARHGAP30	Q7Z6I6	0.019838	0.112931
seq.12831.21	12831-21	Calcineurin B homologous protein 3	TESC	Q96BS2	0.019838	-0.11812
seq.13381.49	13381-49	Beta-1,4-galactosyltransferase 1	B4GALT1	P15291	0.019838	0.372357
seq.13620.10	13620-10	ATPase ASNA1	GET3	O43681	0.019838	-0.09821
seq.16605.2	16605-2	Complement C1q and tumor necrosis factor-related protein 9A	C1QTNF9	P0C862	0.019838	-0.13509
seq.16845.15	16845-15	Myelin protein zero-like protein 1	MPZL1	O95297	0.019838	-0.0796
seq.17734.13	17734-13	Alpha-endosulfine	ENSA	O43768	0.019838	0.136776
seq.17808.37	17808-37	Omega-amidase NIT2	NIT2	Q9NQR4	0.019838	0.379678
seq.18392.19	18392-19	Methionine adenosyltransferase 2 subunit beta	MAT2B	Q9NZL9	0.019838	0.395562
seq.18943.4	18943-4	Prostaglandin reductase 2	PTGR2	Q8N8N7	0.019838	0.233688
seq.19243.2	19243-2	Prefoldin subunit 2	PFDN2	Q9UHV9	0.019838	0.433087
seq.19635.69	19635-69	Fibroleukin	FGL2	Q14314	0.019838	0.18622
seq.21166.1	21166-1	Carboxy-terminal domain RNA polymerase II polypeptide A small phosphatase 1	CTDSP1	Q9GZU7	0.019838	0.427401
seq.21547.6	21547-6	Dentin matrix protein 4	FAM20C	Q8IXL6	0.019838	-0.10285
seq.22001.23	22001-23	Protein-arginine deiminase type-2	PADI2	Q9Y2J8	0.019838	-0.07603
seq.25233.2	25233-2	Mannose-6-phosphate isomerase	MPI	P34949	0.019838	0.420043
seq.2950.57	2950-57	Insulin-like growth factor-binding protein 4	IGFBP4	P22692	0.019838	-0.49723
seq.3427.63	3427-63	Casein kinase II subunit alpha	CSNK2A1	P68400	0.019838	0.791366
seq.3459.49	3459-49	Platelet-derived growth factor receptor beta	PDGFRB	P09619	0.019838	-0.08116
seq.3709.4	3709-4	Alanine aminotransferase 1	GPT	P24298	0.019838	0.270543
seq.3710.49	3710-49	Angiostatin	PLG	P00747	0.019838	-0.09027
seq.4301.58	4301-58	Thymidine kinase, cytosolic	TK1	P04183	0.019838	0.614277
seq.4970.55	4970-55	Carbonic anhydrase 2	CA2	P00918	0.019838	-0.17628
seq.5090.49	5090-49	Leukocyte immunoglobulin-like receptor subfamily B member 1	LILRB1	Q8NHL6	0.019838	-0.09506
seq.5356.2	5356-2	Macrophage migration inhibitory factor	MIF	P14174	0.019838	0.241346
seq.6019.12	6019-12	Complement C1q and tumor necrosis factor-related protein 9A	C1QTNF9	P0C862	0.019838	0.121399
seq.6020.52	6020-52	Urotensin-2	UTS2	O95399	0.019838	0.130854
seq.7096.30	7096-30	Regulator of microtubule dynamics protein 1	RMDN1	Q96DB5	0.019838	-0.55376
seq.7144.234	7144-234	Kazal-type serine protease inhibitor domain-containing protein 1	KAZALD1	Q96I82	0.019838	2.039127
seq.7803.4	7803-4	Carbohydrate sulfotransferase 1	CHST1	O43916	0.019838	-0.06544
seq.8288.27	8288-27	Beta-2-glycoprotein 1	APOH	P02749	0.019838	0.659784
seq.9848.22	9848-22	Cyclin-H	CCNH	P51946	0.019838	0.3301
seq.15602.43	15602-43	Interleukin-6 receptor subunit alpha	IL6R	P08887	0.020387	-0.08772
seq.3067.67	3067-67	Growth/differentiation factor 9	GDF9	O60383	0.020387	-0.16942
seq.11618.83	11618-83	Transcriptional activator Myb	MYB	P10242	0.020387	-0.13602
seq.12558.3	12558-3	Ubiquitin-associated and SH3 domain-containing protein B	UBASH3B	Q8TF42	0.020387	0.232753
seq.22586.24	22586-24	Butyrophilin subfamily 2 member A2	BTN2A2	Q8WVV5	0.020387	-0.16928
seq.4482.66	4482-66	Complement C5b-C6 complex	C5|C6	P01031|P13671	0.020387	-0.14449
seq.8601.167	8601-167	Low-density lipoprotein receptor-related protein 1, soluble:Cytoplasmic domain	LRP1	Q07954	0.020387	-0.17527
seq.11590.5	11590-5	Probable RNA-binding protein 23	RBM23	Q86U06	0.020387	-0.07697
seq.19200.16	19200-16	NADH dehydrogenase [ubiquinone] 1 alpha subcomplex subunit 5	NDUFA5	Q16718	0.020387	-0.13585
seq.21255.2	21255-2	Regulator of G-protein signaling 16	RGS16	O15492	0.020387	-0.05745
seq.5764.4	5764-4	Polypeptide N-acetylgalactosaminyltransferase 2	GALNT2	Q10471	0.020387	-0.12492
seq.7742.11	7742-11	RING finger protein 148:region 1	RNF148	Q8N7C7	0.020387	-0.08587
seq.7918.114	7918-114	Alpha-amylase 1	AMY1A	P04745	0.020387	-0.07796
seq.17736.105	17736-105	Aprataxin	APTX	Q7Z2E3	0.020387	-0.10026
seq.22831.11	22831-11	Z-DNA-binding protein 1	ZBP1	Q9H171	0.020387	-0.1669
seq.3807.1	3807-1	Fibroblast growth factor 23	FGF23	Q9GZV9	0.020387	-0.09761
seq.4801.13	4801-13	Lactoperoxidase	LPO	P22079	0.020387	-0.10105
seq.5301.7	5301-7	Eotaxin	CCL11	P51671	0.020387	-0.13178
seq.5666.64	5666-64	Interleukin-1 receptor type 2	IL1R2	P27930	0.020387	-0.11163
seq.8754.5	8754-5	Neuromedin-U	NMU	P48645	0.020387	-0.09282
seq.8803.61	8803-61	Nectin-1, isoform gamma:Cytoplasmic domain	NECTIN1	Q15223	0.020387	-0.11378
seq.12895.28	12895-28	Diacylglycerol kinase beta	DGKB	Q9Y6T7	0.021597	-0.06005
seq.22115.2	22115-2	cAMP-responsive element-binding protein-like 2	CREBL2	O60519	0.021597	-0.11273
seq.7153.66	7153-66	Neugrin	NGRN	Q9NPE2	0.021597	-0.17357
seq.7983.1	7983-1	Protocadherin gamma-C5	PCDHGC5	Q9Y5F6	0.021597	-0.09818
seq.9248.36	9248-36	Myeloid-derived growth factor	MYDGF	Q969H8	0.021597	-0.09275
seq.17727.1	17727-1	Securin	PTTG1	O95997	0.021597	-0.14489
seq.24702.31	24702-31	Coiled-coil domain-containing protein 149	CCDC149	Q6ZUS6	0.021597	-0.0936
seq.7994.41	7994-41	ERO1-like protein beta	ERO1B	Q86YB8	0.021597	-0.10444
seq.21981.2	21981-2	Integrin alpha-M	ITGAM	P11215	0.021597	-0.08643

Disease Models & Mechanisms • Supplementary information

seq.6356.3	6356-3	Carboxypeptidase B	CPB1	P15086	0.021597	-0.13839
seq.11952.1	11952-1	Immunoglobulin superfamily DCC subclass member 3:Cytoplasmic domain	IGDCC3	Q8IVU1	0.021597	-0.09368
seq.12462.20	12462-20	Histone-lysine N-methyltransferase SETMAR	SETMAR	Q53H47	0.021597	-0.05552
seq.17805.35	17805-35	Survival of motor neuron-related-splicing factor 30	SMNDC1	O75940	0.021597	0.152325
seq.19286.30	19286-30	Myosin light chain 5	MYL5	Q02045	0.021597	0.236124
seq.22383.21	22383-21	MITF	MITF	O75030	0.021597	0.221195
seq.23274.27	23274-27	EF-hand calcium-binding domain-containing protein 1	EFCAB1	Q9HAE3	0.021597	0.235295
seq.24286.48	24286-48	Meiosis expressed gene 1 protein homolog	MEIG1	Q5JSS6	0.021597	-0.0929
seq.2751.16	2751-16	Azurocidin	AZU1	P20160	0.021597	-0.07626
seq.3798.71	3798-71	Carbonic anhydrase 9	CA9	Q16790	0.021597	-0.09138
seq.4140.3	4140-3	Interleukin-7	IL7	P13232	0.021597	-0.1561
seq.4144.13	4144-13	C-C motif chemokine 13	CCL13	Q99616	0.021597	-0.1301
seq.7121.2	7121-2	Synaptotagmin-7	SYT7	O43581	0.021597	-0.13193
seq.8906.60	8906-60	Leucine-rich repeat and transmembrane domain-containing protein 2	LRTM2	Q8N967	0.021597	0.102733
seq.8974.172	8974-172	Collagen alpha-1(XV) chain	COL15A1	P39059	0.021597	0.186255
seq.9725.46	9725-46	RING finger protein 219_MOUSE	Obi1	Q8K2Y0	0.021597	-0.09228
seq.9787.23	9787-23	Collectin-12	COLEC12	Q5KU26	0.021597	-0.09488
seq.10049.112	10049-112	Telomeric repeat-binding factor 1	TERF1	P54274	0.022455	-0.06941
seq.10945.11	10945-11	Syntaxin-6	STX6	O43752	0.022455	0.135846
seq.11211.7	11211-7	Tubulin-specific chaperone E	TBCE	Q15813	0.022455	0.835956
seq.11851.21	11851-21	Triggering receptor expressed on myeloid cells 2	TREM2	Q9NZC2	0.022455	0.114553
seq.12846.3	12846-3	F-box/LRR-repeat protein 5	FBXL5	Q9UKA1	0.022455	-0.12879
seq.14268.4	14268-4	Sulfiredoxin-1	SRXN1	Q9BYN0	0.022455	0.484673
seq.18173.11	18173-11	Aflatoxin B1 aldehyde reductase member 3	AKR7A3	O95154	0.022455	-0.1004
seq.18930.28	18930-28	Slit homolog 2 protein	SLIT2	O94813	0.022455	0.675828
seq.19276.124	19276-124	Small nuclear ribonucleoprotein G	SNRPG	P62308	0.022455	0.375599
seq.19742.3	19742-3	Septin-5	SEPTIN5	Q99719	0.022455	0.244592
seq.19823.75	19823-75	NAD-dependent protein deacetylase sirtuin-1	SIRT1	Q96EB6	0.022455	0.191053
seq.20517.1	20517-1	Desmoglein-2	DSG2	Q14126	0.022455	0.766629
seq.20531.5	20531-5	SLAM family member 9	SLAMF9	Q96A28	0.022455	-0.09477
seq.21692.12	21692-12	CD8A/CD8B Complex	CD8A|CD8B	P01732|P10966	0.022455	-0.08738
seq.22799.17	22799-17	TBC1 domain family member 28	TBC1D28	Q2M2D7	0.022455	-0.0732
seq.23243.120	23243-120	40S ribosomal protein S25	RPS25	P62851	0.022455	0.230056
seq.23330.12	23330-12	Uncharacterized protein C22orf13	GUCD1	Q96NT3	0.022455	-0.12052
seq.25265.8	25265-8	Formin-binding protein 1	FNBP1	Q96RU3	0.022455	0.144223
seq.3616.3	3616-3	N-acetylglucosamine-6-sulfatase	GNS	P15586	0.022455	0.201428
seq.3805.16	3805-16	Endothelial cell-specific molecule 1	ESM1	Q9NQ30	0.022455	-0.10544
seq.5982.50	5982-50	Complement factor H-related protein 1	CFHR1	Q03591	0.022455	-0.0838
seq.6284.7	6284-7	ALK and LTK ligand 2	ALKAL2	Q6UX46	0.022455	0.559308
seq.6379.62	6379-62	ADAMTS-like protein 2	ADAMTSL2	Q86TH1	0.022455	0.30461
seq.7016.12	7016-12	Beta-1,3-galactosyl-O-glycosyl-glycoprotein beta-1,6-N-acetylglucosaminyltransferase	GCNT1	Q02742	0.022455	-0.1428
seq.7756.37	7756-37	Killer cell lectin-like receptor subfamily F member 1	KLRF1	Q9NZS2	0.022455	-0.16415
seq.8528.74	8528-74	Immunoglobulin superfamily containing leucine-rich repeat protein 2	ISLR2	Q6UXK2	0.022455	-0.0805
seq.9002.36	9002-36	Serpin A11	SERPINA11	Q86U17	0.022455	-0.19965
seq.9169.14	9169-14	Small ubiquitin-related modifier 3	SUMO3	P55854	0.022455	0.561773
seq.9876.20	9876-20	Fructose-bisphosphate aldolase C	ALDOC	P09972	0.022455	0.484952
seq.24701.21	24701-21	Dystrobrevin alpha	DTNA	Q9Y4J8	0.022619	-0.18414
seq.10087.10	10087-10	Alpha-crystallin A chain	CRYAA	P02489	0.022619	-0.10079
seq.11137.43	11137-43	Cytokine receptor common subunit beta:Cytoplasmic domain	CSF2RB	P32927	0.022619	-0.09641
seq.13547.5	13547-5	5-hydroxytryptamine receptor 7	HTR7	P34969	0.022619	-0.12763
seq.2381.52	2381-52	Complement C5	C5	P01031	0.022619	-0.14072
seq.25038.5	25038-5	Wee1-like protein kinase 2	WEE2	P0C1S8	0.022619	-0.18374
seq.2616.23	2616-23	Receptor tyrosine-protein kinase erbB-2	ERBB2	P04626	0.022619	-0.08281
seq.3321.2	3321-2	Interleukin-24	IL24	Q13007	0.022619	-0.10441
seq.8897.3	8897-3	Leucine-rich repeat-containing protein 37A2	LRRC37A2	A6NM11	0.022619	-0.0584
seq.9028.5	9028-5	Poly(A) RNA polymerase, mitochondrial	MTPAP	Q9NVV4	0.022619	-0.07379
seq.10063.10	10063-10	E3 ubiquitin-protein ligase FANCL	FANCL	Q9NW38	0.022619	-0.12554
seq.12872.35	12872-35	Cyclic nucleotide-gated olfactory channel	CNGA2	Q16280	0.022619	-0.07488
seq.12940.35	12940-35	Aldehyde dehydrogenase family 3 member B1	ALDH3B1	P43353	0.022619	-0.08499
seq.24907.3	24907-3	Mitochondrial-processing peptidase subunit alpha	PMPCA	Q10713	0.022619	0.153787
seq.24959.20	24959-20	Cytochrome b5 reductase 4	CYB5R4	Q7L1T6	0.022619	0.139885
seq.4126.22	4126-22	Bactericidal permeability-increasing protein	BPI	P17213	0.022619	0.149543
seq.4775.34	4775-34	Gelsolin	GSN	P06396	0.022619	0.692261
seq.5663.18	5663-18	Platelet factor 4 variant	PF4V1	P10720	0.022619	-0.0549
seq.6434.18	6434-18	Retina-specific copper amine oxidase	AOC2	O75106	0.022619	-0.10086
seq.6531.29	6531-29	Protein FAM162A	FAM162A	Q96A26	0.022619	-0.17215
seq.7143.9	7143-9	N-acetyllactosaminide beta-1,6-N-acetylglucosaminyl-transferase, isoform C	None	Q8NFS9	0.022619	-0.11439
seq.9772.153	9772-153	Neuroligin-2:Extracellular domain	NLGN2	Q8NFZ4	0.022619	-0.15005
seq.14178.18	14178-18	Cyclin-dependent kinase inhibitor 3	CDKN3	Q16667	0.023917	-0.06656
seq.14260.112	14260-112	Neuroepithelial cell-transforming gene 1 protein	NET1	Q7Z628	0.023917	-0.06799
seq.19132.1	19132-1	39S ribosomal protein L2, mitochondrial	MRPL2	Q5T653	0.023917	-0.10019
seq.5457.5	5457-5	Collectin-12	COLEC12	Q5KU26	0.023917	-0.08966
seq.13944.3	13944-3	Sulfotransferase 1A3	SULT1A3	P0DMM9	0.023917	-0.11021
seq.8690.25	8690-25	Caveolin-3	CAV3	P56539	0.023917	-0.08883
seq.21164.83	21164-83	Homeobox protein TGIF2LX	TGIF2LX	Q8IUE1	0.023917	-0.20821
seq.5606.24	5606-24	Microfibrillar-associated protein 1	MFAP1	P55081	0.023917	-0.21456
seq.7993.23	7993-23	Astacin-like metalloendopeptidase	ASTL	Q6HA08	0.023917	-0.0549
seq.11692.21	11692-21	SHC-transforming protein 4	SHC4	Q6S5L8	0.023917	-0.10898
seq.13599.15	13599-15	Rab9 effector protein with kelch motifs	RABEPK	Q7Z6M1	0.023917	-0.05343
seq.21342.25	21342-25	Regulator of G-protein signaling 14	RGS14	O43566	0.023917	-0.0706
seq.6388.21	6388-21	Coiled-coil domain-containing protein 126	CCDC126	Q96EE4	0.023917	-0.09359

Disease Models & Mechanisms • Supplementary information

seq.7995.16	7995-16	von Willebrand factor C domain-containing protein 2-like	None	B2RUY7	0.023917	-0.11665
seq.11116.16	11116-16	Uncharacterized protein C11orf87	C11orf87	Q6NUJ2	0.023917	0.174247
seq.12771.19	12771-19	Zinc finger protein 180	ZNF180	Q9UJW8	0.023917	-0.09973
seq.14101.2	14101-2	Ciliary neurotrophic factor receptor subunit alpha	CNTFR	P26992	0.023917	-0.06904
seq.15460.9	15460-9	Dipeptidyl peptidase 4	DPP4	P27487	0.023917	0.122623
seq.17766.5	17766-5	Neutrophil cytosol factor 1	NCF1	P14598	0.023917	-0.13516
seq.21636.63	21636-63	Nuclear cap-binding protein subunit 2	NCBP2	P52298	0.023917	0.111194
seq.21707.15	21707-15	Complement C1q-like protein 3	C1QL3	Q5VWW1	0.023917	-0.1141
seq.2421.7	2421-7	Brain-derived neurotrophic factor	BDNF	P23560	0.023917	-0.16633
seq.24261.202	24261-202	Death-associated protein kinase 3	DAPK3	O43293	0.023917	0.164312
seq.2580.83	2580-83	Myeloperoxidase	MPO	P05164	0.023917	-0.1046
seq.5730.60	5730-60	C-X-C motif chemokine 14	CXCL14	O95715	0.023917	0.264219
seq.6240.70	6240-70	R-spondin-1	RSPO1	Q2MKA7	0.023917	-0.08471
seq.7049.2	7049-2	Disintegrin and metalloproteinase domain-containing protein 23	ADAM23	O75077	0.023917	0.168288
seq.4908.6	4908-6	Endoglin	ENG	P17813	0.024935	-0.13457
seq.18925.24	18925-24	Proteasome subunit alpha type-5	PSMA5	P28066	0.024935	0.202071
seq.20969.114	20969-114	RING1 and YY1-binding protein	RYBP	Q8N488	0.024935	-0.09907
seq.7084.1	7084-1	Draxin	DRAXIN	Q8NBI3	0.024935	-0.12565
seq.12772.8	12772-8	Nuclear pore complex protein Nup98-Nup96	NUP98	P52948	0.024935	-0.09482
seq.13609.11	13609-11	General transcription factor II-I	GTF2I	P78347	0.024935	-0.08172
seq.13668.44	13668-44	Tyrosine-protein kinase receptor TYRO3	TYRO3	Q06418	0.024935	-0.09778
seq.15584.9	15584-9	Complement factor H-related protein 2	CFHR2	P36980	0.024935	-0.15582
seq.18314.88	18314-88	Paired box protein Pax-8	PAX8	Q06710	0.024935	-0.1134
seq.22953.85	22953-85	CCAAT/enhancer-binding protein beta	CEBPB	P17676	0.024935	-0.0789
seq.24256.7	24256-7	Kelch-like protein 40	KLHL40	Q2TBA0	0.024935	-0.11193
seq.6715.63	6715-63	Alkaline phosphatase, placental-like	ALPG	P10696	0.024935	-0.08752
seq.10014.31	10014-31	Zinc finger protein SNAI2	SNAI2	O43623	0.024935	-0.05391
seq.10584.7	10584-7	NADH dehydrogenase [ubiquinone] iron-sulfur protein 4, mitochondrial	NDUFS4	O43181	0.024935	-0.06913
seq.10974.20	10974-20	Serine protease inhibitor Kazal-type 7	SPINK7	P58062	0.024935	-0.12261
seq.14237.1	14237-1	Heat shock 70 kDa protein 1A	HSPA1A	P0DMV8	0.024935	0.05688
seq.15300.66	15300-66	Coiled-coil domain-containing protein 134	CCDC134	Q9H6E4	0.024935	0.244676
seq.15634.139	15634-139	Slit homolog 1 protein	SLIT1	O75093	0.024935	-0.08901
seq.21544.4	21544-4	Atlastin-3	ATL3	Q6DD88	0.024935	-0.09839
seq.22963.3	22963-3	Vesicle-associated membrane protein 2	VAMP2	P63027	0.024935	-0.1604
seq.4931.59	4931-59	Tissue Factor	F3	P13726	0.024935	-0.0754
seq.8935.22	8935-22	Multidrug resistance-associated protein 6	ABCC6	O95255	0.024935	-0.08112
seq.10464.6	10464-6	Anthrax toxin receptor 1	ANTXR1	Q9H6X2	0.024935	-0.08373
seq.11150.3	11150-3	Collagen alpha-1(VI) chain	COL6A1	P12109	0.024935	0.312766
seq.12743.18	12743-18	BAG family molecular chaperone regulator 5	BAG5	Q9UL15	0.024935	-0.07404
seq.13041.47	13041-47	TNF receptor-associated factor 4	TRAF4	Q9BUZ4	0.024935	-0.07269
seq.13459.30	13459-30	Torsin-4A	TOR4A	Q9NXH8	0.024935	-0.09599
seq.13526.5	13526-5	Lupus La protein:RNA recognition motif	SSB	P05455	0.024935	0.193452
seq.13735.1	13735-1	Rap1 GTPase-activating protein 1	RAP1GAP	P47736	0.024935	-0.05531
seq.13738.8	13738-8	Inhibin beta A chain	INHBA	P08476	0.024935	0.971013
seq.13943.38	13943-38	Protein dpy-30 homolog	DPY30	Q9C005	0.024935	0.493906
seq.17760.128	17760-128	WD repeat-containing protein 5	WDR5	P61964	0.024935	0.321184
seq.17799.9	17799-9	6-phosphogluconolactonase	PGLS	O95336	0.024935	0.514518
seq.19127.1	19127-1	Heat shock protein beta-6	HSPB6	O14558	0.024935	0.865387
seq.19808.26	19808-26	Ras-related protein Rab-3D	RAB3D	O95716	0.024935	0.331622
seq.20937.43	20937-43	Ribosomal RNA small subunit methyltransferase NEP1	EMG1	Q92979	0.024935	0.530334
seq.20956.13	20956-13	Prefoldin subunit 4	PFDN4	Q9NQP4	0.024935	0.292322
seq.20984.142	20984-142	Dual specificity protein phosphatase 19	DUSP19	Q8WTR2	0.024935	-0.0551
seq.21260.74	21260-74	Small nuclear ribonucleoprotein E	SNRPE	P62304	0.024935	0.224149
seq.21438.45	21438-45	Tripartite motif-containing protein 5	TRIM5	Q9C035	0.024935	0.194952
seq.21501.30	21501-30	Farnesyl pyrophosphate synthetase	FDPS	P14324	0.024935	0.21847
seq.21581.87	21581-87	Cyclin-dependent kinase 15; EC=2.7.11.22	CDK15	Q96Q40	0.024935	0.170293
seq.21693.14	21693-14	Cadherin-13	CDH13	P55290	0.024935	0.287188
seq.21958.4	21958-4	Active breakpoint cluster region-related protein	ABR	Q12979	0.024935	0.622626
seq.22529.31	22529-31	PHD finger protein 6	PHF6	Q8IWS0	0.024935	-0.07528
seq.25039.10	25039-10	Kelch-like protein 3	KLHL3	Q9UH77	0.024935	-0.07188
seq.25123.198	25123-198	DNA-(apurinic or apyrimidinic site) lyase 2	APEX2	Q9UBZ4	0.024935	-0.0907
seq.25227.20	25227-20	Transcription elongation factor A protein-like 2	TCEAL2	Q9H3H9	0.024935	0.242892
seq.2748.3	2748-3	Activin A	INHBA	P08476	0.024935	1.254326
seq.3214.3	3214-3	Neuropilin-1	NRP1	O14786	0.024935	0.261566
seq.3516.60	3516-60	Stromal cell-derived factor 1	CXCL12	P48061	0.024935	0.370724
seq.3847.56	3847-56	Persulfide dioxygenase ETHE1, mitochondrial	ETHE1	O95571	0.024935	-0.08053
seq.5100.53	5100-53	Lysosome membrane protein 2	SCARB2	Q14108	0.024935	-0.07735
seq.5230.99	5230-99	3-hydroxy-3-methylglutaryl-coenzyme A reductase	HMGCR	P04035	0.024935	-0.08073
seq.6491.59	6491-59	Kallikrein-15	KLK15	Q9H2R5	0.024935	-0.07062
seq.7152.5	7152-5	Hepatitis A virus cellular receptor 2	HAVCR2	Q8TDQ0	0.024935	0.253727
seq.7928.183	7928-183	Protein-tyrosine sulfotransferase 1	TPST1	O60507	0.024935	0.287445
seq.8255.34	8255-34	Protein MRVI1	IRAG1	Q9Y6F6	0.024935	0.214844
seq.8869.5	8869-5	Butyrophilin subfamily 2 member A1	BTN2A1	Q7KYR7	0.024935	-0.09542
seq.8955.60	8955-60	Glycosyltransferase 8 domain-containing protein 1	GLT8D1	Q68CQ7	0.024935	-0.05557
seq.9233.71	9233-71	Tissue factor pathway inhibitor 2	TFPI2	P48307	0.024935	0.776738
seq.9394.19	9394-19	Carboxypeptidase Q	CPQ	Q9Y646	0.024935	0.184287
seq.9478.69	9478-69	Phosphoribosyl pyrophosphate synthase-associated protein 1	PRPSAP1	Q14558	0.024935	0.080179
seq.9997.12	9997-12	UBX domain-containing protein 4:Cytoplasmic domain 1	UBXN4	Q92575	0.024935	0.506549
seq.9470.15	9470-15	Methyltransferase-like protein 24	METTL24	Q5JXM2	0.026337	-0.1082
seq.23929.159	23929-159	Proline-rich transmembrane protein 2	PRRT2	Q7Z6L0	0.026337	-0.0684
seq.12825.18	12825-18	DCC-interacting protein 13-alpha	APPL1	Q9UKG1	0.026337	-0.09268
seq.24658.98	24658-98	Coiled-coil domain-containing protein 115	CCDC115	Q96NT0	0.026337	-0.0742
seq.25941.4	25941-4	2'-5'-oligoadenylate synthetase like protein	OASL	Q15646	0.026337	0.319338
seq.9185.15	9185-15	Trefoil factor 1	TFF1	P04155	0.026337	-0.13325
seq.9932.49	9932-49	Semaphorin-4F	SEMA4F	O95754	0.026337	-0.08809

Disease Models & Mechanisms • Supplementary information

seq.10955.4	10955-4	C-type lectin domain family 10 member A	CLEC10A	Q8IUN9	0.026337	-0.0992
seq.3069.52	3069-52	Immunoglobulin M	IGHM	P01871	0.026337	-0.07238
seq.11715.1	11715-1	POU domain, class 2, transcription factor 1	POU2F1	P14859	0.026337	-0.14626
seq.12785.49	12785-49	Transcriptional regulator Kaiso	ZBTB33	Q86T24	0.026337	-0.09359
seq.13056.18	13056-18	Orphan sodium- and chloride-dependent neurotransmitter transporter NTT5	SLC6A16	Q9GZN6	0.026337	-0.17217
seq.14149.9	14149-9	Interleukin-36 beta	IL36B	Q9NZH7	0.026337	-0.13762
seq.15666.21	15666-21	Bone morphogenetic protein 2	BMP2	P12643	0.026337	-0.09498
seq.21715.40	21715-40	Sentrin-specific protease 1	SENP1	Q9P0U3	0.026337	-0.11689
seq.9828.86	9828-86	L-lactate dehydrogenase C chain	LDHC	P07864	0.026337	-0.08355
seq.10616.67	10616-67	Podocalyxin-like protein 2	PODXL2	Q9NZ53	0.026337	-0.12354
seq.19122.47	19122-47	Myosin regulatory light chain 12B	MYL12B	O14950	0.026337	0.303998
seq.19202.10	19202-10	Tyrosine-protein kinase BTK	BTK	Q06187	0.026337	-0.12546
seq.20183.48	20183-48	Homeobox protein MOX-1	MEOX1	P50221	0.026337	-0.05744
seq.20247.17	20247-17	Dihydropyrimidinase-related protein 4	DPYSL4	O14531	0.026337	0.048109
seq.21572.91	21572-91	Xylosyltransferase 2	XYLT2	Q9H1B5	0.026337	-0.13294
seq.24646.47	24646-47	Serine--pyruvate aminotransferase	AGXT	P21549	0.026337	-0.10458
seq.24688.9	24688-9	Doublesex- and mab-3-related transcription factor B1	DMRTB1	Q96MA1	0.026337	-0.18189
seq.2622.18	2622-18	Heme oxygenase 2	HMOX2	P30519	0.026337	-0.09597
seq.3766.51	3766-51	Syntaxin-1A	STX1A	Q16623	0.026337	-0.0836
seq.5736.1	5736-1	Trem-like transcript 2 protein	TREML2	Q5T2D2	0.026337	-0.09394
seq.8336.267	8336-267	GRAM domain-containing protein 1C	GRAMD1C	Q8IYS0	0.026337	-0.07996
seq.8287.17	8287-17	CMRF35-like molecule 2	CD300E	Q496F6	0.027907	-0.0925
seq.11071.1	11071-1	Interleukin-5	IL5	P05113	0.027907	-0.09091
seq.11281.6	11281-6	Growth factor receptor-bound protein 7	GRB7	Q14451	0.027907	-0.0497
seq.11690.47	11690-47	Anaphase-promoting complex subunit 7	ANAPC7	Q9UJX3	0.027907	-0.09101
seq.12835.101	12835-101	Pyrin domain-containing protein 1	PYDC1	Q8WXC3	0.027907	-0.13852
seq.17348.5	17348-5	Synaptojanin-2-binding protein	SYNJ2BP	P57105	0.027907	-0.19024
seq.21690.31	21690-31	Leucine-rich repeat-containing protein 4	LRRC4	Q9HBW1	0.027907	-0.13173
seq.21891.31	21891-31	Fibulin-7	FBLN7	Q53RD9	0.027907	0.242728
seq.22137.3	22137-3	Protein FAM102B	FAM102B	Q5T8I3	0.027907	-0.14496
seq.23652.15	23652-15	Peroxisomal sarcosine oxidase	PIPOX	Q9P0Z9	0.027907	-0.10871
seq.2447.7	2447-7	Group IIE secretory phospholipase A2	PLA2G2E	Q9NZK7	0.027907	-0.12303
seq.24640.63	24640-63	Hairy/enhancer-of-split related with YRPW motif protein 1	HEY1	Q9Y5J3	0.027907	-0.08104
seq.5204.13	5204-13	Proteasome activator complex subunit 3	PSME3	P61289	0.027907	-0.07756
seq.5915.58	5915-58	Peroxisomal targeting signal 1 receptor	PEX5	P50542	0.027907	-0.09741
seq.7997.118	7997-118	Double C2-like domain-containing protein beta	DOC2B	Q14184	0.027907	-0.1063
seq.9221.6	9221-6	Inositol 1,4,5-trisphosphate receptor-interacting protein-like 1	ITPRIPL1	Q6GPH6	0.027907	0.105555
seq.12445.50	12445-50	Ankyrin repeat domain-containing protein 27	ANKRD27	Q96NW4	0.027907	-0.06906
seq.20934.13	20934-13	Ras-related protein Rab-24	RAB24	Q969Q5	0.027907	0.2125
seq.21706.29	21706-29	Leukocyte immunoglobulin-like receptor subfamily A member 1	LILRA1	O75019	0.027907	-0.13068
seq.3758.63	3758-63	Activated Protein C	PROC	P04070	0.027907	-0.10298
seq.6495.14	6495-14	Endothelin-1	EDN1	P05305	0.027907	-0.10715
seq.8376.25	8376-25	Lutropin subunit beta	LHB	P01229	0.027907	-0.10052
seq.8429.16	8429-16	Axin-2	AXIN2	Q9Y2T1	0.027907	-0.13281
seq.10513.13	10513-13	Calsenilin	KCNIP3	Q9Y2W7	0.028018	-0.13407
seq.10978.39	10978-39	Growth hormone variant	GH2	P01242	0.028018	-0.12471
seq.11200.52	11200-52	Complement component C1q receptor	CD93	Q9NPY3	0.028018	-0.04774
seq.11549.6	11549-6	Insulin gene enhancer protein ISL-1	ISL1	P61371	0.028018	-0.07337
seq.12334.25	12334-25	Serine hydroxymethyltransferase, cytosolic	SHMT1	P34896	0.028018	0.254482
seq.12426.19	12426-19	MOB kinase activator 1A	MOB1A	Q9H8S9	0.028018	0.15194
seq.12784.10	12784-10	Amyloid beta A4 precursor protein-binding family B member 3:Phosphotyrosine Interaction Domain 1,Isoform II	APBB3	O95704	0.028018	-0.20937
seq.13511.29	13511-29	DNA-binding protein SATB1	SATB1	Q01826	0.028018	-0.10784
seq.14748.31	14748-31	Rho GTPase-activating protein 5	ARHGAP5	Q13017	0.028018	0.092977
seq.16828.8	16828-8	Collagen alpha-1(VI) chain	COL6A1	P12109	0.028018	0.504564
seq.18819.21	18819-21	Peptidyl-prolyl cis-trans isomerase C	PPIC	P45877	0.028018	0.504652
seq.18831.6	18831-6	Leucine-rich repeats and immunoglobulin-like domains protein 1	LRIG1	Q96JA1	0.028018	0.542285
seq.18832.65	18832-65	Serum amyloid A-2 protein	SAA2	P0DJI9	0.028018	-0.07014
seq.21535.5	21535-5	Alanyl-tRNA editing protein Aarsd1	AARSD1	Q9BTE6	0.028018	0.208847
seq.21653.205	21653-205	Ubiquitin-related modifier 1	URM1	Q9BTM9	0.028018	0.281361
seq.22990.20	22990-20	Protein lin-7 homolog B	LIN7B	Q9HAP6	0.028018	0.196117
seq.23364.3	23364-3	Uridine-cytidine kinase 1	UCK1	Q9HA47	0.028018	0.2589
seq.23568.41	23568-41	Homeobox protein Meis2	MEIS2	O14770	0.028018	0.433618
seq.24050.26	24050-26	Glycogen synthase kinase-3 beta	GSK3B	P49841	0.028018	0.902689
seq.24647.3	24647-3	C->U-editing enzyme APOBEC-2	APOBEC2	Q9Y235	0.028018	0.606628
seq.24911.57	24911-57	LRP	MVP	Q14764	0.028018	0.421111
seq.25216.8	25216-8	Calgranulin A	S100A8	P05109	0.028018	0.199112
seq.3466.8	3466-8	cAMP-dependent protein kinase catalytic subunit alpha	PRKACA	P17612	0.028018	0.548467
seq.4271.75	4271-75	Prefoldin subunit 5	PFDN5	Q99471	0.028018	0.490826
seq.5586.66	5586-66	Multiple inositol polyphosphate phosphatase 1	MINPP1	Q9UNW1	0.028018	0.328126
seq.5900.11	5900-11	Histidine triad nucleotide-binding protein 1	HINT1	P49773	0.028018	0.355941
seq.6055.53	6055-53	Interferon alpha/beta receptor 1	IFNAR1	P17181	0.028018	0.138722
seq.6060.2	6060-2	Prolactin-inducible protein	PIP	P12273	0.028018	-0.03484
seq.6433.57	6433-57	Pseudokinase FAM20A	FAM20A	Q96MK3	0.028018	-0.08795
seq.6444.15	6444-15	Pregnancy-specific beta-1-glycoprotein 3	PSG3	Q16557	0.028018	-0.10239
seq.6538.90	6538-90	Uncharacterized protein KIAA2013	KIAA2013	Q8IYS2	0.028018	-0.09409
seq.6597.24	6597-24	Membrane protein FAM174A	FAM174A	Q8TBP5	0.028018	-0.11576
seq.7262.191	7262-191	Carbohydrate sulfotransferase 14	CHST14	Q8NCH0	0.028018	-0.09118
seq.8005.1	8005-1	Matrix-remodeling-associated protein 7	MXRA7	P84157	0.028018	0.063661
seq.8957.72	8957-72	Endoplasmic reticulum lectin 1	ERLEC1	Q96DZ1	0.028018	-0.11836
seq.8975.26	8975-26	Cell differentiation protein RCD1 homolog	CNOT9	Q92600	0.028018	0.140988
seq.16609.106	16609-106	Kin of IRRE-like protein 2	KIRREL2	Q6UWL6	0.029036	-0.12282
seq.15603.20	15603-20	Integrin alpha-2	ITGA2	P17301	0.029036	-0.19022
seq.8970.9	8970-9	Receptor-interacting serine/threonine-protein kinase 2	RIPK2	O43353	0.029036	-0.06663
seq.10512.13	10512-13	Cytokine receptor common subunit beta:Extracellular domain	CSF2RB	P32927	0.029036	-0.11854

seq.3053.49	3053-49	Fms-related tyrosine kinase 3 ligand	FLT3LG	P49771	0.029036	-0.09697
seq.11279.42	11279-42	Gamma-aminobutyric acid type B receptor subunit 1	GABBR1	Q9UBS5	0.029036	-0.09383
seq.21967.20	21967-20	Corticosteroid 11-beta-dehydrogenase isozyme 1	HSD11B1	P28845	0.029036	-0.11647
seq.8989.40	8989-40	Signal peptide, CUB and EGF-like domain-containing protein 1	SCUBE1	Q8IWY4	0.029036	0.211336
seq.9606.4	9606-4	Nuclear pore membrane glycoprotein 210-like	NUP210L	Q5VU65	0.029036	-0.13126
seq.10907.116	10907-116	Neurotrimin	NTM	Q9P121	0.029036	-0.08452
seq.13690.26	13690-26	Biglycan	BGN	P21810	0.029036	0.237324
seq.17702.53	17702-53	UDP-glucuronosyltransferase 1-1	UGT1A1	P22309	0.029036	0.112406
seq.18240.6	18240-6	Olfactory marker protein	OMP	P47874	0.029036	-0.1457
seq.18289.16	18289-16	C-C motif chemokine 15	CCL15	Q16663	0.029036	0.050457
seq.9075.121	9075-121	UPF0258 protein KIAA1024	MINAR1	Q9UPX6	0.029036	-0.10255
seq.12357.41	12357-41	Synaptosomal-associated protein 29	SNAP29	O95721	0.029036	-0.08921
seq.12711.19	12711-19	Gap junction alpha-8 protein	GJA8	P48165	0.029036	-0.08219
seq.13587.10	13587-10	Rac GTPase-activating protein 1	RACGAP1	Q9H0H5	0.029036	-0.11156
seq.15381.45	15381-45	Discoidin domain-containing receptor 2	DDR2	Q16832	0.029036	-0.09992
seq.15449.33	15449-33	T-cell immunoglobulin and mucin domain-containing protein 4	TIMD4	Q96H15	0.029036	-0.11533
seq.20106.80	20106-80	Scavenger mRNA-decapping enzyme DcpS	DCPS	Q96C86	0.029036	0.140442
seq.22950.6	22950-6	Butyrophilin subfamily 3 member A3	BTN3A3	O00478	0.029036	-0.04251
seq.25124.21	25124-21	Zinc finger and BTB domain-containing protein 7A	ZBTB7A	O95365	0.029036	-0.06765
seq.25296.3	25296-3	Angiomotin	AMOT	Q4VCS5	0.029036	-0.06495
seq.3296.92	3296-92	Contactin-2	CNTN2	Q02246	0.029036	-0.07516
seq.4973.18	4973-18	Baculoviral IAP repeat-containing protein 3	BIRC3	Q13489	0.029036	-0.20876
seq.5089.11	5089-11	Interleukin-7 receptor subunit alpha	IL7R	P16871	0.029036	-0.10311
seq.7132.55	7132-55	Complement C1q-like protein 4	C1QL4	Q86Z23	0.029036	-0.0822
seq.8079.39	8079-39	EP300-interacting inhibitor of differentiation 3	EID3	Q8N140	0.029036	-0.09921
seq.9950.229	9950-229	Lymphocyte activation gene 3 protein	LAG3	P18627	0.029036	-0.09506
seq.24462.4	24462-4	Cyclic nucleotide-gated cation channel beta-1	CNGB1	Q14028	0.030822	-0.10104
seq.19258.24	19258-24	Glutaryl-CoA dehydrogenase, mitochondrial	GCDH	Q92947	0.030822	-0.05854
seq.3761.4	3761-4	Prostaglandin G/H synthase 2	PTGS2	P35354	0.030822	-0.08025
seq.10949.59	10949-59	60S acidic ribosomal protein P2	RPLP2	P05387	0.030822	-0.06809
seq.17387.27	17387-27	NF-kappa-B inhibitor beta	NFKBIB	Q15653	0.030822	-0.13444
seq.17829.2	17829-2	Three prime repair exonuclease 2	TREX2	Q9BQ50	0.030822	-0.08809
seq.5623.11	5623-11	CMRF35-like molecule 1	CD300LF	Q8TDQ1	0.030822	-0.06095
seq.5671.1	5671-1	Chymotrypsinogen B	CTRB1	P17538	0.030822	-0.06852
seq.5679.16	5679-16	Beta-defensin 103	DEFB103A	P81534	0.030822	-0.06632
seq.6024.68	6024-68	Carboxypeptidase E	CPE	P16870	0.030822	-0.13646
seq.7253.6	7253-6	Protein quaking	QKI	Q96PU8	0.030822	-0.10203
seq.8364.74	8364-74	Uronyl 2-sulfotransferase	UST	Q9Y2C2	0.030822	-0.07454
seq.9585.80	9585-80	CXADR-like membrane protein:Cytoplasmic domain	CLMP	Q9H6B4	0.030822	-0.12487
seq.14136.234	14136-234	Complement component C1q receptor	CD93	Q9NPY3	0.030822	-0.08792
seq.21895.36	21895-36	Interferon lambda-4	None	K9M1U5	0.030822	0.06495
seq.5611.56	5611-56	Beta-defensin 108B	DEFB108B	Q8NET1	0.030822	-0.10692
seq.7826.1	7826-1	Serine/threonine-protein kinase DCLK3	DCLK3	Q9C098	0.030822	-0.07812
seq.10082.251	10082-251	Neurofilament light polypeptide	NEFL	P07196	0.031581	0.588733
seq.11128.29	11128-29	Transmembrane protein 132C:Cytoplasmic domain	TMEM132C	Q8N3T6	0.031581	0.216194
seq.13524.25	13524-25	Heparan-sulfate 6-O-sulfotransferase 2	HS6ST2	Q96MM7	0.031581	-0.08627
seq.14663.44	14663-44	E3 ubiquitin-protein ligase RNF8	RNF8	O76064	0.031581	0.159403
seq.18291.8	18291-8	Cyclin-dependent kinase inhibitor 1	CDKN1A	P38936	0.031581	0.075118
seq.19177.7	19177-7	MOB kinase activator 1B	MOB1B	Q7L9L4	0.031581	0.571836
seq.19361.78	19361-78	Matrilin-3	MATN3	O15232	0.031581	-0.29637
seq.21132.9	21132-9	Synaptotagmin-13	SYT13	Q7L8C5	0.031581	-0.08505
seq.21160.4	21160-4	Charged multivesicular body protein 6	CHMP6	Q96FZ7	0.031581	0.221822
seq.21191.24	21191-24	Dual specificity protein phosphatase 21	DUSP21	Q9H596	0.031581	0.079039
seq.21786.25	21786-25	Cyclin-dependent kinase-like 2	CDKL2	Q92772	0.031581	0.213642
seq.22484.17	22484-17	Casein kinase I isoform alpha-like	CSNK1A1L	Q8N752	0.031581	0.183144
seq.2278.61	2278-61	Metalloproteinase inhibitor 2	TIMP2	P16035	0.031581	0.751744
seq.23340.37	23340-37	Methylthioribose-1-phosphate isomerase	MRI1	Q9BV20	0.031581	0.285095
seq.23567.37	23567-37	Guanine nucleotide-binding protein G(q) subunit alpha	GNAQ	P50148	0.031581	0.35053
seq.23640.10	23640-10	EH domain-containing protein 2	EHD2	Q9NZN4	0.031581	0.38352
seq.24954.83	24954-83	AP-1 complex subunit beta-1	AP1B1	Q10567	0.031581	0.20861
seq.24967.4	24967-4	Protocadherin-1	PCDH1	Q08174	0.031581	-0.53253
seq.25499.37	25499-37	Phospholipase DDHD2	DDHD2	O94830	0.031581	0.15566
seq.25922.7	25922-7	Low-density lipoprotein receptor-related protein 5	LRP5	O75197	0.031581	0.0915
seq.2981.9	2981-9	Endothelial cell-selective adhesion molecule	ESAM	Q96AP7	0.031581	-0.14793
seq.3325.2	3325-2	Matrilin-2	MATN2	O00339	0.031581	-0.36436
seq.5758.49	5758-49	Kallikrein-9	KLK9	Q9UKQ9	0.031581	-0.06327
seq.6342.10	6342-10	Nephronectin	NPNT	Q6UXI9	0.031581	0.641464
seq.7806.33	7806-33	Beta-1,4-galactosyltransferase 7	B4GALT7	Q9UBV7	0.031581	0.19418
seq.8838.10	8838-10	Protein CASC4	GOLM2	Q6P4E1	0.031581	-0.06671
seq.8043.153	8043-153	Cartilage oligomeric matrix protein	COMP	P49747	0.03203	-0.13497
seq.10667.78	10667-78	Uncharacterized protein C1orf185	C1orf185	Q5T7R7	0.03203	-0.14595
seq.10917.40	10917-40	Guanine nucleotide-binding protein G(I)/G(S)/G(O) subunit gamma-T2	GNGT2	O14610	0.03203	-0.12063
seq.25264.102	25264-102	SHIP	INPP5D	Q92835	0.03203	-0.12267
seq.2618.10	2618-10	Receptor tyrosine-protein kinase erbB-4	ERBB4	Q15303	0.03203	-0.16976
seq.3329.14	3329-14	Peptidoglycan recognition protein 1	PGLYRP1	O75594	0.03203	-0.09636
seq.9443.137	9443-137	Cathepsin K	CTSK	P43235	0.03203	-0.15134
seq.12605.1	12605-1	Exosome complex component RRP40	EXOSC3	Q9NQT5	0.03203	-0.08994
seq.16618.7	16618-7	Early activation antigen CD69	CD69	Q07108	0.03203	-0.11639
seq.4540.11	4540-11	Chromobox protein homolog 5	CBX5	P45973	0.03203	-0.09752
seq.7202.107	7202-107	Semaphorin-6C	SEMA6C	Q9H3T2	0.03203	-0.13728
seq.15565.102	15565-102	Mucin-16	MUC16	Q8WXI7	0.03203	-0.19387
seq.3073.51	3073-51	Interleukin-18-binding protein	IL18BP	O95998	0.03203	-0.05441
seq.4479.14	4479-14	Plasma protease C1 inhibitor	SERPING1	P05155	0.03203	-0.08919
seq.8976.13	8976-13	Beta-1,4 N-acetylgalactosaminyltransferase 1	B4GALNT1	Q00973	0.03203	-0.06771
seq.9050.170	9050-170	Arginine/serine-rich protein 1	RSRP1	Q9BUV0	0.03203	-0.08854
seq.14122.132	14122-132	E3 ubiquitin-protein ligase ZNRF3	ZNRF3	Q9ULT6	0.03203	-0.07486

seq.22417.10	22417-10	E3 ubiquitin-protein ligase CBL-C	CBLC	Q9ULV8	0.03203	-0.09191
seq.24942.22	24942-22	Protein unc-13 homolog D	UNC13D	Q70J99	0.03203	-0.08923
seq.2853.68	2853-68	Serine/threonine-protein kinase Chk1	CHEK1	O14757	0.03203	0.15318
seq.3322.52	3322-52	Leucine-rich repeats and immunoglobulin-like domains protein 3	LRIG3	Q6UXM1	0.03203	-0.11751
seq.9873.17	9873-17	Steroid hormone receptor ERR1	ESRRA	P11474	0.03203	0.054994
seq.10048.7	10048-7	Core-binding factor subunit beta	CBFB	Q13951	0.03203	0.126561
seq.10089.7	10089-7	N-acetylserotonin O-methyltransferase-like protein	ASMTL	O95671	0.03203	0.083977
seq.10439.57	10439-57	Alpha-amylase 2B	AMY2B	P19961	0.03203	-0.07933
seq.10916.44	10916-44	Secretory phospholipase A2 receptor	PLA2R1	Q13018	0.03203	-0.2158
seq.10981.56	10981-56	Pro-neuregulin-3, membrane-bound isoform	NRG3	P56975	0.03203	-0.17734
seq.12849.25	12849-25	GSK3-beta interaction protein	GSKIP	Q9P0R6	0.03203	-0.08145
seq.13229.20	13229-20	Protein Mdm4	MDM4	O15151	0.03203	0.051697
seq.13387.55	13387-55	ETS homologous factor	EHF	Q9NZC4	0.03203	-0.0882
seq.13539.131	13539-131	Small conductance calcium-activated potassium channel protein 1	KCNN1	Q92952	0.03203	-0.08192
seq.19768.13	19768-13	Cystatin B	CSTB	P04080	0.03203	0.29151
seq.22099.1	22099-1	Uncharacterized protein C4orf36	C4orf36	Q96KX1	0.03203	-0.09511
seq.24273.43	24273-43	Thioredoxin domain-containing protein 3	NME8	Q8N427	0.03203	-0.09408
seq.2436.49	2436-49	C-X-C motif chemokine 16	CXCL16	Q9H2A7	0.03203	-0.09436
seq.7757.5	7757-5	HLA class II histocompatibility antigen, DQ alpha 2 chain	HLA-DQA2	P01906	0.03203	-0.08339
seq.7792.58	7792-58	Coiled-coil domain-containing protein 90B, mitochondrial	CCDC90B	Q9GZT6	0.03203	-0.13324
seq.8818.13	8818-13	Interferon gamma receptor 2:Cytoplasmic domain	IFNGR2	P38484	0.03203	-0.08918
seq.9474.22	9474-22	Asialoglycoprotein receptor 2	ASGR2	P07307	0.03203	-0.1462
seq.9506.10	9506-10	Apolipoprotein L1	APOL1	O14791	0.03203	0.098517
seq.9514.46	9514-46	EF-hand calcium-binding domain-containing protein 14:C-term	EFCAB14	O75071	0.03203	0.241413
seq.9536.16	9536-16	Epididymal secretory protein E3-alpha	EDDM3A	Q14507	0.03203	0.302025
seq.10756.34	10756-34	Urocortin-3	UCN3	Q969E3	0.033831	-0.07376
seq.11547.84	11547-84	Muscle, skeletal receptor tyrosine-protein kinase	MUSK	O15146	0.033831	-0.07081
seq.3808.76	3808-76	Fibroblast growth factor receptor 2	FGFR2	P21802	0.033831	-0.0872
seq.9055.81	9055-81	Myocardial zonula adherens protein	MYZAP	P0CAP1	0.033831	-0.08798
seq.19578.19	19578-19	Docking protein 2	DOK2	O60496	0.033831	-0.0899
seq.3376.49	3376-49	Interleukin-17 receptor D	IL17RD	Q8NFM7	0.033831	-0.1286
seq.9123.18	9123-18	Protein Dos	CBARP	Q8N350	0.033831	-0.17126
seq.10361.25	10361-25	2'-5'-oligoadenylate synthase 1	OAS1	P00973	0.033831	-0.11028
seq.12945.33	12945-33	Ras-related protein Rab-18	RAB18	Q9NP72	0.033831	0.418426
seq.13421.17	13421-17	Protein kish-B	TMEM167B	Q9NRX6	0.033831	-0.09694
seq.17336.54	17336-54	Cyclic AMP-responsive element-binding protein 3-like protein 2	CREB3L2	Q70SY1	0.033831	-0.14796
seq.19158.1	19158-1	PCNA-associated factor	PCLAF	Q15004	0.033831	-0.16321
seq.23545.6	23545-6	Nuclear distribution protein nudE-like 1	NDEL1	Q9GZM8	0.033831	0.158646
seq.24718.8	24718-8	Leucine-rich repeat-containing protein 75A	LRRC75A	Q8NAA5	0.033831	-0.09859
seq.7903.18	7903-18	Vesicle-associated membrane protein 3	VAMP3	Q15836	0.033831	-0.10904
seq.8892.14	8892-14	Platelet endothelial aggregation receptor 1:Cytoplasmic domain	PEAR1	Q5VY43	0.033831	-0.07614
seq.9573.108	9573-108	Thioredoxin domain-containing protein 11:N-term	TXNDC11	Q6PKC3	0.033831	-0.11892
seq.10620.21	10620-21	Beta-microseminoprotein	MSMB	P08118	0.033831	-0.10159
seq.11110.4	11110-4	Transmembrane protein 119	TMEM119	Q4V9L6	0.033831	-0.15926
seq.21339.19	21339-19	Alanine aminotransferase 2	GPT2	Q8TD30	0.033831	-0.06193
seq.21480.2	21480-2	CD82 antigen	CD82	P27701	0.033831	-0.08657
seq.21588.4	21588-4	BTB/POZ domain-containing protein KCTD4	KCTD4	Q8WVF5	0.033831	-0.16579
seq.24418.14	24418-14	Homeobox protein HMX3	HMX3	A6NHT5	0.033831	-0.11682
seq.2711.6	2711-6	Ciliary neurotrophic factor receptor subunit alpha	CNTFR	P26992	0.033831	-0.06622
seq.7825.7	7825-7	Monoacylglycerol lipase ABHD12	ABHD12	Q8N2K0	0.033831	-0.12221
seq.9097.5	9097-5	UPF0577 protein KIAA1324:Cytoplasmic domain	ELAPOR1	Q6UXG2	0.033831	-0.11363
seq.9125.23	9125-23	Mannan-binding lectin serine protease 1:Sushi 1 and Sushi 2	MASP1	P48740	0.033831	-0.14698
seq.9823.2	9823-2	Dihydrofolate reductase	DHFR	P00374	0.033831	0.115528
seq.11218.84	11218-84	Thiopurine S-methyltransferase	TPMT	P51580	0.034983	0.223469
seq.13955.33	13955-33	Death-associated protein kinase 1	DAPK1	P53355	0.034983	0.128364
seq.13972.4	13972-4	17-beta-hydroxysteroid dehydrogenase 14	HSD17B14	Q9BPX1	0.034983	-0.09022
seq.14013.11	14013-11	TRAF family member-associated NF-kappa-B activator	TANK	Q92844	0.034983	0.128812
seq.14157.21	14157-21	14-3-3 protein epsilon	YWHAE	P62258	0.034983	0.385793
seq.14684.17	14684-17	Calpain-2 catalytic subunit	CAPN2	P17655	0.034983	0.375207
seq.15331.47	15331-47	Histone-binding protein RBBP4	RBBP4	Q09028	0.034983	0.396367
seq.16535.61	16535-61	Green fluorescent protein_AEQVI	GFP	P42212	0.034983	-0.20833
seq.18265.18	18265-18	Zinc finger protein 34	ZNF34	Q8IZ26	0.034983	0.179825
seq.18945.11	18945-11	Regulator of G-protein signaling 1	RGS1	Q08116	0.034983	0.188701
seq.19579.5	19579-5	Neutrophil defensin 1	DEFA1	P59665	0.034983	-0.10599
seq.21135.16	21135-16	Splicing factor 3B subunit 6	SF3B6	Q9Y3B4	0.034983	0.150127
seq.22119.18	22119-18	COP9 signalosome complex subunit 8	COPS8	Q99627	0.034983	0.179263
seq.22374.56	22374-56	Hairy/enhancer-of-split related with YRPW motif protein 1	HEY1	Q9Y5J3	0.034983	-0.18433
seq.23361.20	23361-20	RNA-binding protein 4	RBM4	Q9BWF3	0.034983	0.887591
seq.23639.93	23639-93	Probable aminopeptidase NPEPL1	NPEPL1	Q8NDH3	0.034983	-0.14314
seq.24411.144	24411-144	E3 ubiquitin-protein ligase LRSAM1	LRSAM1	Q6UWE0	0.034983	0.525129
seq.24922.19	24922-19	NHL repeat-containing protein 2	NHLRC2	Q8NBF2	0.034983	0.1551
seq.24956.1	24956-1	N6-adenosine-methyltransferase 70 kDa subunit	METTL3	Q86U44	0.034983	0.172841
seq.25219.17	25219-17	V-type proton ATPase subunit F	ATP6V1F	Q16864	0.034983	0.455532
seq.25963.2	25963-2	Exportin-5	XPO5	Q9HAV4	0.034983	0.192553
seq.3009.3	3009-3	Transforming growth factor beta receptor type 3	TGFBR3	Q03167	0.034983	-0.3261
seq.3041.55	3041-55	C-type mannose receptor 2	MRC2	Q9UBG0	0.034983	0.36053
seq.3348.49	3348-49	Bone morphogenetic protein 1	BMP1	P13497	0.034983	0.179492
seq.3481.87	3481-87	Xaa-Pro aminopeptidase 1	XPNPEP1	Q9NQW7	0.034983	0.56547
seq.3889.64	3889-64	Lamin-B1	LMNB1	P20700	0.034983	-0.06975
seq.4693.72	4693-72	3-hydroxyisobutyrate dehydrogenase, mitochondrial	HIBADH	P31937	0.034983	-0.0894
seq.5346.24	5346-24	Copine-1:Ca2+-dependent membrane-targeting module domains 1 and 2	CPNE1	Q99829	0.034983	0.99753
seq.5717.2	5717-2	Cartilage intermediate layer protein 1	CILP	O75339	0.034983	0.507915
seq.5740.17	5740-17	Roundabout homolog 1	ROBO1	Q9Y6N7	0.034983	-0.16921

Disease Models & Mechanisms • Supplementary information

seq.6563.78	6563-78	Heat shock 70 kDa protein 1A	HSPA1A	P0DMV8	0.034983	0.119257
seq.6930.95	6930-95	Alpha-2,8-sialyltransferase 8F	ST8SIA6	P61647	0.034983	-0.09978
seq.7219.152	7219-152	Heat shock 70 kDa protein 1A	HSPA1A	P0DMV8	0.034983	-0.10891
seq.8018.43	8018-43	V-set and immunoglobulin domain-containing protein 2	VSIG2	Q96IQ7	0.034983	0.181054
seq.8061.102	8061-102	Protein FAM171B:Cytoplasmic domain	FAM171B	Q6P995	0.034983	-0.10783
seq.8390.25	8390-25	Cytochrome c oxidase subunit 7A1, mitochondrial	COX7A1	P24310	0.034983	-0.06869
seq.8397.147	8397-147	Sulfhydryl oxidase 2	QSOX2	Q6ZRP7	0.034983	0.09179
seq.9196.8	9196-8	Galectin-7	LGALS7	P47929	0.034983	0.123673
seq.9368.64	9368-64	Leucine-rich repeat and transmembrane domain-containing protein 1	LRTM1	Q9HBL6	0.034983	-0.07904
seq.9600.55	9600-55	Thrombospondin type-1 domain-containing protein 7A:Thrombospondin type-1 domain 17	THSD7A	Q9UPZ6	0.034983	-0.07608
seq.23000.22	23000-22	Neuroligin-1	NLGN1	Q8N2Q7	0.035016	-0.10271
seq.10832.24	10832-24	Beta-1,4-galactosyltransferase 6	B4GALT6	Q9UBX8	0.035016	-0.08711
seq.13517.3	13517-3	Patched domain-containing protein 3	PTCHD3	Q3KNS1	0.035016	-0.11423
seq.19615.213	19615-213	Cytosolic 5'-nucleotidase 3A	NT5C3A	Q9H0P0	0.035016	0.093629
seq.20946.41	20946-41	Gametocyte-specific factor 1	GTSF1	Q8WW33	0.035016	-0.13426
seq.7258.5	7258-5	Bone marrow proteoglycan	PRG2	P13727	0.035016	-0.13324
seq.12514.16	12514-16	tRNA (guanine-N(7)-)-methyltransferase	METTL1	Q9UBP6	0.035016	-0.05622
seq.13976.9	13976-9	Rho guanine nucleotide exchange factor 1	ARHGEF1	Q92888	0.035016	-0.07606
seq.15613.16	15613-16	Pancreatic triacylglycerol lipase	PNLIP	P16233	0.035016	-0.09328
seq.24486.1	24486-1	B-cell lymphoma/leukemia 11A	BCL11A	Q9H165	0.035016	-0.07086
seq.3415.61	3415-61	Bone sialoprotein 2	IBSP	P21815	0.035016	-0.05424
seq.3437.80	3437-80	Receptor-type tyrosine-protein kinase FLT3	FLT3	P36888	0.035016	0.119026
seq.3727.35	3727-35	Peptide YY	PYY	P10082	0.035016	-0.10777
seq.4913.78	4913-78	C-C motif chemokine 16	CCL16	O15467	0.035016	-0.08086
seq.9015.1	9015-1	Proteoglycan 3	PRG3	Q9Y2Y8	0.035016	-0.09282
seq.11375.49	11375-49	Forkhead box protein L2	FOXL2	P58012	0.035016	-0.07943
seq.12535.2	12535-2	DNA repair protein XRCC1	XRCC1	P18887	0.035016	-0.10291
seq.13666.222	13666-222	Carbonic anhydrase-related protein 10	CA10	Q9NS85	0.035016	-0.09513
seq.17774.38	17774-38	Small nuclear ribonucleoprotein Sm D3	SNRPD3	P62318	0.035016	-0.10848
seq.3404.51	3404-51	Tryptase gamma	TPSG1	Q9NRR2	0.035016	-0.09218
seq.3828.54	3828-54	Platelet-derived growth factor C	PDGFC	Q9NRA1	0.035016	-0.11877
seq.4904.7	4904-7	Caspase-2	CASP2	P42575	0.035016	-0.08539
seq.4929.55	4929-55	Sex hormone-binding globulin	SHBG	P04278	0.035016	-0.08032
seq.5628.21	5628-21	Semaphorin-3G	SEMA3G	Q9NS98	0.035016	-0.12697
seq.8660.33	8660-33	Olfactomedin-like protein 3	OLFML3	Q9NRN5	0.035016	0.099812
seq.11330.15	11330-15	Casein kinase II subunit beta	CSNK2B	P67870	0.035016	0.167714
seq.14063.17	14063-17	Oncostatin-M	OSM	P13725	0.035016	-0.06901
seq.17466.72	17466-72	Acyl-CoA synthetase family member 2, mitochondrial	ACSF2	Q96CM8	0.035016	0.000781
seq.17773.26	17773-26	Gamma-soluble NSF attachment protein	NAPG	Q99747	0.035016	0.168233
seq.18158.45	18158-45	Caspase-8	CASP8	Q14790	0.035016	-0.07478
seq.21548.20	21548-20	Gamma-glutamyltransferase 5	GGT5	P36269	0.035016	-0.06568
seq.22774.20	22774-20	tRNA-splicing endonuclease subunit Sen34	TSEN34	Q9BSV6	0.035016	0.221518
seq.24321.67	24321-67	Nuclear prelamin A recognition factor	NARF	Q9UHQ1	0.035016	-0.10913
seq.5110.84	5110-84	Neurexin-1-beta	NRXN1	P58400	0.035016	-0.09182
seq.5724.58	5724-58	Suprabasin	SBSN	Q6UWP8	0.035016	-0.07906
seq.6387.61	6387-61	Defensin-5	DEFA5	Q01523	0.035016	-0.10358
seq.6493.9	6493-9	Carboxypeptidase Z	CPZ	Q66K79	0.035016	-0.1158
seq.7947.19	7947-19	AP-4 complex accessory subunit tepsin	TEPSIN	Q96N21	0.035016	-0.10578
seq.7953.20	7953-20	Signaling lymphocytic activation molecule	SLAMF1	Q13291	0.035016	0.06642
seq.8388.24	8388-24	Spastin:Cytoplasmic domain	SPAST	Q9UBP0	0.035016	-0.09234
seq.8960.3	8960-3	Endoplasmic reticulum aminopeptidase 2	ERAP2	Q6P179	0.037129	-0.14327
seq.11708.2	11708-2	Lipocalin-1	LCN1	P31025	0.037129	-0.09107
seq.5060.62	5060-62	Programmed cell death 1 ligand 1	CD274	Q9NZQ7	0.037129	-0.08251
seq.8832.55	8832-55	Bone marrow stromal antigen 2	BST2	Q10589	0.037129	-0.08973
seq.19119.10	19119-10	DNA damage-inducible transcript 3 protein	DDIT3	P35638	0.037129	-0.1238
seq.5183.53	5183-53	AMP Kinase (alpha1beta1gamma1)	PRKAA1|PRKAB1|PRKAG1	Q13131|Q9Y478|P54619	0.037129	-0.06644
seq.11712.207	11712-207	Protein unc-45 homolog A	UNC45A	Q9H3U1	0.037129	-0.09363
seq.15530.33	15530-33	Ephrin type-B receptor 4	EPHB4	P54760	0.037129	-0.06335
seq.25491.54	25491-54	Endoplasmic reticulum junction formation protein lunapark	LNPK	Q9C0E8	0.037129	0.132378
seq.4906.35	4906-35	Coagulation Factor V	F5	P12259	0.037129	-0.16325
seq.8766.29	8766-29	Leukocyte immunoglobulin-like receptor subfamily A member 5	LILRA5	A6NI73	0.037129	-0.08846
seq.21528.12	21528-12	Bcl-2-modifying factor	BMF	Q96LC9	0.037129	-0.17498
seq.5813.58	5813-58	Erythropoietin	EPO	P01588	0.037129	-0.08819
seq.6525.17	6525-17	Dual specificity protein phosphatase 13 isoform A	DUSP13	Q6B8I1	0.037129	0.061878
seq.8659.68	8659-68	Protein G6b:Isoform B, Cytoplasmic domain	MPIG6B	O95866	0.037129	-0.10271
seq.9216.100	9216-100	Plexin-B2	PLXNB2	O15031	0.037129	0.2952
seq.23343.6	23343-6	Protein rogdi homolog	ROGDI	Q9GZN7	0.038811	-0.10298
seq.12352.70	12352-70	Arrestin domain-containing protein 3	ARRDC3	Q96B67	0.038811	-0.16081
seq.7905.30	7905-30	Haptoglobin isoform 2	HP	P00738	0.038811	-0.05145
seq.8444.3	8444-3	Magainin-2_XENLA	magainins	P11006	0.038811	-0.08173
seq.11537.12	11537-12	Transferrin receptor protein 2	TFR2	Q9UP52	0.038811	-0.05214
seq.19260.4	19260-4	PDZ domain-containing protein GIPC1	GIPC1	O14908	0.038811	-0.13783
seq.22123.15	22123-15	Homeobox protein DLX-2	DLX2	Q07687	0.038811	-0.19012
seq.5698.60	5698-60	Tenascin-X	TNXB	P22105	0.038811	-0.10605
seq.11130.158	11130-158	Voltage-dependent L-type calcium channel subunit beta-4	CACNB4	O00305	0.038811	0.31355
seq.12471.47	12471-47	Double-stranded RNA-binding protein Staufen homolog 1	STAU1	O95793	0.038811	0.308625
seq.13644.30	13644-30	Hepatocyte growth factor-regulated tyrosine kinase substrate	HGS	O14964	0.038811	0.317082
seq.16015.19	16015-19	Alanine aminotransferase 1	GPT	P24298	0.038811	0.114579
seq.18434.141	18434-141	TATA box-binding protein-like protein 1	TBPL1	P62380	0.038811	0.128092
seq.21674.132	21674-132	Butyrophilin subfamily 1 member A1	BTN1A1	Q13410	0.038811	-0.17075
seq.23571.93	23571-93	Coiled-coil domain-containing protein 94	YJU2	Q9BW85	0.038811	0.248773
seq.23967.8	23967-8	PAXIP1-associated protein 1	PAGR1	Q9BTK6	0.038811	0.164018
seq.25292.6	25292-6	Carboxylesterase 3	CES3	Q6UWW8	0.038811	0.131719

seq.2755.8	2755-8	C3a anaphylatoxin des Arginine	C3	P01024	0.038811	1.436045
seq.2975.19	2975-19	Connective tissue growth factor	CCN2	P29279	0.038811	0.218326
seq.3054.3	3054-3	Haptoglobin	HP	P00738	0.038811	-0.10656
seq.3221.54	3221-54	Secreted frizzled-related protein 1	SFRP1	Q8N474	0.038811	0.644295
seq.3292.75	3292-75	CD48 antigen	CD48	P09326	0.038811	-0.07841
seq.3832.51	3832-51	Protein-tyrosine kinase 6	PTK6	Q13882	0.038811	-0.07883
seq.3890.8	3890-8	L-lactate dehydrogenase B chain	LDHB	P07195	0.038811	0.255794
seq.4995.16	4995-16	15-hydroxyprostaglandin dehydrogenase [NAD(+)]	HPGD	P15428	0.038811	0.173674
seq.5062.60	5062-60	CD226 antigen	CD226	Q15762	0.038811	-0.07875
seq.5735.54	5735-54	C1GALT1-specific chaperone 1	C1GALT1C1	Q96EU7	0.038811	0.167375
seq.6951.26	6951-26	Acid-sensing ion channel 4	ASIC4	Q96FT7	0.038811	0.162804
seq.7243.8	7243-8	Interferon beta	IFNB1	P01574	0.038811	-0.07171
seq.8046.9	8046-9	Galectin-1	LGALS1	P09382	0.038811	0.159345
seq.8320.5	8320-5	SAFB-like transcription modulator	SLTM	Q9NWH9	0.038811	-0.09957
seq.9802.27	9802-27	Patatin-like phospholipase domain-containing protein 2	PNPLA2	Q96AD5	0.038811	0.077067
seq.16927.9	16927-9	Coagulation factor XIII	F13A1|F13B	P00488|P05160	0.038811	-0.09072
seq.18343.10	18343-10	Peroxisomal 2,4-dienoyl-CoA reductase	DECR2	Q9NUI1	0.038811	-0.14659
seq.6364.7	6364-7	Tapasin-related protein	TAPBPL	Q9BX59	0.038811	-0.0928
seq.6897.38	6897-38	Galactosylgalactosylxylosylprotein 3-beta-glucuronosyltransferase 3	B3GAT3	O94766	0.038811	-0.16254
seq.10351.51	10351-51	Interferon regulatory factor 1	IRF1	P10914	0.038811	0.101518
seq.14009.65	14009-65	Tumor necrosis factor alpha-induced protein 3	TNFAIP3	P21580	0.038811	0.060526
seq.22504.3	22504-3	NEDD4-binding protein 2-like 2	N4BP2L2	Q92802	0.038811	-0.10049
seq.5664.57	5664-57	Beta-defensin 106	DEFB106A	Q8N104	0.038811	-0.15792
seq.8318.13	8318-13	Sprouty-related, EVH1 domain-containing protein 1	SPRED1	Q7Z699	0.038811	-0.08426
seq.10069.2	10069-2	Peptidyl-prolyl cis-trans isomerase NIMA-interacting 1	PIN1	Q13526	0.038811	-0.08309
seq.11328.9	11328-9	U6 snRNA phosphodiesterase	USB1	Q9BQ65	0.038811	-0.09089
seq.12382.2	12382-2	Probable ATP-dependent RNA helicase DDX58	DDX58	O95786	0.038811	-0.18515
seq.13723.6	13723-6	Interleukin-10	IL10	P22301	0.038811	0.150404
seq.14294.61	14294-61	Methyl-CpG-binding domain protein 1	MBD1	Q9UIS9	0.038811	-0.04441
seq.21356.6	21356-6	B9 domain-containing protein 2	B9D2	Q9BPU9	0.038811	0.155727
seq.22797.20	22797-20	Transcription initiation factor TFIID subunit 12	TAF12	Q16514	0.038811	-0.16489
seq.24892.8	24892-8	Testicular spindle-associated protein SHCBP1L	SHCBP1L	Q9BZQ2	0.038811	-0.09655
seq.4135.84	4135-84	Immunoglobulin E	IGHE	P01854	0.038811	-0.11107
seq.6236.51	6236-51	Collagen triple helix repeat-containing protein 1	CTHRC1	Q96CG8	0.038811	-0.07639
seq.7108.7	7108-7	Small integral membrane protein 24	SMIM24	O75264	0.038811	-0.08337
seq.8933.84	8933-84	Myocyte-specific enhancer factor 2C	MEF2C	Q06413	0.038811	-0.06507
seq.10510.62	10510-62	SLP adapter and CSK-interacting membrane protein	SCIMP	Q6UWF3	0.041076	-0.1112
seq.19557.3	19557-3	Beta-klotho	KLB	Q86Z14	0.041076	-0.09085
seq.20557.19	20557-19	V-set and immunoglobulin domain-containing protein 8	VSIG8	P0DPA2	0.041076	-0.1799
seq.25088.42	25088-42	Phosphorylase b kinase regulatory subunit alpha, skeletal muscle isoform	PHKA1	P46020	0.041076	-0.09588
seq.9218.7	9218-7	Tumor necrosis factor receptor superfamily member 6	FAS	P25445	0.041076	-0.0923
seq.14081.5	14081-5	NKG2D ligand 1	ULBP1	Q9BZM6	0.041076	-0.06936
seq.10362.35	10362-35	Myc proto-oncogene protein	MYC	P01106	0.041076	-0.19811
seq.10855.55	10855-55	Plexin-B2	PLXNB2	O15031	0.041076	-0.06946
seq.8229.1	8229-1	Glucoside xylosyltransferase 1	GXYLT1	Q4G148	0.041076	-0.15383
seq.12763.69	12763-69	Zinc finger protein 334	ZNF334	Q9HCZ1	0.041076	-0.08216
seq.17153.46	17153-46	Killer cell immunoglobulin-like receptor 2DL3	KIR2DL3	P43628	0.041076	-0.07759
seq.18302.204	18302-204	Protein zwilch homolog	ZWILCH	Q9H900	0.041076	0.091915
seq.20927.43	20927-43	General transcription factor IIF subunit 2	GTF2F2	P13984	0.041076	-0.19186
seq.25080.16	25080-16	UDP-glucuronosyltransferase 2B15	UGT2B15	P54855	0.041076	-0.13923
seq.4542.24	4542-24	Clusterin	CLU	P10909	0.041076	-0.09532
seq.5699.19	5699-19	Protein FAM189A2	FAM189A2	Q15884	0.041076	0.167378
seq.8391.12	8391-12	Beta-defensin 115	DEFB115	Q30KQ5	0.041076	-0.09753
seq.24659.6	24659-6	DnaJ homolog subfamily B member 13	DNAJB13	P59910	0.043218	-0.04771
seq.11629.36	11629-36	TNF receptor-associated factor 4	TRAF4	Q9BUZ4	0.043218	-0.07151
seq.19241.31	19241-31	Retinol-binding protein 5	RBP5	P82980	0.043218	-0.07608
seq.7775.15	7775-15	Kallikrein-11	KLK11	Q9UBX7	0.043218	-0.06904
seq.8874.53	8874-53	Ceroid-lipofuscinosis neuronal protein 5:Lumenal domain	CLN5	O75503	0.043218	-0.06784
seq.22395.7	22395-7	Tristetraproline	ZFP36	P26651	0.043218	0.10409
seq.18311.44	18311-44	Dynactin subunit 6	DCTN6	O00399	0.043218	0.506307
seq.9774.59	9774-59	RING finger protein 150	RNF150	Q9ULK6	0.043218	-0.10895
seq.11282.16	11282-16	Macrophage scavenger receptor types I and II:Extracellular domain	MSR1	P21757	0.043218	-0.07426
seq.11285.8	11285-8	Hematopoietic progenitor cell antigen CD34	CD34	P28906	0.043218	-0.09749
seq.15384.15	15384-15	Klotho	KL	Q9UEF7	0.043218	-0.08584
seq.21943.170	21943-170	Protein Wnt-16	WNT16	Q9UBV4	0.043218	-0.07082
seq.22369.12	22369-12	Doublesex- and mab-3-related transcription factor C2	DMRTC2	Q8IXT2	0.043218	0.066427
seq.23696.256	23696-256	tRNA (adenine(58)-N(1))-methyltransferase non-catalytic subunit TRM6	TRMT6	Q9UJA5	0.043218	0.067462
seq.6550.4	6550-4	Intercellular adhesion molecule 4	ICAM4	Q14773	0.043218	-0.10201
seq.7006.4	7006-4	Collagen alpha-1(XXV) chain	COL25A1	Q9BXS0	0.043218	-0.10227
seq.7082.2	7082-2	UDP-GlcNAc:betaGal beta-1,3-N-acetylglucosaminyltransferase 6	B3GNT6	Q6ZMB0	0.043218	-0.05095
seq.8008.28	8008-28	Activator of apoptosis harakiri	HRK	O00198	0.043218	0.054113
seq.8042.88	8042-88	Serine protease inhibitor Kazal-type 9	SPINK9	Q5DT21	0.043218	-0.07583
seq.8268.98	8268-98	Heparan sulfate glucosamine 3-O-sulfotransferase 3A1	HS3ST3A1	Q9Y663	0.043218	0.093809
seq.9795.9	9795-9	Ceroid-lipofuscinosis neuronal protein 5:Cytoplasmic domain	CLN5	O75503	0.043218	-0.07153
seq.10370.21	10370-21	Signal transducer and activator of transcription 1-alpha/beta	STAT1	P42224	0.043218	0.390803
seq.11245.43	11245-43	Filamin-A:Calponin Homology 1	FLNA	P21333	0.043218	0.132468
seq.11257.1	11257-1	Dihydropteridine reductase	QDPR	P09417	0.043218	0.541962
seq.11311.79	11311-79	V(D)J recombination-activating protein 1	RAG1	P15918	0.043218	-0.0816
seq.12457.10	12457-10	1,2-dihydroxy-3-keto-5-methylthiopentene dioxygenase	ADI1	Q9BV57	0.043218	0.27782
seq.12500.88	12500-88	SUMO-activating enzyme subunit 2	UBA2	Q9UBT2	0.043218	0.569919
seq.12595.11	12595-11	Tropomodulin-1	TMOD1	P28289	0.043218	0.400195

seq.14042.11	14042-11	Secreted frizzled-related protein 1	SFRP1	Q8N474	0.043218	0.621497
seq.15299.102	15299-102	LDLR chaperone MESD	MESD	Q14696	0.043218	0.233646
seq.17784.23	17784-23	Glia maturation factor beta	GMFB	P60983	0.043218	0.405268
seq.18932.84	18932-84	Nicotinamide/nicotinic acid mononucleotide adenylyltransferase 1	NMNAT1	Q9HAN9	0.043218	-0.08482
seq.19272.9	19272-9	Peflin	PEF1	Q9UBV8	0.043218	0.223006
seq.19373.3	19373-3	Myosin regulatory light chain 12A	MYL12A	P19105	0.043218	0.352373
seq.20383.31	20383-31	Probable RNA-binding protein 18	RBM18	Q96H35	0.043218	0.072393
seq.21122.3	21122-3	Dysbindin domain-containing protein 2	DBNDD2	Q9BQY9	0.043218	0.095289
seq.21441.20	21441-20	gp75	TYRP1	P17643	0.043218	0.152222
seq.21708.149	21708-149	IgG receptor FcRn large subunit p51	FCGRT	P55899	0.043218	0.450821
seq.22050.19	22050-19	KxDL motif-containing protein 1	KXD1	Q9BQD3	0.043218	-0.13453
seq.22525.9	22525-9	Paired box protein Pax-3	PAX3	P23760	0.043218	-0.18443
seq.22985.160	22985-160	Insulin-like growth factor-binding protein 2	IGFBP2	P18065	0.043218	1.116156
seq.23037.37	23037-37	Ubiquitin-conjugating enzyme E2 D3	UBE2D3	P61077	0.043218	0.068344
seq.23162.36	23162-36	Mitogen-activated protein kinase kinase kinase 10	MAP3K10	Q02779	0.043218	0.182064
seq.23374.42	23374-42	N-terminal EF-hand calcium-binding protein 3	NECAB3	Q96P71	0.043218	0.22317
seq.24323.43	24323-43	BRO1 domain-containing protein BROX	BROX	Q5VW32	0.043218	-0.06358
seq.24490.16	24490-16	THAP domain-containing protein 4	THAP4	Q8WY91	0.043218	0.706845
seq.24635.25	24635-25	Trafficking protein particle complex subunit 6A	TRAPPC6A	O75865	0.043218	-0.06611
seq.24697.48	24697-48	Heterogeneous nuclear ribonucleoprotein A0	HNRNPA0	Q13151	0.043218	1.549162
seq.25093.96	25093-96	Vang-like protein 1	VANGL1	Q8TAA9	0.043218	-0.07316
seq.2590.69	2590-69	Inactive tyrosine-protein kinase transmembrane receptor ROR1	ROR1	Q01973	0.043218	0.196113
seq.3179.51	3179-51	Lysosomal protective protein	CTSA	P10619	0.043218	0.568496
seq.3312.64	3312-64	High affinity immunoglobulin gamma Fc receptor I	FCGR1A	P12314	0.043218	-0.08531
seq.3394.81	3394-81	Serine/threonine-protein kinase PLK1	PLK1	P53350	0.043218	-0.08094
seq.3896.5	3896-5	Phosphoglycerate mutase 1	PGAM1	P18669	0.043218	0.339762
seq.5493.17	5493-17	Serine/threonine-protein kinase WNK3	WNK3	Q9BYP7	0.043218	0.590798
seq.5852.6	5852-6	Protein S100-A12	S100A12	P80511	0.043218	-0.05383
seq.6467.65	6467-65	Protein O-glucosyltransferase 1	POGLUT1	Q8NBL1	0.043218	0.34321
seq.7015.8	7015-8	Leukocyte immunoglobulin-like receptor subfamily B member 5	LILRB5	O75023	0.043218	0.031089
seq.7136.107	7136-107	Hemoglobin subunit epsilon	HBE1	P02100	0.043218	-0.05279
seq.7210.25	7210-25	Amyloid-like protein 1	APLP1	P51693	0.043218	0.114803
seq.7761.125	7761-125	Choline/ethanolamine kinase	CHKB	Q9Y259	0.043218	0.29751
seq.8459.10	8459-10	Bone morphogenetic protein 6	BMP6	P22004	0.043218	0.374237
seq.9116.28	9116-28	HEPACAM family member 2:Isoform 1, Cytoplasmic domain	HEPACAM2	A8MVW5	0.043218	-0.05167
seq.20074.3	20074-3	Laminin subunit alpha-3	LAMA3	Q16787	0.045069	-0.06543
seq.8778.3	8778-3	Noggin	NOG	Q13253	0.045069	-0.11914
seq.18413.24	18413-24	ADP-ribosylation factor-like protein 4D	ARL4D	P49703	0.045069	-0.11339
seq.12366.16	12366-16	Gamma-crystallin D	CRYGD	P07320	0.045069	-0.087
seq.11989.35	11989-35	ER membrane protein complex subunit 1	EMC1	Q8N766	0.045069	-0.08698
seq.20547.5	20547-5	Neuronal acetylcholine receptor subunit beta-3	CHRNB3	Q05901	0.045069	-0.15117
seq.21811.20	21811-20	Nucleolar protein of 40 kDa	ZCCHC17	Q9NP64	0.045069	-0.15148
seq.24899.13	24899-13	Epidermal growth factor receptor kinase substrate 8-like protein 1	EPS8L1	Q8TE68	0.045069	0.069585
seq.24955.116	24955-116	Zinc fingers and homeoboxes protein 2	ZHX2	Q9Y6X8	0.045069	-0.29493
seq.25452.16	25452-16	Myb/SANT-like DNA-binding domain-containing protein 2	MSANTD2	Q6P1R3	0.045069	0.13947
seq.5106.52	5106-52	Neurogenic locus notch homolog protein 2	NOTCH2	Q04721	0.045069	-0.07843
seq.8089.173	8089-173	Nuclear receptor subfamily 4 group A member 1	NR4A1	P22736	0.045069	-0.06198
seq.9100.32	9100-32	Uncharacterized protein C5orf46	C5orf46	Q6UWT4	0.045069	0.052074
seq.13097.11	13097-11	Bcl-2-like protein 2	BCL2L2	Q92843	0.045069	-0.08382
seq.15506.34	15506-34	Low-density lipoprotein receptor-related protein 12	LRP12	Q9Y561	0.045069	-0.14358
seq.16802.31	16802-31	OCIA domain-containing protein 1	OCIAD1	Q9NX40	0.045069	-0.05839
seq.22980.37	22980-37	Homer protein homolog 1	HOMER1	Q86YM7	0.045069	0.416971
seq.23246.67	23246-67	Biogenesis of lysosome-related organelles complex 1 subunit 1	BLOC1S1	P78537	0.045069	0.197389
seq.25414.11	25414-11	Late cornified envelope protein 3B	LCE3B	Q5TA77	0.045069	-0.07873
seq.3421.54	3421-54	Tumor necrosis factor ligand superfamily member 8	TNFSF8	P32971	0.045069	-0.09063
seq.4397.26	4397-26	Interferon lambda-2	IFNL2	Q8IZJ0	0.045069	-0.07926
seq.6390.18	6390-18	Neuropeptide S	NPS	P0C0P6	0.045069	-0.08192
seq.8066.38	8066-38	Synaptotagmin-9	SYT9	Q86SS6	0.045069	-0.11705
seq.9576.58	9576-58	Motor neuron and pancreas homeobox protein 1	MNX1	P50219	0.045069	-0.12226
seq.9826.135	9826-135	Bis(5'-adenosyl)-triphosphatase	FHIT	P49789	0.045069	0.248995
seq.22392.5	22392-5	SERTA domain-containing protein 3	SERTAD3	Q9UJW9	0.047316	-0.20323
seq.11260.47	11260-47	SUN domain-containing protein 5	SUN5	Q8TC36	0.047316	-0.09055
seq.3512.72	3512-72	Adenylate kinase_GEOSE	adk	P27142	0.047316	-0.14707
seq.23254.31	23254-31	NADH dehydrogenase [ubiquinone] 1 alpha subcomplex assembly factor 3	NDUFAF3	Q9BU61	0.047316	-0.09923
seq.24680.51	24680-51	Thioredoxin domain-containing protein 1	TMX1	Q9H3N1	0.047316	-0.05581
seq.4557.61	4557-61	Kin of IRRE-like protein 3	KIRREL3	Q8IZU9	0.047316	-0.11139
seq.10015.119	10015-119	Voltage-gated potassium channel subunit beta-2	KCNAB2	Q13303	0.047316	-0.07453
seq.25481.66	25481-66	Ataxin-10	ATXN10	Q9UBB4	0.047316	0.083739
seq.3025.50	3025-50	Fibroblast growth factor 2	FGF2	P09038	0.047316	0.250352
seq.6571.75	6571-75	Uncharacterized protein C17orf78:N-term	C17orf78	Q8N4C9	0.047316	-0.10677
seq.11395.5	11395-5	Protein-tyrosine kinase 2-beta:4.1 protein, ezrin, radixin, moesin domain	PTK2B	Q14289	0.047316	-0.05575
seq.12860.7	12860-7	cAMP-regulated phosphoprotein 21	ARPP21	Q9UBL0	0.047316	-0.12458
seq.17152.10	17152-10	Killer cell immunoglobulin-like receptor 2DS4	KIR2DS4	P43632	0.047316	-0.12548
seq.6210.100	6210-100	Interferon alpha-5	IFNA5	P01569	0.047316	-0.07013
seq.6214.84	6214-84	Interferon alpha-8	IFNA8	P32881	0.047316	-0.08826
seq.6577.64	6577-64	Laminin subunit alpha-4	LAMA4	Q16363	0.047316	-0.07196
seq.9232.1	9232-1	Roundabout homolog 4	ROBO4	Q8WZ75	0.047316	-0.12115
seq.10514.5	10514-5	Prostaglandin-H2 D-isomerase	PTGDS	P41222	0.047316	-0.04931
seq.11147.17	11147-17	Trem-like transcript 1 protein:immunoreceptor tyrosine-based inhibition motif	TREML1	Q86YW5	0.047316	-0.07914
seq.11383.41	11383-41	Keratin, type II cytoskeletal 7	KRT7	P08729	0.047316	0.162954

Disease Models & Mechanisms • Supplementary information

seq.11643.73	11643-73	E3 ubiquitin-protein ligase DTX3L	DTX3L	Q8TDB6	0.047316	-0.07592
seq.20546.71	20546-71	Reticulon-4 receptor-like 1	RTN4RL1	Q86UN2	0.047316	0.38942
seq.20553.2	20553-2	Phosphoinositide-3-kinase-interacting protein 1	PIK3IP1	Q96FE7	0.047316	-0.16186
seq.21204.70	21204-70	Desumoylating isopeptidase 1	DESI1	Q6ICB0	0.047316	0.138954
seq.23555.11	23555-11	GTP-binding protein RAD	RRAD	P55042	0.047316	0.086544
seq.25075.2	25075-2	Zinc finger and BTB domain-containing protein 10	ZBTB10	Q96DT7	0.047316	-0.08452
seq.3024.18	3024-18	Alpha-2-antiplasmin	SERPINF2	P08697	0.047316	-0.06786
seq.4535.50	4535-50	ADP-ribosyl cyclase/cyclic ADP-ribose hydrolase 2	BST1	Q10588	0.047316	-0.08371
seq.5128.53	5128-53	SLAM family member 6	SLAMF6	Q96DU3	0.047316	-0.17994
seq.6918.183	6918-183	Cholecystokinin	CCK	P06307	0.047316	-0.11684
seq.6931.10	6931-10	Cancer/testis antigen 1	CTAG1A	P78358	0.047316	-0.16734
seq.7754.11	7754-11	Protein jagged-1:Cytoplasmic domain	JAG1	P78504	0.047316	-0.10491
seq.8069.85	8069-85	T-cell surface glycoprotein CD3 epsilon chain	CD3E	P07766	0.047316	0.12164
seq.8262.20	8262-20	Apolipoprotein D	APOD	P05090	0.047316	-0.07094
seq.8272.22	8272-22	3-keto-steroid reductase:Cytoplasmic domain	HSD17B7	P56937	0.047316	-0.10357
seq.8984.28	8984-28	Leucine-rich repeat and calponin homology domain-containing protein 4:Leucine-rich repeat 5	LRCH4	O75427	0.047316	-0.11148
seq.10056.5	10056-5	Forkhead box protein M1	FOXM1	Q08050	0.047994	0.222252
seq.10507.166	10507-166	Acrosomal protein SP-10	ACRV1	P26436	0.047994	-0.14876
seq.10553.8	10553-8	Torsin-1A-interacting protein 2	TOR1AIP2	Q8NFQ8	0.047994	0.093993
seq.11365.17	11365-17	Teneurin-4	TENM4	Q6N022	0.047994	-0.09605
seq.11369.23	11369-23	Alcohol dehydrogenase class-3	ADH5	P11766	0.047994	0.325998
seq.12461.8	12461-8	NAD-dependent protein deacylase sirtuin-5, mitochondrial	SIRT5	Q9NXA8	0.047994	0.215624
seq.13132.14	13132-14	Semaphorin-5A	SEMA5A	Q13591	0.047994	0.413171
seq.16312.45	16312-45	Cadherin-6	CDH6	P55285	0.047994	0.163613
seq.18208.3	18208-3	G2/mitotic-specific cyclin-B2	CCNB2	O95067	0.047994	0.098233
seq.18376.19	18376-19	Myosin light chain 3	MYL3	P08590	0.047994	0.400087
seq.20427.18	20427-18	Mediator of RNA polymerase II transcription subunit 20	MED20	Q9H944	0.047994	0.113868
seq.21507.48	21507-48	COMM domain-containing protein 8	COMMD8	Q9NX08	0.047994	0.220914
seq.21670.52	21670-52	NEDD4-like E3 ubiquitin-protein ligase WWP2	WWP2	O00308	0.047994	0.133397
seq.22528.1	22528-1	PHD finger protein 11	PHF11	Q9UIL8	0.047994	0.168921
seq.23623.48	23623-48	Diphosphoinositol polyphosphate phosphohydrolase 3-beta	NUDT11	Q96G61	0.047994	0.339989
seq.23767.1	23767-1	UPF0705 protein C11orf49	C11orf49	Q9H6J7	0.047994	0.238389
seq.2732.58	2732-58	Homeobox protein NANOG	NANOG	Q9H9S0	0.047994	-0.08484
seq.4332.6	4332-6	C-type lectin domain family 1 member B	CLEC1B	Q9P126	0.047994	-0.09152
seq.4829.43	4829-43	14-3-3 protein sigma	SFN	P31947	0.047994	0.4725
seq.5669.26	5669-26	Signal-regulatory protein beta-2	SIRPB2	Q5JXA9	0.047994	0.411892
seq.6294.11	6294-11	B melanoma antigen 2	BAGE2	Q86Y30	0.047994	0.56792
seq.7141.21	7141-21	Alpha-1,3-mannosyl-glycoprotein 4-beta-N-acetylglucosaminyltransferase B	MGAT4B	Q9UQ53	0.047994	0.147133
seq.7180.114	7180-114	Interferon alpha-14	IFNA14	P01570	0.047994	-0.10139
seq.7249.307	7249-307	Bcl-2-like protein 10	BCL2L10	Q9HD36	0.047994	-0.08072
seq.9035.2	9035-2	MAX-interacting protein 1	MXI1	P50539	0.047994	-0.07768
seq.23248.1	23248-1	X antigen family member 2	XAGE2	Q96GT9	0.049343	0.23361
seq.2441.2	2441-2	Fibroblast growth factor 10	FGF10	O15520	0.049343	-0.10238
seq.10552.88	10552-88	NKG2-F type II integral membrane protein	KLRC4	O43908	0.049343	-0.1321
seq.11481.25	11481-25	Hepatitis A virus cellular receptor 2	HAVCR2	Q8TDQ0	0.049343	0.101582
seq.13557.3	13557-3	Nectin-3	NECTIN3	Q9NQS3	0.049343	-0.13026
seq.22810.41	22810-41	TIMELESS-interacting protein	TIPIN	Q9BVW5	0.049343	-0.15825
seq.3617.80	3617-80	Hepatocyte growth factor activator	HGFAC	Q04756	0.049343	-0.09196
seq.12451.62	12451-62	Transcription regulator protein BACH1	BACH1	O14867	0.049343	-0.03799
seq.18860.2	18860-2	Calcium/calmodulin-dependent protein kinase kinase 1	CAMKK1	Q8N5S9	0.049343	-0.08587
seq.23679.43	23679-43	Sister chromatid cohesion protein DCC1	DSCC1	Q9BVC3	0.049343	-0.06968
seq.25236.11	25236-11	Methyltransferase-like 26	METTL26	Q96S19	0.049343	-0.07071
seq.4429.51	4429-51	Carbohydrate sulfotransferase 6	CHST6	Q9GZX3	0.049343	-0.07733
seq.4785.30	4785-30	Corticosteroid-binding globulin	SERPINA6	P08185	0.049343	-0.19024
seq.7864.3	7864-3	Sialic acid-binding Ig-like lectin 8	SIGLEC8	Q9NYZ4	0.049343	-0.04909
seq.8232.90	8232-90	Junctional adhesion molecule-like	JAML	Q86YT9	0.049343	-0.09858
seq.8941.4	8941-4	Neuroligin-3	NLGN3	Q9NZ94	0.049343	-0.05113
seq.9486.13	9486-13	Beta-defensin 125	DEFB125	Q8N687	0.049343	-0.08833
seq.10365.132	10365-132	Interleukin-23	IL12B|IL23A	P29460|Q9NPF7	0.049343	-0.10578
seq.13701.2	13701-2	C-X-C motif chemokine 13	CXCL13	O43927	0.049343	-0.07809
seq.15431.31	15431-31	Ornithine carbamoyltransferase, mitochondrial	OTC	P00480	0.049343	-0.05827
seq.15558.63	15558-63	Glutamyl aminopeptidase	ENPEP	Q07075	0.049343	-0.1046
seq.20464.7	20464-7	Putative KHDC1-like protein	KHDC1L	Q5JSQ8	0.049343	-0.12931
seq.21124.17	21124-17	Yae1 domain-containing protein 1	YAE1	Q9NRH1	0.049343	0.134587
seq.23306.37	23306-37	Polyamine-modulated factor 1	PMF1	Q6P1K2	0.049343	0.126548
seq.24453.75	24453-75	Cytochrome c-type heme lyase	HCCS	P53701	0.049343	-0.07502
seq.2993.1	2993-1	Interleukin-18 receptor accessory protein	IL18RAP	O95256	0.049343	-0.13136
seq.3178.5	3178-5	Dipeptidyl peptidase 1	CTSC	P53634	0.049343	-0.07405
seq.3809.1	3809-1	Fibroblast growth factor receptor 3:Cytoplasmic domain	FGFR3	P22607	0.049343	-0.05395
seq.9580.5	9580-5	Laminin subunit gamma-2	LAMC2	Q13753	0.049343	0.105265
seq.9715.15	9715-15	Immunoglobulin superfamily member 3	IGSF3	O75054	0.049343	0.215892
seq.9850.38	9850-38	Eukaryotic translation initiation factor 1A, X-chromosomal	EIF1AX	P47813	0.049343	-0.06637
seq.11140.56	11140-56	Collagen alpha-1(I) chain:C-term propeptide	COL1A1	P02452	3.20E-06	2.91646

Table S3. Full aptamer (Somalogics) dataset.

Available for download at
https://journals.biologists.com/dmm/article-lookup/doi/10.1242/dmm.050487#supplementary-data
